# Intermodule Coupling Analysis of Huang-Lian-Jie-Du Decoction on Stroke

**DOI:** 10.3389/fphar.2019.01288

**Published:** 2019-11-05

**Authors:** Pengqian Wang, Li Dai, Weiwei Zhou, Jing Meng, Miao Zhang, Yin Wu, Hairu Huo, Xingjiang Xiong, Feng Sui

**Affiliations:** ^1^Institute of Chinese Materia Medica, China Academy of Chinese Medical Sciences, Beijing, China; ^2^Guang’anmen Hospital, China Academy of Chinese Medical Sciences, Beijing, China

**Keywords:** Huang-Lian-Jie-Du decoction, stroke, inter-module analysis, pharmacological mechanism, network pharmacology

## Abstract

Huang-Lian-Jie-Du Decoction (HLJDD) is a "Fangji" made up of well-designed Chinese herb array and widely used to treat ischemic stroke. Here we aimed to investigate pharmacological mechanism by introducing an inter-module analysis to identify an overarching view of target profile and action mode of HLJDD. Stroke-related genes were obtained from OMIM (Online Mendelian Inheritance in Man). And the potential target proteins of HLJDD were identified according to TCMsp (Traditional Chinese Medicine Systems Pharmacology Database and Analysis Platform). The two sets of molecules related to stroke and HLJDD were respectively imported into STRING database to construct the stroke network and HLJDD network, which were dissected into modules through MCODE, respectively. We analyzed the inter-module connectivity by quantify "coupling score" (CS) between HLJDD-modules (H-modules) and stroke-modules (S-module) to explore the pharmacological acting pattern of HLJDD on stroke. A total of 267 stroke-related proteins and 15 S-modules, 335 HLJDD putative targeting proteins, and 13 H-modules were identified, respectively. HLJDD directly targeted 28 proteins in stroke network, majority (16, 57.14%) of which were in S-modules 1 and 4. According to the modular map based on inter-module CS analysis, H-modules 1, 2, and 8 densely connected with S-modules 1, 3, and 4 to constitute a module-to-module bridgeness, and the enriched pathways of this bridgeness with top significance were TNF signaling pathway, HIF signaling pathway, and PI3K-Akt signaling pathway. Furthermore, through this bridgeness, H-modules 2 and 4 cooperatively work together to regulate mitochondrial apoptosis against the ischemia injury. Finally, the core protein in H-module 4 account for mitochondrial apoptosis was validated by an *in vivo* experiment. This study has developed an integrative approach by inter-modular analysis for elucidating the "shotgun-like" pharmacological mechanism of HLJDD for stroke.

## Introduction

Stroke is a complex disease featured by various genetic variations and dysfunction (Matthew et al., 2012). The subsequent mess brought by gene interactions and pathway crosstalk makes it difficult to obtain a “magic bullet” acting on “single gene, single target” to achieve therapeutic efficacy (Roth et al., 2004; Frantz, 2005; Hopkins, 2009; Wang et al., 2018). These observations, coupled with the increasing failure rate of drug discovery based on reductionism (Khanna, 2012) have led to calls for a new science of “network medicine” to find a multi-target therapy modulating multiple genes and their interactions (Chandra and Padiadpu, 2013; Wang and Wang, 2013; Harvey et al., 2015; Greene and Loscalzo, 2017). Chinese herbal medicines, known as concoctions of numerous chemical ingredients, have been suggested to act on multiple pharmacological targets and therefore drew increasing attention in the latest decades. “Fangji” was a well-designed Chinese herb array according to principle of traditional Chinese medicine, in order to improve therapeutic efficacy and/or reduce toxicity and adverse reactions (Wang et al., 2011; Wang et al., 2013; Duan et al., 2015; Liu and Wang, 2015). Systematic prediction of multiple drug–target interactions from chemical, genomic, and pharmacological data was expected to accelerate the drug discovery processes (Yu et al., 2012). This may provide a potential avenue to multi-target therapy reversing the disease condition. As amount and sheer diversity of high throughput data generated are enormous in the post-genomic age, it is pertinent to explore underlying pathogenesis and pharmacological mechanism by taking a more overarching view of Fangji multi-target therapies on stroke (Hasan et al., 2012; Gu and Chen, 2014; Huang et al., 2014; Li et al., 2017).

Huang-Lian-Jie-Du Decoction (HLJDD), also known as Hwangryun-Hae-Dok Decoction or oren-gedoku-to in Japan, is an ancient traditional Chinese formula first described in Wang Tao's "Wai Tai Mi Yao" 2,000 years ago. It is composed of four herbs: Coptidis Rhizoma (*Coptis chinensis* Franch., rhizome), Radix Scutellariae (*Scutellaria baicalensis* Georgi., radix), Phellodendri Chinensis Cortex (*Phellodendron chinense* Schneid., cortex), and Gardeniae Fructus (*Gardenia jasminoides* Ellis., fructus) with the ratio of 3:2:2:3. HLJDD was widely applied as a complementary and alternative medicine to treat cerebral ischemia in Asian countries (Kondo et al., 2000; Xu et al., 2000; Zhang et al., 2017; Fu et al., 2019). It is reported that HLJDD could reduce ischemia-reperfusion brain injury (Hwang et al., 2002) and promote functional recovery in stroke (Zou et al., 2016) by alleviating the oxidative stress from reactive oxygen species (ROS), ameliorating inflammatory damage, enhancing cortical neurogenesis, inducing protective autophagy, and so on (Wang et al., 2013; Wang et al., 2014; Zou et al., 2016). The ingredients from HLJDD were also studied for their anti-ischemia effect. For instance, baicalin, an ingredient from *Rhizoma Coptidis*, was reported to reduce ischemic infarct volume by regulating apoptotic and neurophysiological processes (Liu et al., 2017), to protect brains against hypoxic-ischemic injury *via* the PI3K/Akt signaling pathway (Zhou et al., 2017); jasminoidin from *Fructus Gardeniae* could attenuate inflammatory response by suppressing ERK1/2 signaling pathway in brain microvascular endothelial cells (Li et al., 2016). Berberine, baicalin, and jasminoidin are major ingredients responsible for the effectiveness of HLJDD by amelioration of abnormal metabolism and regulation of oxidative stress, neuron autophagy, and inflammatory response (Zhang et al., 2017), and acted synergistically to exert protective effects (Zhang et al., 2016). It is reported that both of baicalin and jasminoidin could attenuate ischemia/reperfusion injury by suppressing mitochondrial apoptosis (Jiang et al., 2016; Zhao et al., 2016; Li, et al., 2017). And the process of mitochondrial apoptosis is largely consequent on the translocation of Bax and Bak of the Bcl-2 family to the mitochondrial outer membrane (Love, 2003; Zhang et al., 2015). However, how the mitochondrial apoptosis is attenuated by the ingredients of HLJDD remains unsettled.

All the above researches enriched the pharmacological targets of HLJDD. However, the next challenge arising may be how to grab the specific targeting community and action mode of HLJDD in the biological network. As a complex adaptive system, biological network constitutes a set of interacting units, "modules," which are suggested to be minimum functional entities (Sales-Pardo, 2017). The rewiring of these interacting modules can bring out the nonlinear phenomena: chaotic behaviors, synchronization, emergence, and subsequent phenotype alteration (Bandyopadhyay et al., 2010; Tang et al., 2014). Therefore, the interactions between these modules, not only the modules themselves, should be investigated to elucidate process of biological network response to perturbation (Bandyopadhyay et al., 2010; Hsu et al., 2011), especially to drugs and multi-target therapies. Accordingly exploring the target-on modules of HLJDD and how they work together to execute sophisticated function causing phenotypic alteration might be a promising opportunity to clarify the pharmacological mechanisms of this multi-ingredient herb array.

**Figure 1:**
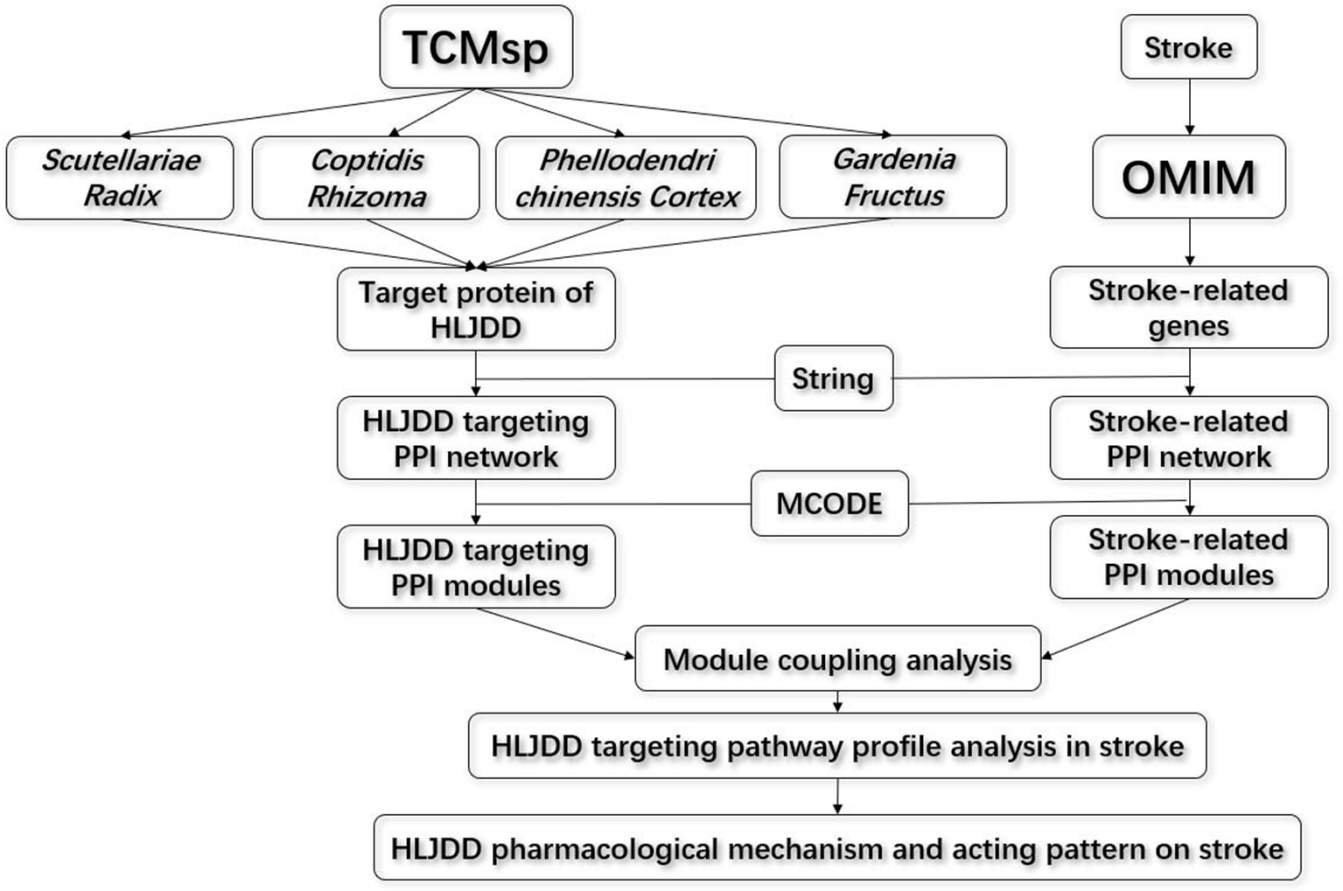
Flow chart for the inter-module coupling analysis strategy.

In this paper, we introduced the modularity analysis integrating inter-module connectivity calculation in protein to protein association network to identify the target profile and action mode of HLJDD (**Figure 1**). Firstly, we constructed the stroke-related network and HLJDD targeting protein network according to OMIM (Online Mendelian Inheritance in Man) databases and TCMsp (Traditional Chinese Medicine Systems Pharmacology Database and Analysis Platform), respectively. Then we dissected the two networks into modules, respectively. The stroke-modules (S-modules) and HLJDD-modules (H-module) were bridged by integrating the two-dimensional network based on protein–protein interaction background from STRING. Next, we analyzed the inter-module connectivity between S-modules and H-modules to explore the pharmacological acting pattern of HLJDD on stroke. Finally, we validated the conclusions by an *in vivo* experiment.

## Materials and Methods

### Stroke-Related Data Source and Network Construction

Genes related to stroke were derived from OMIM (https://www.ncbi.nlm.nih.gov/omim/), a database of human genes and genetic disorders. We searched "stroke" as a keyword in OMIM and filtered the records for gene variations. As a result, all of genes related to stroke were identified and mapped to the background in STRING, which is an online database for functional protein association networks (https://string-db.org/cgi/input.pl), providing associations between proteins based on curated databases, experimentally determined, gene neighborhood, gene fusions, gene co-occurrence, textmining, co-expression, or protein homology. Finally, based on the proteins that correspond to imported stroke genes and protein-to-protein interactions from STRING, proteins association network related to stroke was constructed, in which proteins were represented as vertex, and interaction confidence more than 0.4 (a relatively low confidence to catch the broader scope of proteins related to stroke) was set as the edge connecting corresponding proteins.

### HLJDD Potential Targets and Network Construction

The potential targets of herbs from HLJDD were obtained from TCMsp (http://lsp.nwu.edu.cn/tcmsp.phpm), which is a systems pharmacology platform of Chinese herbal medicines that captures the relationships between drugs and targets (Ru et al., 2014). For we aimed to construct the holistic target landscape of HLJDD, we included all of the ingredients in the HLJDD and all of the potential targets of these ingredients. Then potential targets of the four herbs were merged as the target protein of HLJDD. The union of the HLJDD potential targets was also mapped to STRING background. Therefore, these targets were regarded as vertex, and their interaction from STRING was used as the edge, to construct the HLJDD target network. The weight of edges was equal to the interaction confidence, which is a parameter to evaluate the associations between protein in STRING. The cutoff of the edges was set as 0.7, a mediate confidence to obtain herbs targets interactions with high reliability.

### Module Identification and Inter-Module Analysis

Both the stroke network and the HLJDD network were clustered to find stroke-modules (abbreviated as S-module) and HLJDD-modules (abbreviated as H-module), respectively, by MCODE (molecular complex detection). MCODE was a cluster algorithmic-based software in cytoscape, which can cluster a given network based on topology to find densely connected regions. Pathways of these two groups of modules were enriched according to KEGG database (Kyoto Encyclopedia of Genes and Genomes). All of enriched pathways were classified according to KEGG pathway maps (https://www.kegg.jp/kegg/pathway.html) to conduct the heterogeneity analysis. The connectivity between S-modules and H-modules was bridged by merging the stroke network and HLJDD network to constitute a disease-drug bi-dimensional network. The proteins related to disease or herbs and their interactions can be simplified as a pair of networks D = (V, E) and H = (V, E), representing the disease-related and herbs-related network. The two networks were merged based on the background of STRING database into a union graph, as G = (V, E), which contain the total of two sets of vertices and edges. Therefore, the S-modules and H-modules were all in G. The hypergeometric distribution was employed to calculate the significance of the interaction of a pair of modules.

(1) $$p=\sum_{k=x}^{n}\frac{\left(\frac{M}{k}\right)\left(\frac{N-M}{n-k}\right)}{\left(\frac{N}{n}\right)}$$

where x is observed inter-module connection; k and n represent the numbers of inter-module connections and all possible interactions between the pair of modules, respectively; M and N were the total numbers of inter-module connections and all probably existing inter-module interactions between any two modules in a network, respectively. We set *P ≤* 0.05 as significant. If the P-value of an inter-module connection was less than 0.05, the inter-module connectivity will be quantified by a novel parameter: coupling score (CS), which were introduced to evaluate the inter-module connectivity mediated by nodes and edges. The CS between any two modules was defined as follows:

(2) $$\text{CS}=\text{2t+}\sum_{i\in M_{x},j\in My}a_{\mathrm{ij}}$$

where *M_x_* and *M_y_* denote a disease-module and herb-module connected by at least one edge; t is the total number of overlapping nodes between *M_x_* and *M_y_*. The symbols *i* and *j* represent a gene in *M_x_* and *M_y_*, respectively; *a_ij_* is the weight of edge between genes *i* and *j*. Accordingly, based on this score, the modular map was constructed to include all inter-module connectivity relationships.

### *In Vivo* Experiment Validation

To further validate our conclusion, we employed the middle cerebral artery obstruction (MCAO) animal model to examine the main ingredients' effect on the protein related to new identified mechanism of HLJDD. All the animal experiments were approved by the Ethics Committee of China Academy of Chinese Medicine. The experimental procedures were in accordance with the Prevention of Cruelty to Animals Act 1986 and NIH Guidelines for the Care and Use of Laboratory Animals for Experimental Procedures (National Research Council (US) Institute for Laboratory Animal Research, 1996).

A total of 24 Sprague-Dawley (SD) rats, weighing 200–220 g, were subjected to MCAO in order to induce a focal cerebral ischemia-reperfusion model. All the rats, except those in the sham-operated group, were subjected to MCAO procedure, according to the method described by Longa et al. (1989). Brieﬂy, after being anesthetized with 2% pentobarbital (4 mg/kg, ip), the rats were exposed and the external carotid artery (ECA) was prepared, and an intraluminal filament was inserted from the ECA to ligate the left middle cerebral artery for 1.5. Then the intraluminal filament was withdrawn for reperfusion for 24 h. Rats in the sham group were also subjected to the same surgical preparation for the insertion of the filament as other groups, but no filament was inserted.

The standards of two major ingredients of HLJDD, baicalin (BA) and jasminoidin (JA), were obtained from the National Institutes for Food and Drug Control, and the purity was validated by fingerprint chromatographic methodologies. All compounds were dissolved in 0.9% saline just before the experiment. The rats were randomly divided into four groups: sham-operated group (0.9% saline), vehicle group (0.9% saline), BA (5 mg/ml)-treated group, and JA (25 mg/ml)-treated group in this study. After the reperfusion, the rats received the responding treatment by intraperitoneal injection at 2 ml/kg body weight. After 24 h reperfusion and treatment, the rats were sacrificed, and the hippocampi of these rats were removed for western blotting.

The hippocampi were homogenized. After protein extraction and concentration adjustment, proteins were separated by sodium dodecyl sulfate (SDS)–polyacrylamide gel electrophoresis (PAGE) and transferred to nitrocellulose membranes (Hybond-C, Amersham, Buckinghamshire, UK) by electroblotting. Blots were stained with rabbit anti-Bak (Santa Cruz Biotechnology, Santa Cruz, CA, USA) and anti-β-actin (Abcam, Cambridge, UK) at a concentration of 1:1,000 and 1:5,000, respectively. After cyclic membrane wash and staining by goat anti-rabbit IgG with chemiluminescence (Amersham), the band density was determined with a GS-700 densitometer (Bio-Rad). Each measurement was taken in three replicates.

## Results

### Two S-Module Community With Diverse Functions

**Figure 2:**
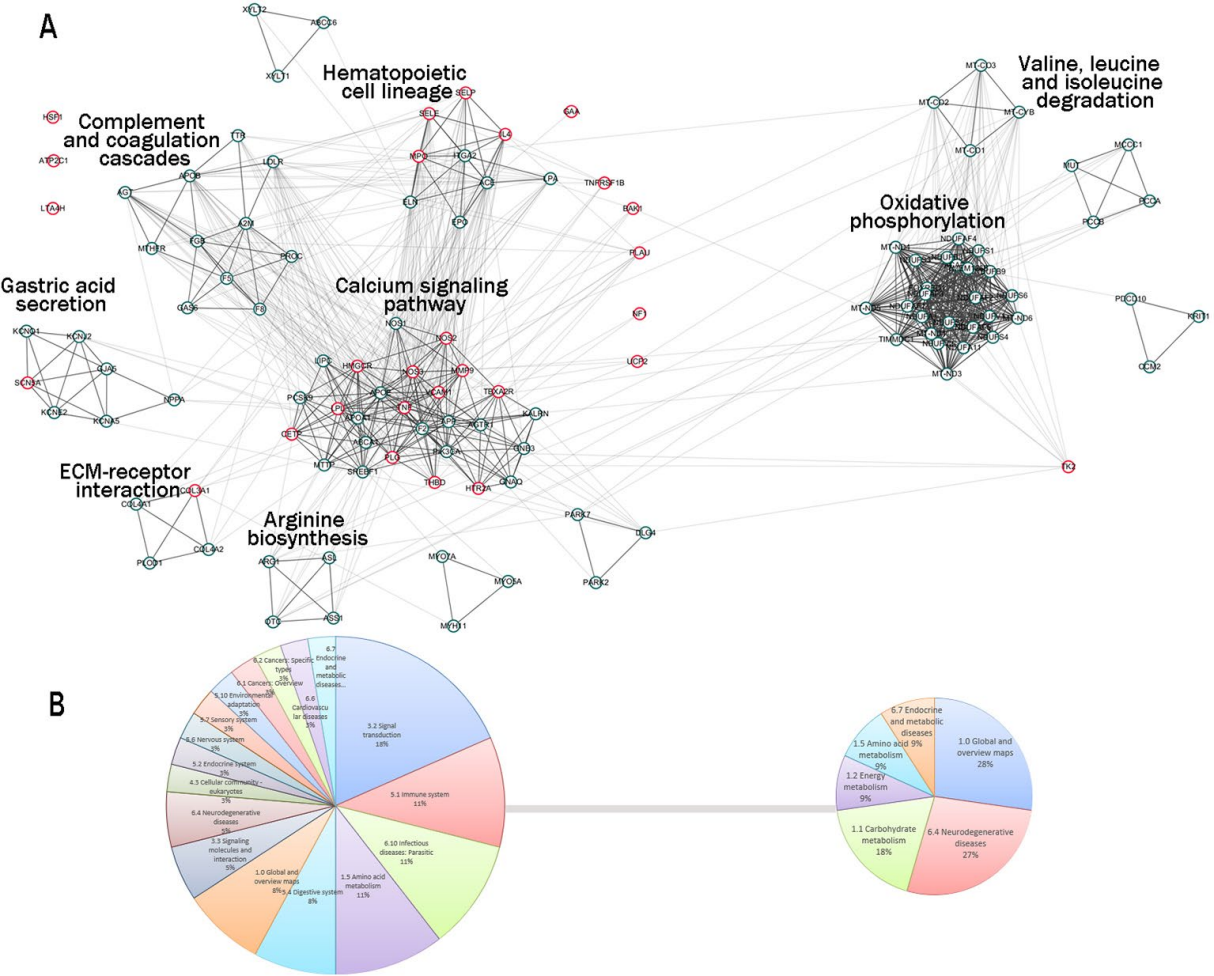
The S-module communities and enrichment KEGG pathways. **(A)** The S-module communities. A total of 13 S-modules constituted two communities: the first was centered by S-module 1; the other was centered by S-module 2. The circles represented the proteins related to stroke; the circles marked by red were S-module proteins targeted by HLJDD. Representative enriched KEGG terms with minimum P-value are used for the annotation of each S-module. **(B)** The categories of enriched KEGG pathways of the two S-module communities. The categories of community 1 concentrated on signal transduction, immune system, infectious diseases: parasitic, and amino acid metabolism; community 2 focused on global and overview maps, neurodegenerative diseases, and carbohydrate metabolism.

As a result, 303 genes related to stroke were identified. A total of 267 out of 303 genes were found corresponding to proteins. The official symbols, domains, and annotations of the 267 proteins are shown in **Supplementary Table 1**.And 256 in 267 proteins were involved in the stroke-related network (named as stroke network), and the other 11 proteins were distributed individually; 1,502 edges were included in the network. According to MCODE, the stroke network was divided into 15 S-modules and many individual nodes (**Supplementary Figure 1**). The S-modules were interacting with each other to constitute a module map. S-module 1 and S-module 2 were in the center of the module map and possessed most neighbor modules: S-module 1 densely associated with S-modules 3, 4, 5, and 7; S-module 2 was densely interacting with S-module 10. The two module groups, led by S-module 1 and S-module 2, constituted two communities of stroke network. The other S-modules were sparsely connected (**Figure 2A**).

**Figure 3:**
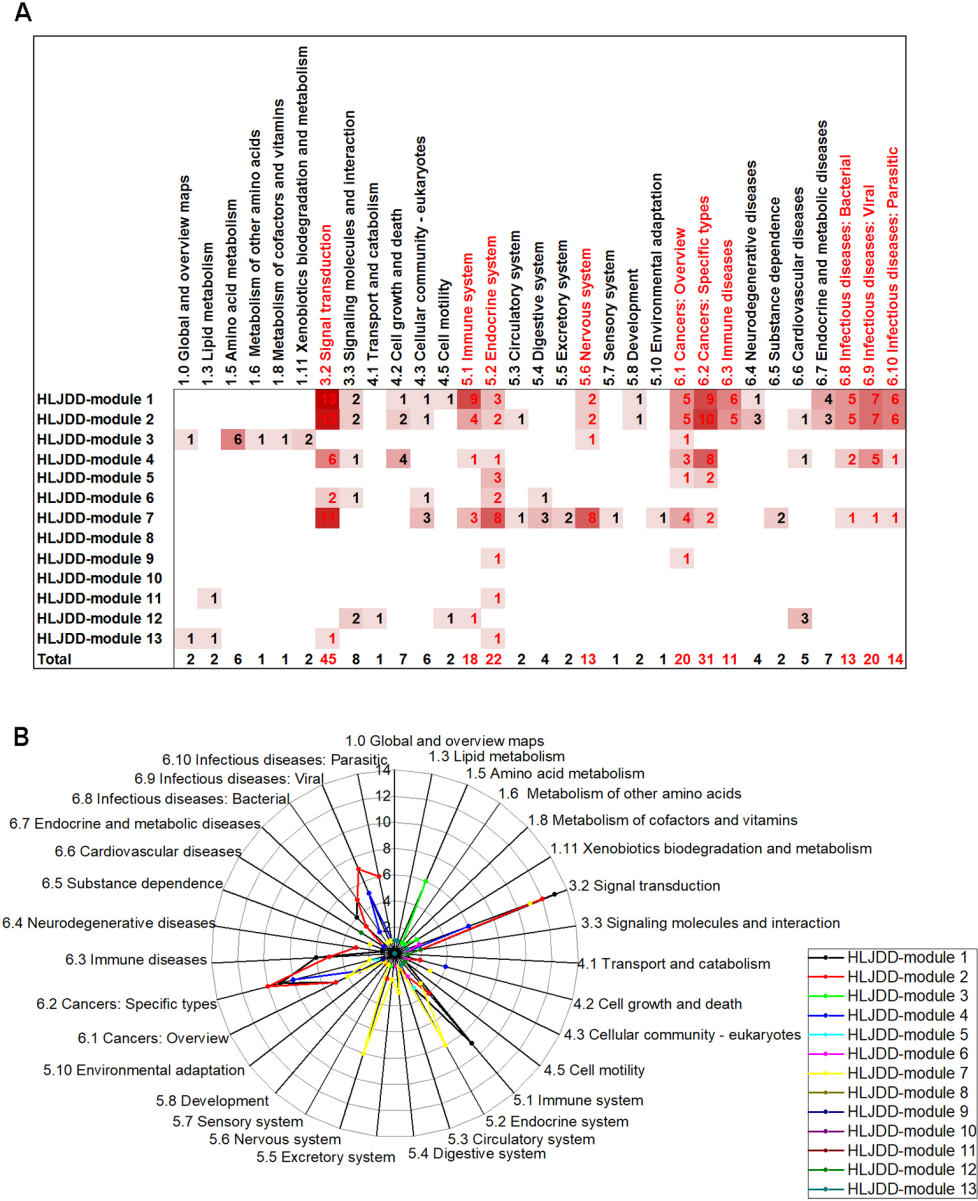
The number and distribution of enriched pathway of H-modules in each category. **(A)** The number of enriched pathways of H-modules in categories. The depth of the color was in proportion to the number of pathways. The letters emphasized by red represented categories with high enrichment frequency. **(B)** The radar chart of categories of pathways. The length of the line in each point position showed the number of the pathways in correspondent categories. The categories circled by red rings were frequently enriched.

To investigate the biological function of S-modules, the KEGG pathway enrichment was conducted (**Supplementary Table 2**). As the center of the two community, S-module 1 and S-module 2 were enriched for 21 and 6 signaling pathways, respectively. The representative enriched annotation term with minimum P-value of S-module 1 was the calcium signaling pathway, and the representative term of S-module 2 was oxidative phosphorylation (**Figure 2A**). Additionally, there were 1, 3, 2, 6, 5, and 5 signaling pathways enriched in S-modules 3, 4, 5, 7, 8, and 9, respectively. Furthermore, the enriched pathways of each S-module were classified. According to the categories of S-modules, the functions of two modular communities varied from each other. For the modular community 1, the top 4 pathway categories were signal transduction, immune system, and infectious diseases: parasitic and amino acid metabolism, accounting for 19%, 11%, 11%, and 11% of the total number of enriched pathways, respectively. These four sections accounted for 52% of the total pathways. For the modular community 2, categories showed more concentrated state: the top 3 pathway categories were global and overview maps, neurodegenerative diseases, and carbohydrate metabolism, accounting for 28%, 27%, and 18% of total enriched pathways. These three categories accounted for 73% of total pathways. Therefore, the pathological functions of the communities were on different aspects (**Figures 2B** and **3A**).

### H-Modules Mainly Regulated Signal Transduction, Immune, Cancer, Infectious Diseases, Nervous System

As collected from the TCMsp database, a total of 105, 35, 102, and 66 compounds and 234, 228, 288, and 310 target proteins of *Rhizoma Coptidis, Radix Scutellariae, Cortex Phellodendri*, and *Fructus Gardeniae* were identified respectively, and listed in **Supplementary Table 3**. After the target merging, there were 400 proteins that were regarded as the potential targets of HLJDD, and 335 out of 400 proteins were found as annotation in the STRING database (**Supplementary Table 4**). As a result, the ultimate HLJDD target network concluded with 300 proteins and 2,775 interactions. To investigate the targeting position in the intra-structure of this network, we also dissected the HLJDD network into modules. A total of 13 H-modules were identified by MCODE and 133 individual proteins not belonging to any module (**Supplementary Figure 1**).

According to KEGG pathway enrichment analysis of the H-modules (**Supplementary Table 5**), the most signaling pathways (76 pathways with significance) were enriched in H-module 1, among which the representative pathway with minimum P-value was TNF signaling pathway. In H-module 2, 72 pathways were enriched, and the minimum P-value pathway was cell cycle. H-modules 1 and 2 were concentrated on signal transduction, immune, cancer, and infectious diseases. The pathways related to the above aspects accounted for 81.58% and 77.78% of the total in H-modules 1 and 2, respectively. Additionally, 13, 33, 6, 7, 53, 2, 2, 8, and 4 pathways were enriched for H-modules 3, 4, 5, 6, 7, 9, 11, 12, and 13, respectively. These pathways were also categorized based on the KEGG pathway maps (**Figures 3A, B**). It is showed that the function of H-modules mainly concentrated on five aspects: signal transduction, immune, cancer, infectious diseases, and nervous system. It is also remarkable that the frequently enriched category was signal transduction, which may indicate the principal function of HLJDD.

### Overlapping Proteins Between HLJDD and Stroke Network Were the Direct Targets

As it is aimed to provide the targeting basis of HLJDD in stroke, we merged the HLJDD network and stroke network to construct the disease-drug bi-dimensional network. This merged network contained 515 proteins and 4,416 interactions, in which S-modules and H-modules were all involved. There were 28 overlapping proteins between stroke-related proteins and HLJDD targeting proteins. This may indicate that the 28 proteins are the potential targets of HLJDD, and the action mode of herbs was to directly regulate the disease gene. In the stroke network, nearly a half (12, accounting for 42.86%) of the 28 overlapping were located in S-module 1; 4 proteins were in S-module 4; and 1 protein was distributed in S-modules 5 and 7, respectively; the other 10 proteins were scattered around S-modules. This may indicate that S-module 1 and its neighbor S-module 4 were the major direct targets of HLJDD (**Figure 2A**), and the direct targets of HLJDD on stroke are mostly involved in stroke community 1 rather than stroke community 2.

**Figure 4:**
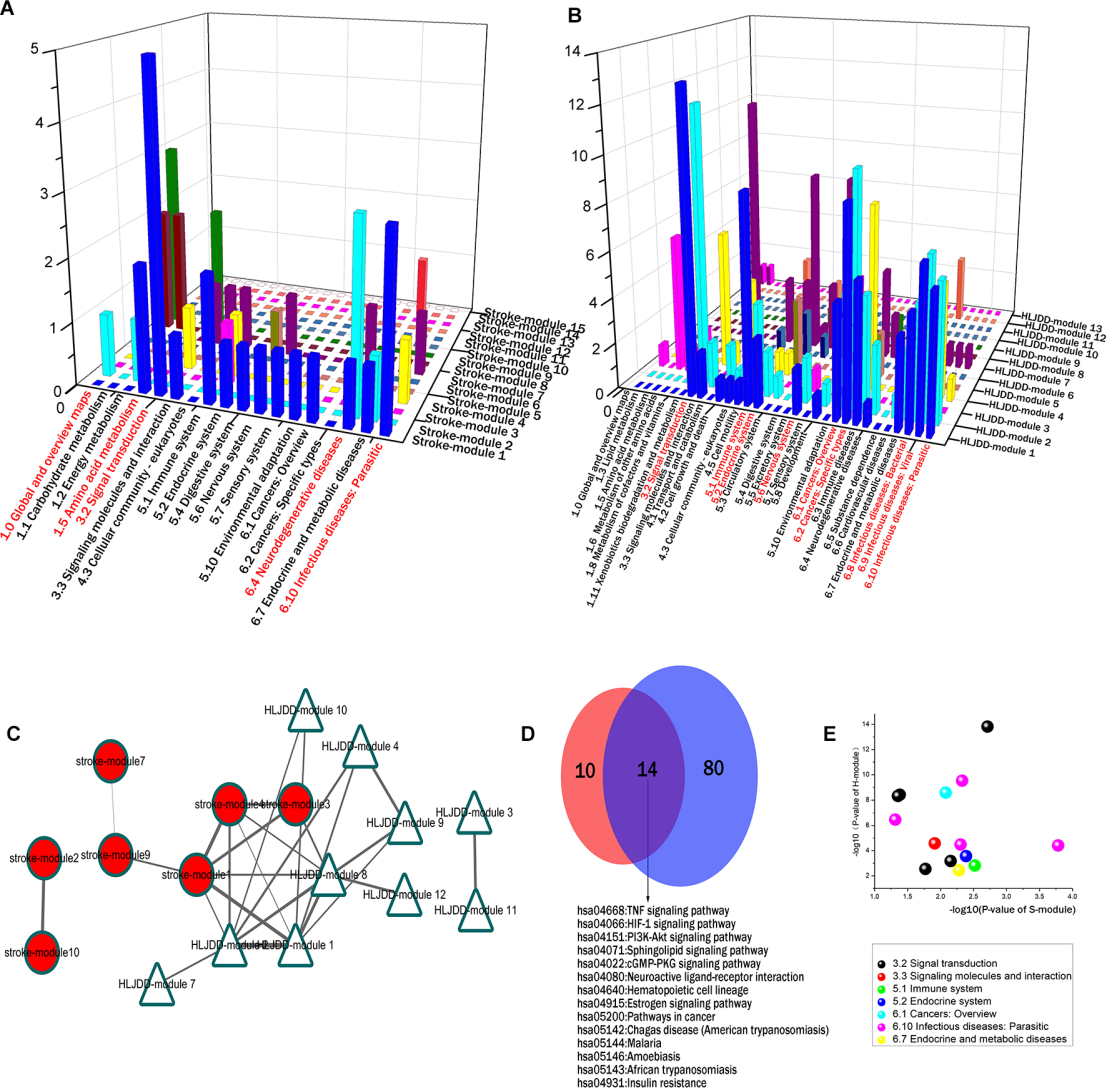
The inter-module coupling connectivity analysis and pathway comparison between S-modules and H-modules. **(A** and **B)** were the pathway categories of S-modules and H-modules, respectively. The categories marked by red were major ones with large proportion. **(C)** The modular map constituted by S-modules and H-modules based on inter-module analysis. H-modules 1, 2, and 8 densely connected with S-modules 1, 3, and 4 to constitute a module-to-module coupling connectivity bridge. **(D)** The overlapping situation of enriched pathways between S-modules 1, 3, and 4 and H-modules 1, 2, and 8 in this bridgeness. **(E)** The P-value of the overlapping pathways between S-modules 1, 3, and 4 and H-modules 1, 2, and 8. X-axis and Y-axis were representative for the -log10 of p-value in S-modules1, 3, and 4 and H-modules 1, 2, and 8, respectively.

Integrating with the pathway categories of stroke S-modules 1 and 4 in the above sections (**Supplementary Table 2**), we can infer that the priority direct regulation of HLJDD relies on the effect on signal transduction, immune, and infectious diseases, especially the signal transduction. For example, 24% pathways of S-module 1 focus on signal transduction, including calcium signaling pathway, TNF signaling pathway, sphingolipid signaling pathway, cGMP-PKG signaling pathway, and HIF-1 signaling pathway. This may be partly accounted for the pharmacological mechanism of HLJDD on stroke (**Figures 4A, B**).

### Inter-Module Connectivity Between H-Modules and S-Modules Bridged More Holistic Target Profile

HLJDD, which as a formula constituted of multiple herbs and numerous ingredients, may act more like the "magic shotguns" mode. As the cellular components were organized in a wide interaction pattern to achieve mutual information propagation, these "shotguns" of HLJDD may affect the stroke network not only by overlapping targets but also by perturbing the fluctuation of this biological adaptive system. Therefore, we employ the inter-module CS to explore a more holistic landscape of the HLJDD action mode.

According to the modular map based on inter-module CS, we have also noticed that several H-modules formed an inter-module coupling connectivity with S-modules. In the modular map, H-modules 1, 2, 4, 7, 8, 9, 10, and 12 were densely connected with S-modules. H-modules 1, 2, and 8 surrounded the major targeted S-modules 1, 3, and and 4 to constitute a module-to-module coupling connectivity to bridge formula-related and disease-related network as a potential targeting pattern (**Figure 4C**).

In this module-to-module bridgeness, by comparing the pathways of S-modules 1, 3, and 4 with H-modules 1, 2, and 8, a total of 14 overlapping pathways were found. These 14 pathways mainly focused on signal transduction (42.86%) and infectious diseases (28.57%), as shown in **Table 1** and **Figures 4D, E**. The other pathways belong to the immune system, endocrine system, metabolic diseases, and cancers. Among these pathways, the TNF signaling pathways were enriched with a minimum p-value (1.52E-14). That also verified that these peripheral H-modules included the same pathways with S-modules; that means, HLJDD regulated these pathological pathways of stroke by forming the module-to-module bridgeness in the biological system.

**Table 1:** The enriched KEGG pathways and corresponding proteins of H-module 1, 2, 8 and S-module 1, 3, 4 in the bridgeness structure.

KEGG pathway	Category	P-value of H-module	Genes of H-module	H-module	S-module	P-value of S-module	Genes of S-module
hsa04668: TNF signaling pathway	3.2 Signal transduction	1.52E-14	ICAM1, CSF2, IL6, TNF, CCL2, PTGS2, RELA, MMP9, CXCL2, EDN1, CXCL10, MAPK1, FOS, JUN	H-module 1, 2	S-module 1	0.001942	NOS1, NOS3, NOS2
hsa04066: HIF-1 signaling pathway	3.2 Signal transduction	3.85E-09	MAPK1, IL6, INS, RELA, BCL2, EDN1, VEGFA, IFNG, TLR4, STAT3	H-module 1, 2	S-module 1	0.04152	PIK3CA, NOS3, NOS2
hsa04151: PI3K-Akt signaling pathway	3.2 Signal transduction	4.75E-09	IL4, IL6, IL2RA, RELA, TP53, TLR4, BCL2L1, MAPK1, INS, CHRM2, BCL2, VEGFA, FGF2, MYC, IL2	H-module 1, 2	S-module 4	0.044181	IL4, ITGA2, EPO
hsa04071: Sphingolipid signaling pathway	3.2 Signal transduction	6.60E-04	MAPK1, TNF, RELA, BCL2, TP53, OPRD1	H-module 1, 2	S-module 1	0.00702	TNF, GNAQ, PIK3CA, NOS3
hsa04022: cGMP-PKG signaling pathway	3.2 Signal transduction	0.002805	MAPK1, INS, ADRA2A, ADRA2C, ADRA2B, OPRD1	H-module 1, 2	S-module 1	0.016942	AGTR1, GNAQ, PIK3CA, NOS3
hsa04080: Neuroactive ligand-receptor interaction	3.3 Signaling molecules and interaction	2.66E-05	OPRM1, PTGER3, CHRM4, C5AR1, DRD3, CHRM2, ADRA2A, ADRA2C, ADRA2B, OPRD1	H-module 1	S-module 1	0.012249	AGTR1, F2, TBXA2R, PLG, HTR2A
hsa04640: Hematopoietic cell lineage	5.1 Immune system	0.001527	IL4, CSF2, IL6, TNF, IL2RA	H-module 1	S-module 4	0.003017	IL4, ITGA2, EPO
hsa04915: Estrogen signaling pathway	5.2 Endocrine system	2.72E-04	OPRM1, MAPK1, FOS, JUN, MMP9, MMP2	H-module 1, 2	S-module 1	0.004103	GNAQ, MMP9, PIK3CA, NOS3
hsa05200: Pathways in cancer	6.1 Cancers: Overview	2.63E-09	IL6, PTGER3, PTGS2, RELA, MMP9, TP53, BCL2L1, MMP2, STAT3, MAPK1, FOS, JUN, BCL2, VEGFA, FGF2, MYC	H-module 1, 2	S-module 1	0.00833	AGTR1, GNAQ, MMP9, PIK3CA, GNB3, NOS2
hsa05142: Chagas disease (American trypanosomiasis)	6.10 Infectious diseases: Parasitic	2.93E-10	MAPK1, FOS, IL6, TNF, CCL2, JUN, RELA, IFNG, TLR4, IL10, IL2	H-module 1, 2	S-module 1	0.004712	TNF, GNAQ, PIK3CA, NOS2
hsa05144: Malaria	6.10 Infectious diseases: Parasitic	3.46E-07	ICAM1, IL6, TNF, CCL2, IFNG, TLR4, IL10	H-module 1, 2	S-module 4	0.048615	SELP, SELE
hsa05146: Amoebiasis	6.10 Infectious diseases: Parasitic	3.29E-05	CSF2, IL6, TNF, RELA, IFNG, TLR4, IL10	H-module 1, 2	S-module 1	0.004971	TNF, GNAQ, PIK3CA, NOS2
hsa05143: African trypanosomiasis	6.10 Infectious diseases: Parasitic	3.86E-05	ICAM1, IL6, TNF, IFNG, IL10	H-module 1, 2	S-module 1	1.65E-04	VCAM1, TNF, APOA1, GNAQ
hsa04931: Insulin resistance	6.7 Endocrine and metabolic diseases	0.003664	IL6, TNF, INS, RELA, STAT3	H-module 1, 2	S-module 1	0.005237	SREBF1, TNF, PIK3CA, NOS3

Furthermore, through this bridgeness, more H-modules were connected to more S-modules: H-modules 4, 7, 9, 10, and 12 could interact with S-modules through this bridgeness to constitute a more complete target profile on the disease network. For instance, viral carcinogenesis was a pathway enriched in H-module 2, involving the protein BAX. And there are also 12 proteins in H-module 4, one of which was Bak_1_, enriched in this pathway. Therefore, these H-modules could work together cooperatively to constitute a more comprehensive target profile.

## Western Blot Validation

To further validate the mechanism of HLJDD identified by inter-module coupling analysis, we selected a protein (Bak_1_) in H-module 4, which may act on S-modules by module-to-module bridgeness, and we employed western blot assays to compare the untreated and treated groups.

**Figure 5:**
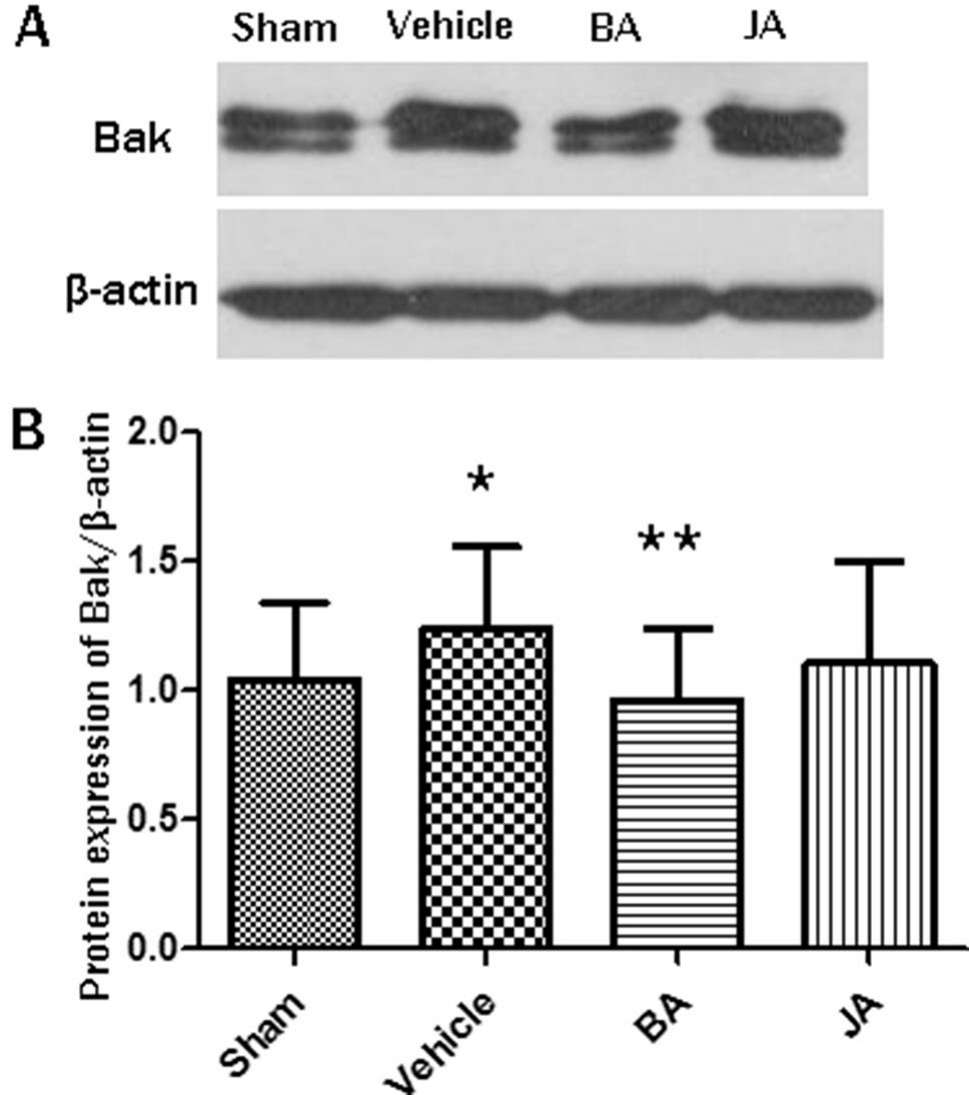
*In vivo* experiment validation of the protein involved in mechanism of HLJDD. **(A)** is the blot of Bak and β-actin; **(B)** are the expression levels of Bak/β-actin among different groups in Western blot; *P < 0.05, **P < 0.01 by one-side paired T-test.

According to western blot, as shown in **Figures 5A, B**, the expression of protein Bak in hippocampi significantly increased in the vehicle group compared with the sham group (paired T-test, one-sided, *P* < *0.05*). Its expression significantly decreased in BA groups compared with the vehicle group (paired T-test, one-sided, *P* < *0.01*). There was no statistical significance between JA and the vehicle group.

## Discussion

HLJDD, as a "fangji" formed by herb array, consists of numerous multi-target ingredients. Therefore, the targets of HLJDD were neither individual gene or protein, nor a single module, but "shotgun-like" target profiles. In this paper, we explored the H-modules, as well as drug- and disease-module inter-module coupling connectivity in stroke to investigate multiple targeting pathways of HLJDD and how they work together to cause phenotypic alteration.

### S-Modules 1 and 4 Were the Core Pathological Module Targeted by HLJDD

**Figure 6:**
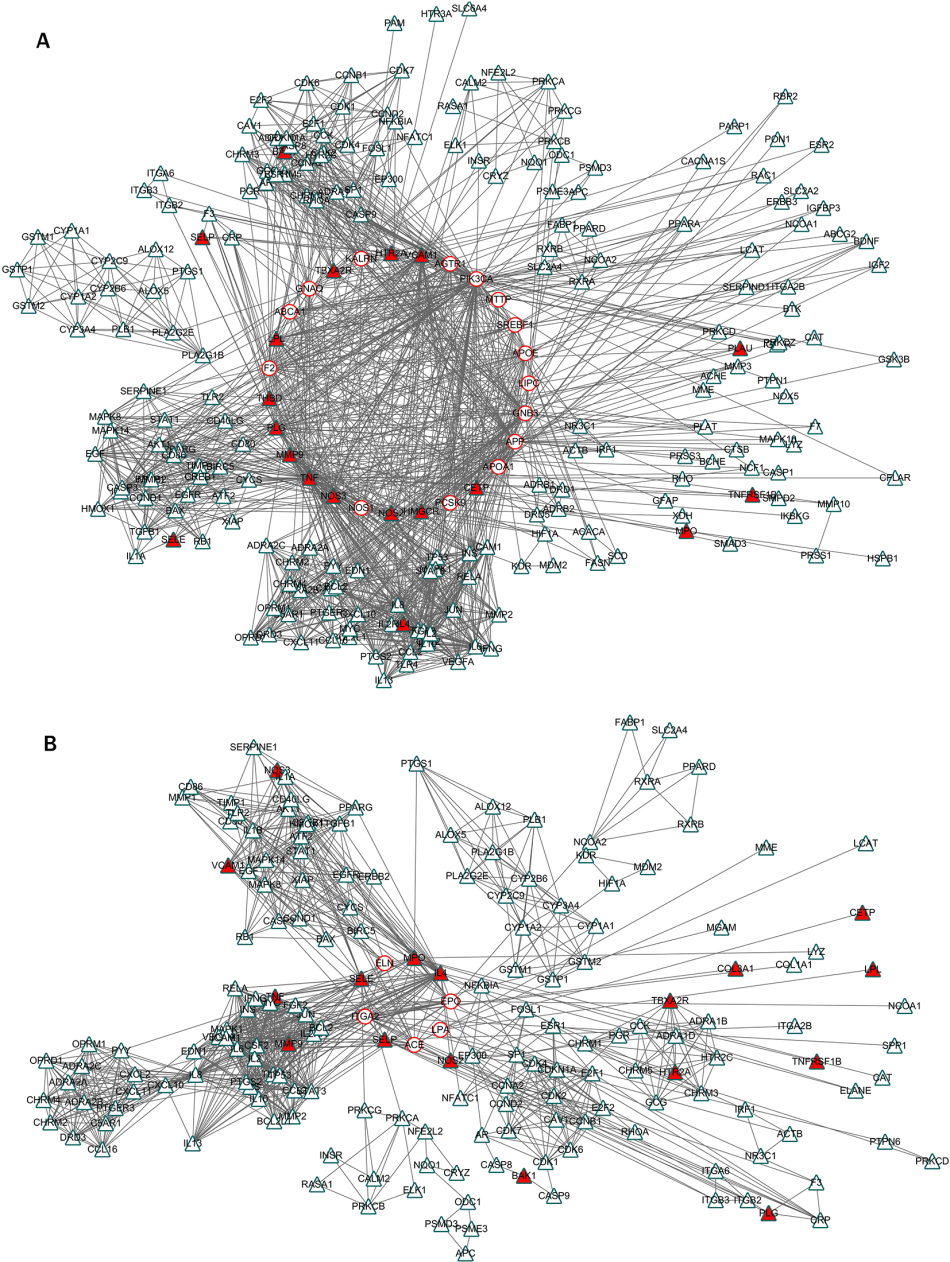
The inter-module connectivity between S-modules 1 and 4 and H-modules. **(A)** The inter-module coupling connectivity between S-module 1 and H-modules. The red circle and blue triangle represented the proteins related to stroke and HLJDD, respectively. The triangles fulfilled with red represented the proteins related to stroke and regulated by HLJDD. **(B)** The coupling connectivity between S-module 4 and H-modules.

In our stroke network, the S-module 1 was in the center of the modular map, circled by S-modules 3, 4, 5, and 7, and so on. Majority of the direct targets of HLJDD was distributed in S-modules 1 and 4. And the modular map also showed that the S-modules 1 and 4 were surrounded by H-modules (**Figure 6**). Therefore, S-modules 1 and 4 were the core targets regulated by HLJDD. Among the enriched pathways of S-module 1, the most frequently enriched category signal transduction, including calcium signaling pathway, TNF signaling pathway, sphingolipid signaling pathway, cGMP-PKG signaling pathway, and HIF-1 signaling pathway, exhibited a close relationship with stroke. All the above pathways showed close relationship with the stroke process. For example, the calcium signaling plays a critical role in the inflammation of stroke, associated with immune- and injury-related functions of astrocyte (Hamby et al., 2012). Ca^2+^ signaling showed beneficial effects on neuronal and brain protection and functional deficits after stroke (Li et al., 2015). TNF signaling is one of the key players in stroke inflammation progression: inhibition of TNF signaling can rescue functional cortical plasticity impaired in early post-stroke period (Liguz-Lecznar et al., 2015). HIF-1α signaling, which was involved in necroptosis, modulated blood–brain barrier integrity after focal ischemia (Geng et al., 2017). Sphingosine-1-phosphate, a key signaling molecule in the sphingolipid signaling pathway, is critical for sequelae after stressful stimulations: regulating glial cell activation, vasoconstriction, endothelial barrier function, and neuronal death pathways, which act as important components in many neurological conditions. Activation of sphingosine-1-phosphate receptor-1 by FTY720, a known sphingosine 1-phosphate receptor agonist, is neuroprotective after ischemic stroke in rats (Rosen et al., 2007; Hasegawa et al., 2010; Pfeilschifter et al., 2010; Maceyka and Spiegel, 2014; Prager et al., 2015; Sun et al., 2016). For pathways of stroke, S-module 4 also exhibited a close relationship to stroke. Also setting the signal transduction category as an example, the PI3K-Akt signaling pathway was enriched in S-module 4. It is reported that regulating PI3K/Akt signaling may induce ischemic damage attenuation in cerebral artery occlusion. In summary, the core pathological modules targeted by HLJDD were S-modules 1 and 4, which mainly include signal transductions related to neuroprotective effect.

### Modular Connectivity Revealed the “Shotgun-Like” Action Pattern of HLJDD

S-modules 1, 3, and 4 and surrounding H-modules 1, 2, and 8 constitute a bridging relationship between disease network to herb network. Among the overlapping pathways of this bridge structure, TNF signaling pathway was the top pathway with the most statistical significance. The “shotgun-like” action was exhibited in the TNF signaling pathway: four proteins (VCAM1, TNF, MMP9, and PIK3CA) in the S-module 1 were found in the TNF signaling pathway downstream; and 14 and 9 proteins of H-modules 1 and 2 were also found not only in the downstream, but also in the upstream of this pathway. The H-modules 1 and 2 constitute a comprehensive targeting set to regulate this pathway. This pathway was suggested to play a role in cerebral ischemia and impaired functional cortical plasticity and to be a primary process of releasing of inflammatory cytokines (Liguz-Lecznar et al., 2015; McCoy and Tansey, 2015; Liu, et al., 2016; Hollander et al., 2017; Wang et al., 2017).

Another pathway with top statistical significance was HIF-1 signaling pathway, which was enriched in S-module 1 and H-modules 1 and 2. A total of 17 target proteins in H-modules 1 and 2 belong to the HIF-1 signaling pathway, including MAPK1, IL6, INS, RELA, BCL2, EDN1, VEGFA, IFNG, TLR4, STAT3, AKT1, EGFR, ERBB2, SERPINE1, NOS3, EGF, and TIMP, which were overlapping with three proteins in S-module 1 in this pathway, PIK3CA, NOS3, and NOS2. And the targets of H-modules 1 and 2 contained the downstream and upstream of the stroke-related proteins in this pathway. It has been reported that HIF-1 played an important role in the antioxidant's neuroprotection in ischemic stroke (Zhang et al., 2014). HIF-1α can be served as an upstream regulator of cerebral glycerol concentrations and brain edema (Higashida et al., 2011). That means HLJDD regulated the pathological proteins and neighboring proteins closely related to stroke process to constitute a targeting network in the HIF signaling pathway.

Other significant pathways were also regulated by HLJDD by the similar action mode. Activation of the PI3K-Akt pathway was reported to promote neuroprotection against cerebral ischemia-reperfusion injury by decreasing nerve cell apoptosis (Lv et al., 2017; Li et al., 2019). The regulation mode on these overlapping pathways may provide a characteristic action pattern of multiple target ingredients, like many "shotguns" to form a regulating pathway profile, mainly concentrating on inflammatory response, antioxidant, and apoptosis.

Besides these overlapping pathways, the specific pathway of S-module 1 also has crosstalk with H-module enriched pathways. For example, inflammatory mediator regulation of TRP channels was a specific pathway in S-module 1. And this pathway can be regulated by MAPK signaling and calcium signaling, which were pathways in H-module 1. Therefore, the HLJDD regulated the upstream pathways of the stroke pathological pathway through the pathway crosstalk. Above all, HLJDD regulated the stroke-related core pathological pathways as well as their upstream and/or downstream pathways to constitute the waterfall of the pathway and to contribute a therapeutic effect.

Furthermore, through the bridgeness structure constituted by S-modules 1, 3, and 4 and H-modules 1, 2, and 8, more H-modules worked cooperatively on S-module. For instance, both the protein Bak_1_ in H-module 4 and BAX in H-module 2 were enriched in the viral carcinogenesis pathway. It is demonstrated that BAX and BAK are required for the initiation of apoptosis at the mitochondria (Ren et al., 2010). It is reported that the oligomerization of the Bax and Bak is an irreversible step leading to the execution of apoptosis, and inhibition of Bax/Bak oligomerization allowed cells to evade apoptotic stimuli and rescued neurons from death after excitotoxicity (Niu et al., 2017). Previous studies have demonstrated that both the ingredients baicalin and jasminoidin extracted from HLJDD could suppress mitochondrial apoptosis induced by ischemia/reperfusion injury, but the exact mechanism involved in these core proteins was far from clear. In our *in vivo* experiment, BA inhibited the protein expression Bak. This suggested that regulating mitochondrial apoptosis by inhibiting the Bak expression is an important mechanism of HLJDD in protecting against the neuronal injury. Therefore, through this module-to-module bridgeness, H-modules 2 and 4 cooperatively work together to regulate more comprehensive aspect of this pathway related to mitochondrial apoptosis against the ischemia injury. This also supported that it is the alteration of interaction between proteins from different H-modules that contributed to the phenotype reversion rather than a single target or a single module.

Accordingly, the core pathological modules were S-modules 1 and 4. We can infer that it is formatting an inter-module coupling connectivity between H-modules and stroke pathological modules, which contributed to pharmacological mechanism of HLJDD in stroke, mainly involving the TNF signaling pathway, the HIF signaling pathway, and the PI3K-Akt signaling pathway. Furthermore, through this bridgeness, H-modules 2 and 4 cooperatively work together to regulate mitochondrial apoptosis against the ischemia injury. These regulation targets were not a simple protein or a single module but constitute targeting pathway profiles, by pathway crosstalk, upstream and downstream and vertically converges to integral regulation.

## Conclusions

Our integrative approach is a step toward elucidating the "shotgun-like" pharmacological mechanism of multi-target and multi-ingredient "Fangji" by inter-modular analysis in complex diseases like stroke. Our methodology identified a subset of modules that can serve as potential targets of response mechanism to drug activity. Our findings offer a glimpse of the “tuning” from target entities to the relationship between these entities.

The targets in our study were based on the open database. It seems to be likely to produce some false positives that prevent us from a flawless work. And the validation experiment is still focusing on single targets. Therefore, one of our future works will be designing an integrative strategy using the effect-based multi-omics data from experiments to detect more mechanism with more accuracy and precision. In addition, we will try to design the further validation experiments for the inter-module connectivity.

## Ethics Statement

All of the animal experiments were approved by Ethics Committee of China Academy of Chinese Medicine. The experimental procedures were in accordance with the Prevention of Cruelty to Animals Act 1986 and NIH Guidelines for the Care and Use of Laboratory Animals for Experimental Procedures [National Research Council (US) Institute for Laboratory Animal Research, 1996].

## Author Contributions

PW and FS designed the research. PW performed the research and wrote the paper. LD, MZ, and YW performed the experiment validation. WZ, JM, HH and XX contributed to data and statistical analysis. PW and XX revised the manuscript.

## Funding

This work was supported by grants from the National Natural Science Foundation of China (81873024; 81773923; 81473372; 81373986) and Inheritance Program from Institute of Chinese Materia Media of China Academy of Chinese Medical Sciences (ZXKT18002).

## Conflict of Interest

The authors declare that the research was conducted in the absence of any commercial or financial relationships that could be construed as a potential conflict of interest.

## Abbreviations

HLJDD, Huang-Lian-Jie-Du Decotion; OMIM, Online Mendelian Inheritance in Man; TCMsp, Traditional Chinese Medicine Systems Pharmacology Database and Analysis Platform; CS, coupling score; MCODE, molecular complex detection; KEGG, Kyoto Encyclopedia of Genes and Genomes; stroke module, S-module; HLJDD module, H-module.

## Supplementary Material

Figure S1 Stroke-related protein-protein interaction network and modules; HLJDD targeting protein-protein interaction network and modules. (A) Stroke-related protein-protein interaction network. (B) HLJDD targeting protein-protein interaction network. (C) Modules detected by MCODE in stroke-related protein-protein interaction network, abbreviated as S-modules. (D) Modules detected by MCODE in HLJDD targeting protein-protein interaction network, abbreviated as H-modules. Each vertex represents a protein, circles and triangles are the stroke-related proteins and HLJDD targeting proteins, respectively. The vertexes fulfilled by red represent the overlapping proteins between the stroke-related proteins and HLJDD targeting proteins.

Table S1 The stroke-related proteins and annotation.

Table S2 The ingredients and target of herbs from HLJDD.

Table S3 Target proteins and annotation of HLJDD.

Table S4 The enriched KEGG pathways and their categories of stroke-modules.

Table S5 The enriched KEGG pathways and their categories of HLJDD-modules.
