# Supplementary figures and images for: Intermodule Coupling Analysis of Huang-Lian-Jie-Du Decoction on Stroke
Source: Front Pharmacol. 2019 Nov 5;10:1288. doi: 10.3389/fphar.2019.01288 (PMC6848980; doi:10.3389/fphar.2019.01288)

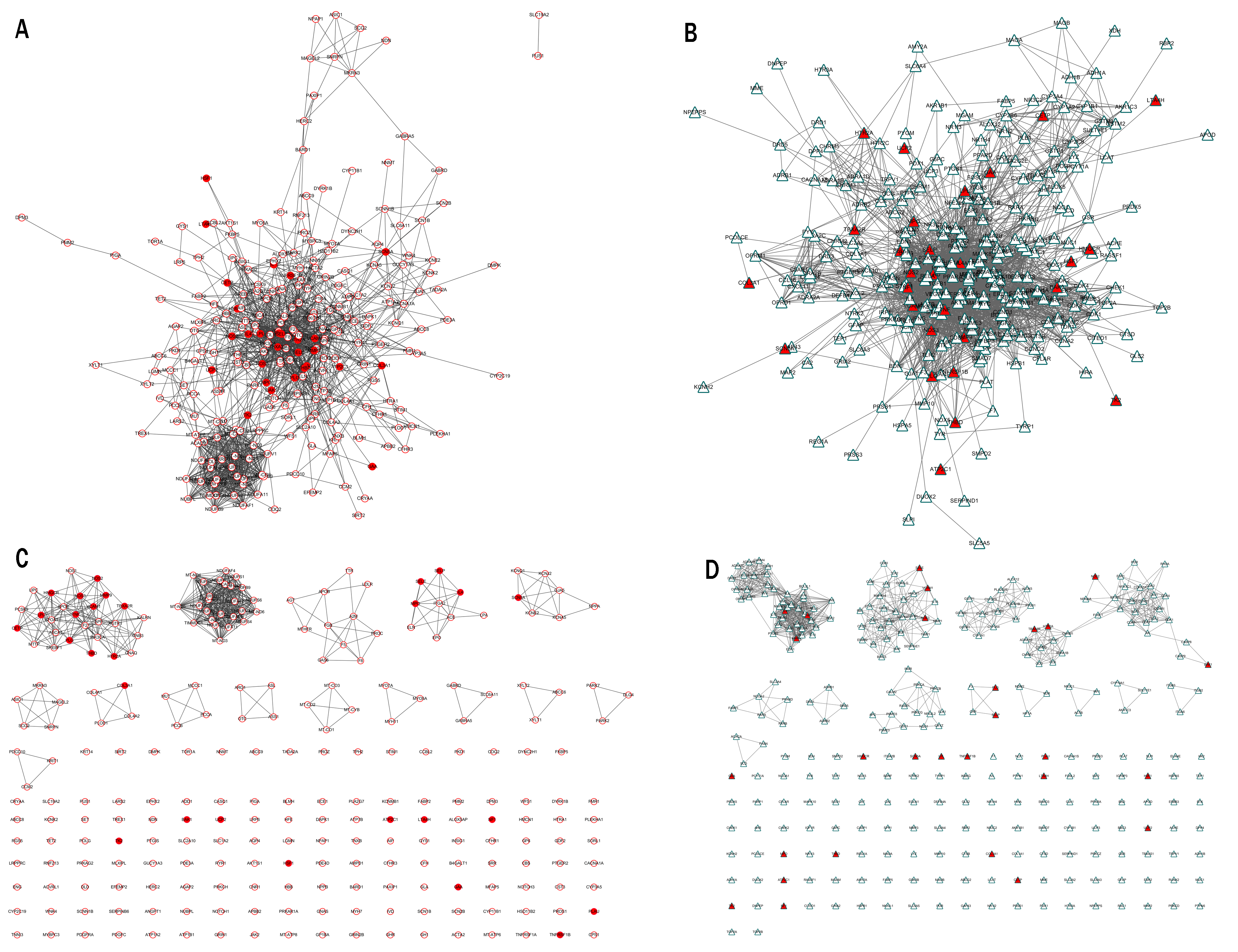

Supplement: Figure S1 — Stroke-related protein-protein interaction network and modules; HLJDD targeting protein-protein interaction network and modules. (A) Stroke-related protein-protein interaction network. (B) HLJDD targeting protein-protein interaction network. (C) Modules detected by MCODE in stroke-related protein-protein interaction network, abbreviated as S-modules. (D) Modules detected by MCODE in HLJDD targeting protein-protein interaction network, abbreviated as H-modules. Each vertex represents a protein, circles and triangles are the stroke-related proteins and HLJDD targeting proteins, respectively. The vertexes fulfilled by red represent the overlapping proteins between the stroke-related proteins and HLJDD targeting proteins. [file Image_1.png]
