# Supplementary material for: Intermodule Coupling Analysis of Huang-Lian-Jie-Du Decoction on Stroke
Source: Front Pharmacol. 2019 Nov 5;10:1288. doi: 10.3389/fphar.2019.01288 (PMC6848980; doi:10.3389/fphar.2019.01288)
Supplement: Table S1 — The stroke-related proteins and annotation. [file Table_1.docx]

Supplementary table 1

| **Official symbol** | **Protein annotation** |
| --- | --- |
| XYLT2 | Xylosyltransferase II; Involved in the formation of heparan sulfate and chondroitin sulfate proteoglycans (PubMed:26027496). Probably catalyzes the first step in biosynthesis of glycosaminoglycan. Transfers D-xylose from UDP-D-xylose to specific serine residues of the core protein. Initial enzyme in the biosynthesis of chondroitin sulfate and dermatan sulfate proteoglycans in fibroblasts and chondrocytes (By similarity). Its enzyme activity has not been demonstrated |
| OTC | Ornithine carbamoyltransferase |
| KCNQ1 | Potassium voltage-gated channel, KQT-like subfamily, member 1; Potassium channel that plays an important role in a number of tissues, including heart, inner ear, stomach and colon (By similarity) (PubMed:10646604). Associates with KCNE beta subunits that modulates current kinetics (By similarity) (PubMed:9312006, PubMed:9108097, PubMed:8900283, PubMed:10646604, PubMed:11101505, PubMed:19687231). Induces a voltage-dependent by rapidly activating and slowly deactivating potassium-selective outward current (By similarity) (PubMed:9312006, PubMed:9108097, PubMed:8900283, PubMed:10646604, P [...] |
| TNFRSF1A | Tumor necrosis factor receptor superfamily, member 1A; Receptor for TNFSF2/TNF-alpha and homotrimeric TNFSF1/lymphotoxin-alpha. The adapter molecule FADD recruits caspase-8 to the activated receptor. The resulting death-inducing signaling complex (DISC) performs caspase-8 proteolytic activation which initiates the subsequent cascade of caspases (aspartate- specific cysteine proteases) mediating apoptosis. Contributes to the induction of non-cytocidal TNF effects including anti-viral state and activation of the acid sphingomyelinase |
| KRT14 | Keratin 14; The nonhelical tail domain is involved in promoting KRT5-KRT14 filaments to self-organize into large bundles and enhances the mechanical properties involved in resilience of keratin intermediate filaments in vitro |
| PLOD1 | Procollagen-lysine, 2-oxoglutarate 5-dioxygenase 1; Forms hydroxylysine residues in -Xaa-Lys-Gly- sequences in collagens. These hydroxylysines serve as sites of attachment for carbohydrate units and are essential for the stability of the intermolecular collagen cross-links |
| CETP | Cholesteryl ester transfer protein, plasma; Involved in the transfer of neutral lipids, including cholesteryl ester and triglyceride, among lipoprotein particles. Allows the net movement of cholesteryl ester from high density lipoproteins/HDL to triglyceride-rich very low density lipoproteins/VLDL, and the equimolar transport of triglyceride from VLDL to HDL (PubMed:3600759, PubMed:24293641). Regulates the reverse cholesterol transport, by which excess cholesterol is removed from peripheral tissues and returned to the liver for elimination (PubMed:17237796) |
| DLD | Dihydrolipoamide dehydrogenase; Lipoamide dehydrogenase is a component of the glycine cleavage system as well as of the alpha-ketoacid dehydrogenase complexes. Involved in the hyperactivation of spermatazoa during capacitation and in the spermatazoal acrosome reaction |
| ABCC6 | ATP-binding cassette, sub-family C (CFTR/MRP), member 6; Isoform 1: May participate directly in the active transport of drugs into subcellular organelles or influence drug distribution indirectly. Transports glutathione conjugates as leukotriene-c4 (LTC4) and N-ethylmaleimide S-glutathione (NEM-GS) |
| GLA | Galactosidase, alpha |
| CYP3A5 | Cytochrome P450, family 3, subfamily A, polypeptide 5; Cytochromes P450 are a group of heme-thiolate monooxygenases. In liver microsomes, this enzyme is involved in an NADPH-dependent electron transport pathway. It oxidizes a variety of structurally unrelated compounds, including steroids, fatty acids, and xenobiotics |
| ACTA2 | Actin, alpha 2, smooth muscle, aorta; Actins are highly conserved proteins that are involved in various types of cell motility and are ubiquitously expressed in all eukaryotic cells |
| MPO | Myeloperoxidase; Part of the host defense system of polymorphonuclear leukocytes. It is responsible for microbicidal activity against a wide range of organisms. In the stimulated PMN, MPO catalyzes the production of hypohalous acids, primarily hypochlorous acid in physiologic situations, and other toxic intermediates that greatly enhance PMN microbicidal activity |
| TADA2A | Transcriptional adaptor 2A; Component of the ATAC complex, a complex with histone acetyltransferase activity on histones H3 and H4. Required for the function of some acidic activation domains, which activate transcription from a distant site (By similarity). Binds double- stranded DNA. Binds dinucleosomes, probably at the linker region between neighboring nucleosomes. Plays a role in chromatin remodeling |
| WFS1 | Wolfram syndrome 1 (wolframin); Participates in the regulation of cellular Ca(2+) homeostasis, at least partly, by modulating the filling state of the endoplasmic reticulum Ca(2+) store |
| ASIC1 | Acid-sensing (proton-gated) ion channel 1 |
| LTA4H | Leukotriene A4 hydrolase; Epoxide hydrolase that catalyzes the final step in the biosynthesis of the proinflammatory mediator leukotriene B4. Has also aminopeptidase activity |
| GNB3 | Guanine nucleotide binding protein (G protein), beta polypeptide 3; Guanine nucleotide-binding proteins (G proteins) are involved as a modulator or transducer in various transmembrane signaling systems. The beta and gamma chains are required for the GTPase activity, for replacement of GDP by GTP, and for G protein- effector interaction |
| GHR | Growth hormone receptor; Receptor for pituitary gland growth hormone involved in regulating postnatal body growth. On ligand binding, couples to the JAK2/STAT5 pathway (By similarity) |
| IL4 | Interleukin 4; Participates in at least several B-cell activation processes as well as of other cell types (PubMed:3016727). It is a costimulator of DNA-synthesis. It induces the expression of class II MHC molecules on resting B-cells. It enhances both secretion and cell surface expression of IgE and IgG1. It also regulates the expression of the low affinity Fc receptor for IgE (CD23) on both lymphocytes and monocytes. Positively regulates IL31RA expression in macrophages (By similarity) |
| APOB | Apolipoprotein B (including Ag(x) antigen); Apolipoprotein B is a major protein constituent of chylomicrons (apo B-48), LDL (apo B-100) and VLDL (apo B-100). Apo B-100 functions as a recognition signal for the cellular binding and internalization of LDL particles by the apoB/E receptor |
| PROC | Protein C (inactivator of coagulation factors Va and VIIIa); Protein C is a vitamin K-dependent serine protease that regulates blood coagulation by inactivating factors Va and VIIIa in the presence of calcium ions and phospholipids (PubMed:25618265). Exerts a protective effect on the endothelial cell barrier function (PubMed:25651845) |
| SLC19A2 | Solute carrier family 19 (thiamine transporter), member 2; High-affinity transporter for the intake of thiamine |
| APOA1 | Apolipoprotein A-I; Participates in the reverse transport of cholesterol from tissues to the liver for excretion by promoting cholesterol efflux from tissues and by acting as a cofactor for the lecithin cholesterol acyltransferase (LCAT). As part of the SPAP complex, activates spermatozoa motility |
| TTR | Transthyretin; Thyroid hormone-binding protein. Probably transports thyroxine from the bloodstream to the brain |
| NDUFB3 | NADH dehydrogenase (ubiquinone) 1 beta subcomplex, 3, 12kDa; Accessory subunit of the mitochondrial membrane respiratory chain NADH dehydrogenase (Complex I), that is believed not to be involved in catalysis. Complex I functions in the transfer of electrons from NADH to the respiratory chain. The immediate electron acceptor for the enzyme is believed to be ubiquinone |
| KALRN | Kalirin, RhoGEF kinase; Promotes the exchange of GDP by GTP. Activates specific Rho GTPase family members, thereby inducing various signaling mechanisms that regulate neuronal shape, growth, and plasticity, through their effects on the actin cytoskeleton. Induces lamellipodia independent of its GEF activity |
| ATP7B | ATPase, Cu++ transporting, beta polypeptide; Involved in the export of copper out of the cells, such as the efflux of hepatic copper into the bile |
| KCNJ2 | Potassium inwardly-rectifying channel, subfamily J, member 2; Probably participates in establishing action potential waveform and excitability of neuronal and muscle tissues. Inward rectifier potassium channels are characterized by a greater tendency to allow potassium to flow into the cell rather than out of it. Their voltage dependence is regulated by the concentration of extracellular potassium; as external potassium is raised, the voltage range of the channel opening shifts to more positive voltages. The inward rectification is mainly due to the blockage of outward current by inter [...] |
| PTGIS | Prostaglandin I2 (prostacyclin) synthase; Catalyzes the isomerization of prostaglandin H2 to prostacyclin (= prostaglandin I2) |
| PTGER2 | Prostaglandin E receptor 2 (subtype EP2), 53kDa; Receptor for prostaglandin E2 (PGE2). The activity of this receptor is mediated by G(s) proteins that stimulate adenylate cyclase. The subsequent raise in intracellular cAMP is responsible for the relaxing effect of this receptor on smooth muscle |
| WNK4 | WNK lysine deficient protein kinase 4; Serine/threonine kinase which plays an important role in the regulation of electrolyte homeostasis, cell signaling, survival and proliferation. Acts as an activator and inhibitor of sodium-coupled chloride cotransporters and potassium-coupled chloride cotransporters respectively. Activates SCNN1A, SCNN1B, SCNN1D, SGK1, TRPV5 and TRPV6. Regulates the activity of the thiazide-sensitive Na-Cl cotransporter, SLC12A3, by phosphorylation which appears to prevent membrane trafficking of SLC12A3. Also inhibits the renal K(+) channel, KCNJ1, via a kinase-i [...] |
| SIRT2 | Sirtuin 2; NAD-dependent protein deacetylase, which deacetylates internal lysines on histone and alpha-tubulin as well as many other proteins such as key transcription factors. Participates in the modulation of multiple and diverse biological processes such as cell cycle control, genomic integrity, microtubule dynamics, cell differentiation, metabolic networks, and autophagy. Plays a major role in the control of cell cycle progression and genomic stability. Functions in the antephase checkpoint preventing precocious mitotic entry in response to microtubule stress agents, and hence allo [...] |
| GDF2 | Growth differentiation factor 2; Potent circulating inhibitor of angiogenesis. Could be involved in bone formation. Signals through the type I activin receptor ACVRL1 but not other Alks. Signaling through SMAD1 in endothelial cells requires TGF-beta coreceptor endoglin/ENG |
| ELN | Elastin; Major structural protein of tissues such as aorta and nuchal ligament, which must expand rapidly and recover completely. Molecular determinant of the late arterial morphogenesis, stabilizing arterial structure by regulating proliferation and organization of vascular smooth muscle (By similarity) |
| KCNA5 | Potassium voltage-gated channel, shaker-related subfamily, member 5; Voltage-gated potassium channel that mediates transmembrane potassium transport in excitable membranes. Forms tetrameric potassium-selective channels through which potassium ions pass in accordance with their electrochemical gradient. The channel alternates between opened and closed conformations in response to the voltage difference across the membrane. Can form functional homotetrameric channels and heterotetrameric channels that contain variable proportions of KCNA1, KCNA2, KCNA4, KCNA5, and possibly other family m [...] |
| APOE | Apolipoprotein E; Mediates the binding, internalization, and catabolism of lipoprotein particles. It can serve as a ligand for the LDL (apo B/E) receptor and for the specific apo-E receptor (chylomicron remnant) of hepatic tissues |
| EPO | Erythropoietin; Erythropoietin is the principal hormone involved in the regulation of erythrocyte differentiation and the maintenance of a physiological level of circulating erythrocyte mass |
| ASS1 | Argininosuccinate synthase 1; Is indirectly involved in the control of blood pressure |
| SLC6A11 | Solute carrier family 6 (neurotransmitter transporter, GABA), member 11; Terminates the action of GABA by its high affinity sodium-dependent reuptake into presynaptic terminals |
| PDGFRA | Platelet-derived growth factor receptor, alpha polypeptide; Tyrosine-protein kinase that acts as a cell-surface receptor for PDGFA, PDGFB and PDGFC and plays an essential role in the regulation of embryonic development, cell proliferation, survival and chemotaxis. Depending on the context, promotes or inhibits cell proliferation and cell migration. Plays an important role in the differentiation of bone marrow-derived mesenchymal stem cells. Required for normal skeleton development and cephalic closure during embryonic development. Required for normal development of the mucosa lining th [...] |
| SORL1 | Sortilin-related receptor, L(DLR class) A repeats containing; Likely to be a multifunctional endocytic receptor, that may be implicated in the uptake of lipoproteins and of proteases. Binds LDL, the major cholesterol-carrying lipoprotein of plasma, and transports it into cells by endocytosis. Binds the receptor- associated protein (RAP). Could play a role in cell-cell interaction. Involved in APP trafficking to and from the Golgi apparatus. It probably acts as a sorting receptor that protects APP from trafficking to late endosome and from processing into amyloid beta, thereby reducing [...] |
| NDUFAF1 | NADH dehydrogenase (ubiquinone) complex I, assembly factor 1; Chaperone protein involved in the assembly of the mitochondrial NADH:ubiquinone oxidoreductase complex (complex I) |
| CCBL2 | Cysteine conjugate-beta lyase 2; Catalyzes the irreversible transamination of the L- tryptophan metabolite L-kynurenine to form kynurenic acid (KA). May catalyze the beta-elimination of S-conjugates and Se- conjugates of L-(seleno)cysteine, resulting in the cleavage of the C-S or C-Se bond (By similarity). Has transaminase activity towards L-kynurenine, tryptophan, phenylalanine, serine, cysteine, methionine, histidine, glutamine and asparagine with glyoxylate as an amino group acceptor (in vitro). Has lower activity with 2- oxoglutarate as amino group acceptor (in vitro) (By similarity) |
| LRPPRC | Leucine-rich pentatricopeptide repeat containing; May play a role in RNA metabolism in both nuclei and mitochondria. In the nucleus binds to HNRPA1-associated poly(A) mRNAs and is part of nmRNP complexes at late stages of mRNA maturation which are possibly associated with nuclear mRNA export. May bind mature mRNA in the nucleus outer membrane. In mitochondria binds to poly(A) mRNA. Plays a role in translation or stability of mitochondrially encoded cytochrome c oxidase (COX) subunits. May be involved in transcription regulation. Cooperates with PPARGC1A to regulate certain mitochondria [...] |
| BARD1 | BRCA1 associated RING domain 1; Probable E3 ubiquitin-protein ligase. The BRCA1-BARD1 heterodimer specifically mediates the formation of 'Lys-6'-linked polyubiquitin chains and coordinates a diverse range of cellular pathways such as DNA damage repair, ubiquitination and transcriptional regulation to maintain genomic stability. Plays a central role in the control of the cell cycle in response to DNA damage. Acts by mediating ubiquitin E3 ligase activity that is required for its tumor suppressor function. Also forms a heterodimer with CSTF1/CSTF-50 to modulate mRNA processing and RNAP I [...] |
| ABCC9 | ATP-binding cassette, sub-family C (CFTR/MRP), member 9; Subunit of ATP-sensitive potassium channels (KATP). Can form cardiac and smooth muscle-type KATP channels with KCNJ11. KCNJ11 forms the channel pore while ABCC9 is required for activation and regulation |
| LRP6 | Low density lipoprotein receptor-related protein 6; Component of the Wnt-Fzd-LRP5-LRP6 complex that triggers beta-catenin signaling through inducing aggregation of receptor- ligand complexes into ribosome-sized signalsomes. Cell-surface coreceptor of Wnt/beta-catenin signaling, which plays a pivotal role in bone formation. The Wnt-induced Fzd/LRP6 coreceptor complex recruits DVL1 polymers to the plasma membrane which, in turn, recruits the AXIN1/GSK3B-complex to the cell surface promoting the formation of signalsomes and inhibiting AXIN1/GSK3- mediated phosphorylation and destruction o [...] |
| XYLT1 | Xylosyltransferase I; Catalyzes the first step in biosynthesis of glycosaminoglycan. Transfers D-xylose from UDP-D-xylose to specific serine residues of the core protein. Initial enzyme in the biosynthesis of chondroitin sulfate and dermatan sulfate proteoglycans in fibroblasts and chondrocytes |
| HERC2 | HECT and RLD domain containing E3 ubiquitin protein ligase 2; E3 ubiquitin-protein ligase that regulates ubiquitin- dependent retention of repair proteins on damaged chromosomes. Recruited to sites of DNA damage in response to ionizing radiation (IR) and facilitates the assembly of UBE2N and RNF8 promoting DNA damage-induced formation of 'Lys-63'-linked ubiquitin chains. Acts as a mediator of binding specificity between UBE2N and RNF8. Involved in the maintenance of RNF168 levels. E3 ubiquitin-protein ligase that promotes the ubiquitination and proteasomal degradation of XPA which infl [...] |
| BLMH | Bleomycin hydrolase; The normal physiological role of BLM hydrolase is unknown, but it catalyzes the inactivation of the antitumor drug BLM (a glycopeptide) by hydrolyzing the carboxamide bond of its B- aminoalaninamide moiety thus protecting normal and malignant cells from BLM toxicity |
| PKD1 | Polycystic kidney disease 1 (autosomal dominant); Involved in renal tubulogenesis (PubMed:12482949). Involved in fluid-flow mechanosensation by the primary cilium in renal epithelium (By similarity). Acts as a regulator of cilium length, together with PKD2 (By similarity). The dynamic control of cilium length is essential in the regulation of mechanotransductive signaling (By similarity). The cilium length response creates a negative feedback loop whereby fluid shear- mediated deflection of the primary cilium, which decreases intracellular cAMP, leads to cilium shortening and thus decr [...] |
| C16orf80 | Chromosome 16 open reading frame 80; Cilium- and flagellum-specific protein that plays a role in axonemal structure organization and motility. Involved in the regulation of the size and morphology of cilia (PubMed:24414207). Required for axonemal microtubules polyglutamylation (PubMed:24414207) |
| NOTCH3 | Notch 3; Functions as a receptor for membrane-bound ligands Jagged1, Jagged2 and Delta1 to regulate cell-fate determination. Upon ligand activation through the released notch intracellular domain (NICD) it forms a transcriptional activator complex with RBPJ/RBPSUH and activates genes of the enhancer of split locus. Affects the implementation of differentiation, proliferation and apoptotic programs (By similarity) |
| FOXRED1 | FAD-dependent oxidoreductase domain containing 1; Required for the assembly of the mitochondrial membrane respiratory chain NADH dehydrogenase (Complex I) (PubMed:20858599, PubMed:25678554). Involved in mid-late stages of complex I assembly (PubMed:25678554) |
| SELP | Selectin P (granule membrane protein 140kDa, antigen CD62); Ca(2+)-dependent receptor for myeloid cells that binds to carbohydrates on neutrophils and monocytes. Mediates the interaction of activated endothelial cells or platelets with leukocytes. The ligand recognized is sialyl-Lewis X. Mediates rapid rolling of leukocyte rolling over vascular surfaces during the initial steps in inflammation through interaction with PSGL1 |
| NDUFS3 | NADH dehydrogenase (ubiquinone) Fe-S protein 3, 30kDa (NADH-coenzyme Q reductase); Core subunit of the mitochondrial membrane respiratory chain NADH dehydrogenase (Complex I) that is believed to belong to the minimal assembly required for catalysis. Complex I functions in the transfer of electrons from NADH to the respiratory chain. The immediate electron acceptor for the enzyme is believed to be ubiquinone (By similarity) |
| PIK3CA | Phosphatidylinositol-4,5-bisphosphate 3-kinase, catalytic subunit alpha; Phosphoinositide-3-kinase (PI3K) that phosphorylates PtdIns (Phosphatidylinositol), PtdIns4P (Phosphatidylinositol 4- phosphate) and PtdIns(4,5)P2 (Phosphatidylinositol 4,5- bisphosphate) to generate phosphatidylinositol 3,4,5-trisphosphate (PIP3). PIP3 plays a key role by recruiting PH domain-containing proteins to the membrane, including AKT1 and PDPK1, activating signaling cascades involved in cell growth, survival, proliferation, motility and morphology. Participates in cellular signaling in response to variou [...] |
| ADD1 | Adducin 1 (alpha); Membrane-cytoskeleton-associated protein that promotes the assembly of the spectrin-actin network. Binds to calmodulin |
| MTTP | Microsomal triglyceride transfer protein; Catalyzes the transport of triglyceride, cholesteryl ester, and phospholipid between phospholipid surfaces (PubMed:23475612, PubMed:8939939, PubMed:26224785, PubMed:25108285, PubMed:22236406). Required for the secretion of plasma lipoproteins that contain apolipoprotein B (PubMed:23475612, PubMed:8939939, PubMed:26224785) |
| LARS2 | leucyl-tRNA synthetase 2, mitochondrial |
| MCCC1 | methylcrotonoyl-CoA carboxylase 1 (alpha); Biotin-attachment subunit of the 3-methylcrotonyl-CoA carboxylase, an enzyme that catalyzes the conversion of 3- methylcrotonyl-CoA to 3-methylglutaconyl-CoA, a critical step for leucine and isovaleric acid catabolism |
| TTLL1 | Tubulin tyrosine ligase-like family, member 1; Catalytic subunit of the neuronal tubulin polyglutamylase complex. Modifies alpha- and beta-tubulin, generating side chains of glutamate on the gamma-carboxyl groups of specific glutamate residues within the C-terminal tail of alpha- and beta-tubulin (By similarity) |
| POLG | Polymerase (DNA directed), gamma; Involved in the replication of mitochondrial DNA. Associates with mitochondrial DNA |
| PMM2 | Phosphomannomutase 2; Involved in the synthesis of the GDP-mannose and dolichol-phosphate-mannose required for a number of critical mannosyl transfer reactions |
| GJA5 | Gap junction protein, alpha 5, 40kDa; One gap junction consists of a cluster of closely packed pairs of transmembrane channels, the connexons, through which materials of low MW diffuse from one cell to a neighboring cell |
| HMCN1 | Hemicentin 1 |
| AGTR1 | Angiotensin II receptor, type 1; Receptor for angiotensin II. Mediates its action by association with G proteins that activate a phosphatidylinositol- calcium second messenger system |
| FABP2 | Fatty acid binding protein 2, intestinal; FABP are thought to play a role in the intracellular transport of long-chain fatty acids and their acyl-CoA esters. FABP2 is probably involved in triglyceride-rich lipoprotein synthesis. Binds saturated long-chain fatty acids with a high affinity, but binds with a lower affinity to unsaturated long- chain fatty acids. FABP2 may also help maintain energy homeostasis by functioning as a lipid sensor |
| NDUFS6 | NADH dehydrogenase (ubiquinone) Fe-S protein 6, 13kDa (NADH-coenzyme Q reductase); Accessory subunit of the mitochondrial membrane respiratory chain NADH dehydrogenase (Complex I), that is believed not to be involved in catalysis. Complex I functions in the transfer of electrons from NADH to the respiratory chain. The immediate electron acceptor for the enzyme is believed to be ubiquinone |
| KCNMB1 | Potassium large conductance calcium-activated channel, subfamily M, beta member 1; Regulatory subunit of the calcium activated potassium KCNMA1 (maxiK) channel. Modulates the calcium sensitivity and gating kinetics of KCNMA1, thereby contributing to KCNMA1 channel diversity. Increases the apparent Ca(2+)/voltage sensitivity of the KCNMA1 channel. It also modifies KCNMA1 channel kinetics and alters its pharmacological properties. It slows down the activation and the deactivation kinetics of the channel. Acts as a negative regulator of smooth muscle contraction by enhancing the calcium s [...] |
| PLA2G7 | Phospholipase A2, group VII (platelet-activating factor acetylhydrolase, plasma); Modulates the action of platelet-activating factor (PAF) by hydrolyzing the sn-2 ester bond to yield the biologically inactive lyso-PAF. Has a specificity for substrates with a short residue at the sn-2 position. It is inactive against long-chain phospholipids |
| MUT | Methylmalonyl CoA mutase; Involved in the degradation of several amino acids, odd- chain fatty acids and cholesterol via propionyl-CoA to the tricarboxylic acid cycle. MCM has different functions in other species |
| NDUFB9 | NADH dehydrogenase (ubiquinone) 1 beta subcomplex, 9, 22kDa; Accessory subunit of the mitochondrial membrane respiratory chain NADH dehydrogenase (Complex I), that is believed to be not involved in catalysis. Complex I functions in the transfer of electrons from NADH to the respiratory chain. The immediate electron acceptor for the enzyme is believed to be ubiquinone |
| NOTCH1 | Notch 1; Functions as a receptor for membrane-bound ligands Jagged1, Jagged2 and Delta1 to regulate cell-fate determination. Upon ligand activation through the released notch intracellular domain (NICD) it forms a transcriptional activator complex with RBPJ/RBPSUH and activates genes of the enhancer of split locus. Affects the implementation of differentiation, proliferation and apoptotic programs. Involved in angiogenesis; negatively regulates endothelial cell proliferation and migration and angiogenic sprouting. Involved in the maturation of both CD4+ and CD8+ cells in the thymus. Im [...] |
| SLC1A2 | Solute carrier family 1 (glial high affinity glutamate transporter), member 2; Transports L-glutamate and also L- and D-aspartate. Essential for terminating the postsynaptic action of glutamate by rapidly removing released glutamate from the synaptic cleft. Acts as a symport by cotransporting sodium |
| SCN2B | Sodium channel, voltage-gated, type II, beta subunit; Crucial in the assembly, expression, and functional modulation of the heterotrimeric complex of the sodium channel. The subunit beta-2 causes an increase in the plasma membrane surface area and in its folding into microvilli. Interacts with TNR may play a crucial role in clustering and regulation of activity of sodium channels at nodes of Ranvier (By similarity) |
| AIP | Aryl hydrocarbon receptor interacting protein; May play a positive role in AHR-mediated (aromatic hydrocarbon receptor) signaling, possibly by influencing its receptivity for ligand and/or its nuclear targeting |
| GRIN2B | Glutamate receptor, ionotropic, N-methyl D-aspartate 2B; NMDA receptor subtype of glutamate-gated ion channels with high calcium permeability and voltage-dependent sensitivity to magnesium. Mediated by glycine. In concert with DAPK1 at extrasynaptic sites, acts as a central mediator for stroke damage. Its phosphorylation at Ser-1303 by DAPK1 enhances synaptic NMDA receptor channel activity inducing injurious Ca2+ influx through them, resulting in an irreversible neuronal death (By similarity) |
| NUBPL | Nucleotide binding protein-like; Required for the assembly of the mitochondrial membrane respiratory chain NADH dehydrogenase (Complex I). May deliver of one or more Fe-S clusters to complex I subunits |
| APP | Amyloid beta (A4) precursor protein; N-APP binds TNFRSF21 triggering caspase activation and degeneration of both neuronal cell bodies (via caspase-3) and axons (via caspase-6) |
| GNAQ | Guanine nucleotide binding protein (G protein), q polypeptide; Guanine nucleotide-binding proteins (G proteins) are involved as modulators or transducers in various transmembrane signaling systems. Regulates B-cell selection and survival and is required to prevent B-cell-dependent autoimmunity. Regulates chemotaxis of BM-derived neutrophils and dendritic cells (in vitro) (By similarity) |
| PRKAG2 | Protein kinase, AMP-activated, gamma 2 non-catalytic subunit; AMP/ATP-binding subunit of AMP-activated protein kinase (AMPK), an energy sensor protein kinase that plays a key role in regulating cellular energy metabolism. In response to reduction of intracellular ATP levels, AMPK activates energy-producing pathways and inhibits energy-consuming processes: inhibits protein, carbohydrate and lipid biosynthesis, as well as cell growth and proliferation. AMPK acts via direct phosphorylation of metabolic enzymes, and by longer-term effects via phosphorylation of transcription regulators. Al [...] |
| HMGCR | 3-hydroxy-3-methylglutaryl-CoA reductase; Transmembrane glycoprotein that is the rate-limiting enzyme in cholesterol biosynthesis as well as in the biosynthesis of nonsterol isoprenoids that are essential for normal cell function including ubiquinone and geranylgeranyl proteins |
| KCNE2 | Potassium voltage-gated channel, Isk-related family, member 2; Ancillary protein that assembles as a beta subunit with a voltage-gated potassium channel complex of pore-forming alpha subunits. Modulates the gating kinetics and enhances stability of the channel complex. Assembled with KCNB1 modulates the gating characteristics of the delayed rectifier voltage-dependent potassium channel KCNB1. Associated with KCNH2/HERG is proposed to form the rapidly activating component of the delayed rectifying potassium current in heart (IKr). May associate with KCNQ2 and/or KCNQ3 and modulate the n [...] |
| ACE | Angiotensin I converting enzyme (peptidyl-dipeptidase A) 1; Converts angiotensin I to angiotensin II by release of the terminal His-Leu, this results in an increase of the vasoconstrictor activity of angiotensin. Also able to inactivate bradykinin, a potent vasodilator. Has also a glycosidase activity which releases GPI-anchored proteins from the membrane by cleaving the mannose linkage in the GPI moiety |
| CRYAA | Crystallin, alpha A; Contributes to the transparency and refractive index of the lens. Has chaperone-like activity, preventing aggregation of various proteins under a wide range of stress conditions |
| CYP11B1 | Cytochrome P450, family 11, subfamily B, polypeptide 1; Has steroid 11-beta-hydroxylase activity. In addition to this activity, the 18 or 19-hydroxylation of steroids and the aromatization of androstendione to estrone have also been ascribed to cytochrome P450 XIB |
| DLG4 | Discs, large homolog 4 (Drosophila); Interacts with the cytoplasmic tail of NMDA receptor subunits and shaker-type potassium channels. Required for synaptic plasticity associated with NMDA receptor signaling. Overexpression or depletion of DLG4 changes the ratio of excitatory to inhibitory synapses in hippocampal neurons. May reduce the amplitude of ASIC3 acid-evoked currents by retaining the channel intracellularly. May regulate the intracellular trafficking of ADR1B (By similarity) |
| VCAM1 | Vascular cell adhesion molecule 1; Important in cell-cell recognition. Appears to function in leukocyte-endothelial cell adhesion. Interacts with integrin alpha-4/beta-1 (ITGA4/ITGB1) on leukocytes, and mediates both adhesion and signal transduction. The VCAM1/ITGA4/ITGB1 interaction may play a pathophysiologic role both in immune responses and in leukocyte emigration to sites of inflammation |
| GUCY1A3 | Guanylate cyclase 1, soluble, alpha 3 |
| ITGA2 | Integrin, alpha 2 (CD49B, alpha 2 subunit of VLA-2 receptor); Integrin alpha-2/beta-1 is a receptor for laminin, collagen, collagen C-propeptides, fibronectin and E-cadherin. It recognizes the proline-hydroxylated sequence G-F-P-G-E-R in collagen. It is responsible for adhesion of platelets and other cells to collagens, modulation of collagen and collagenase gene expression, force generation and organization of newly synthesized extracellular matrix |
| NDUFAF2 | NADH dehydrogenase (ubiquinone) complex I, assembly factor 2; Acts as a molecular chaperone for mitochondrial complex I assembly |
| NDUFS4 | NADH dehydrogenase (ubiquinone) Fe-S protein 4, 18kDa (NADH-coenzyme Q reductase); Accessory subunit of the mitochondrial membrane respiratory chain NADH dehydrogenase (Complex I), that is believed not to be involved in catalysis. Complex I functions in the transfer of electrons from NADH to the respiratory chain. The immediate electron acceptor for the enzyme is believed to be ubiquinone |
| NOS3 | Nitric oxide synthase 3 (endothelial cell); Produces nitric oxide (NO) which is implicated in vascular smooth muscle relaxation through a cGMP-mediated signal transduction pathway. NO mediates vascular endothelial growth factor (VEGF)-induced angiogenesis in coronary vessels and promotes blood clotting through the activation of platelets |
| LIPC | Lipase, hepatic; Hepatic lipase has the capacity to catalyze hydrolysis of phospholipids, mono-, di-, and triglycerides, and acyl-CoA thioesters. It is an important enzyme in HDL metabolism. Hepatic lipase binds heparin |
| NNMT | Nicotinamide N-methyltransferase; Catalyzes the N-methylation of nicotinamide and other pyridines to form pyridinium ions. This activity is important for biotransformation of many drugs and xenobiotic compounds |
| STIM1 | Stromal interaction molecule 1; Plays a role in mediating store-operated Ca(2+) entry (SOCE), a Ca(2+) influx following depletion of intracellular Ca(2+) stores (PubMed:15866891, PubMed:16005298, PubMed:16208375, PubMed:16537481, PubMed:16733527, PubMed:16766533, PubMed:16807233, PubMed:18854159, PubMed:19249086, PubMed:22464749, PubMed:24069340, PubMed:24351972, PubMed:24591628, PubMed:26322679, PubMed:25326555). Acts as Ca(2+) sensor in the endoplasmic reticulum via its EF-hand domain. Upon Ca(2+) depletion, translocates from the endoplasmic reticulum to the plasma membrane where it [...] |
| PCSK9 | Proprotein convertase subtilisin/kexin type 9; Crucial player in the regulation of plasma cholesterol homeostasis. Binds to low-density lipid receptor family members: low density lipoprotein receptor (LDLR), very low density lipoprotein receptor (VLDLR), apolipoprotein E receptor (LRP1/APOER) and apolipoprotein receptor 2 (LRP8/APOER2), and promotes their degradation in intracellular acidic compartments (PubMed:18039658). Acts via a non-proteolytic mechanism to enhance the degradation of the hepatic LDLR through a clathrin LDLRAP1/ARH-mediated pathway. May prevent the recycling of LDLR [...] |
| SCG2 | Secretogranin II; Secretogranin-2 is a neuroendocrine secretory granule protein, which is the precursor for biologically active peptides |
| COL3A1 | Collagen, type III, alpha 1; Collagen type III occurs in most soft connective tissues along with type I collagen. Involved in regulation of cortical development. Is the major ligand of GPR56 in the developing brain and binding to GPR56 inhibits neuronal migration and activates the RhoA pathway by coupling GPR56 to GNA13 and possibly GNA12 |
| GAA | Glucosidase, alpha; acid; Essential for the degradation of glygogen to glucose in lysosomes |
| FGB | Fibrinogen beta chain; Cleaved by the protease thrombin to yield monomers which, together with fibrinogen alpha (FGA) and fibrinogen gamma (FGG), polymerize to form an insoluble fibrin matrix. Fibrin has a major function in hemostasis as one of the primary components of blood clots. In addition, functions during the early stages of wound repair to stabilize the lesion and guide cell migration during re-epithelialization. Was originally thought to be essential for platelet aggregation, based on in vitro studies using anticoagulated blood. However subsequent studies have shown that it is [...] |
| SNRPN | Small nuclear ribonucleoprotein polypeptide N; May be involved in tissue-specific alternative RNA processing events |
| ASL | Argininosuccinate lyase |
| DDIT4 | DNA-damage-inducible transcript 4; Regulates cell growth, proliferation and survival via inhibition of the activity of the mammalian target of rapamycin complex 1 (mTORC1). Inhibition of mTORC1 is mediated by a pathway that involves DDIT4/REDD1, AKT1, the TSC1-TSC2 complex and the GTPase RHEB. Plays an important role in responses to cellular energy levels and cellular stress, including responses to hypoxia and DNA damage. Regulates p53/TP53-mediated apoptosis in response to DNA damage via its effect on mTORC1 activity. Its role in the response to hypoxia depends on the cell type; it me [...] |
| F2 | Coagulation factor II (thrombin); Thrombin, which cleaves bonds after Arg and Lys, converts fibrinogen to fibrin and activates factors V, VII, VIII, XIII, and, in complex with thrombomodulin, protein C. Functions in blood homeostasis, inflammation and wound healing |
| GP6 | Glycoprotein VI (platelet); Collagen receptor involved in collagen-induced platelet adhesion and activation. Plays a key role in platelet procoagulant activity and subsequent thrombin and fibrin formation. This procoagulant function may contribute to arterial and venous thrombus formation. The signaling pathway involves the FcR gamma- chain, the Src kinases (likely Fyn/Lyn), the adapter protein LAT and leads to the activation of phospholipase C gamma2 |
| PLG | Plasminogen; Plasmin dissolves the fibrin of blood clots and acts as a proteolytic factor in a variety of other processes including embryonic development, tissue remodeling, tumor invasion, and inflammation. In ovulation, weakens the walls of the Graafian follicle. It activates the urokinase-type plasminogen activator, collagenases and several complement zymogens, such as C1 and C5. Cleavage of fibronectin and laminin leads to cell detachment and apoptosis. Also cleaves fibrin, thrombospondin and von Willebrand factor. Its role in tissue remodeling and tumor invasion may be modulated b [...] |
| LPL | Lipoprotein lipase; The primary function of this lipase is the hydrolysis of triglycerides of circulating chylomicrons and very low density lipoproteins (VLDL). Binding to heparin sulfate proteogylcans at the cell surface is vital to the function. The apolipoprotein, APOC2, acts as a coactivator of LPL activity in the presence of lipids on the luminal surface of vascular endothelium (By similarity) |
| EFEMP2 | EGF containing fibulin-like extracellular matrix protein 2 |
| COQ2 | Coenzyme Q2 homolog, prenyltransferase (yeast); Catalyzes the prenylation of para-hydroxybenzoate (PHB) with an all-trans polyprenyl group. Mediates the second step in the final reaction sequence of coenzyme Q (CoQ) biosynthesis, which is the condensation of the polyisoprenoid side chain with PHB, generating the first membrane-bound Q intermediate |
| UCP2 | Uncoupling protein 2 (mitochondrial, proton carrier); UCP are mitochondrial transporter proteins that create proton leaks across the inner mitochondrial membrane, thus uncoupling oxidative phosphorylation from ATP synthesis. As a result, energy is dissipated in the form of heat |
| ACAD9 | acyl-CoA dehydrogenase family, member 9; Required for mitochondrial complex I assembly (PubMed:20816094, PubMed:24158852). Has a dehydrogenase activity on palmitoyl-CoA (C16:0) and stearoyl-CoA (C18:0). It is three times more active on palmitoyl-CoA than on stearoyl-CoA. However, it does not play a primary role in long-chain fatty acid oxidation in vivo (PubMed:20816094, PubMed:24158852). Has little activity on octanoyl-CoA (C8:0), butyryl-CoA (C4:0) or isovaleryl-CoA (5:0) |
| GH1 | Growth hormone 1; Plays an important role in growth control. Its major role in stimulating body growth is to stimulate the liver and other tissues to secrete IGF-1. It stimulates both the differentiation and proliferation of myoblasts. It also stimulates amino acid uptake and protein synthesis in muscle and other tissues |
| DYRK1B | Dual-specificity tyrosine-(Y)-phosphorylation regulated kinase 1B; Dual-specificity kinase which possesses both serine/threonine and tyrosine kinase activities. Enhances the transcriptional activity of TCF1/HNF1A and FOXO1. Inhibits epithelial cell migration. Mediates colon carcinoma cell survival in mitogen-poor environments. Inhibits the SHH and WNT1 pathways, thereby enhancing adipogenesis. In addition, promotes expression of the gluconeogenic enzyme glucose-6-phosphatase (G6PC) |
| BVES | Blood vessel epicardial substance; Cell adhesion molecule involved in the establishment and/or maintenance of cell integrity. Involved in the formation and regulation of the tight junction (TJ) paracellular permeability barrier in epithelial cells (PubMed:16188940). Plays a role in VAMP3-mediated vesicular transport and recycling of different receptor molecules through its interaction with VAMP3. Plays a role in the regulation of cell shape and movement by modulating the Rho-family GTPase activity through its interaction with ARHGEF25/GEFT. Induces primordial adhesive contact and aggre [...] |
| MKRN3 | Makorin ring finger protein 3; E3 ubiquitin ligase catalyzing the covalent attachment of ubiquitin moieties onto substrate proteins |
| CFHR1 | Complement factor H-related 1; Involved in complement regulation. The dimerized forms have avidity for tissue-bound complement fragments and efficiently compete with the physiological complement inhibitor CFH. Can associate with lipoproteins and may play a role in lipid metabolism |
| ARL6IP6 | ADP-ribosylation-like factor 6 interacting protein 6 |
| HSD11B2 | Hydroxysteroid (11-beta) dehydrogenase 2; Catalyzes the conversion of cortisol to the inactive metabolite cortisone. Modulates intracellular glucocorticoid levels, thus protecting the nonselective mineralocorticoid receptor from occupation by glucocorticoids |
| GYS1 | Glycogen synthase 1 (muscle); Transfers the glycosyl residue from UDP-Glc to the non- reducing end of alpha-1,4-glucan |
| PRKRA | Protein kinase, interferon-inducible double stranded RNA dependent activator; Activates EIF2AK2/PKR in the absence of double-stranded RNA (dsRNA), leading to phosphorylation of EIF2S1/EFI2-alpha and inhibition of translation and induction of apoptosis. Required for siRNA production by DICER1 and for subsequent siRNA-mediated post- transcriptional gene silencing. Does not seem to be required for processing of pre-miRNA to miRNA by DICER1. Promotes UBC9-p53/TP53 association and sumoylation and phosphorylation of p53/TP53 at 'Lys-386' at 'Ser-392' respectively and enhances its activity in [...] |
| RGS5 | Regulator of G-protein signaling 5; Inhibits signal transduction by increasing the GTPase activity of G protein alpha subunits thereby driving them into their inactive GDP-bound form. Binds to G(i)-alpha and G(o)-alpha, but not to G(s)-alpha (By similarity) |
| MLXIPL | MLX interacting protein-like; Transcriptional repressor. Binds to the canonical and non-canonical E box sequences 5'-CACGTG-3' (By similarity) |
| LPA | Lipoprotein, Lp(a); Apo(a) is the main constituent of lipoprotein(a) (Lp(a)). It has serine proteinase activity and is able of autoproteolysis. Inhibits tissue-type plasminogen activator 1. Lp(a) may be a ligand for megalin/Gp 330 |
| NDUFV1 | NADH dehydrogenase (ubiquinone) flavoprotein 1, 51kDa; Core subunit of the mitochondrial membrane respiratory chain NADH dehydrogenase (Complex I) that is believed to belong to the minimal assembly required for catalysis. Complex I functions in the transfer of electrons from NADH to the respiratory chain. The immediate electron acceptor for the enzyme is believed to be ubiquinone (By similarity) |
| NDUFAF3 | NADH dehydrogenase (ubiquinone) complex I, assembly factor 3; Essential factor for the assembly of mitochondrial NADH:ubiquinone oxidoreductase complex (complex I) |
| A2M | Alpha-2-macroglobulin; Is able to inhibit all four classes of proteinases by a unique 'trapping' mechanism. This protein has a peptide stretch, called the 'bait region' which contains specific cleavage sites for different proteinases. When a proteinase cleaves the bait region, a conformational change is induced in the protein which traps the proteinase. The entrapped enzyme remains active against low molecular weight substrates (activity against high molecular weight substrates is greatly reduced). Following cleavage in the bait region a thioester bond is hydrolyzed and mediates the co [...] |
| APOLD1 | Apolipoprotein L domain containing 1; May be involved in angiogenesis. May play a role in activity-dependent changes of brain vasculature. May affect blood- brain permeability |
| RNF213 | Ring finger protein 213; E3 ubiquitin-protein ligase involved in angiogenesis (PubMed:21799892, PubMed:26278786, PubMed:26766444, PubMed:26126547). Involved in the non-canonical Wnt signaling pathway in vascular development: acts by mediating ubiquitination and degradation of FLNA and NFATC2 downstream of RSPO3, leading to inhibit the non-canonical Wnt signaling pathway and promoting vessel regression (PubMed:26766444). Also has ATPase activity (PubMed:24658080, PubMed:26126547) |
| NOS2 | Nitric oxide synthase 2, inducible; Produces nitric oxide (NO) which is a messenger molecule with diverse functions throughout the body (PubMed:7531687, PubMed:7544004). In macrophages, NO mediates tumoricidal and bactericidal actions. Also has nitrosylase activity and mediates cysteine S-nitrosylation of cytoplasmic target proteins such PTGS2/COX2 (By similarity). As component of the iNOS-S100A8/9 transnitrosylase complex involved in the selective inflammatory stimulus-dependent S-nitrosylation of GAPDH on 'Cys-247' implicated in regulation of the GAIT complex activity and probably mu [...] |
| NDUFV2 | NADH dehydrogenase (ubiquinone) flavoprotein 2, 24kDa; Core subunit of the mitochondrial membrane respiratory chain NADH dehydrogenase (Complex I) that is believed to belong to the minimal assembly required for catalysis. Complex I functions in the transfer of electrons from NADH to the respiratory chain. The immediate electron acceptor for the enzyme is believed to be ubiquinone (By similarity) |
| SCN5A | Sodium channel, voltage-gated, type V, alpha subunit; This protein mediates the voltage-dependent sodium ion permeability of excitable membranes. Assuming opened or closed conformations in response to the voltage difference across the membrane, the protein forms a sodium-selective channel through which Na(+) ions may pass in accordance with their electrochemical gradient. It is a tetrodotoxin-resistant Na(+) channel isoform. This channel is responsible for the initial upstroke of the action potential. Channel inactivation is regulated by intracellular calcium levels |
| TRAK1 | Trafficking protein, kinesin binding 1; Involved in the regulation of endosome-to-lysosome trafficking, including endocytic trafficking of EGF-EGFR complexes and GABA-A receptors |
| TPH2 | Tryptophan hydroxylase 2 |
| PRKCH | Protein kinase C, eta; Calcium-independent, phospholipid- and diacylglycerol (DAG)-dependent serine/threonine-protein kinase that is involved in the regulation of cell differentiation in keratinocytes and pre-B cell receptor, mediates regulation of epithelial tight junction integrity and foam cell formation, and is required for glioblastoma proliferation and apoptosis prevention in MCF-7 cells. In keratinocytes, binds and activates the tyrosine kinase FYN, which in turn blocks epidermal growth factor receptor (EGFR) signaling and leads to keratinocyte growth arrest and differentiation. [...] |
| GP1BA | Glycoprotein Ib (platelet), alpha polypeptide; GP-Ib, a surface membrane protein of platelets, participates in the formation of platelet plugs by binding to the A1 domain of vWF, which is already bound to the subendothelium |
| SELE | Selectin E; Cell-surface glycoprotein having a role in immunoadhesion. Mediates in the adhesion of blood neutrophils in cytokine-activated endothelium through interaction with PSGL1/SELPLG. May have a role in capillary morphogenesis |
| GAS6 | Growth arrest-specific 6 |
| NDN | Necdin homolog (mouse); Growth suppressor that facilitates the entry of the cell into cell cycle arrest. Functionally similar to the retinoblastoma protein it binds to and represses the activity of cell-cycle- promoting proteins such as SV40 large T antigen, adenovirus E1A, and the transcription factor E2F. Necdin also interacts with p53 and works in an additive manner to inhibit cell growth. Functions also as transcription factor and binds directly to specific guanosine-rich DNA sequences (By similarity) |
| NPAP1 | Nuclear pore associated protein 1; May be involved in spermatogenesis |
| HBB | Hemoglobin, beta; Involved in oxygen transport from the lung to the various peripheral tissues |
| LGMN | Legumain; Has a strict specificity for hydrolysis of asparaginyl bonds. Can also cleave aspartyl bonds slowly, especially under acidic conditions. Required for normal lysosomal protein degradation in renal proximal tubules. Required for normal degradation of internalized EGFR. Plays a role in the regulation of cell proliferation via its role in EGFR degradation (By similarity). May be involved in the processing of proteins for MHC class II antigen presentation in the lysosomal/endosomal system |
| GABRA5 | Gamma-aminobutyric acid (GABA) A receptor, alpha 5; GABA, the major inhibitory neurotransmitter in the vertebrate brain, mediates neuronal inhibition by binding to the GABA/benzodiazepine receptor and opening an integral chloride channel |
| NOS1 | Nitric oxide synthase 1 (neuronal); Produces nitric oxide (NO) which is a messenger molecule with diverse functions throughout the body. In the brain and peripheral nervous system, NO displays many properties of a neurotransmitter. Probably has nitrosylase activity and mediates cysteine S-nitrosylation of cytoplasmic target proteins such SRR |
| FKBP5 | FK506 binding protein 5; Immunophilin protein with PPIase and co-chaperone activities. Component of unligated steroid receptors heterocomplexes through interaction with heat-shock protein 90 (HSP90). Plays a role in the intracellular trafficking of heterooligomeric forms of steroid hormone receptors maintaining the complex into the cytoplasm when unliganded |
| SERPINB6 | Serpin peptidase inhibitor, clade B (ovalbumin), member 6; May be involved in the regulation of serine proteinases present in the brain or extravasated from the blood (By similarity). Inhibitor of cathepsin G, kallikrein-8 and thrombin. May play an important role in the inner ear in the protection against leakage of lysosomal content during stress and loss of this protection results in cell death and sensorineural hearing loss |
| SRR | Serine racemase; Catalyzes the synthesis of D-serine from L-serine. D- serine is a key coagonist with glutamate at NMDA receptors. Has dehydratase activity towards both L-serine and D-serine |
| PARK7 | Parkinson protein 7; Protein deglycase that repairs methylglyoxal- and glyoxal-glycated amino acids and proteins, and releases repaired proteins and lactate or glycolate, respectively. Deglycates cysteines, arginines and lysines residues in proteins, and thus reactivates these proteins by reversing glycation by glyoxals. Acts on early glycation intermediates (hemithioacetals and aminocarbinols), preventing the formation of advanced glycation endproducts (AGE) (PubMed:25416785). Plays an important role in cell protection against oxidative stress and cell death acting as oxidative stress [...] |
| AKT1S1 | AKT1 substrate 1 (proline-rich); Subunit of mTORC1, which regulates cell growth and survival in response to nutrient and hormonal signals. mTORC1 is activated in response to growth factors or amino acids. Growth factor-stimulated mTORC1 activation involves a AKT1-mediated phosphorylation of TSC1-TSC2, which leads to the activation of the RHEB GTPase that potently activates the protein kinase activity of mTORC1. Amino acid-signaling to mTORC1 requires its relocalization to the lysosomes mediated by the Ragulator complex and the Rag GTPases. Activated mTORC1 up-regulates protein synthesi [...] |
| TNNI3 | Troponin I type 3 (cardiac); Troponin I is the inhibitory subunit of troponin, the thin filament regulatory complex which confers calcium-sensitivity to striated muscle actomyosin ATPase activity |
| CBS | Cystathionine-beta-synthase; Hydro-lyase catalyzing the first step of the transsulfuration pathway, where the hydroxyl group of L-serine is displaced by L-homocysteine in a beta-replacement reaction to form L-cystathionine, the precursor of L-cysteine. This catabolic route allows the elimination of L-methionine and the toxic metabolite L- homocysteine. Also involved in the production of hydrogen sulfide, a gasotransmitter with signaling and cytoprotective effects on neurons |
| KRIT1 | KRIT1, ankyrin repeat containing; Component of the CCM signaling pathway which is a crucial regulator of heart and vessel formation and integrity (By similarity). Negative regulator of angiogenesis. Inhibits endothelial proliferation, apoptosis, migration, lumen formation and sprouting angiogenesis in primary endothelial cells. Promotes AKT phosphorylation in a NOTCH-dependent and independent manner, and inhibits ERK1/2 phosphorylation indirectly through activation of the DELTA-NOTCH cascade. Acts in concert with CDH5 to establish and maintain correct endothelial cell polarity and vasc [...] |
| INSIG1 | Insulin induced gene 1; Mediates feedback control of cholesterol synthesis by controlling SCAP and HMGCR. Functions by blocking the processing of sterol regulatory element-binding proteins (SREBPs). Capable of retaining the SCAP-SREBF2 complex in the ER thus preventing it from escorting SREBPs to the Golgi. Initiates the sterol-mediated ubiquitin-mediated endoplasmic reticulum-associated degradation (ERAD) of HMGCR via recruitment of the reductase to the ubiquitin ligase, AMFR/gp78. May play a role in growth and differentiation of tissues involved in metabolic control. May play a regul [...] |
| PDE4D | Phosphodiesterase 4D, cAMP-specific; Hydrolyzes the second messenger cAMP, which is a key regulator of many important physiological processes |
| TOR1A | Torsin family 1, member A (torsin A); Protein with chaperone functions important for the control of protein folding, processing, stability and localization as well as for the reduction of misfolded protein aggregates. Involved in the regulation of synaptic vesicle recycling, controls STON2 protein stability in collaboration with the COP9 signalosome complex (CSN). In the nucleus, may link the cytoskeleton with the nuclear envelope, this mechanism seems to be crucial for the control of nuclear polarity, cell movement and, specifically in neurons, nuclear envelope integrity. Participates [...] |
| SCNN1B | Sodium channel, non-voltage-gated 1, beta subunit; Sodium permeable non-voltage-sensitive ion channel inhibited by the diuretic amiloride. Mediates the electrodiffusion of the luminal sodium (and water, which follows osmotically) through the apical membrane of epithelial cells. Plays an essential role in electrolyte and blood pressure homeostasis, but also in airway surface liquid homeostasis, which is important for proper clearance of mucus. Controls the reabsorption of sodium in kidney, colon, lung and sweat glands. Also plays a role in taste perception |
| DMPK | Dystrophia myotonica-protein kinase; Non-receptor serine/threonine protein kinase which is necessary for the maintenance of skeletal muscle structure and function. May play a role in myocyte differentiation and survival by regulating the integrity of the nuclear envelope and the expression of muscle-specific genes. May also phosphorylate PPP1R12A and inhibit the myosin phosphatase activity to regulate myosin phosphorylation. Also critical to the modulation of cardiac contractility and to the maintenance of proper cardiac conduction activity probably through the regulation of cellular c [...] |
| MYH7 | Myosin, heavy chain 7, cardiac muscle, beta; Muscle contraction |
| SREBF1 | Sterol regulatory element binding transcription factor 1; Transcriptional activator required for lipid homeostasis. Regulates transcription of the LDL receptor gene as well as the fatty acid and to a lesser degree the cholesterol synthesis pathway (By similarity). Binds to the sterol regulatory element 1 (SRE-1) (5'-ATCACCCCAC-3'). Has dual sequence specificity binding to both an E-box motif (5'-ATCACGTGA-3') and to SRE-1 (5'-ATCACCCCAC-3') |
| DAPK1 | Death-associated protein kinase 1; Calcium/calmodulin-dependent serine/threonine kinase involved in multiple cellular signaling pathways that trigger cell survival, apoptosis, and autophagy. Regulates both type I apoptotic and type II autophagic cell deaths signal, depending on the cellular setting. The former is caspase-dependent, while the latter is caspase-independent and is characterized by the accumulation of autophagic vesicles. Phosphorylates PIN1 resulting in inhibition of its catalytic activity, nuclear localization, and cellular function. Phosphorylates TPM1, enhancing stress [...] |
| NF1 | Neurofibromin 1; Stimulates the GTPase activity of Ras. NF1 shows greater affinity for Ras GAP, but lower specific activity. May be a regulator of Ras activity |
| PRKAR1A | Protein kinase, cAMP-dependent, regulatory, type I, alpha; Regulatory subunit of the cAMP-dependent protein kinases involved in cAMP signaling in cells |
| TMEM126B | Transmembrane protein 126B; Chaperone protein involved in the assembly of the mitochondrial NADH:ubiquinone oxidoreductase complex (complex I). Participates in constructing the membrane arm of complex I |
| PDE3A | Phosphodiesterase 3A, cGMP-inhibited; Cyclic nucleotide phosphodiesterase with a dual- specificity for the second messengers cAMP and cGMP, which are key regulators of many important physiological processes |
| SLC2A10 | Solute carrier family 2 (facilitated glucose transporter), member 10; Facilitative glucose transporter |
| MFAP5 | Microfibrillar associated protein 5; May play a role in hematopoiesis. In the cardiovascular system, could regulate growth factors or participate in cell signaling in maintaining large vessel integrity (By similarity). Component of the elastin-associated microfibrils (PubMed:8557636) |
| PCNT | Pericentrin; Integral component of the filamentous matrix of the centrosome involved in the initial establishment of organized microtubule arrays in both mitosis and meiosis. Plays a role, together with DISC1, in the microtubule network formation. Is an integral component of the pericentriolar material (PCM). May play an important role in preventing premature centrosome splitting during interphase by inhibiting NEK2 kinase activity at the centrosome |
| RYR1 | Ryanodine receptor 1 (skeletal); Calcium channel that mediates the release of Ca(2+) from the sarcoplasmic reticulum into the cytoplasm and thereby plays a key role in triggering muscle contraction following depolarization of T-tubules. Repeated very high-level exercise increases the open probability of the channel and leads to Ca(2+) leaking into the cytoplasm. Can also mediate the release of Ca(2+) from intracellular stores in neurons, and may thereby promote prolonged Ca(2+) signaling in the brain. Required for normal embryonic development of muscle fibers and skeletal muscle. Requi [...] |
| CACNA1A | Calcium channel, voltage-dependent, P/Q type, alpha 1A subunit; Voltage-sensitive calcium channels (VSCC) mediate the entry of calcium ions into excitable cells and are also involved in a variety of calcium-dependent processes, including muscle contraction, hormone or neurotransmitter release, gene expression, cell motility, cell division and cell death. The isoform alpha-1A gives rise to P and/or Q-type calcium currents. P/Q-type calcium channels belong to the 'high-voltage activated' (HVA) group and are blocked by the funnel toxin (Ftx) and by the omega-agatoxin- IVA (omega-Aga-IVA). [...] |
| F8 | Coagulation factor VIII, procoagulant component; Factor VIII, along with calcium and phospholipid, acts as a cofactor for F9/factor IXa when it converts F10/factor X to the activated form, factor Xa |
| COL4A2 | Collagen, type IV, alpha 2; Type IV collagen is the major structural component of glomerular basement membranes (GBM), forming a 'chicken-wire' meshwork together with laminins, proteoglycans and entactin/nidogen |
| BAK1 | BCL2-antagonist/killer 1; In the presence of an appropriate stimulus, accelerates programmed cell death by binding to, and antagonizing the anti- apoptotic action of BCL2 or its adenovirus homolog E1B 19k protein. Low micromolar levels of zinc ions inhibit the promotion of apoptosis |
| ATP1A2 | ATPase, Na+/K+ transporting, alpha 2 polypeptide; This is the catalytic component of the active enzyme, which catalyzes the hydrolysis of ATP coupled with the exchange of sodium and potassium ions across the plasma membrane. This action creates the electrochemical gradient of sodium and potassium, providing the energy for active transport of various nutrients |
| MT-CO1 | Mitochondrially encoded cytochrome c oxidase I; Cytochrome c oxidase is the component of the respiratory chain that catalyzes the reduction of oxygen to water. Subunits 1- 3 form the functional core of the enzyme complex. CO I is the catalytic subunit of the enzyme. Electrons originating in cytochrome c are transferred via the copper A center of subunit 2 and heme A of subunit 1 to the bimetallic center formed by heme A3 and copper B |
| MT-CYB | Mitochondrially encoded cytochrome b; Component of the ubiquinol-cytochrome c reductase complex (complex III or cytochrome b-c1 complex), which is a respiratory chain that generates an electrochemical potential coupled to ATP synthesis |
| MT-ATP6 | Mitochondrially encoded ATP synthase 6; Mitochondrial membrane ATP synthase (F(1)F(0) ATP synthase or Complex V) produces ATP from ADP in the presence of a proton gradient across the membrane which is generated by electron transport complexes of the respiratory chain. F-type ATPases consist of two structural domains, F(1) - containing the extramembraneous catalytic core and F(0) - containing the membrane proton channel, linked together by a central stalk and a peripheral stalk. During catalysis, ATP synthesis in the catalytic domain of F(1) is coupled via a rotary mechanism of the cent [...] |
| MT-ND6 | Mitochondrially encoded NADH dehydrogenase 6; Core subunit of the mitochondrial membrane respiratory chain NADH dehydrogenase (Complex I) that is believed to belong to the minimal assembly required for catalysis. Complex I functions in the transfer of electrons from NADH to the respiratory chain. The immediate electron acceptor for the enzyme is believed to be ubiquinone (By similarity) |
| MT-ND1 | Mitochondrially encoded NADH dehydrogenase 1; Core subunit of the mitochondrial membrane respiratory chain NADH dehydrogenase (Complex I) that is believed to belong to the minimal assembly required for catalysis. Complex I functions in the transfer of electrons from NADH to the respiratory chain. The immediate electron acceptor for the enzyme is believed to be ubiquinone (By similarity) |
| MT-ND5 | Mitochondrially encoded NADH dehydrogenase 5; Core subunit of the mitochondrial membrane respiratory chain NADH dehydrogenase (Complex I) that is believed to belong to the minimal assembly required for catalysis. Complex I functions in the transfer of electrons from NADH to the respiratory chain. The immediate electron acceptor for the enzyme is believed to be ubiquinone (By similarity) |
| MT-CO2 | Mitochondrially encoded cytochrome c oxidase II; Cytochrome c oxidase is the component of the respiratory chain that catalyzes the reduction of oxygen to water. Subunits 1- 3 form the functional core of the enzyme complex. Subunit 2 transfers the electrons from cytochrome c via its binuclear copper A center to the bimetallic center of the catalytic subunit 1 |
| MT-ND4 | Mitochondrially encoded NADH dehydrogenase 4; Core subunit of the mitochondrial membrane respiratory chain NADH dehydrogenase (Complex I) that is believed to belong to the minimal assembly required for catalysis. Complex I functions in the transfer of electrons from NADH to the respiratory chain. The immediate electron acceptor for the enzyme is believed to be ubiquinone (By similarity) |
| MT-CO3 | Mitochondrially encoded cytochrome c oxidase III; Subunits I, II and III form the functional core of the enzyme complex |
| MT-ND3 | Mitochondrially encoded NADH dehydrogenase 3; Core subunit of the mitochondrial membrane respiratory chain NADH dehydrogenase (Complex I) that is believed to belong to the minimal assembly required for catalysis. Complex I functions in the transfer of electrons from NADH to the respiratory chain. The immediate electron acceptor for the enzyme is believed to be ubiquinone (By similarity) |
| MT-ATP8 | Mitochondrially encoded ATP synthase 8; Mitochondrial membrane ATP synthase (F(1)F(0) ATP synthase or Complex V) produces ATP from ADP in the presence of a proton gradient across the membrane which is generated by electron transport complexes of the respiratory chain. F-type ATPases consist of two structural domains, F(1) - containing the extramembraneous catalytic core and F(0) - containing the membrane proton channel, linked together by a central stalk and a peripheral stalk. During catalysis, ATP synthesis in the catalytic domain of F(1) is coupled via a rotary mechanism of the cent [...] |
| AGT | Angiotensinogen (serpin peptidase inhibitor, clade A, member 8); Essential component of the renin-angiotensin system (RAS), a potent regulator of blood pressure, body fluid and electrolyte homeostasis |
| PARK2 | Parkinson protein 2, E3 ubiquitin protein ligase (parkin); Functions within a multiprotein E3 ubiquitin ligase complex, catalyzing the covalent attachment of ubiquitin moieties onto substrate proteins, such as BCL2, SYT11, CCNE1, GPR37, RHOT1/MIRO1, MFN1, MFN2, STUB1, SNCAIP, SEPT5, TOMM20, USP30, ZNF746 and AIMP2 (PubMed:10973942, PubMed:10888878, PubMed:11431533, PubMed:12150907, PubMed:12628165, PubMed:16135753, PubMed:21376232, PubMed:23754282, PubMed:23620051, PubMed:24660806, PubMed:24751536). Mediates monoubiquitination as well as 'Lys-6', 'Lys-11', 'Lys-48'-linked and 'Lys-63'- [...] |
| CFHR3 | Complement factor H-related 3; Might be involved in complement regulation |
| CFH | Complement factor H; Factor H functions as a cofactor in the inactivation of C3b by factor I and also increases the rate of dissociation of the C3bBb complex (C3 convertase) and the (C3b)NBB complex (C5 convertase) in the alternative complement pathway |
| F5 | Coagulation factor V (proaccelerin, labile factor); Central regulator of hemostasis. It serves as a critical cofactor for the prothrombinase activity of factor Xa that results in the activation of prothrombin to thrombin |
| ATP1B1 | ATPase, Na+/K+ transporting, beta 1 polypeptide; This is the non-catalytic component of the active enzyme, which catalyzes the hydrolysis of ATP coupled with the exchange of Na(+) and K(+) ions across the plasma membrane. The beta subunit regulates, through assembly of alpha/beta heterodimers, the number of sodium pumps transported to the plasma membrane |
| NDUFS2 | NADH dehydrogenase (ubiquinone) Fe-S protein 2, 49kDa (NADH-coenzyme Q reductase); Core subunit of the mitochondrial membrane respiratory chain NADH dehydrogenase (Complex I) that is believed to belong to the minimal assembly required for catalysis. Complex I functions in the transfer of electrons from NADH to the respiratory chain. The immediate electron acceptor for the enzyme is believed to be ubiquinone (By similarity) |
| CASQ1 | Calsequestrin 1 (fast-twitch, skeletal muscle); Calsequestrin is a high-capacity, moderate affinity, calcium-binding protein and thus acts as an internal calcium store in muscle. Calcium ions are bound by clusters of acidic residues at the protein surface, often at the interface between subunits. Can bind around 80 Ca(2+) ions. Regulates the release of lumenal Ca(2+) via the calcium release channel RYR1; this plays an important role in triggering muscle contraction |
| ARG1 | Arginase, liver |
| RNF146 | Ring finger protein 146; E3 ubiquitin-protein ligase that specifically binds poly-ADP-ribosylated (PARsylated) proteins and mediates their ubiquitination and subsequent degradation. May regulate many important biological processes, such as cell survival and DNA damage response. Acts as an activator of the Wnt signaling pathway by mediating the ubiquitination of PARsylated AXIN1 and AXIN2, 2 key components of the beta-catenin destruction complex. Acts in cooperation with tankyrase proteins (TNKS and TNKS2), which mediate PARsylation of target proteins AXIN1, AXIN2, BLZF1, CASC3, TNKS an [...] |
| DPM3 | Dolichyl-phosphate mannosyltransferase polypeptide 3; Stabilizer subunit of the dolichol-phosphate mannose (DPM) synthase complex; tethers catalytic subunit DPM1 to the ER |
| HTRA1 | HtrA serine peptidase 1; Serine protease with a variety of targets, including extracellular matrix proteins such as fibronectin. HTRA1-generated fibronectin fragments further induce synovial cells to up-regulate MMP1 and MMP3 production. May also degrade proteoglycans, such as aggrecan, decorin and fibromodulin. Through cleavage of proteoglycans, may release soluble FGF-glycosaminoglycan complexes that promote the range and intensity of FGF signals in the extracellular space. Regulates the availability of insulin-like growth factors (IGFs) by cleaving IGF-binding proteins. Inhibits sig [...] |
| PLEKHA1 | Pleckstrin homology domain containing, family A (phosphoinositide binding specific) member 1; Binds specifically to phosphatidylinositol 3,4- diphosphate (PtdIns3,4P2), but not to other phosphoinositides. May recruit other proteins to the plasma membrane |
| NDUFAF4 | NADH dehydrogenase (ubiquinone) complex I, assembly factor 4; Involved in the assembly of mitochondrial NADH:ubiquinone oxidoreductase complex (complex I). May be involved in cell proliferation and survival of hormone-dependent tumor cells. May be a regulator of breast tumor cell invasion |
| CNR1 | Cannabinoid receptor 1 (brain); Involved in cannabinoid-induced CNS effects. Acts by inhibiting adenylate cyclase. Could be a receptor for anandamide. Inhibits L-type Ca(2+) channel current. Isoform 2 and isoform 3 have altered ligand binding |
| FMR1 | Fragile X mental retardation 1; Translation repressor. Component of the CYFIP1-EIF4E- FMR1 complex which binds to the mRNA cap and mediates translational repression. In the CYFIP1-EIF4E-FMR1 complex this subunit mediates translation repression (By similarity). RNA- binding protein that plays a role in intracellular RNA transport and in the regulation of translation of target mRNAs. Associated with polysomes. May play a role in the transport of mRNA from the nucleus to the cytoplasm. Binds strongly to poly(G), binds moderately to poly(U) but shows very little binding to poly(A) or poly(C) |
| GNAS | GNAS complex locus; Guanine nucleotide-binding proteins (G proteins) function as transducers in numerous signaling pathways controlled by G protein-coupled receptors (GPCRs). Signaling involves the activation of adenylyl cyclases, resulting in increased levels of the signaling molecule cAMP. GNAS functions downstream of several GPCRs, including beta-adrenergic receptors. XLas isoforms interact with the same set of receptors as GNAS isoforms (By similarity) |
| CYP2C19 | Cytochrome P450, family 2, subfamily C, polypeptide 19; Responsible for the metabolism of a number of therapeutic agents such as the anticonvulsant drug S-mephenytoin, omeprazole, proguanil, certain barbiturates, diazepam, propranolol, citalopram and imipramine |
| NDUFA1 | NADH dehydrogenase (ubiquinone) 1 alpha subcomplex, 1, 7.5kDa; Accessory subunit of the mitochondrial membrane respiratory chain NADH dehydrogenase (Complex I), that is believed not to be involved in catalysis. Complex I functions in the transfer of electrons from NADH to the respiratory chain. The immediate electron acceptor for the enzyme is believed to be ubiquinone |
| GRIN1 | Glutamate receptor, ionotropic, N-methyl D-aspartate 1; NMDA receptor subtype of glutamate-gated ion channels with high calcium permeability and voltage-dependent sensitivity to magnesium. Mediated by glycine. This protein plays a key role in synaptic plasticity, synaptogenesis, excitotoxicity, memory acquisition and learning. It mediates neuronal functions in glutamate neurotransmission. Is involved in the cell surface targeting of NMDA receptors (By similarity) |
| MMP9 | Matrix metallopeptidase 9 (gelatinase B, 92kDa gelatinase, 92kDa type IV collagenase); May play an essential role in local proteolysis of the extracellular matrix and in leukocyte migration. Could play a role in bone osteoclastic resorption. Cleaves KiSS1 at a Gly-\|-Leu bond. Cleaves type IV and type V collagen into large C-terminal three quarter fragments and shorter N-terminal one quarter fragments. Degrades fibronectin but not laminin or Pz-peptide |
| SET | SET nuclear oncogene; Multitasking protein, involved in apoptosis, transcription, nucleosome assembly and histone chaperoning. Isoform 2 anti-apoptotic activity is mediated by inhibition of the GZMA-activated DNase, NME1. In the course of cytotoxic T- lymphocyte (CTL)-induced apoptosis, GZMA cleaves SET, disrupting its binding to NME1 and releasing NME1 inhibition. Isoform 1 and isoform 2 are potent inhibitors of protein phosphatase 2A. Isoform 1 and isoform 2 inhibit EP300/CREBBP and PCAF-mediated acetylation of histones (HAT) and nucleosomes, most probably by masking the accessibilit [...] |
| PLAU | Plasminogen activator, urokinase; Specifically cleaves the zymogen plasminogen to form the active enzyme plasmin |
| ENG | Endoglin; Major glycoprotein of vascular endothelium. Involved in the regulation of angiogenesis. May play a critical role in the binding of endothelial cells to integrins and/or other RGD receptors. Acts as TGF-beta coreceptor and is involved in the TGF- beta/BMP signaling cascade. Required for GDF2/BMP9 signaling through SMAD1 in endothelial cells and modulates TGF-beta1 signaling through SMAD3 |
| ABCA1 | ATP-binding cassette, sub-family A (ABC1), member 1; cAMP-dependent and sulfonylurea-sensitive anion transporter. Key gatekeeper influencing intracellular cholesterol transport |
| ECE1 | Endothelin converting enzyme 1; Converts big endothelin-1 to endothelin-1 |
| PROZ | Protein Z, vitamin K-dependent plasma glycoprotein; Appears to assist hemostasis by binding thrombin and promoting its association with phospholipid vesicles. Inhibits activity of the coagulation protease factor Xa in the presence of SERPINA10, calcium and phospholipids |
| COL4A1 | Collagen, type IV, alpha 1; Type IV collagen is the major structural component of glomerular basement membranes (GBM), forming a 'chicken-wire' meshwork together with laminins, proteoglycans and entactin/nidogen |
| TNFRSF1B | Tumor necrosis factor receptor superfamily, member 1B; Receptor with high affinity for TNFSF2/TNF-alpha and approximately 5-fold lower affinity for homotrimeric TNFSF1/lymphotoxin-alpha. The TRAF1/TRAF2 complex recruits the apoptotic suppressors BIRC2 and BIRC3 to TNFRSF1B/TNFR2. This receptor mediates most of the metabolic effects of TNF-alpha. Isoform 2 blocks TNF-alpha-induced apoptosis, which suggests that it regulates TNF-alpha function by antagonizing its biological activity |
| PCCA | Propionyl CoA carboxylase, alpha polypeptide |
| NPPB | Natriuretic peptide B; Cardiac hormone which may function as a paracrine antifibrotic factor in the heart. Also plays a key role in cardiovascular homeostasis through natriuresis, diuresis, vasorelaxation, and inhibition of renin and aldosterone secretion. Specifically binds and stimulates the cGMP production of the NPR1 receptor. Binds the clearance receptor NPR3 |
| NPPA | Natriuretic peptide A; Hormone playing a key role in cardiovascular homeostasis through regulation of natriuresis, diuresis, and vasodilation. Also plays a role in female pregnancy by promoting trophoblast invasion and spiral artery remodeling in uterus. Specifically binds and stimulates the cGMP production of the NPR1 receptor. Binds the clearance receptor NPR3 |
| MTHFR | Methylenetetrahydrofolate reductase (NAD(P)H); Catalyzes the conversion of 5,10- methylenetetrahydrofolate to 5-methyltetrahydrofolate, a co- substrate for homocysteine remethylation to methionine |
| PUS1 | Pseudouridylate synthase 1; Converts specific uridines to PSI in a number of tRNA substrates. Acts on positions 27/28 in the anticodon stem and also positions 34 and 36 in the anticodon of an intron containing tRNA. Involved in regulation of nuclear receptor activity possibly through pseudouridylation of SRA1 RNA (By similarity) |
| CST3 | Cystatin C; As an inhibitor of cysteine proteinases, this protein is thought to serve an important physiological role as a local regulator of this enzyme activity |
| THBD | Thrombomodulin; Thrombomodulin is a specific endothelial cell receptor that forms a 1:1 stoichiometric complex with thrombin. This complex is responsible for the conversion of protein C to the activated protein C (protein Ca). Once evolved, protein Ca scissions the activated cofactors of the coagulation mechanism, factor Va and factor VIIIa, and thereby reduces the amount of thrombin generated |
| NDUFAF5 | NADH dehydrogenase (ubiquinone) complex I, assembly factor 5; Involved in the assembly of mitochondrial NADH:ubiquinone oxidoreductase complex (complex I, MT-ND1) at early stages. May have methyltransferase activity |
| GABRD | Gamma-aminobutyric acid (GABA) A receptor, delta; GABA, the major inhibitory neurotransmitter in the vertebrate brain, mediates neuronal inhibition by binding to the GABA/benzodiazepine receptor and opening an integral chloride channel |
| HTR2A | 5-hydroxytryptamine (serotonin) receptor 2A, G protein-coupled; G-protein coupled receptor for 5-hydroxytryptamine (serotonin). Also functions as a receptor for various drugs and psychoactive substances, including mescaline, psilocybin, 1-(2,5- dimethoxy-4-iodophenyl)-2-aminopropane (DOI) and lysergic acid diethylamide (LSD). Ligand binding causes a conformation change that triggers signaling via guanine nucleotide-binding proteins (G proteins) and modulates the activity of down-stream effectors. Beta-arrestin family members inhibit signaling via G proteins and mediate activation of al [...] |
| B4GALT1 | UDP-Gal:betaGlcNAc beta 1,4- galactosyltransferase, polypeptide 1; The Golgi complex form catalyzes the production of lactose in the lactating mammary gland and could also be responsible for the synthesis of complex-type N-linked oligosaccharides in many glycoproteins as well as the carbohydrate moieties of glycolipids |
| TET2 | Tet methylcytosine dioxygenase 2; Dioxygenase that catalyzes the conversion of the modified genomic base 5-methylcytosine (5mC) into 5- hydroxymethylcytosine (5hmC) and plays a key role in active DNA demethylation. Has a preference for 5-hydroxymethylcytosine in CpG motifs. Also mediates subsequent conversion of 5hmC into 5- formylcytosine (5fC), and conversion of 5fC to 5-carboxylcytosine (5caC). Conversion of 5mC into 5hmC, 5fC and 5caC probably constitutes the first step in cytosine demethylation. Methylation at the C5 position of cytosine bases is an epigenetic modification of the [...] |
| PIGA | Phosphatidylinositol glycan anchor biosynthesis, class A; Necessary for the synthesis of N-acetylglucosaminyl- phosphatidylinositol, the very early intermediate in GPI-anchor biosynthesis |
| ALOX5AP | Arachidonate 5-lipoxygenase-activating protein; Required for leukotriene biosynthesis by ALOX5 (5- lipoxygenase). Anchors ALOX5 to the membrane. Binds arachidonic acid, and could play an essential role in the transfer of arachidonic acid to ALOX5. Binds to MK-886, a compound that blocks the biosynthesis of leukotrienes |
| CCM2 | Cerebral cavernous malformation 2; Component of the CCM signaling pathway which is a crucial regulator of heart and vessel formation and integrity. May act through the stabilization of endothelial cell junctions (By similarity). May function as a scaffold protein for MAP2K3-MAP3K3 signaling. Seems to play a major role in the modulation of MAP3K3- dependent p38 activation induced by hyperosmotic shock (By similarity) |
| JAK2 | Janus kinase 2; Non-receptor tyrosine kinase involved in various processes such as cell growth, development, differentiation or histone modifications. Mediates essential signaling events in both innate and adaptive immunity. In the cytoplasm, plays a pivotal role in signal transduction via its association with type I receptors such as growth hormone (GHR), prolactin (PRLR), leptin (LEPR), erythropoietin (EPOR), thrombopoietin (THPO); or type II receptors including IFN-alpha, IFN-beta, IFN-gamma and multiple interleukins (PubMed:7615558). Following ligand-binding to cell surface recepto [...] |
| AQP4 | Aquaporin 4; Forms a water-specific channel. Osmoreceptor which regulates body water balance and mediates water flow within the central nervous system |
| ACVRL1 | Activin A receptor type II-like 1; Type I receptor for TGF-beta family ligands BMP9/GDF2 and BMP10 and important regulator of normal blood vessel development. On ligand binding, forms a receptor complex consisting of two type II and two type I transmembrane serine/threonine kinases. Type II receptors phosphorylate and activate type I receptors which autophosphorylate, then bind and activate SMAD transcriptional regulators. May bind activin as well |
| ABCC8 | ATP-binding cassette, sub-family C (CFTR/MRP), member 8; Subunit of the beta-cell ATP-sensitive potassium channel (KATP). Regulator of ATP-sensitive K(+) channels and insulin release |
| PDCD10 | Programmed cell death 10; Promotes cell proliferation. Modulates apoptotic pathways. Increases mitogen-activated protein kinase activity and STK26 activity. Important for cell migration, and for normal structure and assembly of the Golgi complex. Important for KDR/VEGFR2 signaling. Increases the stability of KDR/VEGFR2 and prevents its breakdown. Required for normal cardiovascular development. Required for normal angiogenesis, vasculogenesis and hematopoiesis during embryonic development (By similarity) |
| ATP2C1 | ATPase, Ca++ transporting, type 2C, member 1; This magnesium-dependent enzyme catalyzes the hydrolysis of ATP coupled with the transport of the calcium |
| PROS1 | Protein S (alpha); Anticoagulant plasma protein; it is a cofactor to activated protein C in the degradation of coagulation factors Va and VIIIa. It helps to prevent coagulation and stimulating fibrinolysis |
| MYH11 | Myosin, heavy chain 11, smooth muscle; Muscle contraction |
| DYNC2H1 | Dynein, cytoplasmic 2, heavy chain 1; May function as a motor for intraflagellar retrograde transport. Functions in cilia biogenesis. May play a role in transport between endoplasmic reticulum and Golgi or organization of the Golgi in cells (By similarity) |
| MYO5A | Myosin VA (heavy chain 12, myoxin); Processive actin-based motor that can move in large steps approximating the 36-nm pseudo-repeat of the actin filament. Involved in melanosome transport. Also mediates the transport of vesicles to the plasma membrane. May also be required for some polarization process involved in dendrite formation |
| PAXIP1 | PAX interacting (with transcription-activation domain) protein 1; Involved in DNA damage response and in transcriptional regulation through histone methyltransferase (HMT) complexes. Plays a role in early development. In DNA damage response is required for cell survival after ionizing radiation. In vitro shown to be involved in the homologous recombination mechanism for the repair of double-strand breaks (DSBs). Its localization to DNA damage foci requires RNF8 and UBE2N. Recruits TP53BP1 to DNA damage foci and, at least in particular repair processes, effective DNA damage response app [...] |
| MYO7A | Myosin VIIA; Myosins are actin-based motor molecules with ATPase activity. Unconventional myosins serve in intracellular movements. Their highly divergent tails bind to membranous compartments, which are then moved relative to actin filaments. In the retina, plays an important role in the renewal of the outer photoreceptor disks. Plays an important role in the distribution and migration of retinal pigment epithelial (RPE) melanosomes and phagosomes, and in the regulation of opsin transport in retinal photoreceptors. In the inner ear, plays an important role in differentiation, morphoge [...] |
| NDUFA11 | NADH dehydrogenase (ubiquinone) 1 alpha subcomplex, 11, 14.7kDa; Accessory subunit of the mitochondrial membrane respiratory chain NADH dehydrogenase (Complex I), that is believed not to be involved in catalysis. Complex I functions in the transfer of electrons from NADH to the respiratory chain. The immediate electron acceptor for the enzyme is believed to be ubiquinone (By similarity) |
| TREX1 | Three prime repair exonuclease 1; Major cellular 3'-to-5' DNA exonuclease which digests single-stranded DNA (ssDNA) and double-stranded DNA (dsDNA) with mismatched 3' termini. Prevents cell-intrinsic initiation of autoimmunity. Acts by metabolizing DNA fragments from endogenous retroelements, including L1, LTR and SINE elements. Unless degraded, these DNA fragments accumulate in the cytosol and activate the IFN-stimulatory DNA (ISD) response and innate immune signaling. Prevents chronic ATM-dependent checkpoint activation, by processing ssDNA polynucleotide species arising from the pro [...] |
| NDUFS1 | NADH dehydrogenase (ubiquinone) Fe-S protein 1, 75kDa (NADH-coenzyme Q reductase); Core subunit of the mitochondrial membrane respiratory chain NADH dehydrogenase (Complex I) that is believed to belong to the minimal assembly required for catalysis. Complex I functions in the transfer of electrons from NADH to the respiratory chain. The immediate electron acceptor for the enzyme is believed to be ubiquinone (By similarity). This is the largest subunit of complex I and it is a component of the iron-sulfur (IP) fragment of the enzyme. It may form part of the active site crevice where NAD [...] |
| TBXA2R | Thromboxane A2 receptor; Receptor for thromboxane A2 (TXA2), a potent stimulator of platelet aggregation. The activity of this receptor is mediated by a G-protein that activates a phosphatidylinositol-calcium second messenger system. In the kidney, the binding of TXA2 to glomerular TP receptors causes intense vasoconstriction. Activates phospholipase C. Isoform 1 activates adenylyl cyclase. Isoform 2 inhibits adenylyl cyclase |
| KCNK2 | Potassium channel, subfamily K, member 2; Ion channel that contributes to passive transmembrane potassium transport (PubMed:23169818). Reversibly converts between a voltage-insensitive potassium leak channel and a voltage- dependent outward rectifying potassium channel in a phosphorylation-dependent manner (PubMed:11319556). In astrocytes, forms mostly heterodimeric potassium channels with KCNK1, with only a minor proportion of functional channels containing homodimeric KCNK2. In astrocytes, the heterodimer formed by KCNK1 and KCNK2 is required for rapid glutamate release in response t [...] |
| SCN1B | Sodium channel, voltage-gated, type I, beta subunit; Crucial in the assembly, expression, and functional modulation of the heterotrimeric complex of the sodium channel. The subunit beta-1 can modulate multiple alpha subunit isoforms from brain, skeletal muscle, and heart. Its association with neurofascin may target the sodium channels to the nodes of Ranvier of developing axons and retain these channels at the nodes in mature myelinated axons |
| TNF | Tumor necrosis factor; Cytokine that binds to TNFRSF1A/TNFR1 and TNFRSF1B/TNFBR. It is mainly secreted by macrophages and can induce cell death of certain tumor cell lines. It is potent pyrogen causing fever by direct action or by stimulation of interleukin-1 secretion and is implicated in the induction of cachexia, Under certain conditions it can stimulate cell proliferation and induce cell differentiation. Impairs regulatory T-cells (Treg) function in individuals with rheumatoid arthritis via FOXP3 dephosphorylation. Upregulates the expression of protein phosphatase 1 (PP1), which de [...] |
| CPS1 | Carbamoyl-phosphate synthase 1, mitochondrial; Involved in the urea cycle of ureotelic animals where the enzyme plays an important role in removing excess ammonia from the cell |
| TNXB | Tenascin XB; Appears to mediate interactions between cells and the extracellular matrix. Substrate-adhesion molecule that appears to inhibit cell migration. Accelerates collagen fibril formation. May play a role in supporting the growth of epithelial tumors |
| TK2 | Thymidine kinase 2, mitochondrial; Deoxyribonucleoside kinase that phosphorylates thymidine, deoxycytidine, and deoxyuridine. Also phosphorylates anti-viral and anti-cancer nucleoside analogs |
| EBP | Emopamil binding protein (sterol isomerase); Catalyzes the conversion of Delta(8)-sterols to their corresponding Delta(7)-isomers |
| HFE | Hemochromatosis; Binds to transferrin receptor (TFR) and reduces its affinity for iron-loaded transferrin |
| IVD | isovaleryl-CoA dehydrogenase |
| TIMMDC1 | Translocase of inner mitochondrial membrane domain containing 1; Chaperone protein involved in the assembly of the mitochondrial NADH:ubiquinone oxidoreductase complex (complex I). Participates in constructing the membrane arm of complex I |
| PCCB | Propionyl CoA carboxylase, beta polypeptide |
| PDGFC | Platelet derived growth factor C; Growth factor that plays an essential role in the regulation of embryonic development, cell proliferation, cell migration, survival and chemotaxis. Potent mitogen and chemoattractant for cells of mesenchymal origin. Required for normal skeleton formation during embryonic development, especially for normal development of the craniofacial skeleton and for normal development of the palate. Required for normal skin morphogenesis during embryonic development. Plays an important role in wound healing, where it appears to be involved in three stages: inflamma [...] |
| APBB2 | Amyloid beta (A4) precursor protein-binding, family B, member 2; May modulate the internalization of beta-amyloid precursor protein |
| ANGPT1 | Angiopoietin 1; Binds and activates TEK/TIE2 receptor by inducing its dimerization and tyrosine phosphorylation. Plays an important role in the regulation of angiogenesis, endothelial cell survival, proliferation, migration, adhesion and cell spreading, reorganization of the actin cytoskeleton, but also maintenance of vascular quiescence. Required for normal angiogenesis and heart development during embryogenesis. After birth, activates or inhibits angiogenesis, depending on the context. Inhibits angiogenesis and promotes vascular stability in quiescent vessels, where endothelial cells [...] |
| AMPD1 | Adenosine monophosphate deaminase 1; AMP deaminase plays a critical role in energy metabolism |
| EPHX2 | Epoxide hydrolase 2, cytoplasmic; Bifunctional enzyme. The C-terminal domain has epoxide hydrolase activity and acts on epoxides (alkene oxides, oxiranes) and arene oxides. Plays a role in xenobiotic metabolism by degrading potentially toxic epoxides. Also determines steady-state levels of physiological mediators. The N-terminal domain has lipid phosphatase activity, with the highest activity towards threo- 9,10-phosphonooxy-hydroxy-octadecanoic acid, followed by erythro- 9,10-phosphonooxy-hydroxy-octadecanoic acid, 12-phosphonooxy- octadec-9Z-enoic acid, 12-phosphonooxy-octadec-9E-eno [...] |
| HSF1 | Heat shock transcription factor 1; DNA-binding protein that specifically binds heat shock promoter elements (HSE) and activates transcription. In higher eukaryotes, HSF is unable to bind to the HSE unless the cells are heat shocked |
| MAGEL2 | MAGE-like 2; Probably enhances ubiquitin ligase activity of RING-type zinc finger-containing E3 ubiquitin-protein ligases, possibly through recruitment and/or stabilization of the Ubl-conjugating enzyme (E2) at the E3:substrate complex. Acts as a regulator of retrograde transport via its interaction with VPS35. Recruited to retromer-containing endosomes and promotes the formation of 'Lys- 63'-linked polyubiquitin chains at 'Lys-220' of WASH1 together with TRIM27, leading to promote endosomal F-actin assembly (PubMed:23452853). Regulates the circadian clock by repressing the transcripti [...] |
| MYBPC3 | Myosin binding protein C, cardiac; Thick filament-associated protein located in the crossbridge region of vertebrate striated muscle a bands. In vitro it binds MHC, F-actin and native thin filaments, and modifies the activity of actin-activated myosin ATPase. It may modulate muscle contraction or may play a more structural role |
| AGAP2 | ArfGAP with GTPase domain, ankyrin repeat and PH domain 2; GTPase-activating protein (GAP) for ARF1 and ARF5, which also shows strong GTPase activity. Isoform 1 participates in the prevention of neuronal apoptosis by enhancing PI3 kinase activity. It aids the coupling of metabotropic glutamate receptor 1 (GRM1) to cytoplasmic PI3 kinase by interacting with Homer scaffolding proteins, and also seems to mediate anti-apoptotic effects of NGF by activating nuclear PI3 kinase. Isoform 2 does not stimulate PI3 kinase but may protect cells from apoptosis by stimulating Akt. |
| LDLR | Low density lipoprotein receptor; Binds LDL, the major cholesterol-carrying lipoprotein of plasma, and transports it into cells by endocytosis. In order to be internalized, the receptor-ligand complexes must first cluster into clathrin-coated pits |

Supplementary table 1. The stroke-related proteins and annotation.
