# Supplementary material for: Intermodule Coupling Analysis of Huang-Lian-Jie-Du Decoction on Stroke
Source: Front Pharmacol. 2019 Nov 5;10:1288. doi: 10.3389/fphar.2019.01288 (PMC6848980; doi:10.3389/fphar.2019.01288)
Supplement: Table S2 — The ingredients and target of herbs from HLJDD. [file Table_2.docx]

Supplementary table 2

| **Herb** | **Ingredient** | **Target** |
| --- | --- | --- |
| Scutellariae Radix | 1,8-cineole | Nitric-oxide synthase, endothelial |
| Scutellariae Radix | 1,8-cineole | Gamma-aminobutyric-acid receptor alpha-2 subunit |
| Scutellariae Radix | 1,8-cineole | Gamma-aminobutyric acid receptor subunit alpha-1 |
| Scutellariae Radix | 1,8-cineole | Cytochrome P450-cam |
| Scutellariae Radix | 1,8-cineole | Prostaglandin G/H synthase 2 |
| Scutellariae Radix | 1,8-cineole | Gamma-aminobutyric-acid receptor alpha-5 subunit |
| Scutellariae Radix | 1,8-cineole | Alcohol dehydrogenase 1B |
| Scutellariae Radix | 1,8-cineole | Alcohol dehydrogenase 1C |
| Scutellariae Radix | 1,8-cineole | Neuronal acetylcholine receptor subunit alpha-2 |
| Scutellariae Radix | 1,8-cineole | Gamma-aminobutyric-acid receptor alpha-3 subunit |
| Scutellariae Radix | 1,8-cineole | Proline dehydrogenase, mitochondrial |
| Scutellariae Radix | PEL | Alcohol dehydrogenase 1B |
| Scutellariae Radix | PEL | Alcohol dehydrogenase 1C |
| Scutellariae Radix | PEL | Nicotinate-nucleotide--dimethylbenzimidazole phosphoribosyltransferase |
| Scutellariae Radix | PEL | Trypsin-3 |
| Scutellariae Radix | PEL | Alcohol dehydrogenase 1A |
| Scutellariae Radix | PEL | Amine oxidase [flavin-containing] A |
| Scutellariae Radix | acacetin | Nitric oxide synthase, inducible |
| Scutellariae Radix | acacetin | Prostaglandin G/H synthase 1 |
| Scutellariae Radix | acacetin | Androgen receptor |
| Scutellariae Radix | acacetin | Prostaglandin G/H synthase 2 |
| Scutellariae Radix | acacetin | Dipeptidyl peptidase IV |
| Scutellariae Radix | acacetin | Heat shock protein HSP 90 |
| Scutellariae Radix | acacetin | Cell division protein kinase 2 |
| Scutellariae Radix | acacetin | mRNA of PKA Catalytic Subunit C-alpha |
| Scutellariae Radix | acacetin | Trypsin-1 |
| Scutellariae Radix | acacetin | Nuclear receptor coactivator 2 |
| Scutellariae Radix | acacetin | Nuclear receptor coactivator 1 |
| Scutellariae Radix | acacetin | Calmodulin |
| Scutellariae Radix | acacetin | Phosphatidylinositol-4,5-bisphosphate 3-kinase catalytic subunit, gamma isoform |
| Scutellariae Radix | acacetin | Serine/threonine-protein kinase Chk1 |
| Scutellariae Radix | acacetin | Beta-2 adrenergic receptor |
| Scutellariae Radix | acacetin | CGMP-inhibited 3',5'-cyclic phosphodiesterase A |
| Scutellariae Radix | acacetin | Transcription factor p65 |
| Scutellariae Radix | acacetin | Apoptosis regulator Bcl-2 |
| Scutellariae Radix | acacetin | Cyclin-dependent kinase inhibitor 1 |
| Scutellariae Radix | acacetin | Apoptosis regulator BAX |
| Scutellariae Radix | acacetin | Caspase-3 |
| Scutellariae Radix | acacetin | Cellular tumor antigen p53 |
| Scutellariae Radix | acacetin | Caspase-8 |
| Scutellariae Radix | acacetin | Fatty acid synthase |
| Scutellariae Radix | acacetin | Tumor necrosis factor ligand superfamily member 6 |
| Scutellariae Radix | acacetin | Cytochrome P450 19A1 |
| Scutellariae Radix | wogonin | Nitric oxide synthase, inducible |
| Scutellariae Radix | wogonin | Prostaglandin G/H synthase 1 |
| Scutellariae Radix | wogonin | Estrogen receptor |
| Scutellariae Radix | wogonin | Androgen receptor |
| Scutellariae Radix | wogonin | Sodium channel protein type 5 subunit alpha |
| Scutellariae Radix | wogonin | Peroxisome proliferator activated receptor gamma |
| Scutellariae Radix | wogonin | Prostaglandin G/H synthase 2 |
| Scutellariae Radix | wogonin | Retinoic acid receptor RXR-alpha |
| Scutellariae Radix | wogonin | CGMP-inhibited 3',5'-cyclic phosphodiesterase A |
| Scutellariae Radix | wogonin | Dipeptidyl peptidase IV |
| Scutellariae Radix | wogonin | Mitogen-activated protein kinase 14 |
| Scutellariae Radix | wogonin | Glycogen synthase kinase-3 beta |
| Scutellariae Radix | wogonin | Heat shock protein HSP 90 |
| Scutellariae Radix | wogonin | Cell division protein kinase 2 |
| Scutellariae Radix | wogonin | Phosphatidylinositol-4,5-bisphosphate 3-kinase catalytic subunit, gamma isoform |
| Scutellariae Radix | wogonin | Serine/threonine-protein kinase Chk1 |
| Scutellariae Radix | wogonin | mRNA of PKA Catalytic Subunit C-alpha |
| Scutellariae Radix | wogonin | Trypsin-1 |
| Scutellariae Radix | wogonin | Calmodulin |
| Scutellariae Radix | wogonin | Beta-2 adrenergic receptor |
| Scutellariae Radix | wogonin | Gamma-aminobutyric acid receptor subunit alpha-1 |
| Scutellariae Radix | wogonin | Transcription factor p65 |
| Scutellariae Radix | wogonin | RAC-alpha serine/threonine-protein kinase |
| Scutellariae Radix | wogonin | G1/S-specific cyclin-D1 |
| Scutellariae Radix | wogonin | Apoptosis regulator Bcl-2 |
| Scutellariae Radix | wogonin | Cyclin-dependent kinase inhibitor 1 |
| Scutellariae Radix | wogonin | Eukaryotic translation initiation factor 6 |
| Scutellariae Radix | wogonin | Apoptosis regulator BAX |
| Scutellariae Radix | wogonin | Caspase-9 |
| Scutellariae Radix | wogonin | Vascular endothelial growth factor receptor 2 |
| Scutellariae Radix | wogonin | Tumor necrosis factor |
| Scutellariae Radix | wogonin | Transcription factor AP-1 |
| Scutellariae Radix | wogonin | Interleukin-6 |
| Scutellariae Radix | wogonin | Activator of 90 kDa heat shock protein ATPase homolog 1 |
| Scutellariae Radix | wogonin | Caspase-3 |
| Scutellariae Radix | wogonin | Cellular tumor antigen p53 |
| Scutellariae Radix | wogonin | Bcl-2-binding component 3 |
| Scutellariae Radix | wogonin | Telomerase protein component 1 |
| Scutellariae Radix | wogonin | Interstitial collagenase |
| Scutellariae Radix | wogonin | C-C motif chemokine 2 |
| Scutellariae Radix | wogonin | Protein kinase C delta type |
| Scutellariae Radix | wogonin | Prostaglandin E2 receptor EP3 subtype |
| Scutellariae Radix | wogonin | Fibronectin |
| Scutellariae Radix | wogonin | Interleukin-8 |
| Scutellariae Radix | wogonin | Induced myeloid leukemia cell differentiation protein Mcl-1 |
| Scutellariae Radix | Oroxindin | Thrombin |
| Scutellariae Radix | Oroxindin | Coagulation factor Xa |
| Scutellariae Radix | Oroxindin | Carbonic anhydrase II |
| Scutellariae Radix | Oroxindin | mRNA of Protein-tyrosine phosphatase, non-receptor type 1 |
| Scutellariae Radix | (+/-)-Isoborneol | Cytochrome P450-cam |
| Scutellariae Radix | (+/-)-Isoborneol | Gamma-aminobutyric-acid receptor alpha-2 subunit |
| Scutellariae Radix | (+/-)-Isoborneol | Muscarinic acetylcholine receptor M2 |
| Scutellariae Radix | (+/-)-Isoborneol | Gamma-aminobutyric acid receptor subunit alpha-1 |
| Scutellariae Radix | (+/-)-Isoborneol | Muscarinic acetylcholine receptor M3 |
| Scutellariae Radix | (+/-)-Isoborneol | Muscarinic acetylcholine receptor M1 |
| Scutellariae Radix | (+/-)-Isoborneol | Prostaglandin G/H synthase 2 |
| Scutellariae Radix | (+/-)-Isoborneol | Gamma-aminobutyric-acid receptor alpha-5 subunit |
| Scutellariae Radix | (+/-)-Isoborneol | Sodium-dependent noradrenaline transporter |
| Scutellariae Radix | (+/-)-Isoborneol | Alpha-1A adrenergic receptor |
| Scutellariae Radix | (+/-)-Isoborneol | Gamma-aminobutyric-acid receptor alpha-3 subunit |
| Scutellariae Radix | (+/-)-Isoborneol | Gamma-aminobutyric-acid receptor subunit alpha-6 |
| Scutellariae Radix | (+/-)-Isoborneol | Progesterone receptor |
| Scutellariae Radix | (R)-linalool | Muscarinic acetylcholine receptor M1 |
| Scutellariae Radix | (R)-linalool | Gamma-aminobutyric-acid receptor alpha-2 subunit |
| Scutellariae Radix | (R)-linalool | Muscarinic acetylcholine receptor M2 |
| Scutellariae Radix | (R)-linalool | Gamma-aminobutyric-acid receptor alpha-3 subunit |
| Scutellariae Radix | (R)-linalool | Gamma-aminobutyric-acid receptor subunit alpha-6 |
| Scutellariae Radix | (R)-linalool | Prostaglandin G/H synthase 1 |
| Scutellariae Radix | (R)-linalool | Potassium voltage-gated channel subfamily H member 2 |
| Scutellariae Radix | (R)-linalool | Sodium channel protein type 5 subunit alpha |
| Scutellariae Radix | (R)-linalool | Coagulation factor Xa |
| Scutellariae Radix | (R)-linalool | Prostaglandin G/H synthase 2 |
| Scutellariae Radix | (R)-linalool | Vascular endothelial growth factor receptor 2 |
| Scutellariae Radix | (R)-linalool | DNA topoisomerase II |
| Scutellariae Radix | (R)-linalool | Heat shock protein HSP 90 |
| Scutellariae Radix | (R)-linalool | Nuclear receptor coactivator 2 |
| Scutellariae Radix | (R)-linalool | Calcium-activated potassium channel subunit alpha 1 |
| Scutellariae Radix | (R)-linalool | Muscarinic acetylcholine receptor M3 |
| Scutellariae Radix | (R)-linalool | Gamma-aminobutyric-acid receptor alpha-5 subunit |
| Scutellariae Radix | (R)-linalool | Gamma-aminobutyric acid receptor subunit alpha-1 |
| Scutellariae Radix | (R)-linalool | Sodium-dependent noradrenaline transporter |
| Scutellariae Radix | (R)-linalool | Progesterone receptor |
| Scutellariae Radix | BOX | Amine oxidase [flavin-containing] B |
| Scutellariae Radix | BOX | Alcohol dehydrogenase 1B |
| Scutellariae Radix | BOX | Alcohol dehydrogenase 1C |
| Scutellariae Radix | BOX | Alcohol dehydrogenase 1A |
| Scutellariae Radix | BOX | Lysozyme |
| Scutellariae Radix | BOX | Bacillolysin |
| Scutellariae Radix | BOX | Nicotinate-nucleotide--dimethylbenzimidazole phosphoribosyltransferase |
| Scutellariae Radix | BOX | Group IIE secretory phospholipase A2 |
| Scutellariae Radix | BOX | Trypsin-3 |
| Scutellariae Radix | BOX | D-amino-acid oxidase |
| Scutellariae Radix | BOX | 2-hydroxy-6-oxo-7-methylocta-2,4-dienoate hydrolase |
| Scutellariae Radix | BOX | Muscarinic acetylcholine receptor M3 |
| Scutellariae Radix | BOX | Prostaglandin G/H synthase 2 |
| Scutellariae Radix | BOX | Gamma-aminobutyric-acid receptor alpha-2 subunit |
| Scutellariae Radix | BOX | Retinoic acid receptor RXR-alpha |
| Scutellariae Radix | BOX | Gamma-aminobutyric acid receptor subunit alpha-1 |
| Scutellariae Radix | BOX | Nuclear receptor coactivator 2 |
| Scutellariae Radix | (2R)-7-hydroxy-5-methoxy-2-phenylchroman-4-one | Prostaglandin G/H synthase 1 |
| Scutellariae Radix | (2R)-7-hydroxy-5-methoxy-2-phenylchroman-4-one | Dopamine D1 receptor |
| Scutellariae Radix | (2R)-7-hydroxy-5-methoxy-2-phenylchroman-4-one | Muscarinic acetylcholine receptor M3 |
| Scutellariae Radix | (2R)-7-hydroxy-5-methoxy-2-phenylchroman-4-one | Muscarinic acetylcholine receptor M1 |
| Scutellariae Radix | (2R)-7-hydroxy-5-methoxy-2-phenylchroman-4-one | Estrogen receptor |
| Scutellariae Radix | (2R)-7-hydroxy-5-methoxy-2-phenylchroman-4-one | Sodium channel protein type 5 subunit alpha |
| Scutellariae Radix | (2R)-7-hydroxy-5-methoxy-2-phenylchroman-4-one | Prostaglandin G/H synthase 2 |
| Scutellariae Radix | (2R)-7-hydroxy-5-methoxy-2-phenylchroman-4-one | Retinoic acid receptor RXR-alpha |
| Scutellariae Radix | (2R)-7-hydroxy-5-methoxy-2-phenylchroman-4-one | CGMP-inhibited 3',5'-cyclic phosphodiesterase A |
| Scutellariae Radix | (2R)-7-hydroxy-5-methoxy-2-phenylchroman-4-one | Alpha-1A adrenergic receptor |
| Scutellariae Radix | (2R)-7-hydroxy-5-methoxy-2-phenylchroman-4-one | Alpha-1B adrenergic receptor |
| Scutellariae Radix | (2R)-7-hydroxy-5-methoxy-2-phenylchroman-4-one | Sodium-dependent dopamine transporter |
| Scutellariae Radix | (2R)-7-hydroxy-5-methoxy-2-phenylchroman-4-one | Beta-2 adrenergic receptor |
| Scutellariae Radix | (2R)-7-hydroxy-5-methoxy-2-phenylchroman-4-one | Sodium-dependent serotonin transporter |
| Scutellariae Radix | (2R)-7-hydroxy-5-methoxy-2-phenylchroman-4-one | Gamma-aminobutyric acid receptor subunit alpha-1 |
| Scutellariae Radix | (2R)-7-hydroxy-5-methoxy-2-phenylchroman-4-one | Heat shock protein HSP 90 |
| Scutellariae Radix | (2R)-7-hydroxy-5-methoxy-2-phenylchroman-4-one | Phosphatidylinositol-4,5-bisphosphate 3-kinase catalytic subunit, gamma isoform |
| Scutellariae Radix | (2R)-7-hydroxy-5-methoxy-2-phenylchroman-4-one | mRNA of PKA Catalytic Subunit C-alpha |
| Scutellariae Radix | (2R)-7-hydroxy-5-methoxy-2-phenylchroman-4-one | cAMP-dependent protein kinase inhibitor alpha |
| Scutellariae Radix | (2R)-7-hydroxy-5-methoxy-2-phenylchroman-4-one | Neuronal acetylcholine receptor protein, alpha-7 chain |
| Scutellariae Radix | (2R)-7-hydroxy-5-methoxy-2-phenylchroman-4-one | Amine oxidase [flavin-containing] B |
| Scutellariae Radix | (2R)-7-hydroxy-5-methoxy-2-phenylchroman-4-one | Calmodulin |
| Scutellariae Radix | alpha-humulene | Prostaglandin G/H synthase 2 |
| Scutellariae Radix | alpha-humulene | Sodium-dependent noradrenaline transporter |
| Scutellariae Radix | alpha-humulene | Gamma-aminobutyric acid receptor subunit alpha-1 |
| Scutellariae Radix | alpha-humulene | Nuclear receptor coactivator 2 |
| Scutellariae Radix | alpha-humulene | Renin, renal |
| Scutellariae Radix | alpha-humulene | Alcohol dehydrogenase 1B |
| Scutellariae Radix | alpha-humulene | Alcohol dehydrogenase 1C |
| Scutellariae Radix | alpha-humulene | Tumor necrosis factor |
| Scutellariae Radix | alpha-humulene | Interleukin-1 beta |
| Scutellariae Radix | eugenol | Prostaglandin G/H synthase 1 |
| Scutellariae Radix | eugenol | Dopamine D1 receptor |
| Scutellariae Radix | eugenol | Muscarinic acetylcholine receptor M3 |
| Scutellariae Radix | eugenol | Muscarinic acetylcholine receptor M1 |
| Scutellariae Radix | eugenol | Beta-1 adrenergic receptor |
| Scutellariae Radix | eugenol | Prostaglandin G/H synthase 2 |
| Scutellariae Radix | eugenol | Nitric-oxide synthase, endothelial |
| Scutellariae Radix | eugenol | Alpha-2A adrenergic receptor |
| Scutellariae Radix | eugenol | Alpha-2C adrenergic receptor |
| Scutellariae Radix | eugenol | Sodium-dependent noradrenaline transporter |
| Scutellariae Radix | eugenol | Alpha-1A adrenergic receptor |
| Scutellariae Radix | eugenol | Muscarinic acetylcholine receptor M2 |
| Scutellariae Radix | eugenol | Alpha-1B adrenergic receptor |
| Scutellariae Radix | eugenol | Sodium-dependent dopamine transporter |
| Scutellariae Radix | eugenol | Beta-2 adrenergic receptor |
| Scutellariae Radix | eugenol | Beta-lactamase |
| Scutellariae Radix | eugenol | Amine oxidase [flavin-containing] B |
| Scutellariae Radix | eugenol | Amine oxidase [flavin-containing] A |
| Scutellariae Radix | eugenol | Lysozyme |
| Scutellariae Radix | eugenol | Chymotrypsinogen B |
| Scutellariae Radix | eugenol | Alpha-1D adrenergic receptor |
| Scutellariae Radix | eugenol | Leukotriene A-4 hydrolase |
| Scutellariae Radix | eugenol | Alpha-2B adrenergic receptor |
| Scutellariae Radix | eugenol | Urokinase-type plasminogen activator |
| Scutellariae Radix | eugenol | Thermolysin |
| Scutellariae Radix | eugenol | Sodium channel protein type 5 subunit alpha |
| Scutellariae Radix | eugenol | Transcription factor p65 |
| Scutellariae Radix | eugenol | Cytochrome P450 1A1 |
| Scutellariae Radix | eugenol | Cytochrome P450 1B1 |
| Scutellariae Radix | eugenol | Arachidonate 5-lipoxygenase |
| Scutellariae Radix | eugenol | Aryl hydrocarbon receptor |
| Scutellariae Radix | eugenol | Canalicular multispecific organic anion transporter 1 |
| Scutellariae Radix | eugenol | T-lymphocyte activation antigen CD86 |
| Scutellariae Radix | eugenol | Serine/threonine-protein phosphatase 2B catalytic subunit alpha isoform |
| Scutellariae Radix | eugenol | Pepsin A |
| Scutellariae Radix | eugenol | Mucin-1 |
| Scutellariae Radix | eugenol | Quinone oxidoreductase |
| Scutellariae Radix | eugenol | Calcium-transporting ATPase type 2C member 1 |
| Scutellariae Radix | eugenol | Short transient receptor potential channel 3 |
| Scutellariae Radix | eugenol | Transient receptor potential cation channel subfamily V member 3 |
| Scutellariae Radix | eugenol | Ecto-NOX disulfide-thiol exchanger 2 |
| Scutellariae Radix | chrysin | Prostaglandin G/H synthase 1 |
| Scutellariae Radix | chrysin | Androgen receptor |
| Scutellariae Radix | chrysin | Prostaglandin G/H synthase 2 |
| Scutellariae Radix | chrysin | CGMP-inhibited 3',5'-cyclic phosphodiesterase A |
| Scutellariae Radix | chrysin | Sodium-dependent serotonin transporter |
| Scutellariae Radix | chrysin | Gamma-aminobutyric acid receptor subunit alpha-1 |
| Scutellariae Radix | chrysin | Dipeptidyl peptidase IV |
| Scutellariae Radix | chrysin | Heat shock protein HSP 90 |
| Scutellariae Radix | chrysin | Phosphatidylinositol-4,5-bisphosphate 3-kinase catalytic subunit, gamma isoform |
| Scutellariae Radix | chrysin | Amine oxidase [flavin-containing] B |
| Scutellariae Radix | chrysin | mRNA of PKA Catalytic Subunit C-alpha |
| Scutellariae Radix | chrysin | cAMP-dependent protein kinase inhibitor alpha |
| Scutellariae Radix | chrysin | Thrombin |
| Scutellariae Radix | chrysin | Cyclin-dependent kinase inhibitor 1 |
| Scutellariae Radix | chrysin | Transforming growth factor beta-1 |
| Scutellariae Radix | chrysin | Interleukin-4 |
| Scutellariae Radix | chrysin | Cytochrome P450 19A1 |
| Scutellariae Radix | chrysin | Interleukin-13 |
| Scutellariae Radix | chrysin | High affinity immunoglobulin epsilon receptor subunit beta |
| Scutellariae Radix | 尾-patchoulene | Muscarinic acetylcholine receptor M3 |
| Scutellariae Radix | 尾-patchoulene | Prostaglandin G/H synthase 2 |
| Scutellariae Radix | 尾-patchoulene | Gamma-aminobutyric acid receptor subunit alpha-1 |
| Scutellariae Radix | 尾-patchoulene | Nuclear receptor coactivator 2 |
| Scutellariae Radix | 尾-patchoulene | Gamma-aminobutyric-acid receptor alpha-2 subunit |
| Scutellariae Radix | 尾-patchoulene | Gamma-aminobutyric-acid receptor subunit alpha-6 |
| Scutellariae Radix | baicalein | Prostaglandin G/H synthase 1 |
| Scutellariae Radix | baicalein | Androgen receptor |
| Scutellariae Radix | baicalein | Prostaglandin G/H synthase 2 |
| Scutellariae Radix | baicalein | Heat shock protein HSP 90 |
| Scutellariae Radix | baicalein | mRNA of PKA Catalytic Subunit C-alpha |
| Scutellariae Radix | baicalein | Dipeptidyl peptidase IV |
| Scutellariae Radix | baicalein | Phosphatidylinositol-4,5-bisphosphate 3-kinase catalytic subunit, gamma isoform |
| Scutellariae Radix | baicalein | CGMP-inhibited 3',5'-cyclic phosphodiesterase A |
| Scutellariae Radix | baicalein | Trypsin-1 |
| Scutellariae Radix | baicalein | Nuclear receptor coactivator 2 |
| Scutellariae Radix | baicalein | Nuclear receptor coactivator 1 |
| Scutellariae Radix | baicalein | Calmodulin |
| Scutellariae Radix | baicalein | Transcription factor p65 |
| Scutellariae Radix | baicalein | RAC-alpha serine/threonine-protein kinase |
| Scutellariae Radix | baicalein | Vascular endothelial growth factor A |
| Scutellariae Radix | baicalein | Apoptosis regulator Bcl-2 |
| Scutellariae Radix | baicalein | Proto-oncogene c-Fos |
| Scutellariae Radix | baicalein | Apoptosis regulator BAX |
| Scutellariae Radix | baicalein | Matrix metalloproteinase-9 |
| Scutellariae Radix | baicalein | Caspase-3 |
| Scutellariae Radix | baicalein | Cellular tumor antigen p53 |
| Scutellariae Radix | baicalein | Hypoxia-inducible factor 1-alpha |
| Scutellariae Radix | baicalein | Fos-related antigen 1 |
| Scutellariae Radix | baicalein | Fos-related antigen 2 |
| Scutellariae Radix | baicalein | Cell division control protein 2 homolog |
| Scutellariae Radix | baicalein | G2/mitotic-specific cyclin-B1 |
| Scutellariae Radix | baicalein | Myeloperoxidase |
| Scutellariae Radix | baicalein | Aryl hydrocarbon receptor |
| Scutellariae Radix | baicalein | Insulin-like growth factor II |
| Scutellariae Radix | baicalein | Cytochrome c |
| Scutellariae Radix | baicalein | Arachidonate 12-lipoxygenase, 12S-type |
| Scutellariae Radix | baicalein | Nuclear factor of activated T-cells, cytoplasmic 1 |
| Scutellariae Radix | baicalein | Tudor domain-containing protein 7 |
| Scutellariae Radix | baicalein | Egl nine homolog 1 |
| Scutellariae Radix | baicalein | NADPH oxidase 5 |
| Scutellariae Radix | baicalein | Fatty acid-binding protein, epidermal |
| Scutellariae Radix | baicalein | Apolipoprotein D |
| Scutellariae Radix | scutellarein | Prostaglandin G/H synthase 1 |
| Scutellariae Radix | scutellarein | Androgen receptor |
| Scutellariae Radix | scutellarein | Prostaglandin G/H synthase 2 |
| Scutellariae Radix | scutellarein | Heat shock protein HSP 90 |
| Scutellariae Radix | scutellarein | Phosphatidylinositol-4,5-bisphosphate 3-kinase catalytic subunit, gamma isoform |
| Scutellariae Radix | scutellarein | mRNA of PKA Catalytic Subunit C-alpha |
| Scutellariae Radix | scutellarein | Trypsin-1 |
| Scutellariae Radix | scutellarein | Nuclear receptor coactivator 2 |
| Scutellariae Radix | scutellarein | Vascular endothelial growth factor A |
| Scutellariae Radix | 5,7,2,5-tetrahydroxy-8,6-dimethoxyflavone | Nitric oxide synthase, inducible |
| Scutellariae Radix | 5,7,2,5-tetrahydroxy-8,6-dimethoxyflavone | Thrombin |
| Scutellariae Radix | 5,7,2,5-tetrahydroxy-8,6-dimethoxyflavone | Androgen receptor |
| Scutellariae Radix | 5,7,2,5-tetrahydroxy-8,6-dimethoxyflavone | Coagulation factor Xa |
| Scutellariae Radix | 5,7,2,5-tetrahydroxy-8,6-dimethoxyflavone | Prostaglandin G/H synthase 2 |
| Scutellariae Radix | 5,7,2,5-tetrahydroxy-8,6-dimethoxyflavone | mRNA of Protein-tyrosine phosphatase, non-receptor type 1 |
| Scutellariae Radix | 5,7,2,5-tetrahydroxy-8,6-dimethoxyflavone | DNA topoisomerase II |
| Scutellariae Radix | 5,7,2,5-tetrahydroxy-8,6-dimethoxyflavone | Dipeptidyl peptidase IV |
| Scutellariae Radix | 5,7,2,5-tetrahydroxy-8,6-dimethoxyflavone | Glycogen phosphorylase, muscle form |
| Scutellariae Radix | 5,7,2,5-tetrahydroxy-8,6-dimethoxyflavone | Heat shock protein HSP 90 |
| Scutellariae Radix | 5,7,2,5-tetrahydroxy-8,6-dimethoxyflavone | Trypsin-1 |
| Scutellariae Radix | 5,7,2,5-tetrahydroxy-8,6-dimethoxyflavone | Nuclear receptor coactivator 2 |
| Scutellariae Radix | 5,7,2,5-tetrahydroxy-8,6-dimethoxyflavone | Calcium-activated potassium channel subunit alpha 1 |
| Scutellariae Radix | Carthamidin | Prostaglandin G/H synthase 1 |
| Scutellariae Radix | Carthamidin | Prostaglandin G/H synthase 2 |
| Scutellariae Radix | Carthamidin | Heat shock protein HSP 90 |
| Scutellariae Radix | Carthamidin | mRNA of PKA Catalytic Subunit C-alpha |
| Scutellariae Radix | Dihydrobaicalin | Coagulation factor Xa |
| Scutellariae Radix | Dihydrobaicalin_qt | Prostaglandin G/H synthase 1 |
| Scutellariae Radix | Dihydrobaicalin_qt | Prostaglandin G/H synthase 2 |
| Scutellariae Radix | Dihydrobaicalin_qt | Heat shock protein HSP 90 |
| Scutellariae Radix | Dihydrobaicalin_qt | mRNA of PKA Catalytic Subunit C-alpha |
| Scutellariae Radix | Eriodyctiol (flavanone) | Prostaglandin G/H synthase 1 |
| Scutellariae Radix | Eriodyctiol (flavanone) | Prostaglandin G/H synthase 2 |
| Scutellariae Radix | Eriodyctiol (flavanone) | Heat shock protein HSP 90 |
| Scutellariae Radix | Eriodyctiol (flavanone) | mRNA of PKA Catalytic Subunit C-alpha |
| Scutellariae Radix | Eriodyctiol (flavanone) | Nuclear receptor coactivator 2 |
| Scutellariae Radix | Eriodyctiol (flavanone) | Phosphatidylinositol-4,5-bisphosphate 3-kinase catalytic subunit, gamma isoform |
| Scutellariae Radix | Eriodyctiol (flavanone) | Glycogen phosphorylase, muscle form |
| Scutellariae Radix | Eriodyctiol (flavanone) | Calmodulin |
| Scutellariae Radix | Salvigenin | Nitric oxide synthase, inducible |
| Scutellariae Radix | Salvigenin | Prostaglandin G/H synthase 1 |
| Scutellariae Radix | Salvigenin | Thrombin |
| Scutellariae Radix | Salvigenin | Sodium channel protein type 5 subunit alpha |
| Scutellariae Radix | Salvigenin | Coagulation factor Xa |
| Scutellariae Radix | Salvigenin | Prostaglandin G/H synthase 2 |
| Scutellariae Radix | Salvigenin | Nitric-oxide synthase, endothelial |
| Scutellariae Radix | Salvigenin | Retinoic acid receptor RXR-alpha |
| Scutellariae Radix | Salvigenin | Acetylcholinesterase |
| Scutellariae Radix | Salvigenin | Alpha-1B adrenergic receptor |
| Scutellariae Radix | Salvigenin | Beta-2 adrenergic receptor |
| Scutellariae Radix | Salvigenin | Dipeptidyl peptidase IV |
| Scutellariae Radix | Salvigenin | Heat shock protein HSP 90 |
| Scutellariae Radix | Salvigenin | Ig gamma-1 chain C region |
| Scutellariae Radix | Salvigenin | Trypsin-1 |
| Scutellariae Radix | Salvigenin | Nuclear receptor coactivator 2 |
| Scutellariae Radix | Salvigenin | Calmodulin |
| Scutellariae Radix | Salvigenin | Coagulation factor VII |
| Scutellariae Radix | 2-(2,6-dihydroxyphenyl)-3,5,7-trihydroxy-chromone | Nitric oxide synthase, inducible |
| Scutellariae Radix | 2-(2,6-dihydroxyphenyl)-3,5,7-trihydroxy-chromone | Prostaglandin G/H synthase 1 |
| Scutellariae Radix | 2-(2,6-dihydroxyphenyl)-3,5,7-trihydroxy-chromone | Androgen receptor |
| Scutellariae Radix | 2-(2,6-dihydroxyphenyl)-3,5,7-trihydroxy-chromone | Peroxisome proliferator activated receptor gamma |
| Scutellariae Radix | 2-(2,6-dihydroxyphenyl)-3,5,7-trihydroxy-chromone | Prostaglandin G/H synthase 2 |
| Scutellariae Radix | 2-(2,6-dihydroxyphenyl)-3,5,7-trihydroxy-chromone | Heat shock protein HSP 90 |
| Scutellariae Radix | 2-(2,6-dihydroxyphenyl)-3,5,7-trihydroxy-chromone | Phosphatidylinositol-4,5-bisphosphate 3-kinase catalytic subunit, gamma isoform |
| Scutellariae Radix | 5,2',6'-Trihydroxy-7,8-dimethoxyflavone | Nitric oxide synthase, inducible |
| Scutellariae Radix | 5,2',6'-Trihydroxy-7,8-dimethoxyflavone | Prostaglandin G/H synthase 1 |
| Scutellariae Radix | 5,2',6'-Trihydroxy-7,8-dimethoxyflavone | Androgen receptor |
| Scutellariae Radix | 5,2',6'-Trihydroxy-7,8-dimethoxyflavone | Sodium channel protein type 5 subunit alpha |
| Scutellariae Radix | 5,2',6'-Trihydroxy-7,8-dimethoxyflavone | Coagulation factor Xa |
| Scutellariae Radix | 5,2',6'-Trihydroxy-7,8-dimethoxyflavone | Prostaglandin G/H synthase 2 |
| Scutellariae Radix | 5,2',6'-Trihydroxy-7,8-dimethoxyflavone | DNA topoisomerase II |
| Scutellariae Radix | 5,2',6'-Trihydroxy-7,8-dimethoxyflavone | Estrogen receptor beta |
| Scutellariae Radix | 5,2',6'-Trihydroxy-7,8-dimethoxyflavone | Dipeptidyl peptidase IV |
| Scutellariae Radix | 5,2',6'-Trihydroxy-7,8-dimethoxyflavone | Heat shock protein HSP 90 |
| Scutellariae Radix | 5,2',6'-Trihydroxy-7,8-dimethoxyflavone | Cell division protein kinase 2 |
| Scutellariae Radix | 5,2',6'-Trihydroxy-7,8-dimethoxyflavone | Phosphatidylinositol-4,5-bisphosphate 3-kinase catalytic subunit, gamma isoform |
| Scutellariae Radix | 5,2',6'-Trihydroxy-7,8-dimethoxyflavone | Serine/threonine-protein kinase Chk1 |
| Scutellariae Radix | 5,2',6'-Trihydroxy-7,8-dimethoxyflavone | Trypsin-1 |
| Scutellariae Radix | 5,2',6'-Trihydroxy-7,8-dimethoxyflavone | Nuclear receptor coactivator 2 |
| Scutellariae Radix | 5,2',6'-Trihydroxy-7,8-dimethoxyflavone | Calmodulin |
| Scutellariae Radix | 5,2',6'-Trihydroxy-7,8-dimethoxyflavone | Calcium-activated potassium channel subunit alpha 1 |
| Scutellariae Radix | Ganhuangenin | Nitric oxide synthase, inducible |
| Scutellariae Radix | Ganhuangenin | Thrombin |
| Scutellariae Radix | Ganhuangenin | Androgen receptor |
| Scutellariae Radix | Ganhuangenin | Coagulation factor Xa |
| Scutellariae Radix | Ganhuangenin | Prostaglandin G/H synthase 2 |
| Scutellariae Radix | Ganhuangenin | Coagulation factor VII |
| Scutellariae Radix | Ganhuangenin | mRNA of Protein-tyrosine phosphatase, non-receptor type 1 |
| Scutellariae Radix | Ganhuangenin | DNA topoisomerase II |
| Scutellariae Radix | Ganhuangenin | Estrogen receptor beta |
| Scutellariae Radix | Ganhuangenin | Dipeptidyl peptidase IV |
| Scutellariae Radix | Ganhuangenin | Trypsin-1 |
| Scutellariae Radix | Ganhuangenin | Calcium-activated potassium channel subunit alpha 1 |
| Scutellariae Radix | Viscidulin III | Nitric oxide synthase, inducible |
| Scutellariae Radix | Viscidulin III | Thrombin |
| Scutellariae Radix | Viscidulin III | Androgen receptor |
| Scutellariae Radix | Viscidulin III | Sodium channel protein type 5 subunit alpha |
| Scutellariae Radix | Viscidulin III | Coagulation factor Xa |
| Scutellariae Radix | Viscidulin III | Prostaglandin G/H synthase 2 |
| Scutellariae Radix | Viscidulin III | Coagulation factor VII |
| Scutellariae Radix | Viscidulin III | DNA topoisomerase II |
| Scutellariae Radix | Viscidulin III | Dipeptidyl peptidase IV |
| Scutellariae Radix | Viscidulin III | Heat shock protein HSP 90 |
| Scutellariae Radix | Viscidulin III | Trypsin-1 |
| Scutellariae Radix | Viscidulin III | Nuclear receptor coactivator 2 |
| Scutellariae Radix | Viscidulin III | Calmodulin |
| Scutellariae Radix | alpha-Guaiene | Prostaglandin G/H synthase 1 |
| Scutellariae Radix | alpha-Guaiene | Muscarinic acetylcholine receptor M1 |
| Scutellariae Radix | alpha-Guaiene | Prostaglandin G/H synthase 2 |
| Scutellariae Radix | alpha-Guaiene | Gamma-aminobutyric-acid receptor alpha-2 subunit |
| Scutellariae Radix | alpha-Guaiene | Retinoic acid receptor RXR-alpha |
| Scutellariae Radix | alpha-Guaiene | Sodium-dependent noradrenaline transporter |
| Scutellariae Radix | alpha-Guaiene | Gamma-aminobutyric acid receptor subunit alpha-1 |
| Scutellariae Radix | alpha-Guaiene | Amine oxidase [flavin-containing] B |
| Scutellariae Radix | alpha-Guaiene | Nuclear receptor coactivator 2 |
| Scutellariae Radix | alpha-Guaiene | Gamma-aminobutyric-acid receptor subunit alpha-6 |
| Scutellariae Radix | alpha-Guaiene | Muscarinic acetylcholine receptor M3 |
| Scutellariae Radix | alpha-Guaiene | CGMP-inhibited 3',5'-cyclic phosphodiesterase A |
| Scutellariae Radix | alpha-Guaiene | 5-hydroxytryptamine 2A receptor |
| Scutellariae Radix | alpha-Guaiene | Alpha-1B adrenergic receptor |
| Scutellariae Radix | alpha-Guaiene | Beta-2 adrenergic receptor |
| Scutellariae Radix | alpha-Guaiene | Sodium-dependent serotonin transporter |
| Scutellariae Radix | 5-(2-hydroxyethyl)-2-methoxyphenol | Prostaglandin G/H synthase 1 |
| Scutellariae Radix | 5-(2-hydroxyethyl)-2-methoxyphenol | Beta-1 adrenergic receptor |
| Scutellariae Radix | 5-(2-hydroxyethyl)-2-methoxyphenol | Prostaglandin G/H synthase 2 |
| Scutellariae Radix | 5-(2-hydroxyethyl)-2-methoxyphenol | Alpha-2A adrenergic receptor |
| Scutellariae Radix | 5-(2-hydroxyethyl)-2-methoxyphenol | Alpha-2C adrenergic receptor |
| Scutellariae Radix | 5-(2-hydroxyethyl)-2-methoxyphenol | Sodium-dependent noradrenaline transporter |
| Scutellariae Radix | 5-(2-hydroxyethyl)-2-methoxyphenol | Alpha-1A adrenergic receptor |
| Scutellariae Radix | 5-(2-hydroxyethyl)-2-methoxyphenol | Alpha-2B adrenergic receptor |
| Scutellariae Radix | 5-(2-hydroxyethyl)-2-methoxyphenol | Sodium-dependent dopamine transporter |
| Scutellariae Radix | 5-(2-hydroxyethyl)-2-methoxyphenol | Beta-2 adrenergic receptor |
| Scutellariae Radix | 5-(2-hydroxyethyl)-2-methoxyphenol | Beta-lactamase |
| Scutellariae Radix | 5-(2-hydroxyethyl)-2-methoxyphenol | Amine oxidase [flavin-containing] B |
| Scutellariae Radix | 5-(2-hydroxyethyl)-2-methoxyphenol | Amine oxidase [flavin-containing] A |
| Scutellariae Radix | 5-(2-hydroxyethyl)-2-methoxyphenol | Lysozyme |
| Scutellariae Radix | 5-(2-hydroxyethyl)-2-methoxyphenol | Muscarinic acetylcholine receptor M1 |
| Scutellariae Radix | darendoside B | Thrombin |
| Scutellariae Radix | darendoside B_qt | Nitric oxide synthase, inducible |
| Scutellariae Radix | darendoside B_qt | Thrombin |
| Scutellariae Radix | darendoside B_qt | Estrogen receptor |
| Scutellariae Radix | darendoside B_qt | Prostaglandin G/H synthase 2 |
| Scutellariae Radix | darendoside B_qt | Dipeptidyl peptidase IV |
| Scutellariae Radix | darendoside B_qt | Cell division protein kinase 2 |
| Scutellariae Radix | darendoside B_qt | Beta-lactamase |
| Scutellariae Radix | 5,7,2',6'-Tetrahydroxyflavone | Prostaglandin G/H synthase 1 |
| Scutellariae Radix | 5,7,2',6'-Tetrahydroxyflavone | Androgen receptor |
| Scutellariae Radix | 5,7,2',6'-Tetrahydroxyflavone | Prostaglandin G/H synthase 2 |
| Scutellariae Radix | 5,7,2',6'-Tetrahydroxyflavone | Dipeptidyl peptidase IV |
| Scutellariae Radix | 5,7,2',6'-Tetrahydroxyflavone | Heat shock protein HSP 90 |
| Scutellariae Radix | 5,7,2',6'-Tetrahydroxyflavone | mRNA of PKA Catalytic Subunit C-alpha |
| Scutellariae Radix | Skullcapflavone II | Nitric oxide synthase, inducible |
| Scutellariae Radix | Skullcapflavone II | Prostaglandin G/H synthase 1 |
| Scutellariae Radix | Skullcapflavone II | Thrombin |
| Scutellariae Radix | Skullcapflavone II | Potassium voltage-gated channel subfamily H member 2 |
| Scutellariae Radix | Skullcapflavone II | Androgen receptor |
| Scutellariae Radix | Skullcapflavone II | Sodium channel protein type 5 subunit alpha |
| Scutellariae Radix | Skullcapflavone II | Coagulation factor Xa |
| Scutellariae Radix | Skullcapflavone II | Prostaglandin G/H synthase 2 |
| Scutellariae Radix | Skullcapflavone II | Nitric-oxide synthase, endothelial |
| Scutellariae Radix | Skullcapflavone II | Coagulation factor VII |
| Scutellariae Radix | Skullcapflavone II | Vascular endothelial growth factor receptor 2 |
| Scutellariae Radix | Skullcapflavone II | Voltage-dependent calcium channel subunit alpha-2/delta-1 |
| Scutellariae Radix | Skullcapflavone II | DNA topoisomerase II |
| Scutellariae Radix | Skullcapflavone II | Dipeptidyl peptidase IV |
| Scutellariae Radix | Skullcapflavone II | Heat shock protein HSP 90 |
| Scutellariae Radix | Skullcapflavone II | Ig gamma-1 chain C region |
| Scutellariae Radix | Skullcapflavone II | Trypsin-1 |
| Scutellariae Radix | Skullcapflavone II | Nuclear receptor coactivator 2 |
| Scutellariae Radix | Skullcapflavone II | Nuclear receptor coactivator 1 |
| Scutellariae Radix | Skullcapflavone II | Calcium-activated potassium channel subunit alpha 1 |
| Scutellariae Radix | Skullcapflavone II | Calmodulin |
| Scutellariae Radix | oroxylin a | Nitric oxide synthase, inducible |
| Scutellariae Radix | oroxylin a | Prostaglandin G/H synthase 1 |
| Scutellariae Radix | oroxylin a | Androgen receptor |
| Scutellariae Radix | oroxylin a | Sodium channel protein type 5 subunit alpha |
| Scutellariae Radix | oroxylin a | Prostaglandin G/H synthase 2 |
| Scutellariae Radix | oroxylin a | Retinoic acid receptor RXR-alpha |
| Scutellariae Radix | oroxylin a | CGMP-inhibited 3',5'-cyclic phosphodiesterase A |
| Scutellariae Radix | oroxylin a | Alpha-1B adrenergic receptor |
| Scutellariae Radix | oroxylin a | Beta-2 adrenergic receptor |
| Scutellariae Radix | oroxylin a | Dipeptidyl peptidase IV |
| Scutellariae Radix | oroxylin a | Heat shock protein HSP 90 |
| Scutellariae Radix | oroxylin a | Phosphatidylinositol-4,5-bisphosphate 3-kinase catalytic subunit, gamma isoform |
| Scutellariae Radix | oroxylin a | mRNA of PKA Catalytic Subunit C-alpha |
| Scutellariae Radix | oroxylin a | Trypsin-1 |
| Scutellariae Radix | oroxylin a | Nuclear receptor coactivator 1 |
| Scutellariae Radix | oroxylin a | Calmodulin |
| Scutellariae Radix | oroxylin a | Nuclear receptor coactivator 2 |
| Scutellariae Radix | oroxylin a | cAMP-dependent protein kinase inhibitor alpha |
| Scutellariae Radix | oroxylin a | Apoptosis regulator Bcl-2 |
| Scutellariae Radix | oroxylin a | Interleukin-6 |
| Scutellariae Radix | oroxylin a | Caspase-3 |
| Scutellariae Radix | oroxylin a | Cell division control protein 2 homolog |
| Scutellariae Radix | oroxylin a | Cytochrome P450 1A2 |
| Scutellariae Radix | oroxylin a | G2/mitotic-specific cyclin-B1 |
| Scutellariae Radix | oroxylin a | Cell division protein kinase 7 |
| Scutellariae Radix | oroxylin a | Cytochrome P450 2C9 |
| Scutellariae Radix | Tyrosol | Beta-1 adrenergic receptor |
| Scutellariae Radix | Tyrosol | Sodium-dependent noradrenaline transporter |
| Scutellariae Radix | Tyrosol | Alpha-1A adrenergic receptor |
| Scutellariae Radix | Tyrosol | Sodium-dependent dopamine transporter |
| Scutellariae Radix | Tyrosol | Beta-lactamase |
| Scutellariae Radix | Tyrosol | Amine oxidase [flavin-containing] A |
| Scutellariae Radix | Tyrosol | Alcohol dehydrogenase 1C |
| Scutellariae Radix | Tyrosol | Lysozyme |
| Scutellariae Radix | Tyrosol | Bacillolysin |
| Scutellariae Radix | Tyrosol | Nicotinate-nucleotide--dimethylbenzimidazole phosphoribosyltransferase |
| Scutellariae Radix | Tyrosol | Trypsin-3 |
| Scutellariae Radix | Tyrosol | Alpha-1B adrenergic receptor |
| Scutellariae Radix | Tyrosol | Amine oxidase [flavin-containing] B |
| Scutellariae Radix | Tyrosol | DNA topoisomerase II |
| Scutellariae Radix | scutellarin | Coagulation factor Xa |
| Scutellariae Radix | scutellarin | mRNA of Protein-tyrosine phosphatase, non-receptor type 1 |
| Scutellariae Radix | scutellarin | Bcl-2-like protein 1 |
| Scutellariae Radix | scutellarin | Activator of 90 kDa heat shock protein ATPase homolog 1 |
| Scutellariae Radix | scutellarin | Caspase-3 |
| Scutellariae Radix | scutellarin | Protein kinase C gamma type |
| Scutellariae Radix | Panicolin | Nitric oxide synthase, inducible |
| Scutellariae Radix | Panicolin | Prostaglandin G/H synthase 1 |
| Scutellariae Radix | Panicolin | Androgen receptor |
| Scutellariae Radix | Panicolin | Sodium channel protein type 5 subunit alpha |
| Scutellariae Radix | Panicolin | Prostaglandin G/H synthase 2 |
| Scutellariae Radix | Panicolin | Estrogen receptor beta |
| Scutellariae Radix | Panicolin | Dipeptidyl peptidase IV |
| Scutellariae Radix | Panicolin | Heat shock protein HSP 90 |
| Scutellariae Radix | Panicolin | Cell division protein kinase 2 |
| Scutellariae Radix | Panicolin | Serine/threonine-protein kinase Chk1 |
| Scutellariae Radix | Panicolin | Trypsin-1 |
| Scutellariae Radix | Panicolin | Calmodulin |
| Scutellariae Radix | Panicolin | Phosphatidylinositol-4,5-bisphosphate 3-kinase catalytic subunit, gamma isoform |
| Scutellariae Radix | Panicolin | Nuclear receptor coactivator 1 |
| Scutellariae Radix | 5,7,4'-Trihydroxy-8-methoxyflavone | Nitric oxide synthase, inducible |
| Scutellariae Radix | 5,7,4'-Trihydroxy-8-methoxyflavone | Prostaglandin G/H synthase 1 |
| Scutellariae Radix | 5,7,4'-Trihydroxy-8-methoxyflavone | Estrogen receptor |
| Scutellariae Radix | 5,7,4'-Trihydroxy-8-methoxyflavone | Androgen receptor |
| Scutellariae Radix | 5,7,4'-Trihydroxy-8-methoxyflavone | Peroxisome proliferator activated receptor gamma |
| Scutellariae Radix | 5,7,4'-Trihydroxy-8-methoxyflavone | Prostaglandin G/H synthase 2 |
| Scutellariae Radix | 5,7,4'-Trihydroxy-8-methoxyflavone | Dipeptidyl peptidase IV |
| Scutellariae Radix | 5,7,4'-Trihydroxy-8-methoxyflavone | Glycogen phosphorylase, muscle form |
| Scutellariae Radix | 5,7,4'-Trihydroxy-8-methoxyflavone | Mitogen-activated protein kinase 14 |
| Scutellariae Radix | 5,7,4'-Trihydroxy-8-methoxyflavone | Glycogen synthase kinase-3 beta |
| Scutellariae Radix | 5,7,4'-Trihydroxy-8-methoxyflavone | Heat shock protein HSP 90 |
| Scutellariae Radix | 5,7,4'-Trihydroxy-8-methoxyflavone | Cell division protein kinase 2 |
| Scutellariae Radix | 5,7,4'-Trihydroxy-8-methoxyflavone | Phosphatidylinositol-4,5-bisphosphate 3-kinase catalytic subunit, gamma isoform |
| Scutellariae Radix | 5,7,4'-Trihydroxy-8-methoxyflavone | Serine/threonine-protein kinase Chk1 |
| Scutellariae Radix | 5,7,4'-Trihydroxy-8-methoxyflavone | Trypsin-1 |
| Scutellariae Radix | 5,7,4'-Trihydroxy-8-methoxyflavone | Nuclear receptor coactivator 2 |
| Scutellariae Radix | 5,7,4'-Trihydroxy-8-methoxyflavone | Calmodulin |
| Scutellariae Radix | 5,7,4'-Trihydroxy-8-methoxyflavone | mRNA of PKA Catalytic Subunit C-alpha |
| Scutellariae Radix | NEOBAICALEIN | Nitric oxide synthase, inducible |
| Scutellariae Radix | NEOBAICALEIN | Thrombin |
| Scutellariae Radix | NEOBAICALEIN | Potassium voltage-gated channel subfamily H member 2 |
| Scutellariae Radix | NEOBAICALEIN | Estrogen receptor |
| Scutellariae Radix | NEOBAICALEIN | Androgen receptor |
| Scutellariae Radix | NEOBAICALEIN | Sodium channel protein type 5 subunit alpha |
| Scutellariae Radix | NEOBAICALEIN | Peroxisome proliferator activated receptor gamma |
| Scutellariae Radix | NEOBAICALEIN | Coagulation factor Xa |
| Scutellariae Radix | NEOBAICALEIN | Prostaglandin G/H synthase 2 |
| Scutellariae Radix | NEOBAICALEIN | Coagulation factor VII |
| Scutellariae Radix | NEOBAICALEIN | mRNA of Protein-tyrosine phosphatase, non-receptor type 1 |
| Scutellariae Radix | NEOBAICALEIN | DNA topoisomerase II |
| Scutellariae Radix | NEOBAICALEIN | Estrogen receptor beta |
| Scutellariae Radix | NEOBAICALEIN | Dipeptidyl peptidase IV |
| Scutellariae Radix | NEOBAICALEIN | Glycogen phosphorylase, muscle form |
| Scutellariae Radix | NEOBAICALEIN | Glycogen synthase kinase-3 beta |
| Scutellariae Radix | NEOBAICALEIN | Heat shock protein HSP 90 |
| Scutellariae Radix | NEOBAICALEIN | Serine/threonine-protein kinase Chk1 |
| Scutellariae Radix | NEOBAICALEIN | Trypsin-1 |
| Scutellariae Radix | NEOBAICALEIN | Nuclear receptor coactivator 2 |
| Scutellariae Radix | NEOBAICALEIN | Calcium-activated potassium channel subunit alpha 1 |
| Scutellariae Radix | NEOBAICALEIN | Calmodulin |
| Scutellariae Radix | Baicalin | Coagulation factor Xa |
| Scutellariae Radix | Baicalin | DNA topoisomerase II |
| Scutellariae Radix | 5,8-Dihydroxy-6,7-dimethoxyflavone | Nitric oxide synthase, inducible |
| Scutellariae Radix | 5,8-Dihydroxy-6,7-dimethoxyflavone | Prostaglandin G/H synthase 1 |
| Scutellariae Radix | 5,8-Dihydroxy-6,7-dimethoxyflavone | Estrogen receptor |
| Scutellariae Radix | 5,8-Dihydroxy-6,7-dimethoxyflavone | Androgen receptor |
| Scutellariae Radix | 5,8-Dihydroxy-6,7-dimethoxyflavone | Sodium channel protein type 5 subunit alpha |
| Scutellariae Radix | 5,8-Dihydroxy-6,7-dimethoxyflavone | Peroxisome proliferator activated receptor gamma |
| Scutellariae Radix | 5,8-Dihydroxy-6,7-dimethoxyflavone | Prostaglandin G/H synthase 2 |
| Scutellariae Radix | 5,8-Dihydroxy-6,7-dimethoxyflavone | Coagulation factor VII |
| Scutellariae Radix | 5,8-Dihydroxy-6,7-dimethoxyflavone | Estrogen receptor beta |
| Scutellariae Radix | 5,8-Dihydroxy-6,7-dimethoxyflavone | Dipeptidyl peptidase IV |
| Scutellariae Radix | 5,8-Dihydroxy-6,7-dimethoxyflavone | Peroxisome proliferator activated receptor delta |
| Scutellariae Radix | 5,8-Dihydroxy-6,7-dimethoxyflavone | Mitogen-activated protein kinase 14 |
| Scutellariae Radix | 5,8-Dihydroxy-6,7-dimethoxyflavone | Glycogen synthase kinase-3 beta |
| Scutellariae Radix | 5,8-Dihydroxy-6,7-dimethoxyflavone | Heat shock protein HSP 90 |
| Scutellariae Radix | 5,8-Dihydroxy-6,7-dimethoxyflavone | Cell division protein kinase 2 |
| Scutellariae Radix | 5,8-Dihydroxy-6,7-dimethoxyflavone | Serine/threonine-protein kinase Chk1 |
| Scutellariae Radix | 5,8-Dihydroxy-6,7-dimethoxyflavone | mRNA of PKA Catalytic Subunit C-alpha |
| Scutellariae Radix | 5,8-Dihydroxy-6,7-dimethoxyflavone | Trypsin-1 |
| Scutellariae Radix | 5,8-Dihydroxy-6,7-dimethoxyflavone | Nuclear receptor coactivator 2 |
| Scutellariae Radix | 5,8-Dihydroxy-6,7-dimethoxyflavone | Nuclear receptor coactivator 1 |
| Scutellariae Radix | 5,8-Dihydroxy-6,7-dimethoxyflavone | Calmodulin |
| Scutellariae Radix | DIHYDROOROXYLIN | Prostaglandin G/H synthase 1 |
| Scutellariae Radix | DIHYDROOROXYLIN | Sodium channel protein type 5 subunit alpha |
| Scutellariae Radix | DIHYDROOROXYLIN | Prostaglandin G/H synthase 2 |
| Scutellariae Radix | DIHYDROOROXYLIN | Retinoic acid receptor RXR-alpha |
| Scutellariae Radix | DIHYDROOROXYLIN | CGMP-inhibited 3',5'-cyclic phosphodiesterase A |
| Scutellariae Radix | DIHYDROOROXYLIN | Alpha-1B adrenergic receptor |
| Scutellariae Radix | DIHYDROOROXYLIN | Beta-2 adrenergic receptor |
| Scutellariae Radix | DIHYDROOROXYLIN | Heat shock protein HSP 90 |
| Scutellariae Radix | DIHYDROOROXYLIN | mRNA of PKA Catalytic Subunit C-alpha |
| Scutellariae Radix | DIHYDROOROXYLIN | Calmodulin |
| Scutellariae Radix | DIHYDROOROXYLIN | Nuclear receptor coactivator 1 |
| Scutellariae Radix | Sitogluside | Progesterone receptor |
| Scutellariae Radix | Sitogluside | Prostaglandin G/H synthase 1 |
| Scutellariae Radix | Sitogluside | Muscarinic acetylcholine receptor M3 |
| Scutellariae Radix | Sitogluside | Potassium voltage-gated channel subfamily H member 2 |
| Scutellariae Radix | Sitogluside | Muscarinic acetylcholine receptor M1 |
| Scutellariae Radix | Sitogluside | Sodium channel protein type 5 subunit alpha |
| Scutellariae Radix | Sitogluside | Coagulation factor Xa |
| Scutellariae Radix | Sitogluside | Prostaglandin G/H synthase 2 |
| Scutellariae Radix | Sitogluside | 5-hydroxytryptamine receptor 3A |
| Scutellariae Radix | Sitogluside | Retinoic acid receptor RXR-alpha |
| Scutellariae Radix | Sitogluside | CGMP-inhibited 3',5'-cyclic phosphodiesterase A |
| Scutellariae Radix | Sitogluside | Alpha-1B adrenergic receptor |
| Scutellariae Radix | Sitogluside | Beta-2 adrenergic receptor |
| Scutellariae Radix | Sitogluside | Alpha-1D adrenergic receptor |
| Scutellariae Radix | Sitogluside | Heat shock protein HSP 90 |
| Scutellariae Radix | Sitogluside | Nuclear receptor coactivator 2 |
| Scutellariae Radix | Sitogluside | Calmodulin |
| Scutellariae Radix | beta-sitosterol | Progesterone receptor |
| Scutellariae Radix | beta-sitosterol | Nuclear receptor coactivator 2 |
| Scutellariae Radix | beta-sitosterol | Prostaglandin G/H synthase 1 |
| Scutellariae Radix | beta-sitosterol | Prostaglandin G/H synthase 2 |
| Scutellariae Radix | beta-sitosterol | Heat shock protein HSP 90 |
| Scutellariae Radix | beta-sitosterol | Phosphatidylinositol-4,5-bisphosphate 3-kinase catalytic subunit, gamma isoform |
| Scutellariae Radix | beta-sitosterol | Potassium voltage-gated channel subfamily H member 2 |
| Scutellariae Radix | beta-sitosterol | mRNA of PKA Catalytic Subunit C-alpha |
| Scutellariae Radix | beta-sitosterol | Dopamine D1 receptor |
| Scutellariae Radix | beta-sitosterol | Muscarinic acetylcholine receptor M3 |
| Scutellariae Radix | beta-sitosterol | Muscarinic acetylcholine receptor M1 |
| Scutellariae Radix | beta-sitosterol | Sodium channel protein type 5 subunit alpha |
| Scutellariae Radix | beta-sitosterol | Gamma-aminobutyric-acid receptor alpha-2 subunit |
| Scutellariae Radix | beta-sitosterol | Muscarinic acetylcholine receptor M4 |
| Scutellariae Radix | beta-sitosterol | CGMP-inhibited 3',5'-cyclic phosphodiesterase A |
| Scutellariae Radix | beta-sitosterol | 5-hydroxytryptamine 2A receptor |
| Scutellariae Radix | beta-sitosterol | Gamma-aminobutyric-acid receptor alpha-5 subunit |
| Scutellariae Radix | beta-sitosterol | Alpha-1A adrenergic receptor |
| Scutellariae Radix | beta-sitosterol | Gamma-aminobutyric-acid receptor alpha-3 subunit |
| Scutellariae Radix | beta-sitosterol | Muscarinic acetylcholine receptor M2 |
| Scutellariae Radix | beta-sitosterol | Alpha-1B adrenergic receptor |
| Scutellariae Radix | beta-sitosterol | Beta-2 adrenergic receptor |
| Scutellariae Radix | beta-sitosterol | Neuronal acetylcholine receptor subunit alpha-2 |
| Scutellariae Radix | beta-sitosterol | Sodium-dependent serotonin transporter |
| Scutellariae Radix | beta-sitosterol | Mu-type opioid receptor |
| Scutellariae Radix | beta-sitosterol | Gamma-aminobutyric acid receptor subunit alpha-1 |
| Scutellariae Radix | beta-sitosterol | Neuronal acetylcholine receptor protein, alpha-7 chain |
| Scutellariae Radix | beta-sitosterol | Cytochrome P450-cam |
| Scutellariae Radix | beta-sitosterol | Apoptosis regulator Bcl-2 |
| Scutellariae Radix | beta-sitosterol | Apoptosis regulator BAX |
| Scutellariae Radix | beta-sitosterol | Caspase-9 |
| Scutellariae Radix | beta-sitosterol | Transcription factor AP-1 |
| Scutellariae Radix | beta-sitosterol | Caspase-3 |
| Scutellariae Radix | beta-sitosterol | Caspase-8 |
| Scutellariae Radix | beta-sitosterol | Protein kinase C alpha type |
| Scutellariae Radix | beta-sitosterol | Transforming growth factor beta-1 |
| Scutellariae Radix | beta-sitosterol | Serum paraoxonase/arylesterase 1 |
| Scutellariae Radix | beta-sitosterol | Microtubule-associated protein 2 |
| Scutellariae Radix | sitosterol | Progesterone receptor |
| Scutellariae Radix | sitosterol | Nuclear receptor coactivator 2 |
| Scutellariae Radix | sitosterol | Mineralocorticoid receptor |
| Scutellariae Radix | (+)-Syringaresinol | Potassium voltage-gated channel subfamily H member 2 |
| Scutellariae Radix | (+)-Syringaresinol | Sodium channel protein type 5 subunit alpha |
| Scutellariae Radix | (+)-Syringaresinol | Coagulation factor Xa |
| Scutellariae Radix | (+)-Syringaresinol | Prostaglandin G/H synthase 2 |
| Scutellariae Radix | (+)-Syringaresinol | DNA topoisomerase II |
| Scutellariae Radix | (+)-Syringaresinol | Nuclear receptor coactivator 2 |
| Scutellariae Radix | (+)-Syringaresinol | Calmodulin |
| Scutellariae Radix | (+)-Syringaresinol | Heat shock protein HSP 90 |
| Scutellariae Radix | campesterol | Progesterone receptor |
| Scutellariae Radix | Norwogonin | Nitric oxide synthase, inducible |
| Scutellariae Radix | Norwogonin | Prostaglandin G/H synthase 1 |
| Scutellariae Radix | Norwogonin | Androgen receptor |
| Scutellariae Radix | Norwogonin | Peroxisome proliferator activated receptor gamma |
| Scutellariae Radix | Norwogonin | Prostaglandin G/H synthase 2 |
| Scutellariae Radix | Norwogonin | CGMP-inhibited 3',5'-cyclic phosphodiesterase A |
| Scutellariae Radix | Norwogonin | Dipeptidyl peptidase IV |
| Scutellariae Radix | Norwogonin | Heat shock protein HSP 90 |
| Scutellariae Radix | Norwogonin | Cell division protein kinase 2 |
| Scutellariae Radix | Norwogonin | Phosphatidylinositol-4,5-bisphosphate 3-kinase catalytic subunit, gamma isoform |
| Scutellariae Radix | Norwogonin | Serine/threonine-protein kinase Chk1 |
| Scutellariae Radix | Norwogonin | mRNA of PKA Catalytic Subunit C-alpha |
| Scutellariae Radix | 5,2'-Dihydroxy-6,7,8-trimethoxyflavone | Nitric oxide synthase, inducible |
| Scutellariae Radix | 5,2'-Dihydroxy-6,7,8-trimethoxyflavone | Prostaglandin G/H synthase 1 |
| Scutellariae Radix | 5,2'-Dihydroxy-6,7,8-trimethoxyflavone | Thrombin |
| Scutellariae Radix | 5,2'-Dihydroxy-6,7,8-trimethoxyflavone | Potassium voltage-gated channel subfamily H member 2 |
| Scutellariae Radix | 5,2'-Dihydroxy-6,7,8-trimethoxyflavone | Androgen receptor |
| Scutellariae Radix | 5,2'-Dihydroxy-6,7,8-trimethoxyflavone | Sodium channel protein type 5 subunit alpha |
| Scutellariae Radix | 5,2'-Dihydroxy-6,7,8-trimethoxyflavone | Coagulation factor Xa |
| Scutellariae Radix | 5,2'-Dihydroxy-6,7,8-trimethoxyflavone | Prostaglandin G/H synthase 2 |
| Scutellariae Radix | 5,2'-Dihydroxy-6,7,8-trimethoxyflavone | Nitric-oxide synthase, endothelial |
| Scutellariae Radix | 5,2'-Dihydroxy-6,7,8-trimethoxyflavone | Coagulation factor VII |
| Scutellariae Radix | 5,2'-Dihydroxy-6,7,8-trimethoxyflavone | DNA topoisomerase II |
| Scutellariae Radix | 5,2'-Dihydroxy-6,7,8-trimethoxyflavone | Estrogen receptor beta |
| Scutellariae Radix | 5,2'-Dihydroxy-6,7,8-trimethoxyflavone | Dipeptidyl peptidase IV |
| Scutellariae Radix | 5,2'-Dihydroxy-6,7,8-trimethoxyflavone | Peroxisome proliferator activated receptor delta |
| Scutellariae Radix | 5,2'-Dihydroxy-6,7,8-trimethoxyflavone | Heat shock protein HSP 90 |
| Scutellariae Radix | 5,2'-Dihydroxy-6,7,8-trimethoxyflavone | Trypsin-1 |
| Scutellariae Radix | 5,2'-Dihydroxy-6,7,8-trimethoxyflavone | Nuclear receptor coactivator 2 |
| Scutellariae Radix | 5,2'-Dihydroxy-6,7,8-trimethoxyflavone | Calmodulin |
| Scutellariae Radix | 5,2'-Dihydroxy-6,7,8-trimethoxyflavone | Vascular endothelial growth factor receptor 2 |
| Scutellariae Radix | 5,2'-Dihydroxy-6,7,8-trimethoxyflavone | Nuclear receptor coactivator 1 |
| Scutellariae Radix | 5,2'-Dihydroxy-6,7,8-trimethoxyflavone | Calcium-activated potassium channel subunit alpha 1 |
| Scutellariae Radix | (-)-alpha-cedrene | Muscarinic acetylcholine receptor M3 |
| Scutellariae Radix | (-)-alpha-cedrene | Prostaglandin G/H synthase 2 |
| Scutellariae Radix | (-)-alpha-cedrene | Gamma-aminobutyric-acid receptor alpha-2 subunit |
| Scutellariae Radix | (-)-alpha-cedrene | Retinoic acid receptor RXR-alpha |
| Scutellariae Radix | (-)-alpha-cedrene | Gamma-aminobutyric acid receptor subunit alpha-1 |
| Scutellariae Radix | (-)-alpha-cedrene | Nuclear receptor coactivator 2 |
| Scutellariae Radix | (-)-alpha-cedrene | Muscarinic acetylcholine receptor M1 |
| Scutellariae Radix | (-)-alpha-cedrene | Alcohol dehydrogenase 1B |
| Scutellariae Radix | (-)-alpha-cedrene | Alcohol dehydrogenase 1C |
| Scutellariae Radix | Cosmetin | Prostaglandin G/H synthase 2 |
| Scutellariae Radix | Cosmetin | Calmodulin |
| Scutellariae Radix | (S)-Matsutake alcohol | Alcohol dehydrogenase 1C |
| Scutellariae Radix | (S)-Matsutake alcohol | Trypsin-3 |
| Scutellariae Radix | l-Menthone | Gamma-aminobutyric-acid receptor alpha-2 subunit |
| Scutellariae Radix | l-Menthone | Gamma-aminobutyric-acid receptor alpha-5 subunit |
| Scutellariae Radix | l-Menthone | Muscarinic acetylcholine receptor M2 |
| Scutellariae Radix | l-Menthone | Gamma-aminobutyric acid receptor subunit alpha-1 |
| Scutellariae Radix | l-Menthone | Cytochrome P450-cam |
| Scutellariae Radix | l-Menthone | Ig gamma-1 chain C region |
| Scutellariae Radix | l-Menthone | Cholinesterase |
| Scutellariae Radix | l-Menthone | Muscarinic acetylcholine receptor M3 |
| Scutellariae Radix | l-Menthone | Muscarinic acetylcholine receptor M1 |
| Scutellariae Radix | l-Menthone | Gamma-aminobutyric-acid receptor alpha-3 subunit |
| Scutellariae Radix | l-Menthone | Alpha-1B adrenergic receptor |
| Scutellariae Radix | l-Menthone | Neuronal acetylcholine receptor subunit alpha-2 |
| Scutellariae Radix | apigenin | Prostaglandin G/H synthase 1 |
| Scutellariae Radix | apigenin | Androgen receptor |
| Scutellariae Radix | apigenin | Prostaglandin G/H synthase 2 |
| Scutellariae Radix | apigenin | Heat shock protein HSP 90 |
| Scutellariae Radix | apigenin | Trypsin-1 |
| Scutellariae Radix | apigenin | Nuclear receptor coactivator 2 |
| Scutellariae Radix | apigenin | Phosphatidylinositol-4,5-bisphosphate 3-kinase catalytic subunit, gamma isoform |
| Scutellariae Radix | apigenin | mRNA of PKA Catalytic Subunit C-alpha |
| Scutellariae Radix | apigenin | Sodium channel protein type 5 subunit alpha |
| Scutellariae Radix | apigenin | Coagulation factor Xa |
| Scutellariae Radix | apigenin | Coagulation factor VII |
| Scutellariae Radix | apigenin | DNA topoisomerase II |
| Scutellariae Radix | apigenin | Dipeptidyl peptidase IV |
| Scutellariae Radix | apigenin | Calmodulin |
| Scutellariae Radix | apigenin | Transcription factor p65 |
| Scutellariae Radix | apigenin | RAC-alpha serine/threonine-protein kinase |
| Scutellariae Radix | apigenin | Vascular endothelial growth factor A |
| Scutellariae Radix | apigenin | G1/S-specific cyclin-D1 |
| Scutellariae Radix | apigenin | Apoptosis regulator Bcl-2 |
| Scutellariae Radix | apigenin | Bcl-2-like protein 1 |
| Scutellariae Radix | apigenin | Proto-oncogene c-Fos |
| Scutellariae Radix | apigenin | Cyclin-dependent kinase inhibitor 1 |
| Scutellariae Radix | apigenin | Eukaryotic translation initiation factor 6 |
| Scutellariae Radix | apigenin | Apoptosis regulator BAX |
| Scutellariae Radix | apigenin | Caspase-9 |
| Scutellariae Radix | apigenin | Urokinase-type plasminogen activator |
| Scutellariae Radix | apigenin | Matrix metalloproteinase-9 |
| Scutellariae Radix | apigenin | Retinoblastoma-associated protein |
| Scutellariae Radix | apigenin | Cell division protein kinase 4 |
| Scutellariae Radix | apigenin | Tumor necrosis factor |
| Scutellariae Radix | apigenin | Transcription factor AP-1 |
| Scutellariae Radix | apigenin | Cell division protein kinase 6 |
| Scutellariae Radix | apigenin | Cyclin-dependent kinase inhibitor 2A, isoforms 1/2/3 |
| Scutellariae Radix | apigenin | Eukaryotic translation elongation factor 1 epsilon-1 |
| Scutellariae Radix | apigenin | Activator of 90 kDa heat shock protein ATPase homolog 1 |
| Scutellariae Radix | apigenin | Caspase-3 |
| Scutellariae Radix | apigenin | Cellular tumor antigen p53 |
| Scutellariae Radix | apigenin | NF-kappa-B inhibitor alpha |
| Scutellariae Radix | apigenin | Ornithine decarboxylase |
| Scutellariae Radix | apigenin | E3 ubiquitin-protein ligase Mdm2 |
| Scutellariae Radix | apigenin | Bcl2 antagonist of cell death |
| Scutellariae Radix | apigenin | Interstitial collagenase |
| Scutellariae Radix | apigenin | Hypoxia-inducible factor 1-alpha |
| Scutellariae Radix | apigenin | Insulin-like growth factor 1 receptor |
| Scutellariae Radix | apigenin | Protein CBFA2T1 |
| Scutellariae Radix | apigenin | Probable E3 ubiquitin-protein ligase HERC5 |
| Scutellariae Radix | apigenin | Cell division control protein 2 homolog |
| Scutellariae Radix | apigenin | Acetyl-CoA carboxylase 1 |
| Scutellariae Radix | apigenin | Heme oxygenase 1 |
| Scutellariae Radix | apigenin | Intercellular adhesion molecule 1 |
| Scutellariae Radix | apigenin | Induced myeloid leukemia cell differentiation protein Mcl-1 |
| Scutellariae Radix | apigenin | G1/S-specific cyclin-D2 |
| Scutellariae Radix | apigenin | Interleukin-2 |
| Scutellariae Radix | apigenin | G2/mitotic-specific cyclin-B1 |
| Scutellariae Radix | apigenin | Plasminogen activator inhibitor 1 |
| Scutellariae Radix | apigenin | Interferon gamma |
| Scutellariae Radix | apigenin | Interleukin-4 |
| Scutellariae Radix | apigenin | NF-kappa-B essential modulator |
| Scutellariae Radix | apigenin | Cytochrome P450 19A1 |
| Scutellariae Radix | apigenin | Baculoviral IAP repeat-containing protein 4 |
| Scutellariae Radix | apigenin | 26S proteasome non-ATPase regulatory subunit 3 |
| Scutellariae Radix | apigenin | Solute carrier family 2, facilitated glucose transporter member 4 |
| Scutellariae Radix | apigenin | Insulin receptor |
| Scutellariae Radix | apigenin | CD40 ligand |
| Scutellariae Radix | apigenin | Cytochrome c |
| Scutellariae Radix | apigenin | CASP8 and FADD-like apoptosis regulator |
| Scutellariae Radix | apigenin | Alpha- and gamma-adaptin-binding protein p34 |
| Scutellariae Radix | apigenin | Insulin |
| Scutellariae Radix | apigenin | Low affinity immunoglobulin epsilon Fc receptor |
| Scutellariae Radix | apigenin | Interleukin-13 |
| Scutellariae Radix | apigenin | High affinity immunoglobulin epsilon receptor subunit beta |
| Scutellariae Radix | apigenin | Intestinal-type alkaline phosphatase |
| Scutellariae Radix | apigenin | Proteasome activator complex subunit 3 |
| Scutellariae Radix | apigenin | Glucose-6-phosphatase |
| Scutellariae Radix | apigenin | Adenomatous polyposis coli protein |
| Scutellariae Radix | apigenin | Transient receptor potential cation channel subfamily M member 2 |
| Scutellariae Radix | apigenin | Aldo-keto reductase family 1 member C3 |
| Scutellariae Radix | apigenin | Sodium/iodide cotransporter |
| Scutellariae Radix | apigenin | Sodium/potassium-transporting ATPase subunit gamma |
| Scutellariae Radix | apigenin | Dolichyl-phosphate beta-glucosyltransferase |
| Scutellariae Radix | Germacrene D | Prostaglandin G/H synthase 1 |
| Scutellariae Radix | Germacrene D | Prostaglandin G/H synthase 2 |
| Scutellariae Radix | Germacrene D | Sodium-dependent noradrenaline transporter |
| Scutellariae Radix | Germacrene D | Nuclear receptor coactivator 2 |
| Scutellariae Radix | beta-Selinene | Prostaglandin G/H synthase 1 |
| Scutellariae Radix | beta-Selinene | Muscarinic acetylcholine receptor M3 |
| Scutellariae Radix | beta-Selinene | Muscarinic acetylcholine receptor M1 |
| Scutellariae Radix | beta-Selinene | Prostaglandin G/H synthase 2 |
| Scutellariae Radix | beta-Selinene | Gamma-aminobutyric-acid receptor alpha-2 subunit |
| Scutellariae Radix | beta-Selinene | Retinoic acid receptor RXR-alpha |
| Scutellariae Radix | beta-Selinene | Sodium-dependent noradrenaline transporter |
| Scutellariae Radix | beta-Selinene | Gamma-aminobutyric-acid receptor alpha-3 subunit |
| Scutellariae Radix | beta-Selinene | Muscarinic acetylcholine receptor M2 |
| Scutellariae Radix | beta-Selinene | Alpha-1B adrenergic receptor |
| Scutellariae Radix | beta-Selinene | Gamma-aminobutyric acid receptor subunit alpha-1 |
| Scutellariae Radix | beta-Selinene | Nuclear receptor coactivator 2 |
| Scutellariae Radix | beta-Selinene | Gamma-aminobutyric-acid receptor subunit alpha-6 |
| Scutellariae Radix | palmitic acid | Cathepsin D |
| Scutellariae Radix | palmitic acid | Alcohol dehydrogenase 1B |
| Scutellariae Radix | palmitic acid | Alcohol dehydrogenase 1C |
| Scutellariae Radix | palmitic acid | Prostaglandin G/H synthase 1 |
| Scutellariae Radix | palmitic acid | Prostaglandin G/H synthase 2 |
| Scutellariae Radix | palmitic acid | Rhodopsin |
| Scutellariae Radix | palmitic acid | Ig gamma-1 chain C region |
| Scutellariae Radix | palmitic acid | Ferrichrome-iron receptor |
| Scutellariae Radix | palmitic acid | Nuclear receptor coactivator 2 |
| Scutellariae Radix | palmitic acid | Apoptosis regulator Bcl-2 |
| Scutellariae Radix | palmitic acid | Interleukin-10 |
| Scutellariae Radix | palmitic acid | Tumor necrosis factor |
| Scutellariae Radix | palmitic acid | Collagen alpha-1(I) chain |
| Scutellariae Radix | palmitic acid | Phosphatidylinositol-3,4,5-trisphosphate 3-phosphatase and dual-specificity protein phosphatase PTEN |
| Scutellariae Radix | palmitic acid | Putative beta-glucuronidase-like protein SMA3 |
| Scutellariae Radix | palmitic acid | Solute carrier family 22 member 5 |
| Scutellariae Radix | palmitic acid | Choline-phosphate cytidylyltransferase A |
| Scutellariae Radix | ent-Epicatechin | Prostaglandin G/H synthase 1 |
| Scutellariae Radix | ent-Epicatechin | Estrogen receptor |
| Scutellariae Radix | ent-Epicatechin | Prostaglandin G/H synthase 2 |
| Scutellariae Radix | ent-Epicatechin | Heat shock protein HSP 90 |
| Scutellariae Radix | ent-Epicatechin | Beta-lactamase |
| Scutellariae Radix | ent-Epicatechin | mRNA of PKA Catalytic Subunit C-alpha |
| Scutellariae Radix | EIC | Prostaglandin G/H synthase 1 |
| Scutellariae Radix | EIC | Prostaglandin G/H synthase 2 |
| Scutellariae Radix | EIC | Retinoic acid receptor RXR-alpha |
| Scutellariae Radix | EIC | Nuclear receptor coactivator 2 |
| Scutellariae Radix | EIC | Lysozyme |
| Scutellariae Radix | EIC | Nicotinate-nucleotide--dimethylbenzimidazole phosphoribosyltransferase |
| Scutellariae Radix | EIC | Sodium-dependent noradrenaline transporter |
| Scutellariae Radix | EIC | Ig gamma-1 chain C region |
| Scutellariae Radix | EIC | Gamma-aminobutyric-acid receptor alpha-2 subunit |
| Scutellariae Radix | EIC | Gamma-aminobutyric acid receptor subunit alpha-1 |
| Scutellariae Radix | EIC | Cytochrome P450-cam |
| Scutellariae Radix | EIC | Transient receptor potential cation channel subfamily V member 1 |
| Scutellariae Radix | EIC | Muscarinic acetylcholine receptor M1 |
| Scutellariae Radix | EIC | Muscarinic acetylcholine receptor M2 |
| Scutellariae Radix | EIC | Gamma-aminobutyric-acid receptor subunit alpha-6 |
| Scutellariae Radix | caprylic acid | Phospholipase A2 |
| Scutellariae Radix | caprylic acid | Alcohol dehydrogenase 1B |
| Scutellariae Radix | caprylic acid | Alcohol dehydrogenase 1C |
| Scutellariae Radix | caprylic acid | Acyl-CoA thioesterase I precursor |
| Scutellariae Radix | caprylic acid | Trypsin-3 |
| Scutellariae Radix | caprylic acid | Interleukin-8 |
| Scutellariae Radix | caprylic acid | Peroxisome proliferator-activated receptor alpha |
| Scutellariae Radix | caprylic acid | Histone acetyltransferase p300 |
| Scutellariae Radix | caprylic acid | Fatty acid-binding protein, liver |
| Scutellariae Radix | caprylic acid | Retinol-binding protein 2 |
| Scutellariae Radix | Stigmasterol | Progesterone receptor |
| Scutellariae Radix | Stigmasterol | Mineralocorticoid receptor |
| Scutellariae Radix | Stigmasterol | Nuclear receptor coactivator 2 |
| Scutellariae Radix | Stigmasterol | Alcohol dehydrogenase 1C |
| Scutellariae Radix | Stigmasterol | Ig gamma-1 chain C region |
| Scutellariae Radix | Stigmasterol | Retinoic acid receptor RXR-alpha |
| Scutellariae Radix | Stigmasterol | Nuclear receptor coactivator 1 |
| Scutellariae Radix | Stigmasterol | Prostaglandin G/H synthase 1 |
| Scutellariae Radix | Stigmasterol | Prostaglandin G/H synthase 2 |
| Scutellariae Radix | Stigmasterol | Alpha-2A adrenergic receptor |
| Scutellariae Radix | Stigmasterol | Sodium-dependent noradrenaline transporter |
| Scutellariae Radix | Stigmasterol | Sodium-dependent dopamine transporter |
| Scutellariae Radix | Stigmasterol | Beta-2 adrenergic receptor |
| Scutellariae Radix | Stigmasterol | Aldose reductase |
| Scutellariae Radix | Stigmasterol | Urokinase-type plasminogen activator |
| Scutellariae Radix | Stigmasterol | Leukotriene A-4 hydrolase |
| Scutellariae Radix | Stigmasterol | Amine oxidase [flavin-containing] B |
| Scutellariae Radix | Stigmasterol | Amine oxidase [flavin-containing] A |
| Scutellariae Radix | Stigmasterol | mRNA of PKA Catalytic Subunit C-alpha |
| Scutellariae Radix | Stigmasterol | Chymotrypsinogen B |
| Scutellariae Radix | Stigmasterol | Muscarinic acetylcholine receptor M3 |
| Scutellariae Radix | Stigmasterol | Muscarinic acetylcholine receptor M1 |
| Scutellariae Radix | Stigmasterol | Beta-1 adrenergic receptor |
| Scutellariae Radix | Stigmasterol | Sodium channel protein type 5 subunit alpha |
| Scutellariae Radix | Stigmasterol | 5-hydroxytryptamine 2A receptor |
| Scutellariae Radix | Stigmasterol | Alpha-1A adrenergic receptor |
| Scutellariae Radix | Stigmasterol | Gamma-aminobutyric-acid receptor alpha-3 subunit |
| Scutellariae Radix | Stigmasterol | Muscarinic acetylcholine receptor M2 |
| Scutellariae Radix | Stigmasterol | Alpha-1B adrenergic receptor |
| Scutellariae Radix | Stigmasterol | Gamma-aminobutyric acid receptor subunit alpha-1 |
| Scutellariae Radix | Stigmasterol | Neuronal acetylcholine receptor protein, alpha-7 chain |
| Scutellariae Radix | (S)-camphor | Cytochrome P450-cam |
| Scutellariae Radix | (S)-camphor | Gamma-aminobutyric-acid receptor alpha-2 subunit |
| Scutellariae Radix | (S)-camphor | Gamma-aminobutyric acid receptor subunit alpha-1 |
| Scutellariae Radix | DBP | Muscarinic acetylcholine receptor M3 |
| Scutellariae Radix | DBP | Muscarinic acetylcholine receptor M1 |
| Scutellariae Radix | DBP | Sodium-dependent noradrenaline transporter |
| Scutellariae Radix | DBP | Sodium-dependent dopamine transporter |
| Scutellariae Radix | DBP | Beta-2 adrenergic receptor |
| Scutellariae Radix | DBP | Sodium-dependent serotonin transporter |
| Scutellariae Radix | DBP | Prostaglandin G/H synthase 1 |
| Scutellariae Radix | DBP | Prostaglandin G/H synthase 2 |
| Scutellariae Radix | DBP | Nitric-oxide synthase, endothelial |
| Scutellariae Radix | DBP | Retinoic acid receptor RXR-alpha |
| Scutellariae Radix | DBP | Glycogen synthase kinase-3 beta |
| Scutellariae Radix | DBP | Heat shock protein HSP 90 |
| Scutellariae Radix | DBP | Beta-lactamase |
| Scutellariae Radix | DBP | mRNA of PKA Catalytic Subunit C-alpha |
| Scutellariae Radix | DBP | CGMP-inhibited 3',5'-cyclic phosphodiesterase A |
| Scutellariae Radix | DBP | Alpha-1A adrenergic receptor |
| Scutellariae Radix | DBP | Muscarinic acetylcholine receptor M2 |
| Scutellariae Radix | DBP | Ig gamma-1 chain C region |
| Scutellariae Radix | DBP | 5-hydroxytryptamine 2A receptor |
| Scutellariae Radix | DBP | Gamma-aminobutyric acid receptor subunit alpha-1 |
| Scutellariae Radix | DBP | Neuronal acetylcholine receptor protein, alpha-7 chain |
| Scutellariae Radix | DBP | Gamma-aminobutyric-acid receptor subunit alpha-6 |
| Scutellariae Radix | WLN: VHR | Alcohol dehydrogenase 1B |
| Scutellariae Radix | WLN: VHR | Alcohol dehydrogenase 1C |
| Scutellariae Radix | WLN: VHR | Alcohol dehydrogenase 1A |
| Scutellariae Radix | WLN: VHR | Lysozyme |
| Scutellariae Radix | WLN: VHR | Nicotinate-nucleotide--dimethylbenzimidazole phosphoribosyltransferase |
| Scutellariae Radix | WLN: VHR | Trypsin-3 |
| Scutellariae Radix | Hyacinthin | Alcohol dehydrogenase 1B |
| Scutellariae Radix | Hyacinthin | Alcohol dehydrogenase 1C |
| Scutellariae Radix | Hyacinthin | Alcohol dehydrogenase 1A |
| Scutellariae Radix | Hyacinthin | Nicotinate-nucleotide--dimethylbenzimidazole phosphoribosyltransferase |
| Scutellariae Radix | Hyacinthin | Trypsin-3 |
| Scutellariae Radix | Hyacinthin | Bacillolysin |
| Scutellariae Radix | d-isomenthone | Gamma-aminobutyric-acid receptor alpha-2 subunit |
| Scutellariae Radix | d-isomenthone | Muscarinic acetylcholine receptor M2 |
| Scutellariae Radix | d-isomenthone | Gamma-aminobutyric acid receptor subunit alpha-1 |
| Scutellariae Radix | d-isomenthone | Cholinesterase |
| Scutellariae Radix | d-isomenthone | Cytochrome P450-cam |
| Scutellariae Radix | d-isomenthone | Ig gamma-1 chain C region |
| Scutellariae Radix | p-coumaric acid | Prostaglandin G/H synthase 1 |
| Scutellariae Radix | p-coumaric acid | Prostaglandin G/H synthase 2 |
| Scutellariae Radix | p-coumaric acid | Amine oxidase [flavin-containing] B |
| Scutellariae Radix | p-coumaric acid | Amine oxidase [flavin-containing] A |
| Scutellariae Radix | p-coumaric acid | Lysozyme |
| Scutellariae Radix | p-coumaric acid | Chymotrypsinogen B |
| Scutellariae Radix | p-coumaric acid | Group IIE secretory phospholipase A2 |
| Scutellariae Radix | p-coumaric acid | Thermolysin |
| Scutellariae Radix | p-coumaric acid | Bacillolysin |
| Scutellariae Radix | p-coumaric acid | Nitric oxide synthase, endothelial |
| Scutellariae Radix | p-coumaric acid | Tyrosinase |
| Scutellariae Radix | p-coumaric acid | Polyphenol oxidase I, chloroplastic |
| Scutellariae Radix | p-coumaric acid | C-C motif chemokine 16 |
| Scutellariae Radix | jatrorrizine | Nitric oxide synthase, inducible |
| Scutellariae Radix | jatrorrizine | Prostaglandin G/H synthase 1 |
| Scutellariae Radix | jatrorrizine | Potassium voltage-gated channel subfamily H member 2 |
| Scutellariae Radix | jatrorrizine | Estrogen receptor |
| Scutellariae Radix | jatrorrizine | Androgen receptor |
| Scutellariae Radix | jatrorrizine | Sodium channel protein type 5 subunit alpha |
| Scutellariae Radix | jatrorrizine | Prostaglandin G/H synthase 2 |
| Scutellariae Radix | jatrorrizine | Nitric-oxide synthase, endothelial |
| Scutellariae Radix | jatrorrizine | Retinoic acid receptor RXR-alpha |
| Scutellariae Radix | jatrorrizine | Estrogen receptor beta |
| Scutellariae Radix | jatrorrizine | Heat shock protein HSP 90 |
| Scutellariae Radix | jatrorrizine | Cell division protein kinase 2 |
| Scutellariae Radix | jatrorrizine | Trypsin-1 |
| Scutellariae Radix | jatrorrizine | Proto-oncogene serine/threonine-protein kinase Pim-1 |
| Scutellariae Radix | jatrorrizine | Cyclin-A2 |
| Scutellariae Radix | jatrorrizine | Nuclear receptor coactivator 2 |
| Scutellariae Radix | jatrorrizine | Calmodulin |
| Scutellariae Radix | jatrorrizine | Calcium-activated potassium channel subunit alpha 1 |
| Scutellariae Radix | jatrorrizine | DNA topoisomerase II |
| Scutellariae Radix | jatrorrizine | mRNA of PKA Catalytic Subunit C-alpha |
| Scutellariae Radix | stearic acid | Prostaglandin G/H synthase 1 |
| Scutellariae Radix | stearic acid | Prostaglandin G/H synthase 2 |
| Scutellariae Radix | stearic acid | Retinoic acid receptor RXR-alpha |
| Scutellariae Radix | stearic acid | Nuclear receptor coactivator 2 |
| Scutellariae Radix | stearic acid | Ig gamma-1 chain C region |
| Scutellariae Radix | stearic acid | Transcription factor Sp1 |
| Scutellariae Radix | stearic acid | Ectonucleotide pyrophosphatase/phosphodiesterase family member 7 |
| Scutellariae Radix | HEXATRIACONTANE | Prostaglandin G/H synthase 1 |
| Scutellariae Radix | HEXATRIACONTANE | Prostaglandin G/H synthase 2 |
| Scutellariae Radix | HEXATRIACONTANE | Heat shock protein HSP 90 |
| Scutellariae Radix | HEXATRIACONTANE | Phosphatidylinositol-4,5-bisphosphate 3-kinase catalytic subunit, gamma isoform |
| Scutellariae Radix | HEXATRIACONTANE | mRNA of PKA Catalytic Subunit C-alpha |
| Scutellariae Radix | HEXATRIACONTANE | Nuclear receptor coactivator 2 |
| Scutellariae Radix | methyl palmitate | Prostaglandin G/H synthase 1 |
| Scutellariae Radix | methyl palmitate | Nuclear receptor coactivator 2 |
| Scutellariae Radix | methyl palmitate | Ig gamma-1 chain C region |
| Scutellariae Radix | methyl palmitate | Transcription factor p65 |
| Scutellariae Radix | methyl palmitate | Interleukin-10 |
| Scutellariae Radix | methyl palmitate | Tumor necrosis factor |
| Scutellariae Radix | methyl palmitate | Interleukin-6 |
| Scutellariae Radix | methyl palmitate | Prostaglandin G/H synthase 2 |
| Scutellariae Radix | methyl palmitate | Prostaglandin E2 receptor EP3 subtype |
| Scutellariae Radix | longipinene | Muscarinic acetylcholine receptor M3 |
| Scutellariae Radix | longipinene | Prostaglandin G/H synthase 2 |
| Scutellariae Radix | longipinene | Gamma-aminobutyric acid receptor subunit alpha-1 |
| Scutellariae Radix | Methyl laurate | Ig gamma-1 chain C region |
| Scutellariae Radix | Methyl laurate | Nuclear receptor coactivator 2 |
| Scutellariae Radix | myristic acid | Prostaglandin G/H synthase 1 |
| Scutellariae Radix | myristic acid | Prostaglandin G/H synthase 2 |
| Scutellariae Radix | myristic acid | Cholinesterase |
| Scutellariae Radix | myristic acid | Phospholipase A2 |
| Scutellariae Radix | myristic acid | Rhinovirus coat protein |
| Scutellariae Radix | myristic acid | Ig gamma-1 chain C region |
| Scutellariae Radix | myristic acid | Ferrichrome-iron receptor |
| Scutellariae Radix | myristic acid | 3-oxoacyl-[acyl-carrier-protein] synthase 1 |
| Scutellariae Radix | myristic acid | Nuclear receptor coactivator 2 |
| Scutellariae Radix | myristic acid | Nuclear receptor coactivator 1 |
| Scutellariae Radix | myristic acid | Phosphatidylcholine-sterol acyltransferase |
| Scutellariae Radix | coptisine | Nitric oxide synthase, inducible |
| Scutellariae Radix | coptisine | Prostaglandin G/H synthase 1 |
| Scutellariae Radix | coptisine | Potassium voltage-gated channel subfamily H member 2 |
| Scutellariae Radix | coptisine | Estrogen receptor |
| Scutellariae Radix | coptisine | Androgen receptor |
| Scutellariae Radix | coptisine | Sodium channel protein type 5 subunit alpha |
| Scutellariae Radix | coptisine | Prostaglandin G/H synthase 2 |
| Scutellariae Radix | coptisine | Nitric-oxide synthase, endothelial |
| Scutellariae Radix | coptisine | Trypsin-1 |
| Scutellariae Radix | bis[(2S)-2-ethylhexyl] benzene-1,2-dicarboxylate | Sodium channel protein type 5 subunit alpha |
| Scutellariae Radix | Hypnon | Alcohol dehydrogenase 1B |
| Scutellariae Radix | Hypnon | Alcohol dehydrogenase 1C |
| Scutellariae Radix | Hypnon | Alcohol dehydrogenase 1A |
| Scutellariae Radix | Hypnon | Lysozyme |
| Scutellariae Radix | Hypnon | Bacillolysin |
| Scutellariae Radix | Hypnon | Nicotinate-nucleotide--dimethylbenzimidazole phosphoribosyltransferase |
| Scutellariae Radix | Hypnon | Trypsin-3 |
| Scutellariae Radix | Methyl palmitelaidate | Prostaglandin G/H synthase 1 |
| Scutellariae Radix | Methyl palmitelaidate | Retinoic acid receptor RXR-alpha |
| Scutellariae Radix | Methyl palmitelaidate | Nuclear receptor coactivator 2 |
| Scutellariae Radix | Methyl palmitelaidate | Prostaglandin G/H synthase 2 |
| Scutellariae Radix | Methyl linolelaidate | Nuclear receptor coactivator 2 |
| Scutellariae Radix | Methyl linolelaidate | Prostaglandin G/H synthase 1 |
| Scutellariae Radix | Methyl linolelaidate | Prostaglandin G/H synthase 2 |
| Scutellariae Radix | Methyl linolelaidate | Retinoic acid receptor RXR-alpha |
| Scutellariae Radix | Pulegone | Gamma-aminobutyric-acid receptor alpha-2 subunit |
| Scutellariae Radix | Pulegone | Gamma-aminobutyric acid receptor subunit alpha-1 |
| Scutellariae Radix | Pulegone | Cytochrome P450-cam |
| Scutellariae Radix | Pulegone | Gamma-aminobutyric-acid receptor alpha-5 subunit |
| Scutellariae Radix | Pulegone | Muscarinic acetylcholine receptor M2 |
| Scutellariae Radix | Pulegone | Muscarinic acetylcholine receptor M1 |
| Scutellariae Radix | Pulegone | Sodium-dependent noradrenaline transporter |
| Scutellariae Radix | Pulegone | Gamma-aminobutyric-acid receptor subunit alpha-6 |
| Scutellariae Radix | hexanoic acid | Alcohol dehydrogenase 1C |
| Scutellariae Radix | hexanoic acid | Trypsin-3 |
| Scutellariae Radix | hexanoic acid | Bacillolysin |
| Scutellariae Radix | hexanoic acid | Inositol-3-phosphate synthase 1 |
| Scutellariae Radix | tetramethylpyrazine | Vascular endothelial growth factor A |
| Scutellariae Radix | tetramethylpyrazine | Hypoxia-inducible factor 1-alpha |
| Scutellariae Radix | tetramethylpyrazine | Thromboxane-A synthase |
| Scutellariae Radix | tetramethylpyrazine | Integrin alpha-IIb |
| Scutellariae Radix | catalpol | Dipeptidyl peptidase IV |
| Scutellariae Radix | catalpol | Apoptosis regulator Bcl-2 |
| Scutellariae Radix | catalpol | Caspase-3 |
| Scutellariae Radix | catalpol | Superoxide dismutase [Cu-Zn] |
| Scutellariae Radix | Diop | Sodium channel protein type 5 subunit alpha |
| Scutellariae Radix | Diop | Beta-2 adrenergic receptor |
| Scutellariae Radix | Diop | Muscarinic acetylcholine receptor M3 |
| Scutellariae Radix | epiberberine | Nitric oxide synthase, inducible |
| Scutellariae Radix | epiberberine | Potassium voltage-gated channel subfamily H member 2 |
| Scutellariae Radix | epiberberine | Estrogen receptor |
| Scutellariae Radix | epiberberine | Androgen receptor |
| Scutellariae Radix | epiberberine | Prostaglandin G/H synthase 2 |
| Scutellariae Radix | epiberberine | Nitric-oxide synthase, endothelial |
| Scutellariae Radix | epiberberine | Retinoic acid receptor RXR-alpha |
| Scutellariae Radix | epiberberine | mRNA of PKA Catalytic Subunit C-alpha |
| Scutellariae Radix | epiberberine | Trypsin-1 |
| Scutellariae Radix | epiberberine | Nuclear receptor coactivator 2 |
| Scutellariae Radix | epiberberine | cAMP and cAMP-inhibited cGMP 3',5'-cyclic phosphodiesterase 10A |
| Scutellariae Radix | nonanoic acid | Phospholipase A2 |
| Scutellariae Radix | nonanoic acid | Alcohol dehydrogenase 1B |
| Scutellariae Radix | nonanoic acid | Alcohol dehydrogenase 1C |
| Scutellariae Radix | nonanoic acid | Cathepsin D |
| Scutellariae Radix | nonanoic acid | Inositol-3-phosphate synthase 1 |
| Scutellariae Radix | Patchoulene | Muscarinic acetylcholine receptor M3 |
| Scutellariae Radix | Patchoulene | Muscarinic acetylcholine receptor M1 |
| Scutellariae Radix | Patchoulene | Gamma-aminobutyric-acid receptor alpha-2 subunit |
| Scutellariae Radix | Patchoulene | Muscarinic acetylcholine receptor M2 |
| Scutellariae Radix | Patchoulene | Gamma-aminobutyric acid receptor subunit alpha-1 |
| Scutellariae Radix | MEHQ | Alcohol dehydrogenase 1B |
| Scutellariae Radix | MEHQ | Alcohol dehydrogenase 1C |
| Scutellariae Radix | MEHQ | Lysozyme |
| Scutellariae Radix | MEHQ | Bacillolysin |
| Scutellariae Radix | MEHQ | Nicotinate-nucleotide--dimethylbenzimidazole phosphoribosyltransferase |
| Scutellariae Radix | Methyl octylate | Alcohol dehydrogenase 1B |
| Scutellariae Radix | Methyl octylate | Alcohol dehydrogenase 1C |
| Scutellariae Radix | Mipax | Prostaglandin G/H synthase 2 |
| Scutellariae Radix | Mipax | CGMP-inhibited 3',5'-cyclic phosphodiesterase A |
| Scutellariae Radix | Mipax | Glutamate receptor 2 |
| Scutellariae Radix | Mipax | Alpha-2A adrenergic receptor |
| Scutellariae Radix | 13-Tetradecenyl acetate | Nuclear receptor coactivator 2 |
| Scutellariae Radix | Methyl margarate | Prostaglandin G/H synthase 1 |
| Scutellariae Radix | Methyl margarate | Nuclear receptor coactivator 2 |
| Scutellariae Radix | undecanal | Olfactory receptor 1D2 |
| Scutellariae Radix | Clorius | Lysozyme |
| Scutellariae Radix | Clorius | Nicotinate-nucleotide--dimethylbenzimidazole phosphoribosyltransferase |
| Scutellariae Radix | Clorius | Alcohol dehydrogenase 1C |
| Scutellariae Radix | Azulol | Prostaglandin G/H synthase 1 |
| Scutellariae Radix | Azulol | Thrombin |
| Scutellariae Radix | Azulol | Muscarinic acetylcholine receptor M1 |
| Scutellariae Radix | Azulol | Estrogen receptor |
| Scutellariae Radix | Azulol | Prostaglandin G/H synthase 2 |
| Scutellariae Radix | Azulol | Alpha-1A adrenergic receptor |
| Scutellariae Radix | 5-o-caffeoylquinic acid | Lysosomal alpha-glucosidase |
| Scutellariae Radix | DFA | Muscarinic acetylcholine receptor M1 |
| Scutellariae Radix | DFA | Beta-1 adrenergic receptor |
| Scutellariae Radix | DFA | Amine oxidase [flavin-containing] B |
| Scutellariae Radix | DFA | Prostaglandin G/H synthase 1 |
| Scutellariae Radix | DFA | Sodium channel protein type 5 subunit alpha |
| Scutellariae Radix | DFA | Prostaglandin G/H synthase 2 |
| Scutellariae Radix | DFA | Alpha-2C adrenergic receptor |
| Scutellariae Radix | DFA | Alpha-1B adrenergic receptor |
| Scutellariae Radix | DFA | Sodium-dependent dopamine transporter |
| Scutellariae Radix | DFA | Sodium-dependent serotonin transporter |
| Scutellariae Radix | Moslosooflavone | Nitric oxide synthase, inducible |
| Scutellariae Radix | Moslosooflavone | Prostaglandin G/H synthase 1 |
| Scutellariae Radix | Moslosooflavone | Thrombin |
| Scutellariae Radix | Moslosooflavone | Androgen receptor |
| Scutellariae Radix | Moslosooflavone | Sodium channel protein type 5 subunit alpha |
| Scutellariae Radix | Moslosooflavone | Peroxisome proliferator activated receptor gamma |
| Scutellariae Radix | Moslosooflavone | Prostaglandin G/H synthase 2 |
| Scutellariae Radix | Moslosooflavone | Retinoic acid receptor RXR-alpha |
| Scutellariae Radix | Moslosooflavone | Estrogen receptor beta |
| Scutellariae Radix | Moslosooflavone | Gamma-aminobutyric acid receptor subunit alpha-1 |
| Scutellariae Radix | Moslosooflavone | Dipeptidyl peptidase IV |
| Scutellariae Radix | Moslosooflavone | Mitogen-activated protein kinase 14 |
| Scutellariae Radix | Moslosooflavone | Glycogen synthase kinase-3 beta |
| Scutellariae Radix | Moslosooflavone | Heat shock protein HSP 90 |
| Scutellariae Radix | Moslosooflavone | Cell division protein kinase 2 |
| Scutellariae Radix | Moslosooflavone | Phosphatidylinositol-4,5-bisphosphate 3-kinase catalytic subunit, gamma isoform |
| Scutellariae Radix | Moslosooflavone | Serine/threonine-protein kinase Chk1 |
| Scutellariae Radix | Moslosooflavone | mRNA of PKA Catalytic Subunit C-alpha |
| Scutellariae Radix | Moslosooflavone | Trypsin-1 |
| Scutellariae Radix | Moslosooflavone | Nuclear receptor coactivator 1 |
| Scutellariae Radix | Moslosooflavone | Calcium-activated potassium channel subunit alpha 1 |
| Scutellariae Radix | Moslosooflavone | Calmodulin |
| Scutellariae Radix | Moslosooflavone | Alpha-1B adrenergic receptor |
| Scutellariae Radix | Moslosooflavone | Beta-2 adrenergic receptor |
| Scutellariae Radix | Moslosooflavone | Neuronal acetylcholine receptor protein, alpha-7 chain |
| Scutellariae Radix | 11,13-Eicosadienoic acid, methyl ester | Nuclear receptor coactivator 2 |
| Scutellariae Radix | Methyl (Z)-cinnamate | Prostaglandin G/H synthase 1 |
| Scutellariae Radix | Methyl (Z)-cinnamate | Prostaglandin G/H synthase 2 |
| Scutellariae Radix | Methyl (Z)-cinnamate | Alpha-2A adrenergic receptor |
| Scutellariae Radix | Methyl (Z)-cinnamate | Alpha-2C adrenergic receptor |
| Scutellariae Radix | Methyl (Z)-cinnamate | Sodium-dependent noradrenaline transporter |
| Scutellariae Radix | Methyl (Z)-cinnamate | Sodium-dependent dopamine transporter |
| Scutellariae Radix | Methyl (Z)-cinnamate | Amine oxidase [flavin-containing] B |
| Scutellariae Radix | Methyl (Z)-cinnamate | Lysozyme |
| Scutellariae Radix | Methyl (Z)-cinnamate | Thermolysin |
| Scutellariae Radix | Linolenic acid methyl ester | Prostaglandin G/H synthase 1 |
| Scutellariae Radix | Linolenic acid methyl ester | Prostaglandin G/H synthase 2 |
| Scutellariae Radix | 2',3',5,7-tetrahydroxyflavone | Prostaglandin G/H synthase 1 |
| Scutellariae Radix | 2',3',5,7-tetrahydroxyflavone | Prostaglandin G/H synthase 2 |
| Scutellariae Radix | 2',3',5,7-tetrahydroxyflavone | Carbonic anhydrase II |
| Scutellariae Radix | 2',3',5,7-tetrahydroxyflavone | mRNA of Protein-tyrosine phosphatase, non-receptor type 1 |
| Scutellariae Radix | 2',3',5,7-tetrahydroxyflavone | Dipeptidyl peptidase IV |
| Scutellariae Radix | 2',3',5,7-tetrahydroxyflavone | Heat shock protein HSP 90 |
| Scutellariae Radix | 2',3',5,7-tetrahydroxyflavone | Trypsin-1 |
| Scutellariae Radix | 2',3',5,7-tetrahydroxyflavone | Nuclear receptor coactivator 2 |
| Scutellariae Radix | 5,7,4'-trihydroxy-6-methoxyflavanone | Prostaglandin G/H synthase 1 |
| Scutellariae Radix | 5,7,4'-trihydroxy-6-methoxyflavanone | Prostaglandin G/H synthase 2 |
| Scutellariae Radix | 5,7,4'-trihydroxy-6-methoxyflavanone | Carbonic anhydrase II |
| Scutellariae Radix | 5,7,4'-trihydroxy-6-methoxyflavanone | Heat shock protein HSP 90 |
| Scutellariae Radix | 5,7,4'-trihydroxy-6-methoxyflavanone | mRNA of PKA Catalytic Subunit C-alpha |
| Scutellariae Radix | 5,7,4'-trihydroxy-6-methoxyflavanone | Calmodulin |
| Scutellariae Radix | 5,7,4'-trihydroxy-8-methoxyflavanone | Prostaglandin G/H synthase 1 |
| Scutellariae Radix | 5,7,4'-trihydroxy-8-methoxyflavanone | Prostaglandin G/H synthase 2 |
| Scutellariae Radix | 5,7,4'-trihydroxy-8-methoxyflavanone | Carbonic anhydrase II |
| Scutellariae Radix | 5,7,4'-trihydroxy-8-methoxyflavanone | Heat shock protein HSP 90 |
| Scutellariae Radix | 5,7,4'-trihydroxy-8-methoxyflavanone | Phosphatidylinositol-4,5-bisphosphate 3-kinase catalytic subunit, gamma isoform |
| Scutellariae Radix | 5,7,4'-trihydroxy-8-methoxyflavanone | mRNA of PKA Catalytic Subunit C-alpha |
| Scutellariae Radix | rivularin | Nitric oxide synthase, inducible |
| Scutellariae Radix | rivularin | Prostaglandin G/H synthase 1 |
| Scutellariae Radix | rivularin | Thrombin |
| Scutellariae Radix | rivularin | Potassium voltage-gated channel subfamily H member 2 |
| Scutellariae Radix | rivularin | Androgen receptor |
| Scutellariae Radix | rivularin | Sodium channel protein type 5 subunit alpha |
| Scutellariae Radix | rivularin | Coagulation factor Xa |
| Scutellariae Radix | rivularin | Prostaglandin G/H synthase 2 |
| Scutellariae Radix | rivularin | Nitric-oxide synthase, endothelial |
| Scutellariae Radix | rivularin | Carbonic anhydrase II |
| Scutellariae Radix | rivularin | Coagulation factor VII |
| Scutellariae Radix | rivularin | Vascular endothelial growth factor receptor 2 |
| Scutellariae Radix | rivularin | Retinoic acid receptor RXR-alpha |
| Scutellariae Radix | rivularin | DNA topoisomerase II |
| Scutellariae Radix | rivularin | Estrogen receptor beta |
| Scutellariae Radix | rivularin | Dipeptidyl peptidase IV |
| Scutellariae Radix | rivularin | Heat shock protein HSP 90 |
| Scutellariae Radix | rivularin | Trypsin-1 |
| Scutellariae Radix | rivularin | Nuclear receptor coactivator 2 |
| Scutellariae Radix | rivularin | Nuclear receptor coactivator 1 |
| Scutellariae Radix | rivularin | Calcium-activated potassium channel subunit alpha 1 |
| Scutellariae Radix | rivularin | Calmodulin |
| Scutellariae Radix | Scutevulin | Nitric oxide synthase, inducible |
| Scutellariae Radix | Scutevulin | Prostaglandin G/H synthase 1 |
| Scutellariae Radix | Scutevulin | Androgen receptor |
| Scutellariae Radix | Scutevulin | Prostaglandin G/H synthase 2 |
| Scutellariae Radix | Scutevulin | Carbonic anhydrase II |
| Scutellariae Radix | Scutevulin | mRNA of Protein-tyrosine phosphatase, non-receptor type 1 |
| Scutellariae Radix | Scutevulin | Dipeptidyl peptidase IV |
| Scutellariae Radix | Scutevulin | Glycogen phosphorylase, muscle form |
| Scutellariae Radix | Scutevulin | Mitogen-activated protein kinase 14 |
| Scutellariae Radix | Scutevulin | Glycogen synthase kinase-3 beta |
| Scutellariae Radix | Scutevulin | Heat shock protein HSP 90 |
| Scutellariae Radix | Scutevulin | Cell division protein kinase 2 |
| Scutellariae Radix | Scutevulin | Phosphatidylinositol-4,5-bisphosphate 3-kinase catalytic subunit, gamma isoform |
| Scutellariae Radix | Scutevulin | Serine/threonine-protein kinase Chk1 |
| Scutellariae Radix | Scutevulin | mRNA of PKA Catalytic Subunit C-alpha |
| Scutellariae Radix | Scutevulin | Trypsin-1 |
| Scutellariae Radix | Scutevulin | Calmodulin |
| Scutellariae Radix | BZQ | Prostaglandin G/H synthase 1 |
| Scutellariae Radix | BZQ | Sodium channel protein type 5 subunit alpha |
| Scutellariae Radix | BZQ | Prostaglandin G/H synthase 2 |
| Scutellariae Radix | BZQ | Carbonic anhydrase II |
| Scutellariae Radix | BZQ | Leukotriene A-4 hydrolase |
| Scutellariae Radix | BZQ | Amine oxidase [flavin-containing] B |
| Coptidis Rhizoma | berberine | Nitric oxide synthase, inducible |
| Coptidis Rhizoma | berberine | Prostaglandin G/H synthase 1 |
| Coptidis Rhizoma | berberine | Potassium voltage-gated channel subfamily H member 2 |
| Coptidis Rhizoma | berberine | Estrogen receptor |
| Coptidis Rhizoma | berberine | Androgen receptor |
| Coptidis Rhizoma | berberine | Sodium channel protein type 5 subunit alpha |
| Coptidis Rhizoma | berberine | Coagulation factor Xa |
| Coptidis Rhizoma | berberine | Prostaglandin G/H synthase 2 |
| Coptidis Rhizoma | berberine | Nitric-oxide synthase, endothelial |
| Coptidis Rhizoma | berberine | Retinoic acid receptor RXR-alpha |
| Coptidis Rhizoma | berberine | Beta-2 adrenergic receptor |
| Coptidis Rhizoma | berberine | Heat shock protein HSP 90 |
| Coptidis Rhizoma | berberine | mRNA of PKA Catalytic Subunit C-alpha |
| Coptidis Rhizoma | berberine | Trypsin-1 |
| Coptidis Rhizoma | berberine | Nuclear receptor coactivator 2 |
| Coptidis Rhizoma | berberine | cAMP and cAMP-inhibited cGMP 3',5'-cyclic phosphodiesterase 10A |
| Coptidis Rhizoma | berberine | Calmodulin |
| Coptidis Rhizoma | columbamine | Nitric oxide synthase, inducible |
| Coptidis Rhizoma | columbamine | Prostaglandin G/H synthase 1 |
| Coptidis Rhizoma | columbamine | Potassium voltage-gated channel subfamily H member 2 |
| Coptidis Rhizoma | columbamine | Androgen receptor |
| Coptidis Rhizoma | columbamine | Sodium channel protein type 5 subunit alpha |
| Coptidis Rhizoma | columbamine | Coagulation factor Xa |
| Coptidis Rhizoma | columbamine | Prostaglandin G/H synthase 2 |
| Coptidis Rhizoma | columbamine | Nitric-oxide synthase, endothelial |
| Coptidis Rhizoma | columbamine | Coagulation factor VII |
| Coptidis Rhizoma | columbamine | Heat shock protein HSP 90 |
| Coptidis Rhizoma | columbamine | mRNA of PKA Catalytic Subunit C-alpha |
| Coptidis Rhizoma | columbamine | Trypsin-1 |
| Coptidis Rhizoma | columbamine | Nuclear receptor coactivator 2 |
| Coptidis Rhizoma | columbamine | Nuclear receptor coactivator 1 |
| Coptidis Rhizoma | columbamine | Calmodulin |
| Coptidis Rhizoma | columbamine | Retinoic acid receptor RXR-alpha |
| Coptidis Rhizoma | columbamine | Beta-2 adrenergic receptor |
| Coptidis Rhizoma | columbamine | Beta-secretase |
| Coptidis Rhizoma | columbamine | Pregnane X receptor |
| Coptidis Rhizoma | columbamine | Calcium-activated potassium channel subunit alpha 1 |
| Coptidis Rhizoma | columbamine | cAMP and cAMP-inhibited cGMP 3',5'-cyclic phosphodiesterase 10A |
| Coptidis Rhizoma | Isovanillin | Prostaglandin G/H synthase 1 |
| Coptidis Rhizoma | Isovanillin | Prostaglandin G/H synthase 2 |
| Coptidis Rhizoma | Isovanillin | Amine oxidase [flavin-containing] B |
| Coptidis Rhizoma | Isovanillin | Amine oxidase [flavin-containing] A |
| Coptidis Rhizoma | Isovanillin | Lysozyme |
| Coptidis Rhizoma | Isovanillin | Chymotrypsinogen B |
| Coptidis Rhizoma | Fagarine | Heat shock protein HSP 90 |
| Coptidis Rhizoma | magnoflorine | Prostaglandin G/H synthase 1 |
| Coptidis Rhizoma | magnoflorine | Potassium voltage-gated channel subfamily H member 2 |
| Coptidis Rhizoma | magnoflorine | Muscarinic acetylcholine receptor M1 |
| Coptidis Rhizoma | magnoflorine | Androgen receptor |
| Coptidis Rhizoma | magnoflorine | Sodium channel protein type 5 subunit alpha |
| Coptidis Rhizoma | magnoflorine | Prostaglandin G/H synthase 2 |
| Coptidis Rhizoma | magnoflorine | Carbonic anhydrase II |
| Coptidis Rhizoma | magnoflorine | Retinoic acid receptor RXR-alpha |
| Coptidis Rhizoma | magnoflorine | Acetylcholinesterase |
| Coptidis Rhizoma | magnoflorine | Alpha-1B adrenergic receptor |
| Coptidis Rhizoma | magnoflorine | Beta-2 adrenergic receptor |
| Coptidis Rhizoma | magnoflorine | Alpha-1D adrenergic receptor |
| Coptidis Rhizoma | magnoflorine | DNA topoisomerase II |
| Coptidis Rhizoma | magnoflorine | Pregnane X receptor |
| Coptidis Rhizoma | magnoflorine | Trypsin-1 |
| Coptidis Rhizoma | magnoflorine | Nuclear receptor coactivator 2 |
| Coptidis Rhizoma | magnoflorine | Nuclear receptor coactivator 1 |
| Coptidis Rhizoma | magnoflorine | Calmodulin |
| Coptidis Rhizoma | magnoflorine | Muscarinic acetylcholine receptor M3 |
| Coptidis Rhizoma | magnoflorine | Nitric-oxide synthase, endothelial |
| Coptidis Rhizoma | magnoflorine | Heat shock protein HSP 90 |
| Coptidis Rhizoma | magnoflorine | Calcium-activated potassium channel subunit alpha 1 |
| Coptidis Rhizoma | magnoflorine | Delta-type opioid receptor |
| Coptidis Rhizoma | magnoflorine | Mu-type opioid receptor |
| Coptidis Rhizoma | berberrubine | Nitric oxide synthase, inducible |
| Coptidis Rhizoma | berberrubine | Prostaglandin G/H synthase 1 |
| Coptidis Rhizoma | berberrubine | Potassium voltage-gated channel subfamily H member 2 |
| Coptidis Rhizoma | berberrubine | Estrogen receptor |
| Coptidis Rhizoma | berberrubine | Androgen receptor |
| Coptidis Rhizoma | berberrubine | Sodium channel protein type 5 subunit alpha |
| Coptidis Rhizoma | berberrubine | Prostaglandin G/H synthase 2 |
| Coptidis Rhizoma | berberrubine | Nitric-oxide synthase, endothelial |
| Coptidis Rhizoma | berberrubine | Retinoic acid receptor RXR-alpha |
| Coptidis Rhizoma | berberrubine | mRNA of PKA Catalytic Subunit C-alpha |
| Coptidis Rhizoma | berberrubine | Trypsin-1 |
| Coptidis Rhizoma | berberrubine | Nuclear receptor coactivator 2 |
| Coptidis Rhizoma | berberrubine | Calmodulin |
| Coptidis Rhizoma | Corydaldine | Prostaglandin G/H synthase 1 |
| Coptidis Rhizoma | Corydaldine | Muscarinic acetylcholine receptor M3 |
| Coptidis Rhizoma | Corydaldine | Muscarinic acetylcholine receptor M1 |
| Coptidis Rhizoma | Corydaldine | Beta-1 adrenergic receptor |
| Coptidis Rhizoma | Corydaldine | Sodium channel protein type 5 subunit alpha |
| Coptidis Rhizoma | Corydaldine | Prostaglandin G/H synthase 2 |
| Coptidis Rhizoma | Corydaldine | Alpha-2A adrenergic receptor |
| Coptidis Rhizoma | Corydaldine | CGMP-inhibited 3',5'-cyclic phosphodiesterase A |
| Coptidis Rhizoma | Corydaldine | Sodium-dependent noradrenaline transporter |
| Coptidis Rhizoma | Corydaldine | Alpha-1A adrenergic receptor |
| Coptidis Rhizoma | Corydaldine | Alpha-1B adrenergic receptor |
| Coptidis Rhizoma | Corydaldine | Sodium-dependent dopamine transporter |
| Coptidis Rhizoma | Corydaldine | Beta-2 adrenergic receptor |
| Coptidis Rhizoma | Corydaldine | Sodium-dependent serotonin transporter |
| Coptidis Rhizoma | Corydaldine | Gamma-aminobutyric acid receptor subunit alpha-1 |
| Coptidis Rhizoma | Corydaldine | Leukotriene A-4 hydrolase |
| Coptidis Rhizoma | Corydaldine | Amine oxidase [flavin-containing] B |
| Coptidis Rhizoma | Corydaldine | mRNA of PKA Catalytic Subunit C-alpha |
| Coptidis Rhizoma | Corydaldine | cAMP-dependent protein kinase inhibitor alpha |
| Coptidis Rhizoma | epiberberine | Nitric oxide synthase, inducible |
| Coptidis Rhizoma | epiberberine | Potassium voltage-gated channel subfamily H member 2 |
| Coptidis Rhizoma | epiberberine | Estrogen receptor |
| Coptidis Rhizoma | epiberberine | Androgen receptor |
| Coptidis Rhizoma | epiberberine | Prostaglandin G/H synthase 2 |
| Coptidis Rhizoma | epiberberine | Nitric-oxide synthase, endothelial |
| Coptidis Rhizoma | epiberberine | Retinoic acid receptor RXR-alpha |
| Coptidis Rhizoma | epiberberine | mRNA of PKA Catalytic Subunit C-alpha |
| Coptidis Rhizoma | epiberberine | Trypsin-1 |
| Coptidis Rhizoma | epiberberine | Nuclear receptor coactivator 2 |
| Coptidis Rhizoma | epiberberine | cAMP and cAMP-inhibited cGMP 3',5'-cyclic phosphodiesterase 10A |
| Coptidis Rhizoma | groenlandicine | Nitric oxide synthase, inducible |
| Coptidis Rhizoma | groenlandicine | Prostaglandin G/H synthase 1 |
| Coptidis Rhizoma | groenlandicine | Estrogen receptor |
| Coptidis Rhizoma | groenlandicine | Androgen receptor |
| Coptidis Rhizoma | groenlandicine | Prostaglandin G/H synthase 2 |
| Coptidis Rhizoma | groenlandicine | Nitric-oxide synthase, endothelial |
| Coptidis Rhizoma | groenlandicine | Retinoic acid receptor RXR-alpha |
| Coptidis Rhizoma | groenlandicine | mRNA of PKA Catalytic Subunit C-alpha |
| Coptidis Rhizoma | groenlandicine | Trypsin-1 |
| Coptidis Rhizoma | groenlandicine | Nuclear receptor coactivator 2 |
| Coptidis Rhizoma | groenlandicine | Potassium voltage-gated channel subfamily H member 2 |
| Coptidis Rhizoma | groenlandicine | Sodium channel protein type 5 subunit alpha |
| Coptidis Rhizoma | groenlandicine | Coagulation factor Xa |
| Coptidis Rhizoma | groenlandicine | Beta-2 adrenergic receptor |
| Coptidis Rhizoma | groenlandicine | Heat shock protein HSP 90 |
| Coptidis Rhizoma | groenlandicine | Phosphatidylinositol-4,5-bisphosphate 3-kinase catalytic subunit, gamma isoform |
| Coptidis Rhizoma | groenlandicine | Calcium-activated potassium channel subunit alpha 1 |
| Coptidis Rhizoma | limonin | Cytochrome P450 3A4 |
| Coptidis Rhizoma | Noroxyhydrastinine | Prostaglandin G/H synthase 1 |
| Coptidis Rhizoma | Noroxyhydrastinine | Muscarinic acetylcholine receptor M1 |
| Coptidis Rhizoma | Noroxyhydrastinine | Prostaglandin G/H synthase 2 |
| Coptidis Rhizoma | Noroxyhydrastinine | Alpha-2A adrenergic receptor |
| Coptidis Rhizoma | Noroxyhydrastinine | CGMP-inhibited 3',5'-cyclic phosphodiesterase A |
| Coptidis Rhizoma | Noroxyhydrastinine | Beta-2 adrenergic receptor |
| Coptidis Rhizoma | Noroxyhydrastinine | Amine oxidase [flavin-containing] B |
| Coptidis Rhizoma | Noroxyhydrastinine | Queuine tRNA-ribosyltransferase |
| Coptidis Rhizoma | phellodendrine | Prostaglandin G/H synthase 1 |
| Coptidis Rhizoma | phellodendrine | Muscarinic acetylcholine receptor M3 |
| Coptidis Rhizoma | phellodendrine | Potassium voltage-gated channel subfamily H member 2 |
| Coptidis Rhizoma | phellodendrine | Muscarinic acetylcholine receptor M1 |
| Coptidis Rhizoma | phellodendrine | Sodium channel protein type 5 subunit alpha |
| Coptidis Rhizoma | phellodendrine | Muscarinic acetylcholine receptor M5 |
| Coptidis Rhizoma | phellodendrine | Prostaglandin G/H synthase 2 |
| Coptidis Rhizoma | phellodendrine | Carbonic anhydrase II |
| Coptidis Rhizoma | phellodendrine | Muscarinic acetylcholine receptor M4 |
| Coptidis Rhizoma | phellodendrine | Alpha-1B adrenergic receptor |
| Coptidis Rhizoma | phellodendrine | Beta-2 adrenergic receptor |
| Coptidis Rhizoma | phellodendrine | Mu-type opioid receptor |
| Coptidis Rhizoma | phellodendrine | Heat shock protein HSP 90 |
| Coptidis Rhizoma | phellodendrine | Ig gamma-1 chain C region |
| Coptidis Rhizoma | phellodendrine | Nuclear receptor coactivator 2 |
| Coptidis Rhizoma | phellodendrine | Nuclear receptor coactivator 1 |
| Coptidis Rhizoma | phellodendrine | Calmodulin |
| Coptidis Rhizoma | Ethyl caffeate | Prostaglandin G/H synthase 1 |
| Coptidis Rhizoma | Ethyl caffeate | Prostaglandin G/H synthase 2 |
| Coptidis Rhizoma | Ethyl caffeate | Beta-2 adrenergic receptor |
| Coptidis Rhizoma | Ethyl caffeate | Amine oxidase [flavin-containing] B |
| Coptidis Rhizoma | (R)-Canadine | Prostaglandin G/H synthase 1 |
| Coptidis Rhizoma | (R)-Canadine | Muscarinic acetylcholine receptor M3 |
| Coptidis Rhizoma | (R)-Canadine | Potassium voltage-gated channel subfamily H member 2 |
| Coptidis Rhizoma | (R)-Canadine | Muscarinic acetylcholine receptor M1 |
| Coptidis Rhizoma | (R)-Canadine | Sodium channel protein type 5 subunit alpha |
| Coptidis Rhizoma | (R)-Canadine | Coagulation factor Xa |
| Coptidis Rhizoma | (R)-Canadine | Muscarinic acetylcholine receptor M5 |
| Coptidis Rhizoma | (R)-Canadine | Prostaglandin G/H synthase 2 |
| Coptidis Rhizoma | (R)-Canadine | 5-hydroxytryptamine receptor 3A |
| Coptidis Rhizoma | (R)-Canadine | Alpha-2C adrenergic receptor |
| Coptidis Rhizoma | (R)-Canadine | Muscarinic acetylcholine receptor M4 |
| Coptidis Rhizoma | (R)-Canadine | Delta-type opioid receptor |
| Coptidis Rhizoma | (R)-Canadine | 5-hydroxytryptamine 2A receptor |
| Coptidis Rhizoma | (R)-Canadine | 5-hydroxytryptamine 2C receptor |
| Coptidis Rhizoma | (R)-Canadine | Alpha-1B adrenergic receptor |
| Coptidis Rhizoma | (R)-Canadine | Sodium-dependent dopamine transporter |
| Coptidis Rhizoma | (R)-Canadine | Beta-2 adrenergic receptor |
| Coptidis Rhizoma | (R)-Canadine | Alpha-1D adrenergic receptor |
| Coptidis Rhizoma | (R)-Canadine | Sodium-dependent serotonin transporter |
| Coptidis Rhizoma | (R)-Canadine | Mu-type opioid receptor |
| Coptidis Rhizoma | (R)-Canadine | Heat shock protein HSP 90 |
| Coptidis Rhizoma | (R)-Canadine | mRNA of PKA Catalytic Subunit C-alpha |
| Coptidis Rhizoma | (R)-Canadine | cAMP and cAMP-inhibited cGMP 3',5'-cyclic phosphodiesterase 10A |
| Coptidis Rhizoma | (R)-Canadine | Calmodulin |
| Coptidis Rhizoma | (R)-Canadine | Dopamine D1 receptor |
| Coptidis Rhizoma | (R)-Canadine | D(1B) dopamine receptor |
| Coptidis Rhizoma | (R)-Canadine | Retinoic acid receptor RXR-alpha |
| Coptidis Rhizoma | (R)-Canadine | Sodium-dependent noradrenaline transporter |
| Coptidis Rhizoma | (R)-Canadine | Alpha-1A adrenergic receptor |
| Coptidis Rhizoma | (R)-Canadine | Muscarinic acetylcholine receptor M2 |
| Coptidis Rhizoma | (R)-Canadine | Calcium-activated potassium channel subunit alpha 1 |
| Coptidis Rhizoma | Berlambine | Nitric oxide synthase, inducible |
| Coptidis Rhizoma | Berlambine | Prostaglandin G/H synthase 1 |
| Coptidis Rhizoma | Berlambine | Muscarinic acetylcholine receptor M3 |
| Coptidis Rhizoma | Berlambine | Potassium voltage-gated channel subfamily H member 2 |
| Coptidis Rhizoma | Berlambine | Androgen receptor |
| Coptidis Rhizoma | Berlambine | Sodium channel protein type 5 subunit alpha |
| Coptidis Rhizoma | Berlambine | Coagulation factor Xa |
| Coptidis Rhizoma | Berlambine | Prostaglandin G/H synthase 2 |
| Coptidis Rhizoma | Berlambine | Nitric-oxide synthase, endothelial |
| Coptidis Rhizoma | Berlambine | Coagulation factor VII |
| Coptidis Rhizoma | Berlambine | Retinoic acid receptor RXR-alpha |
| Coptidis Rhizoma | Berlambine | Alpha-1B adrenergic receptor |
| Coptidis Rhizoma | Berlambine | Beta-2 adrenergic receptor |
| Coptidis Rhizoma | Berlambine | Alpha-1D adrenergic receptor |
| Coptidis Rhizoma | Berlambine | Heat shock protein HSP 90 |
| Coptidis Rhizoma | Berlambine | mRNA of PKA Catalytic Subunit C-alpha |
| Coptidis Rhizoma | Berlambine | Trypsin-1 |
| Coptidis Rhizoma | Berlambine | Nuclear receptor coactivator 2 |
| Coptidis Rhizoma | Berlambine | Calcium-activated potassium channel subunit alpha 1 |
| Coptidis Rhizoma | Berlambine | Calmodulin |
| Coptidis Rhizoma | Zosimin | Prostaglandin G/H synthase 1 |
| Coptidis Rhizoma | Zosimin | Muscarinic acetylcholine receptor M3 |
| Coptidis Rhizoma | Zosimin | Thrombin |
| Coptidis Rhizoma | Zosimin | Muscarinic acetylcholine receptor M1 |
| Coptidis Rhizoma | Zosimin | Sodium channel protein type 5 subunit alpha |
| Coptidis Rhizoma | Zosimin | Coagulation factor Xa |
| Coptidis Rhizoma | Zosimin | Prostaglandin G/H synthase 2 |
| Coptidis Rhizoma | Zosimin | Acetylcholinesterase |
| Coptidis Rhizoma | Zosimin | Alpha-1B adrenergic receptor |
| Coptidis Rhizoma | Zosimin | mRNA of Protein-tyrosine phosphatase, non-receptor type 1 |
| Coptidis Rhizoma | Zosimin | Beta-2 adrenergic receptor |
| Coptidis Rhizoma | Zosimin | Dipeptidyl peptidase IV |
| Coptidis Rhizoma | Zosimin | Heat shock protein HSP 90 |
| Coptidis Rhizoma | Zosimin | Trypsin-1 |
| Coptidis Rhizoma | Zosimin | Calmodulin |
| Coptidis Rhizoma | Zosimin | Muscarinic acetylcholine receptor M5 |
| Coptidis Rhizoma | Corchoroside A_qt | Mineralocorticoid receptor |
| Coptidis Rhizoma | Corchoroside A_qt | Nuclear receptor coactivator 2 |
| Coptidis Rhizoma | FER | Prostaglandin G/H synthase 1 |
| Coptidis Rhizoma | FER | Prostaglandin G/H synthase 2 |
| Coptidis Rhizoma | FER | Nitric-oxide synthase, endothelial |
| Coptidis Rhizoma | FER | Alpha-2A adrenergic receptor |
| Coptidis Rhizoma | FER | Sodium-dependent noradrenaline transporter |
| Coptidis Rhizoma | FER | Alpha-1A adrenergic receptor |
| Coptidis Rhizoma | FER | Sodium-dependent dopamine transporter |
| Coptidis Rhizoma | FER | Beta-2 adrenergic receptor |
| Coptidis Rhizoma | FER | Leukotriene A-4 hydrolase |
| Coptidis Rhizoma | FER | Amine oxidase [flavin-containing] B |
| Coptidis Rhizoma | FER | Amine oxidase [flavin-containing] A |
| Coptidis Rhizoma | FER | Chymotrypsinogen B |
| Coptidis Rhizoma | FER | mRNA of PKA Catalytic Subunit C-alpha |
| Coptidis Rhizoma | FER | Alpha-2B adrenergic receptor |
| Coptidis Rhizoma | FER | Urokinase-type plasminogen activator |
| Coptidis Rhizoma | FER | Heat shock protein HSP 90 |
| Coptidis Rhizoma | Magnograndiolide | Gamma-aminobutyric-acid receptor alpha-2 subunit |
| Coptidis Rhizoma | Magnograndiolide | Gamma-aminobutyric acid receptor subunit alpha-1 |
| Coptidis Rhizoma | Magnograndiolide | Glutamate receptor 2 |
| Coptidis Rhizoma | Magnograndiolide | Gamma-aminobutyric-acid receptor subunit alpha-6 |
| Coptidis Rhizoma | 6-O-E-Feruloylajugol | Estrogen receptor |
| Coptidis Rhizoma | 6-O-E-Feruloylajugol | Carbonic anhydrase II |
| Coptidis Rhizoma | 6-O-E-Feruloylajugol | Glycogen synthase kinase-3 beta |
| Coptidis Rhizoma | 6-O-E-Feruloylajugol | Beta-lactamase |
| Coptidis Rhizoma | 6-O-E-Feruloylajugol | Trypsin-1 |
| Coptidis Rhizoma | 6-O-E-Feruloylajugol | Cyclin-A2 |
| Coptidis Rhizoma | 6-O-E-Feruloylajugol_qt | Estrogen receptor |
| Coptidis Rhizoma | 6-O-E-Feruloylajugol_qt | Carbonic anhydrase II |
| Coptidis Rhizoma | 6-O-E-Feruloylajugol_qt | Glycogen synthase kinase-3 beta |
| Coptidis Rhizoma | 6-O-E-Feruloylajugol_qt | Beta-lactamase |
| Coptidis Rhizoma | 6-O-E-Feruloylajugol_qt | Trypsin-1 |
| Coptidis Rhizoma | 6-O-E-Feruloylajugol_qt | Cyclin-A2 |
| Coptidis Rhizoma | palmatine | Nitric oxide synthase, inducible |
| Coptidis Rhizoma | palmatine | Prostaglandin G/H synthase 1 |
| Coptidis Rhizoma | palmatine | Potassium voltage-gated channel subfamily H member 2 |
| Coptidis Rhizoma | palmatine | Estrogen receptor |
| Coptidis Rhizoma | palmatine | Androgen receptor |
| Coptidis Rhizoma | palmatine | Sodium channel protein type 5 subunit alpha |
| Coptidis Rhizoma | palmatine | Prostaglandin G/H synthase 2 |
| Coptidis Rhizoma | palmatine | Nitric-oxide synthase, endothelial |
| Coptidis Rhizoma | palmatine | Retinoic acid receptor RXR-alpha |
| Coptidis Rhizoma | palmatine | Beta-2 adrenergic receptor |
| Coptidis Rhizoma | palmatine | Estrogen receptor beta |
| Coptidis Rhizoma | palmatine | Heat shock protein HSP 90 |
| Coptidis Rhizoma | palmatine | Trypsin-1 |
| Coptidis Rhizoma | palmatine | Proto-oncogene serine/threonine-protein kinase Pim-1 |
| Coptidis Rhizoma | palmatine | Nuclear receptor coactivator 2 |
| Coptidis Rhizoma | palmatine | Calmodulin |
| Coptidis Rhizoma | palmatine | mRNA of PKA Catalytic Subunit C-alpha |
| Coptidis Rhizoma | palmatine | Cell division protein kinase 2 |
| Coptidis Rhizoma | palmatine | Coagulation factor VII |
| Coptidis Rhizoma | jatrorrizine | Nitric oxide synthase, inducible |
| Coptidis Rhizoma | jatrorrizine | Prostaglandin G/H synthase 1 |
| Coptidis Rhizoma | jatrorrizine | Potassium voltage-gated channel subfamily H member 2 |
| Coptidis Rhizoma | jatrorrizine | Estrogen receptor |
| Coptidis Rhizoma | jatrorrizine | Androgen receptor |
| Coptidis Rhizoma | jatrorrizine | Sodium channel protein type 5 subunit alpha |
| Coptidis Rhizoma | jatrorrizine | Prostaglandin G/H synthase 2 |
| Coptidis Rhizoma | jatrorrizine | Nitric-oxide synthase, endothelial |
| Coptidis Rhizoma | jatrorrizine | Retinoic acid receptor RXR-alpha |
| Coptidis Rhizoma | jatrorrizine | Estrogen receptor beta |
| Coptidis Rhizoma | jatrorrizine | Heat shock protein HSP 90 |
| Coptidis Rhizoma | jatrorrizine | Cell division protein kinase 2 |
| Coptidis Rhizoma | jatrorrizine | Trypsin-1 |
| Coptidis Rhizoma | jatrorrizine | Proto-oncogene serine/threonine-protein kinase Pim-1 |
| Coptidis Rhizoma | jatrorrizine | Cyclin-A2 |
| Coptidis Rhizoma | jatrorrizine | Nuclear receptor coactivator 2 |
| Coptidis Rhizoma | jatrorrizine | Calmodulin |
| Coptidis Rhizoma | jatrorrizine | Calcium-activated potassium channel subunit alpha 1 |
| Coptidis Rhizoma | jatrorrizine | DNA topoisomerase II |
| Coptidis Rhizoma | jatrorrizine | mRNA of PKA Catalytic Subunit C-alpha |
| Coptidis Rhizoma | quercetin | Prostaglandin G/H synthase 1 |
| Coptidis Rhizoma | quercetin | Androgen receptor |
| Coptidis Rhizoma | quercetin | Peroxisome proliferator activated receptor gamma |
| Coptidis Rhizoma | quercetin | Prostaglandin G/H synthase 2 |
| Coptidis Rhizoma | quercetin | Heat shock protein HSP 90 |
| Coptidis Rhizoma | quercetin | Phosphatidylinositol-4,5-bisphosphate 3-kinase catalytic subunit, gamma isoform |
| Coptidis Rhizoma | quercetin | Nuclear receptor coactivator 2 |
| Coptidis Rhizoma | quercetin | Dipeptidyl peptidase IV |
| Coptidis Rhizoma | quercetin | Aldose reductase |
| Coptidis Rhizoma | quercetin | Trypsin-1 |
| Coptidis Rhizoma | quercetin | DNA topoisomerase II |
| Coptidis Rhizoma | quercetin | Thrombin |
| Coptidis Rhizoma | quercetin | Potassium voltage-gated channel subfamily H member 2 |
| Coptidis Rhizoma | quercetin | Sodium channel protein type 5 subunit alpha |
| Coptidis Rhizoma | quercetin | Coagulation factor Xa |
| Coptidis Rhizoma | quercetin | Beta-2 adrenergic receptor |
| Coptidis Rhizoma | quercetin | Stromelysin-1 |
| Coptidis Rhizoma | quercetin | mRNA of PKA Catalytic Subunit C-alpha |
| Coptidis Rhizoma | quercetin | Coagulation factor VII |
| Coptidis Rhizoma | quercetin | Nitric-oxide synthase, endothelial |
| Coptidis Rhizoma | quercetin | Retinoic acid receptor RXR-alpha |
| Coptidis Rhizoma | quercetin | Acetylcholinesterase |
| Coptidis Rhizoma | quercetin | Gamma-aminobutyric acid receptor subunit alpha-1 |
| Coptidis Rhizoma | quercetin | Amine oxidase [flavin-containing] B |
| Coptidis Rhizoma | quercetin | Transcription factor p65 |
| Coptidis Rhizoma | quercetin | Epidermal growth factor receptor |
| Coptidis Rhizoma | quercetin | RAC-alpha serine/threonine-protein kinase |
| Coptidis Rhizoma | quercetin | Vascular endothelial growth factor A |
| Coptidis Rhizoma | quercetin | G1/S-specific cyclin-D1 |
| Coptidis Rhizoma | quercetin | Apoptosis regulator Bcl-2 |
| Coptidis Rhizoma | quercetin | Bcl-2-like protein 1 |
| Coptidis Rhizoma | quercetin | Proto-oncogene c-Fos |
| Coptidis Rhizoma | quercetin | Cyclin-dependent kinase inhibitor 1 |
| Coptidis Rhizoma | quercetin | Eukaryotic translation initiation factor 6 |
| Coptidis Rhizoma | quercetin | Apoptosis regulator BAX |
| Coptidis Rhizoma | quercetin | Caspase-9 |
| Coptidis Rhizoma | quercetin | Urokinase-type plasminogen activator |
| Coptidis Rhizoma | quercetin | 72 kDa type IV collagenase |
| Coptidis Rhizoma | quercetin | Matrix metalloproteinase-9 |
| Coptidis Rhizoma | quercetin | Mitogen-activated protein kinase 1 |
| Coptidis Rhizoma | quercetin | Interleukin-10 |
| Coptidis Rhizoma | quercetin | Pro-epidermal growth factor |
| Coptidis Rhizoma | quercetin | Retinoblastoma-associated protein |
| Coptidis Rhizoma | quercetin | Tumor necrosis factor |
| Coptidis Rhizoma | quercetin | Transcription factor AP-1 |
| Coptidis Rhizoma | quercetin | Interleukin-6 |
| Coptidis Rhizoma | quercetin | Cyclin-dependent kinase inhibitor 2A, isoforms 1/2/3 |
| Coptidis Rhizoma | quercetin | Activator of 90 kDa heat shock protein ATPase homolog 1 |
| Coptidis Rhizoma | quercetin | Caspase-3 |
| Coptidis Rhizoma | quercetin | Cellular tumor antigen p53 |
| Coptidis Rhizoma | quercetin | ETS domain-containing protein Elk-1 |
| Coptidis Rhizoma | quercetin | NF-kappa-B inhibitor alpha |
| Coptidis Rhizoma | quercetin | NADPH--cytochrome P450 reductase |
| Coptidis Rhizoma | quercetin | Ornithine decarboxylase |
| Coptidis Rhizoma | quercetin | Xanthine dehydrogenase/oxidase |
| Coptidis Rhizoma | quercetin | Caspase-8 |
| Coptidis Rhizoma | quercetin | DNA topoisomerase 1 |
| Coptidis Rhizoma | quercetin | RAF proto-oncogene serine/threonine-protein kinase |
| Coptidis Rhizoma | quercetin | Superoxide dismutase [Cu-Zn] |
| Coptidis Rhizoma | quercetin | Protein kinase C alpha type |
| Coptidis Rhizoma | quercetin | Interstitial collagenase |
| Coptidis Rhizoma | quercetin | Hypoxia-inducible factor 1-alpha |
| Coptidis Rhizoma | quercetin | Signal transducer and activator of transcription 1-alpha/beta |
| Coptidis Rhizoma | quercetin | Protein CBFA2T1 |
| Coptidis Rhizoma | quercetin | Probable E3 ubiquitin-protein ligase HERC5 |
| Coptidis Rhizoma | quercetin | Cell division control protein 2 homolog |
| Coptidis Rhizoma | quercetin | 78 kDa glucose-regulated protein |
| Coptidis Rhizoma | quercetin | Receptor tyrosine-protein kinase erbB-2 |
| Coptidis Rhizoma | quercetin | Peroxisome proliferator-activated receptor gamma |
| Coptidis Rhizoma | quercetin | Acetyl-CoA carboxylase 1 |
| Coptidis Rhizoma | quercetin | Heme oxygenase 1 |
| Coptidis Rhizoma | quercetin | Cytochrome P450 3A4 |
| Coptidis Rhizoma | quercetin | Cytochrome P450 1A2 |
| Coptidis Rhizoma | quercetin | Caveolin-1 |
| Coptidis Rhizoma | quercetin | Myc proto-oncogene protein |
| Coptidis Rhizoma | quercetin | Tissue factor |
| Coptidis Rhizoma | quercetin | Gap junction alpha-1 protein |
| Coptidis Rhizoma | quercetin | Cytochrome P450 1A1 |
| Coptidis Rhizoma | quercetin | Intercellular adhesion molecule 1 |
| Coptidis Rhizoma | quercetin | Interleukin-1 beta |
| Coptidis Rhizoma | quercetin | C-C motif chemokine 2 |
| Coptidis Rhizoma | quercetin | E-selectin |
| Coptidis Rhizoma | quercetin | Vascular cell adhesion protein 1 |
| Coptidis Rhizoma | quercetin | Prostaglandin E2 receptor EP3 subtype |
| Coptidis Rhizoma | quercetin | Interleukin-8 |
| Coptidis Rhizoma | quercetin | Protein kinase C beta type |
| Coptidis Rhizoma | quercetin | Baculoviral IAP repeat-containing protein 5 |
| Coptidis Rhizoma | quercetin | Dual oxidase 2 |
| Coptidis Rhizoma | quercetin | Nitric oxide synthase, endothelial |
| Coptidis Rhizoma | quercetin | Heat shock protein beta-1 |
| Coptidis Rhizoma | quercetin | Transforming growth factor beta-1 |
| Coptidis Rhizoma | quercetin | Estrogen sulfotransferase |
| Coptidis Rhizoma | quercetin | Maltase-glucoamylase, intestinal |
| Coptidis Rhizoma | quercetin | Interleukin-2 |
| Coptidis Rhizoma | quercetin | Nuclear receptor subfamily 1 group I member 2 |
| Coptidis Rhizoma | quercetin | Cytochrome P450 1B1 |
| Coptidis Rhizoma | quercetin | G2/mitotic-specific cyclin-B1 |
| Coptidis Rhizoma | quercetin | Tissue-type plasminogen activator |
| Coptidis Rhizoma | quercetin | Thrombomodulin |
| Coptidis Rhizoma | quercetin | Plasminogen activator inhibitor 1 |
| Coptidis Rhizoma | quercetin | Collagen alpha-1(I) chain |
| Coptidis Rhizoma | quercetin | Interferon gamma |
| Coptidis Rhizoma | quercetin | Arachidonate 5-lipoxygenase |
| Coptidis Rhizoma | quercetin | Phosphatidylinositol-3,4,5-trisphosphate 3-phosphatase and dual-specificity protein phosphatase PTEN |
| Coptidis Rhizoma | quercetin | Interleukin-1 alpha |
| Coptidis Rhizoma | quercetin | Myeloperoxidase |
| Coptidis Rhizoma | quercetin | DNA topoisomerase 2-alpha |
| Coptidis Rhizoma | quercetin | Neutrophil cytosol factor 1 |
| Coptidis Rhizoma | quercetin | ATP-binding cassette sub-family G member 2 |
| Coptidis Rhizoma | quercetin | Hyaluronan synthase 2 |
| Coptidis Rhizoma | quercetin | Glutathione S-transferase P |
| Coptidis Rhizoma | quercetin | Nuclear factor erythroid 2-related factor 2 |
| Coptidis Rhizoma | quercetin | NAD(P)H dehydrogenase [quinone] 1 |
| Coptidis Rhizoma | quercetin | Poly [ADP-ribose] polymerase 1 |
| Coptidis Rhizoma | quercetin | Aryl hydrocarbon receptor |
| Coptidis Rhizoma | quercetin | 26S proteasome non-ATPase regulatory subunit 3 |
| Coptidis Rhizoma | quercetin | Solute carrier family 2, facilitated glucose transporter member 4 |
| Coptidis Rhizoma | quercetin | Collagen alpha-1(III) chain |
| Coptidis Rhizoma | quercetin | DNA gyrase subunit B |
| Coptidis Rhizoma | quercetin | C-X-C motif chemokine 11 |
| Coptidis Rhizoma | quercetin | C-X-C motif chemokine 2 |
| Coptidis Rhizoma | quercetin | DDB1- and CUL4-associated factor 5 |
| Coptidis Rhizoma | quercetin | Nuclear receptor subfamily 1 group I member 3 |
| Coptidis Rhizoma | quercetin | Serine/threonine-protein kinase Chk2 |
| Coptidis Rhizoma | quercetin | Insulin receptor |
| Coptidis Rhizoma | quercetin | Claudin-4 |
| Coptidis Rhizoma | quercetin | Peroxisome proliferator-activated receptor alpha |
| Coptidis Rhizoma | quercetin | Peroxisome proliferator-activated receptor delta |
| Coptidis Rhizoma | quercetin | Heat shock factor protein 1 |
| Coptidis Rhizoma | quercetin | C-reactive protein |
| Coptidis Rhizoma | quercetin | C-X-C motif chemokine 10 |
| Coptidis Rhizoma | quercetin | Inhibitor of nuclear factor kappa-B kinase subunit alpha |
| Coptidis Rhizoma | quercetin | Osteopontin |
| Coptidis Rhizoma | quercetin | Runt-related transcription factor 2 |
| Coptidis Rhizoma | quercetin | Ras association domain-containing protein 1 |
| Coptidis Rhizoma | quercetin | Transcription factor E2F1 |
| Coptidis Rhizoma | quercetin | Transcription factor E2F2 |
| Coptidis Rhizoma | quercetin | Prostatic acid phosphatase |
| Coptidis Rhizoma | quercetin | Cathepsin D |
| Coptidis Rhizoma | quercetin | Insulin-like growth factor-binding protein 3 |
| Coptidis Rhizoma | quercetin | Insulin-like growth factor II |
| Coptidis Rhizoma | quercetin | CD40 ligand |
| Coptidis Rhizoma | quercetin | Interferon regulatory factor 1 |
| Coptidis Rhizoma | quercetin | Receptor tyrosine-protein kinase erbB-3 |
| Coptidis Rhizoma | quercetin | Serum paraoxonase/arylesterase 1 |
| Coptidis Rhizoma | quercetin | Type I iodothyronine deiodinase |
| Coptidis Rhizoma | quercetin | Procollagen C-endopeptidase enhancer 1 |
| Coptidis Rhizoma | quercetin | Puromycin-sensitive aminopeptidase |
| Coptidis Rhizoma | quercetin | Hexokinase-2 |
| Coptidis Rhizoma | quercetin | Homeobox protein Nkx-3.1 |
| Coptidis Rhizoma | quercetin | Ras GTPase-activating protein 1 |
| Coptidis Rhizoma | quercetin | Peroxidase C1A |
| Coptidis Rhizoma | quercetin | Glutathione S-transferase Mu 1 |
| Coptidis Rhizoma | quercetin | Glutathione S-transferase Mu 2 |
| Coptidis Rhizoma | vanillic acid | Prostaglandin G/H synthase 2 |
| Coptidis Rhizoma | vanillic acid | Amine oxidase [flavin-containing] B |
| Coptidis Rhizoma | vanillic acid | Amine oxidase [flavin-containing] A |
| Coptidis Rhizoma | vanillic acid | Alpha-2A adrenergic receptor |
| Coptidis Rhizoma | vanillic acid | Prostaglandin G/H synthase 1 |
| Coptidis Rhizoma | vanillic acid | Lysozyme |
| Coptidis Rhizoma | vanillic acid | Nitric oxide synthase, endothelial |
| Coptidis Rhizoma | vanillic acid | 5,6-dihydroxyindole-2-carboxylic acid oxidase |
| Coptidis Rhizoma | hydroxytyrosol | Dopamine D1 receptor |
| Coptidis Rhizoma | hydroxytyrosol | Beta-1 adrenergic receptor |
| Coptidis Rhizoma | hydroxytyrosol | Alpha-2A adrenergic receptor |
| Coptidis Rhizoma | hydroxytyrosol | Alpha-2C adrenergic receptor |
| Coptidis Rhizoma | hydroxytyrosol | Sodium-dependent noradrenaline transporter |
| Coptidis Rhizoma | hydroxytyrosol | Alpha-1A adrenergic receptor |
| Coptidis Rhizoma | hydroxytyrosol | Alpha-2B adrenergic receptor |
| Coptidis Rhizoma | hydroxytyrosol | Alpha-1B adrenergic receptor |
| Coptidis Rhizoma | hydroxytyrosol | Sodium-dependent dopamine transporter |
| Coptidis Rhizoma | hydroxytyrosol | Alpha-1D adrenergic receptor |
| Coptidis Rhizoma | hydroxytyrosol | Urokinase-type plasminogen activator |
| Coptidis Rhizoma | hydroxytyrosol | Amine oxidase [flavin-containing] B |
| Coptidis Rhizoma | hydroxytyrosol | Amine oxidase [flavin-containing] A |
| Coptidis Rhizoma | hydroxytyrosol | Lysozyme |
| Coptidis Rhizoma | hydroxytyrosol | Chymotrypsinogen B |
| Coptidis Rhizoma | hydroxytyrosol | Nicotinate-nucleotide--dimethylbenzimidazole phosphoribosyltransferase |
| Coptidis Rhizoma | hydroxytyrosol | Trypsin-3 |
| Coptidis Rhizoma | hydroxytyrosol | Alcohol dehydrogenase 1C |
| Coptidis Rhizoma | hydroxytyrosol | Prostaglandin G/H synthase 1 |
| Coptidis Rhizoma | hydroxytyrosol | Muscarinic acetylcholine receptor M1 |
| Coptidis Rhizoma | hydroxytyrosol | Prostaglandin G/H synthase 2 |
| Coptidis Rhizoma | hydroxytyrosol | Gamma-aminobutyric-acid receptor alpha-2 subunit |
| Coptidis Rhizoma | hydroxytyrosol | Gamma-aminobutyric-acid receptor alpha-3 subunit |
| Coptidis Rhizoma | hydroxytyrosol | Muscarinic acetylcholine receptor M2 |
| Coptidis Rhizoma | hydroxytyrosol | Gamma-aminobutyric acid receptor subunit alpha-1 |
| Coptidis Rhizoma | hydroxytyrosol | Glutamate receptor 2 |
| Coptidis Rhizoma | hydroxytyrosol | Gamma-aminobutyric-acid receptor subunit alpha-6 |
| Coptidis Rhizoma | hydroxytyrosol | Transcription factor p65 |
| Coptidis Rhizoma | hydroxytyrosol | Signal transducer and activator of transcription 1-alpha/beta |
| Coptidis Rhizoma | hydroxytyrosol | Interferon regulatory factor 1 |
| Coptidis Rhizoma | p-coumaric acid | Prostaglandin G/H synthase 1 |
| Coptidis Rhizoma | p-coumaric acid | Prostaglandin G/H synthase 2 |
| Coptidis Rhizoma | p-coumaric acid | Amine oxidase [flavin-containing] B |
| Coptidis Rhizoma | p-coumaric acid | Amine oxidase [flavin-containing] A |
| Coptidis Rhizoma | p-coumaric acid | Lysozyme |
| Coptidis Rhizoma | p-coumaric acid | Chymotrypsinogen B |
| Coptidis Rhizoma | p-coumaric acid | Group IIE secretory phospholipase A2 |
| Coptidis Rhizoma | p-coumaric acid | Thermolysin |
| Coptidis Rhizoma | p-coumaric acid | Bacillolysin |
| Coptidis Rhizoma | p-coumaric acid | Nitric oxide synthase, endothelial |
| Coptidis Rhizoma | p-coumaric acid | Tyrosinase |
| Coptidis Rhizoma | p-coumaric acid | Polyphenol oxidase I, chloroplastic |
| Coptidis Rhizoma | p-coumaric acid | C-C motif chemokine 16 |
| Coptidis Rhizoma | coptisine | Nitric oxide synthase, inducible |
| Coptidis Rhizoma | coptisine | Prostaglandin G/H synthase 1 |
| Coptidis Rhizoma | coptisine | Potassium voltage-gated channel subfamily H member 2 |
| Coptidis Rhizoma | coptisine | Estrogen receptor |
| Coptidis Rhizoma | coptisine | Androgen receptor |
| Coptidis Rhizoma | coptisine | Sodium channel protein type 5 subunit alpha |
| Coptidis Rhizoma | coptisine | Prostaglandin G/H synthase 2 |
| Coptidis Rhizoma | coptisine | Nitric-oxide synthase, endothelial |
| Coptidis Rhizoma | coptisine | Trypsin-1 |
| Coptidis Rhizoma | clemastanin B_qt | Coagulation factor Xa |
| Coptidis Rhizoma | clemastanin B_qt | Prostaglandin G/H synthase 2 |
| Coptidis Rhizoma | clemastanin B_qt | Heat shock protein HSP 90 |
| Coptidis Rhizoma | clemastanin B_qt | mRNA of PKA Catalytic Subunit C-alpha |
| Coptidis Rhizoma | clemastanin B_qt | Calmodulin |
| Coptidis Rhizoma | tetrandrine | Transcription factor p65 |
| Coptidis Rhizoma | tetrandrine | G1/S-specific cyclin-D1 |
| Coptidis Rhizoma | tetrandrine | Proto-oncogene c-Fos |
| Coptidis Rhizoma | tetrandrine | Cyclin-dependent kinase inhibitor 1 |
| Coptidis Rhizoma | tetrandrine | Cell division protein kinase 4 |
| Coptidis Rhizoma | tetrandrine | Cellular tumor antigen p53 |
| Coptidis Rhizoma | tetrandrine | Probable E3 ubiquitin-protein ligase HERC5 |
| Coptidis Rhizoma | tetrandrine | Lipopolysaccharide-induced tumor necrosis factor-alpha factor |
| Coptidis Rhizoma | tetrandrine | Transforming growth factor beta-1 |
| Coptidis Rhizoma | tetrandrine | Interleukin-2 |
| Coptidis Rhizoma | tetrandrine | Interferon gamma |
| Coptidis Rhizoma | tetrandrine | Interleukin-4 |
| Coptidis Rhizoma | tetrandrine | Transcription factor E2F1 |
| Coptidis Rhizoma | tetrandrine | Interleukin-2 receptor subunit alpha |
| Coptidis Rhizoma | tetrandrine | Mothers against decapentaplegic homolog 7 |
| Coptidis Rhizoma | tetrandrine | Mothers against decapentaplegic homolog 3 |
| Coptidis Rhizoma | tetrandrine | Low affinity immunoglobulin epsilon Fc receptor |
| Coptidis Rhizoma | Worenine | Nitric oxide synthase, inducible |
| Coptidis Rhizoma | Worenine | Prostaglandin G/H synthase 1 |
| Coptidis Rhizoma | Worenine | Estrogen receptor |
| Coptidis Rhizoma | Worenine | Androgen receptor |
| Coptidis Rhizoma | Worenine | Prostaglandin G/H synthase 2 |
| Coptidis Rhizoma | Worenine | Serine/threonine-protein kinase Chk1 |
| Coptidis Rhizoma | Worenine | Proto-oncogene serine/threonine-protein kinase Pim-1 |
| Coptidis Rhizoma | GENOP | Prostaglandin G/H synthase 1 |
| Coptidis Rhizoma | GENOP | Arachidonate 5-lipoxygenase |
| Coptidis Rhizoma | GENOP | Prostaglandin G/H synthase 2 |
| Coptidis Rhizoma | GENOP | Amine oxidase [flavin-containing] B |
| Coptidis Rhizoma | GENOP | Alcohol dehydrogenase 1C |
| Coptidis Rhizoma | GENOP | Lysozyme |
| Coptidis Rhizoma | GENOP | Chymotrypsinogen B |
| Coptidis Rhizoma | GENOP | Nicotinate-nucleotide--dimethylbenzimidazole phosphoribosyltransferase |
| Coptidis Rhizoma | Methyl protocatechuate | Prostaglandin G/H synthase 1 |
| Coptidis Rhizoma | Methyl protocatechuate | Prostaglandin G/H synthase 2 |
| Coptidis Rhizoma | Methyl protocatechuate | Alpha-2A adrenergic receptor |
| Coptidis Rhizoma | Methyl protocatechuate | Amine oxidase [flavin-containing] B |
| Coptidis Rhizoma | Methyl protocatechuate | Amine oxidase [flavin-containing] A |
| Coptidis Rhizoma | Cinnamic acid, 3,4-dimethoxy- (8CI) | Prostaglandin G/H synthase 1 |
| Coptidis Rhizoma | Cinnamic acid, 3,4-dimethoxy- (8CI) | Muscarinic acetylcholine receptor M1 |
| Coptidis Rhizoma | Cinnamic acid, 3,4-dimethoxy- (8CI) | Beta-1 adrenergic receptor |
| Coptidis Rhizoma | Cinnamic acid, 3,4-dimethoxy- (8CI) | Prostaglandin G/H synthase 2 |
| Coptidis Rhizoma | Cinnamic acid, 3,4-dimethoxy- (8CI) | Nitric-oxide synthase, endothelial |
| Coptidis Rhizoma | Cinnamic acid, 3,4-dimethoxy- (8CI) | Alpha-2A adrenergic receptor |
| Coptidis Rhizoma | Cinnamic acid, 3,4-dimethoxy- (8CI) | CGMP-inhibited 3',5'-cyclic phosphodiesterase A |
| Coptidis Rhizoma | Cinnamic acid, 3,4-dimethoxy- (8CI) | Sodium-dependent noradrenaline transporter |
| Coptidis Rhizoma | Cinnamic acid, 3,4-dimethoxy- (8CI) | Alpha-1A adrenergic receptor |
| Coptidis Rhizoma | Cinnamic acid, 3,4-dimethoxy- (8CI) | Sodium-dependent dopamine transporter |
| Coptidis Rhizoma | Cinnamic acid, 3,4-dimethoxy- (8CI) | Beta-2 adrenergic receptor |
| Coptidis Rhizoma | Cinnamic acid, 3,4-dimethoxy- (8CI) | Sodium-dependent serotonin transporter |
| Coptidis Rhizoma | Cinnamic acid, 3,4-dimethoxy- (8CI) | Leukotriene A-4 hydrolase |
| Coptidis Rhizoma | Cinnamic acid, 3,4-dimethoxy- (8CI) | Amine oxidase [flavin-containing] B |
| Coptidis Rhizoma | Cinnamic acid, 3,4-dimethoxy- (8CI) | Amine oxidase [flavin-containing] A |
| Coptidis Rhizoma | Cinnamic acid, 3,4-dimethoxy- (8CI) | mRNA of PKA Catalytic Subunit C-alpha |
| Coptidis Rhizoma | Cinnamic acid, 3,4-dimethoxy- (8CI) | cAMP-dependent protein kinase inhibitor alpha |
| Coptidis Rhizoma | danshensu | Prostaglandin G/H synthase 1 |
| Coptidis Rhizoma | danshensu | Thrombin |
| Coptidis Rhizoma | danshensu | Beta-1 adrenergic receptor |
| Coptidis Rhizoma | danshensu | Prostaglandin G/H synthase 2 |
| Coptidis Rhizoma | danshensu | Alpha-2A adrenergic receptor |
| Coptidis Rhizoma | danshensu | Alpha-2C adrenergic receptor |
| Coptidis Rhizoma | danshensu | Acetylcholinesterase |
| Coptidis Rhizoma | danshensu | Alpha-2B adrenergic receptor |
| Coptidis Rhizoma | danshensu | Beta-2 adrenergic receptor |
| Coptidis Rhizoma | danshensu | Alpha-1D adrenergic receptor |
| Coptidis Rhizoma | danshensu | Dipeptidyl peptidase IV |
| Coptidis Rhizoma | danshensu | Beta-lactamase |
| Coptidis Rhizoma | danshensu | Leukotriene A-4 hydrolase |
| Coptidis Rhizoma | danshensu | Amine oxidase [flavin-containing] B |
| Coptidis Rhizoma | danshensu | Chymotrypsinogen B |
| Coptidis Rhizoma | danshensu | Transcription factor p65 |
| Coptidis Rhizoma | danshensu | Superoxide dismutase [Cu-Zn] |
| Coptidis Rhizoma | danshensu | Metalloproteinase inhibitor 1 |
| Coptidis Rhizoma | danshensu | Transforming growth factor beta-1 |
| Coptidis Rhizoma | danshensu | Collagen alpha-1(I) chain |
| Coptidis Rhizoma | danshensu | Collagen alpha-1(III) chain |
| Coptidis Rhizoma | danshensu | Actin, cytoplasmic 1 |
| Coptidis Rhizoma | danshensu | Atrial natriuretic factor |
| Coptidis Rhizoma | danshensu | Neurofibromin |
| Phellodendri chinensis Cortex | berberine | Nitric oxide synthase, inducible |
| Phellodendri chinensis Cortex | berberine | Prostaglandin G/H synthase 1 |
| Phellodendri chinensis Cortex | berberine | Potassium voltage-gated channel subfamily H member 2 |
| Phellodendri chinensis Cortex | berberine | Estrogen receptor |
| Phellodendri chinensis Cortex | berberine | Androgen receptor |
| Phellodendri chinensis Cortex | berberine | Sodium channel protein type 5 subunit alpha |
| Phellodendri chinensis Cortex | berberine | Coagulation factor Xa |
| Phellodendri chinensis Cortex | berberine | Prostaglandin G/H synthase 2 |
| Phellodendri chinensis Cortex | berberine | Nitric-oxide synthase, endothelial |
| Phellodendri chinensis Cortex | berberine | Retinoic acid receptor RXR-alpha |
| Phellodendri chinensis Cortex | berberine | Beta-2 adrenergic receptor |
| Phellodendri chinensis Cortex | berberine | Heat shock protein HSP 90 |
| Phellodendri chinensis Cortex | berberine | mRNA of PKA Catalytic Subunit C-alpha |
| Phellodendri chinensis Cortex | berberine | Trypsin-1 |
| Phellodendri chinensis Cortex | berberine | Nuclear receptor coactivator 2 |
| Phellodendri chinensis Cortex | berberine | cAMP and cAMP-inhibited cGMP 3',5'-cyclic phosphodiesterase 10A |
| Phellodendri chinensis Cortex | berberine | Calmodulin |
| Phellodendri chinensis Cortex | coptisine | Nitric oxide synthase, inducible |
| Phellodendri chinensis Cortex | coptisine | Prostaglandin G/H synthase 1 |
| Phellodendri chinensis Cortex | coptisine | Potassium voltage-gated channel subfamily H member 2 |
| Phellodendri chinensis Cortex | coptisine | Estrogen receptor |
| Phellodendri chinensis Cortex | coptisine | Androgen receptor |
| Phellodendri chinensis Cortex | coptisine | Sodium channel protein type 5 subunit alpha |
| Phellodendri chinensis Cortex | coptisine | Prostaglandin G/H synthase 2 |
| Phellodendri chinensis Cortex | coptisine | Nitric-oxide synthase, endothelial |
| Phellodendri chinensis Cortex | coptisine | Trypsin-1 |
| Phellodendri chinensis Cortex | Dauricine (8CI) | Prostaglandin G/H synthase 2 |
| Phellodendri chinensis Cortex | Dauricine (8CI) | Carbonic anhydrase II |
| Phellodendri chinensis Cortex | Dauricine (8CI) | Beta-lactamase |
| Phellodendri chinensis Cortex | Dauricine (8CI) | Amine oxidase [flavin-containing] B |
| Phellodendri chinensis Cortex | Dauricine (8CI) | Amine oxidase [flavin-containing] A |
| Phellodendri chinensis Cortex | Dauricine (8CI) | Lysozyme |
| Phellodendri chinensis Cortex | Dauricine (8CI) | Bacillolysin |
| Phellodendri chinensis Cortex | (S)-(+)-alpha-Phellandrene | Acetylcholinesterase |
| Phellodendri chinensis Cortex | (S)-(+)-alpha-Phellandrene | Gamma-aminobutyric acid receptor subunit alpha-1 |
| Phellodendri chinensis Cortex | (S)-(+)-alpha-Phellandrene | Dipeptidyl peptidase IV |
| Phellodendri chinensis Cortex | (S)-(+)-alpha-Phellandrene | Cytochrome P450-cam |
| Phellodendri chinensis Cortex | L-Limonen | Prostaglandin G/H synthase 2 |
| Phellodendri chinensis Cortex | L-Limonen | Gamma-aminobutyric acid receptor subunit alpha-1 |
| Phellodendri chinensis Cortex | L-Limonen | Alcohol dehydrogenase 1C |
| Phellodendri chinensis Cortex | L-Limonen | Cytochrome P450-cam |
| Phellodendri chinensis Cortex | L-Limonen | Alcohol dehydrogenase 1B |
| Phellodendri chinensis Cortex | L-Limonen | DNA topoisomerase II |
| Phellodendri chinensis Cortex | (s)-carvone | Gamma-aminobutyric-acid receptor alpha-2 subunit |
| Phellodendri chinensis Cortex | (s)-carvone | Gamma-aminobutyric acid receptor subunit alpha-1 |
| Phellodendri chinensis Cortex | (s)-carvone | Cytochrome P450-cam |
| Phellodendri chinensis Cortex | (s)-carvone | Glutathione S-transferase P |
| Phellodendri chinensis Cortex | (s)-carvone | Glutathione reductase, mitochondrial |
| Phellodendri chinensis Cortex | (±)-lyoniresinol | Nitric oxide synthase, inducible |
| Phellodendri chinensis Cortex | (±)-lyoniresinol | Estrogen receptor |
| Phellodendri chinensis Cortex | (±)-lyoniresinol | Androgen receptor |
| Phellodendri chinensis Cortex | (±)-lyoniresinol | Peroxisome proliferator activated receptor gamma |
| Phellodendri chinensis Cortex | (±)-lyoniresinol | Coagulation factor Xa |
| Phellodendri chinensis Cortex | (±)-lyoniresinol | Prostaglandin G/H synthase 2 |
| Phellodendri chinensis Cortex | (±)-lyoniresinol | Carbonic anhydrase II |
| Phellodendri chinensis Cortex | (±)-lyoniresinol | Coagulation factor VII |
| Phellodendri chinensis Cortex | (±)-lyoniresinol | Vascular endothelial growth factor receptor 2 |
| Phellodendri chinensis Cortex | (±)-lyoniresinol | DNA topoisomerase II |
| Phellodendri chinensis Cortex | (±)-lyoniresinol | Estrogen receptor beta |
| Phellodendri chinensis Cortex | (±)-lyoniresinol | Heat shock protein HSP 90 |
| Phellodendri chinensis Cortex | (±)-lyoniresinol | Proto-oncogene serine/threonine-protein kinase Pim-1 |
| Phellodendri chinensis Cortex | (±)-lyoniresinol | Cyclin-A2 |
| Phellodendri chinensis Cortex | (±)-lyoniresinol | Nuclear receptor coactivator 2 |
| Phellodendri chinensis Cortex | (±)-lyoniresinol | Calmodulin |
| Phellodendri chinensis Cortex | Phellavin_qt | Prostaglandin G/H synthase 2 |
| Phellodendri chinensis Cortex | Phellavin_qt | Coagulation factor VII |
| Phellodendri chinensis Cortex | Phellavin_qt | Heat shock protein HSP 90 |
| Phellodendri chinensis Cortex | phellodendrine | Prostaglandin G/H synthase 1 |
| Phellodendri chinensis Cortex | phellodendrine | Muscarinic acetylcholine receptor M3 |
| Phellodendri chinensis Cortex | phellodendrine | Potassium voltage-gated channel subfamily H member 2 |
| Phellodendri chinensis Cortex | phellodendrine | Muscarinic acetylcholine receptor M1 |
| Phellodendri chinensis Cortex | phellodendrine | Sodium channel protein type 5 subunit alpha |
| Phellodendri chinensis Cortex | phellodendrine | Coagulation factor Xa |
| Phellodendri chinensis Cortex | phellodendrine | Muscarinic acetylcholine receptor M5 |
| Phellodendri chinensis Cortex | phellodendrine | Prostaglandin G/H synthase 2 |
| Phellodendri chinensis Cortex | phellodendrine | Carbonic anhydrase II |
| Phellodendri chinensis Cortex | phellodendrine | Muscarinic acetylcholine receptor M4 |
| Phellodendri chinensis Cortex | phellodendrine | Retinoic acid receptor RXR-alpha |
| Phellodendri chinensis Cortex | phellodendrine | Delta-type opioid receptor |
| Phellodendri chinensis Cortex | phellodendrine | Alpha-1A adrenergic receptor |
| Phellodendri chinensis Cortex | phellodendrine | Alpha-1B adrenergic receptor |
| Phellodendri chinensis Cortex | phellodendrine | Beta-2 adrenergic receptor |
| Phellodendri chinensis Cortex | phellodendrine | Alpha-1D adrenergic receptor |
| Phellodendri chinensis Cortex | phellodendrine | DNA topoisomerase II |
| Phellodendri chinensis Cortex | phellodendrine | Mu-type opioid receptor |
| Phellodendri chinensis Cortex | phellodendrine | Heat shock protein HSP 90 |
| Phellodendri chinensis Cortex | phellodendrine | Ig gamma-1 chain C region |
| Phellodendri chinensis Cortex | phellodendrine | Nuclear receptor coactivator 2 |
| Phellodendri chinensis Cortex | phellodendrine | Nuclear receptor coactivator 1 |
| Phellodendri chinensis Cortex | phellodendrine | Calmodulin |
| Phellodendri chinensis Cortex | delta 7-stigmastenol | Progesterone receptor |
| Phellodendri chinensis Cortex | Phellopterin | Thrombin |
| Phellodendri chinensis Cortex | Phellopterin | Muscarinic acetylcholine receptor M1 |
| Phellodendri chinensis Cortex | Phellopterin | Sodium channel protein type 5 subunit alpha |
| Phellodendri chinensis Cortex | Phellopterin | Prostaglandin G/H synthase 2 |
| Phellodendri chinensis Cortex | Phellopterin | Retinoic acid receptor RXR-alpha |
| Phellodendri chinensis Cortex | Phellopterin | Alpha-1B adrenergic receptor |
| Phellodendri chinensis Cortex | Phellopterin | mRNA of Protein-tyrosine phosphatase, non-receptor type 1 |
| Phellodendri chinensis Cortex | Phellopterin | Beta-2 adrenergic receptor |
| Phellodendri chinensis Cortex | Phellopterin | Gamma-aminobutyric acid receptor subunit alpha-1 |
| Phellodendri chinensis Cortex | Phellopterin | Dipeptidyl peptidase IV |
| Phellodendri chinensis Cortex | Phellopterin | Heat shock protein HSP 90 |
| Phellodendri chinensis Cortex | Phellopterin | Neuronal acetylcholine receptor protein, alpha-7 chain |
| Phellodendri chinensis Cortex | PEA | Alcohol dehydrogenase 1B |
| Phellodendri chinensis Cortex | PEA | Alcohol dehydrogenase 1C |
| Phellodendri chinensis Cortex | PEA | Nicotinate-nucleotide--dimethylbenzimidazole phosphoribosyltransferase |
| Phellodendri chinensis Cortex | PEA | Trypsin-3 |
| Phellodendri chinensis Cortex | Vanillyl alcohol | Alpha-2C adrenergic receptor |
| Phellodendri chinensis Cortex | Vanillyl alcohol | Amine oxidase [flavin-containing] A |
| Phellodendri chinensis Cortex | Vanillyl alcohol | Lysozyme |
| Phellodendri chinensis Cortex | Vanillyl alcohol | Amine oxidase [flavin-containing] B |
| Phellodendri chinensis Cortex | Vanillyl alcohol | Alpha-2A adrenergic receptor |
| Phellodendri chinensis Cortex | (4R)-limonene 1beta,2beta-epoxide | Gamma-aminobutyric-acid receptor alpha-2 subunit |
| Phellodendri chinensis Cortex | (4R)-limonene 1beta,2beta-epoxide | Gamma-aminobutyric-acid receptor alpha-5 subunit |
| Phellodendri chinensis Cortex | (4R)-limonene 1beta,2beta-epoxide | Gamma-aminobutyric-acid receptor alpha-3 subunit |
| Phellodendri chinensis Cortex | (4R)-limonene 1beta,2beta-epoxide | Muscarinic acetylcholine receptor M2 |
| Phellodendri chinensis Cortex | (4R)-limonene 1beta,2beta-epoxide | Gamma-aminobutyric acid receptor subunit alpha-1 |
| Phellodendri chinensis Cortex | (4R)-limonene 1beta,2beta-epoxide | Dipeptidyl peptidase IV |
| Phellodendri chinensis Cortex | (4R)-limonene 1beta,2beta-epoxide | Cytochrome P450-cam |
| Phellodendri chinensis Cortex | (4R)-limonene 1beta,2beta-epoxide | Trypsin-1 |
| Phellodendri chinensis Cortex | Coniferol | Prostaglandin G/H synthase 1 |
| Phellodendri chinensis Cortex | Coniferol | Muscarinic acetylcholine receptor M1 |
| Phellodendri chinensis Cortex | Coniferol | Beta-1 adrenergic receptor |
| Phellodendri chinensis Cortex | Coniferol | Prostaglandin G/H synthase 2 |
| Phellodendri chinensis Cortex | Coniferol | Nitric-oxide synthase, endothelial |
| Phellodendri chinensis Cortex | Coniferol | Alpha-2A adrenergic receptor |
| Phellodendri chinensis Cortex | Coniferol | Alpha-2C adrenergic receptor |
| Phellodendri chinensis Cortex | Coniferol | Sodium-dependent noradrenaline transporter |
| Phellodendri chinensis Cortex | Coniferol | Alpha-1A adrenergic receptor |
| Phellodendri chinensis Cortex | Coniferol | Alpha-1B adrenergic receptor |
| Phellodendri chinensis Cortex | Coniferol | Sodium-dependent dopamine transporter |
| Phellodendri chinensis Cortex | Coniferol | Beta-2 adrenergic receptor |
| Phellodendri chinensis Cortex | Coniferol | Leukotriene A-4 hydrolase |
| Phellodendri chinensis Cortex | Coniferol | Amine oxidase [flavin-containing] B |
| Phellodendri chinensis Cortex | Coniferol | Amine oxidase [flavin-containing] A |
| Phellodendri chinensis Cortex | Coniferol | Muscarinic acetylcholine receptor M2 |
| Phellodendri chinensis Cortex | Coniferol | Alpha-2B adrenergic receptor |
| Phellodendri chinensis Cortex | Coniferol | Chymotrypsinogen B |
| Phellodendri chinensis Cortex | Dehydrotanshinone II A | Dopamine D1 receptor |
| Phellodendri chinensis Cortex | Dehydrotanshinone II A | Muscarinic acetylcholine receptor M3 |
| Phellodendri chinensis Cortex | Dehydrotanshinone II A | Thrombin |
| Phellodendri chinensis Cortex | Dehydrotanshinone II A | Muscarinic acetylcholine receptor M1 |
| Phellodendri chinensis Cortex | Dehydrotanshinone II A | Estrogen receptor |
| Phellodendri chinensis Cortex | Dehydrotanshinone II A | Androgen receptor |
| Phellodendri chinensis Cortex | Dehydrotanshinone II A | Sodium channel protein type 5 subunit alpha |
| Phellodendri chinensis Cortex | Dehydrotanshinone II A | Peroxisome proliferator activated receptor gamma |
| Phellodendri chinensis Cortex | Dehydrotanshinone II A | Muscarinic acetylcholine receptor M5 |
| Phellodendri chinensis Cortex | Dehydrotanshinone II A | Prostaglandin G/H synthase 2 |
| Phellodendri chinensis Cortex | Dehydrotanshinone II A | Muscarinic acetylcholine receptor M4 |
| Phellodendri chinensis Cortex | Dehydrotanshinone II A | Delta-type opioid receptor |
| Phellodendri chinensis Cortex | Dehydrotanshinone II A | Acetylcholinesterase |
| Phellodendri chinensis Cortex | Dehydrotanshinone II A | 5-hydroxytryptamine 2A receptor |
| Phellodendri chinensis Cortex | Dehydrotanshinone II A | Alpha-1A adrenergic receptor |
| Phellodendri chinensis Cortex | Dehydrotanshinone II A | Beta-2 adrenergic receptor |
| Phellodendri chinensis Cortex | Dehydrotanshinone II A | Mu-type opioid receptor |
| Phellodendri chinensis Cortex | Dehydrotanshinone II A | Gamma-aminobutyric acid receptor subunit alpha-1 |
| Phellodendri chinensis Cortex | Dehydrotanshinone II A | Dipeptidyl peptidase IV |
| Phellodendri chinensis Cortex | Dehydrotanshinone II A | Neuronal acetylcholine receptor protein, alpha-7 chain |
| Phellodendri chinensis Cortex | Dehydrotanshinone II A | Nuclear receptor coactivator 1 |
| Phellodendri chinensis Cortex | Amurensin_qt | Thrombin |
| Phellodendri chinensis Cortex | Amurensin_qt | Estrogen receptor |
| Phellodendri chinensis Cortex | Amurensin_qt | Androgen receptor |
| Phellodendri chinensis Cortex | Amurensin_qt | Peroxisome proliferator activated receptor gamma |
| Phellodendri chinensis Cortex | Amurensin_qt | Coagulation factor Xa |
| Phellodendri chinensis Cortex | Amurensin_qt | Prostaglandin G/H synthase 2 |
| Phellodendri chinensis Cortex | Amurensin_qt | Coagulation factor VII |
| Phellodendri chinensis Cortex | Amurensin_qt | DNA topoisomerase II |
| Phellodendri chinensis Cortex | Amurensin_qt | Dipeptidyl peptidase IV |
| Phellodendri chinensis Cortex | Amurensin_qt | Mitogen-activated protein kinase 14 |
| Phellodendri chinensis Cortex | Amurensin_qt | Glycogen synthase kinase-3 beta |
| Phellodendri chinensis Cortex | Amurensin_qt | Heat shock protein HSP 90 |
| Phellodendri chinensis Cortex | Amurensin_qt | Cell division protein kinase 2 |
| Phellodendri chinensis Cortex | Amurensin_qt | Trypsin-1 |
| Phellodendri chinensis Cortex | Amurensin_qt | Proto-oncogene serine/threonine-protein kinase Pim-1 |
| Phellodendri chinensis Cortex | Amurensin_qt | Vascular endothelial growth factor receptor 2 |
| Phellodendri chinensis Cortex | Amurensin_qt | Calmodulin |
| Phellodendri chinensis Cortex | rutaecarpine | Prostaglandin G/H synthase 1 |
| Phellodendri chinensis Cortex | rutaecarpine | Androgen receptor |
| Phellodendri chinensis Cortex | rutaecarpine | Sodium channel protein type 5 subunit alpha |
| Phellodendri chinensis Cortex | rutaecarpine | Coagulation factor Xa |
| Phellodendri chinensis Cortex | rutaecarpine | Prostaglandin G/H synthase 2 |
| Phellodendri chinensis Cortex | rutaecarpine | Nitric-oxide synthase, endothelial |
| Phellodendri chinensis Cortex | rutaecarpine | 5-hydroxytryptamine receptor 3A |
| Phellodendri chinensis Cortex | rutaecarpine | Retinoic acid receptor RXR-alpha |
| Phellodendri chinensis Cortex | rutaecarpine | Phosphatidylinositol-4,5-bisphosphate 3-kinase catalytic subunit, gamma isoform |
| Phellodendri chinensis Cortex | rutaecarpine | Serine/threonine-protein kinase Chk1 |
| Phellodendri chinensis Cortex | rutaecarpine | mRNA of PKA Catalytic Subunit C-alpha |
| Phellodendri chinensis Cortex | rutaecarpine | 72 kDa type IV collagenase |
| Phellodendri chinensis Cortex | rutaecarpine | Matrix metalloproteinase-9 |
| Phellodendri chinensis Cortex | rutaecarpine | Tumor necrosis factor |
| Phellodendri chinensis Cortex | rutaecarpine | Cytochrome P450 3A4 |
| Phellodendri chinensis Cortex | rutaecarpine | Cytochrome P450 1A2 |
| Phellodendri chinensis Cortex | rutaecarpine | Interleukin-4 |
| Phellodendri chinensis Cortex | rutaecarpine | Cytochrome P450 2B6 |
| Phellodendri chinensis Cortex | Skimmianin | Retinoic acid receptor RXR-alpha |
| Phellodendri chinensis Cortex | Skimmianin | Gamma-aminobutyric acid receptor subunit alpha-1 |
| Phellodendri chinensis Cortex | Skimmianin | Heat shock protein HSP 90 |
| Phellodendri chinensis Cortex | Skimmianin | mRNA of PKA Catalytic Subunit C-alpha |
| Phellodendri chinensis Cortex | Skimmianin | Phosphatidylinositol-4,5-bisphosphate 3-kinase catalytic subunit, gamma isoform |
| Phellodendri chinensis Cortex | Fagarine | Heat shock protein HSP 90 |
| Phellodendri chinensis Cortex | Ferulic Acid | Prostaglandin G/H synthase 1 |
| Phellodendri chinensis Cortex | Ferulic Acid | Muscarinic acetylcholine receptor M1 |
| Phellodendri chinensis Cortex | Ferulic Acid | Beta-1 adrenergic receptor |
| Phellodendri chinensis Cortex | Ferulic Acid | Prostaglandin G/H synthase 2 |
| Phellodendri chinensis Cortex | Ferulic Acid | Nitric-oxide synthase, endothelial |
| Phellodendri chinensis Cortex | Ferulic Acid | Alpha-2A adrenergic receptor |
| Phellodendri chinensis Cortex | Ferulic Acid | Sodium-dependent noradrenaline transporter |
| Phellodendri chinensis Cortex | Ferulic Acid | Alpha-1A adrenergic receptor |
| Phellodendri chinensis Cortex | Ferulic Acid | Muscarinic acetylcholine receptor M2 |
| Phellodendri chinensis Cortex | Ferulic Acid | Alpha-2B adrenergic receptor |
| Phellodendri chinensis Cortex | Ferulic Acid | Sodium-dependent dopamine transporter |
| Phellodendri chinensis Cortex | Ferulic Acid | Beta-2 adrenergic receptor |
| Phellodendri chinensis Cortex | Ferulic Acid | Beta-lactamase |
| Phellodendri chinensis Cortex | Ferulic Acid | Leukotriene A-4 hydrolase |
| Phellodendri chinensis Cortex | Ferulic Acid | Amine oxidase [flavin-containing] B |
| Phellodendri chinensis Cortex | Ferulic Acid | Amine oxidase [flavin-containing] A |
| Phellodendri chinensis Cortex | Ferulic Acid | cAMP-dependent protein kinase inhibitor alpha |
| Phellodendri chinensis Cortex | Chelerythrine | Prostaglandin G/H synthase 1 |
| Phellodendri chinensis Cortex | Chelerythrine | Potassium voltage-gated channel subfamily H member 2 |
| Phellodendri chinensis Cortex | Chelerythrine | Prostaglandin G/H synthase 2 |
| Phellodendri chinensis Cortex | Chelerythrine | Retinoic acid receptor RXR-alpha |
| Phellodendri chinensis Cortex | Chelerythrine | mRNA of PKA Catalytic Subunit C-alpha |
| Phellodendri chinensis Cortex | Chelerythrine | Nuclear receptor coactivator 2 |
| Phellodendri chinensis Cortex | Stigmasterol | Progesterone receptor |
| Phellodendri chinensis Cortex | Stigmasterol | Mineralocorticoid receptor |
| Phellodendri chinensis Cortex | Stigmasterol | Nuclear receptor coactivator 2 |
| Phellodendri chinensis Cortex | Stigmasterol | Alcohol dehydrogenase 1C |
| Phellodendri chinensis Cortex | Stigmasterol | Ig gamma-1 chain C region |
| Phellodendri chinensis Cortex | Stigmasterol | Retinoic acid receptor RXR-alpha |
| Phellodendri chinensis Cortex | Stigmasterol | Nuclear receptor coactivator 1 |
| Phellodendri chinensis Cortex | Stigmasterol | Prostaglandin G/H synthase 1 |
| Phellodendri chinensis Cortex | Stigmasterol | Prostaglandin G/H synthase 2 |
| Phellodendri chinensis Cortex | Stigmasterol | Alpha-2A adrenergic receptor |
| Phellodendri chinensis Cortex | Stigmasterol | Sodium-dependent noradrenaline transporter |
| Phellodendri chinensis Cortex | Stigmasterol | Sodium-dependent dopamine transporter |
| Phellodendri chinensis Cortex | Stigmasterol | Beta-2 adrenergic receptor |
| Phellodendri chinensis Cortex | Stigmasterol | Aldose reductase |
| Phellodendri chinensis Cortex | Stigmasterol | Urokinase-type plasminogen activator |
| Phellodendri chinensis Cortex | Stigmasterol | Leukotriene A-4 hydrolase |
| Phellodendri chinensis Cortex | Stigmasterol | Amine oxidase [flavin-containing] B |
| Phellodendri chinensis Cortex | Stigmasterol | Amine oxidase [flavin-containing] A |
| Phellodendri chinensis Cortex | Stigmasterol | mRNA of PKA Catalytic Subunit C-alpha |
| Phellodendri chinensis Cortex | Stigmasterol | Chymotrypsinogen B |
| Phellodendri chinensis Cortex | Stigmasterol | Muscarinic acetylcholine receptor M3 |
| Phellodendri chinensis Cortex | Stigmasterol | Muscarinic acetylcholine receptor M1 |
| Phellodendri chinensis Cortex | Stigmasterol | Beta-1 adrenergic receptor |
| Phellodendri chinensis Cortex | Stigmasterol | Sodium channel protein type 5 subunit alpha |
| Phellodendri chinensis Cortex | Stigmasterol | 5-hydroxytryptamine 2A receptor |
| Phellodendri chinensis Cortex | Stigmasterol | Alpha-1A adrenergic receptor |
| Phellodendri chinensis Cortex | Stigmasterol | Gamma-aminobutyric-acid receptor alpha-3 subunit |
| Phellodendri chinensis Cortex | Stigmasterol | Muscarinic acetylcholine receptor M2 |
| Phellodendri chinensis Cortex | Stigmasterol | Alpha-1B adrenergic receptor |
| Phellodendri chinensis Cortex | Stigmasterol | Gamma-aminobutyric acid receptor subunit alpha-1 |
| Phellodendri chinensis Cortex | Stigmasterol | Neuronal acetylcholine receptor protein, alpha-7 chain |
| Phellodendri chinensis Cortex | Worenine | Nitric oxide synthase, inducible |
| Phellodendri chinensis Cortex | Worenine | Prostaglandin G/H synthase 1 |
| Phellodendri chinensis Cortex | Worenine | Estrogen receptor |
| Phellodendri chinensis Cortex | Worenine | Androgen receptor |
| Phellodendri chinensis Cortex | Worenine | Prostaglandin G/H synthase 2 |
| Phellodendri chinensis Cortex | Worenine | Serine/threonine-protein kinase Chk1 |
| Phellodendri chinensis Cortex | Worenine | Proto-oncogene serine/threonine-protein kinase Pim-1 |
| Phellodendri chinensis Cortex | Cavidine | Prostaglandin G/H synthase 1 |
| Phellodendri chinensis Cortex | Cavidine | Muscarinic acetylcholine receptor M3 |
| Phellodendri chinensis Cortex | Cavidine | Potassium voltage-gated channel subfamily H member 2 |
| Phellodendri chinensis Cortex | Cavidine | Muscarinic acetylcholine receptor M1 |
| Phellodendri chinensis Cortex | Cavidine | Beta-1 adrenergic receptor |
| Phellodendri chinensis Cortex | Cavidine | Sodium channel protein type 5 subunit alpha |
| Phellodendri chinensis Cortex | Cavidine | Coagulation factor Xa |
| Phellodendri chinensis Cortex | Cavidine | Muscarinic acetylcholine receptor M5 |
| Phellodendri chinensis Cortex | Cavidine | Prostaglandin G/H synthase 2 |
| Phellodendri chinensis Cortex | Cavidine | 5-hydroxytryptamine receptor 3A |
| Phellodendri chinensis Cortex | Cavidine | Alpha-2C adrenergic receptor |
| Phellodendri chinensis Cortex | Cavidine | Muscarinic acetylcholine receptor M4 |
| Phellodendri chinensis Cortex | Cavidine | Retinoic acid receptor RXR-alpha |
| Phellodendri chinensis Cortex | Cavidine | Delta-type opioid receptor |
| Phellodendri chinensis Cortex | Cavidine | 5-hydroxytryptamine 2A receptor |
| Phellodendri chinensis Cortex | Cavidine | 5-hydroxytryptamine 2C receptor |
| Phellodendri chinensis Cortex | Cavidine | Alpha-1B adrenergic receptor |
| Phellodendri chinensis Cortex | Cavidine | Beta-2 adrenergic receptor |
| Phellodendri chinensis Cortex | Cavidine | Alpha-1D adrenergic receptor |
| Phellodendri chinensis Cortex | Cavidine | DNA topoisomerase II |
| Phellodendri chinensis Cortex | Cavidine | Mu-type opioid receptor |
| Phellodendri chinensis Cortex | Cavidine | Heat shock protein HSP 90 |
| Phellodendri chinensis Cortex | Cavidine | Retinoic acid receptor RXR-beta |
| Phellodendri chinensis Cortex | Cavidine | Calmodulin |
| Phellodendri chinensis Cortex | Cavidine | Dopamine D1 receptor |
| Phellodendri chinensis Cortex | Cavidine | Sodium-dependent serotonin transporter |
| Phellodendri chinensis Cortex | Cavidine | Coagulation factor VII |
| Phellodendri chinensis Cortex | Cavidine | cAMP and cAMP-inhibited cGMP 3',5'-cyclic phosphodiesterase 10A |
| Phellodendri chinensis Cortex | Hericenone H | Thrombin |
| Phellodendri chinensis Cortex | Syrigin | Prostaglandin G/H synthase 2 |
| Phellodendri chinensis Cortex | Syrigin | Nitric-oxide synthase, endothelial |
| Phellodendri chinensis Cortex | Syrigin | Prostaglandin G/H synthase 1 |
| Phellodendri chinensis Cortex | Syrigin | Muscarinic acetylcholine receptor M3 |
| Phellodendri chinensis Cortex | Syrigin | Muscarinic acetylcholine receptor M1 |
| Phellodendri chinensis Cortex | Syrigin | Beta-1 adrenergic receptor |
| Phellodendri chinensis Cortex | Syrigin | Alpha-2A adrenergic receptor |
| Phellodendri chinensis Cortex | Syrigin | Sodium-dependent noradrenaline transporter |
| Phellodendri chinensis Cortex | Syrigin | Alpha-1A adrenergic receptor |
| Phellodendri chinensis Cortex | Syrigin | Muscarinic acetylcholine receptor M2 |
| Phellodendri chinensis Cortex | Syrigin | Alpha-2B adrenergic receptor |
| Phellodendri chinensis Cortex | Syrigin | Alpha-1B adrenergic receptor |
| Phellodendri chinensis Cortex | Syrigin | Sodium-dependent dopamine transporter |
| Phellodendri chinensis Cortex | Syrigin | Beta-2 adrenergic receptor |
| Phellodendri chinensis Cortex | Syrigin | Gamma-aminobutyric acid receptor subunit alpha-1 |
| Phellodendri chinensis Cortex | Syrigin | Leukotriene A-4 hydrolase |
| Phellodendri chinensis Cortex | Syrigin | Amine oxidase [flavin-containing] B |
| Phellodendri chinensis Cortex | Syrigin | cAMP-dependent protein kinase inhibitor alpha |
| Phellodendri chinensis Cortex | 4-[(Z)-3-hydroxyprop-1-enyl]-2,6-dimethoxyphenol | Nitric oxide synthase, inducible |
| Phellodendri chinensis Cortex | 4-[(Z)-3-hydroxyprop-1-enyl]-2,6-dimethoxyphenol | Prostaglandin G/H synthase 1 |
| Phellodendri chinensis Cortex | 4-[(Z)-3-hydroxyprop-1-enyl]-2,6-dimethoxyphenol | Dopamine D1 receptor |
| Phellodendri chinensis Cortex | 4-[(Z)-3-hydroxyprop-1-enyl]-2,6-dimethoxyphenol | Muscarinic acetylcholine receptor M3 |
| Phellodendri chinensis Cortex | 4-[(Z)-3-hydroxyprop-1-enyl]-2,6-dimethoxyphenol | Muscarinic acetylcholine receptor M1 |
| Phellodendri chinensis Cortex | 4-[(Z)-3-hydroxyprop-1-enyl]-2,6-dimethoxyphenol | Beta-1 adrenergic receptor |
| Phellodendri chinensis Cortex | 4-[(Z)-3-hydroxyprop-1-enyl]-2,6-dimethoxyphenol | Prostaglandin G/H synthase 2 |
| Phellodendri chinensis Cortex | 4-[(Z)-3-hydroxyprop-1-enyl]-2,6-dimethoxyphenol | Nitric-oxide synthase, endothelial |
| Phellodendri chinensis Cortex | 4-[(Z)-3-hydroxyprop-1-enyl]-2,6-dimethoxyphenol | Alpha-2A adrenergic receptor |
| Phellodendri chinensis Cortex | 4-[(Z)-3-hydroxyprop-1-enyl]-2,6-dimethoxyphenol | Sodium-dependent noradrenaline transporter |
| Phellodendri chinensis Cortex | 4-[(Z)-3-hydroxyprop-1-enyl]-2,6-dimethoxyphenol | Alpha-1A adrenergic receptor |
| Phellodendri chinensis Cortex | 4-[(Z)-3-hydroxyprop-1-enyl]-2,6-dimethoxyphenol | Muscarinic acetylcholine receptor M2 |
| Phellodendri chinensis Cortex | 4-[(Z)-3-hydroxyprop-1-enyl]-2,6-dimethoxyphenol | Alpha-2B adrenergic receptor |
| Phellodendri chinensis Cortex | 4-[(Z)-3-hydroxyprop-1-enyl]-2,6-dimethoxyphenol | Alpha-1B adrenergic receptor |
| Phellodendri chinensis Cortex | 4-[(Z)-3-hydroxyprop-1-enyl]-2,6-dimethoxyphenol | Sodium-dependent dopamine transporter |
| Phellodendri chinensis Cortex | 4-[(Z)-3-hydroxyprop-1-enyl]-2,6-dimethoxyphenol | Beta-2 adrenergic receptor |
| Phellodendri chinensis Cortex | 4-[(Z)-3-hydroxyprop-1-enyl]-2,6-dimethoxyphenol | Gamma-aminobutyric acid receptor subunit alpha-1 |
| Phellodendri chinensis Cortex | 4-[(Z)-3-hydroxyprop-1-enyl]-2,6-dimethoxyphenol | Urokinase-type plasminogen activator |
| Phellodendri chinensis Cortex | 4-[(Z)-3-hydroxyprop-1-enyl]-2,6-dimethoxyphenol | Heat shock protein HSP 90 |
| Phellodendri chinensis Cortex | 4-[(Z)-3-hydroxyprop-1-enyl]-2,6-dimethoxyphenol | Leukotriene A-4 hydrolase |
| Phellodendri chinensis Cortex | 4-[(Z)-3-hydroxyprop-1-enyl]-2,6-dimethoxyphenol | Amine oxidase [flavin-containing] B |
| Phellodendri chinensis Cortex | 4-[(Z)-3-hydroxyprop-1-enyl]-2,6-dimethoxyphenol | cAMP-dependent protein kinase inhibitor alpha |
| Phellodendri chinensis Cortex | beta-sitosterol | Progesterone receptor |
| Phellodendri chinensis Cortex | beta-sitosterol | Nuclear receptor coactivator 2 |
| Phellodendri chinensis Cortex | beta-sitosterol | Prostaglandin G/H synthase 1 |
| Phellodendri chinensis Cortex | beta-sitosterol | Prostaglandin G/H synthase 2 |
| Phellodendri chinensis Cortex | beta-sitosterol | Heat shock protein HSP 90 |
| Phellodendri chinensis Cortex | beta-sitosterol | Phosphatidylinositol-4,5-bisphosphate 3-kinase catalytic subunit, gamma isoform |
| Phellodendri chinensis Cortex | beta-sitosterol | Potassium voltage-gated channel subfamily H member 2 |
| Phellodendri chinensis Cortex | beta-sitosterol | mRNA of PKA Catalytic Subunit C-alpha |
| Phellodendri chinensis Cortex | beta-sitosterol | Dopamine D1 receptor |
| Phellodendri chinensis Cortex | beta-sitosterol | Muscarinic acetylcholine receptor M3 |
| Phellodendri chinensis Cortex | beta-sitosterol | Muscarinic acetylcholine receptor M1 |
| Phellodendri chinensis Cortex | beta-sitosterol | Sodium channel protein type 5 subunit alpha |
| Phellodendri chinensis Cortex | beta-sitosterol | Gamma-aminobutyric-acid receptor alpha-2 subunit |
| Phellodendri chinensis Cortex | beta-sitosterol | Muscarinic acetylcholine receptor M4 |
| Phellodendri chinensis Cortex | beta-sitosterol | CGMP-inhibited 3',5'-cyclic phosphodiesterase A |
| Phellodendri chinensis Cortex | beta-sitosterol | 5-hydroxytryptamine 2A receptor |
| Phellodendri chinensis Cortex | beta-sitosterol | Gamma-aminobutyric-acid receptor alpha-5 subunit |
| Phellodendri chinensis Cortex | beta-sitosterol | Alpha-1A adrenergic receptor |
| Phellodendri chinensis Cortex | beta-sitosterol | Gamma-aminobutyric-acid receptor alpha-3 subunit |
| Phellodendri chinensis Cortex | beta-sitosterol | Muscarinic acetylcholine receptor M2 |
| Phellodendri chinensis Cortex | beta-sitosterol | Alpha-1B adrenergic receptor |
| Phellodendri chinensis Cortex | beta-sitosterol | Beta-2 adrenergic receptor |
| Phellodendri chinensis Cortex | beta-sitosterol | Neuronal acetylcholine receptor subunit alpha-2 |
| Phellodendri chinensis Cortex | beta-sitosterol | Sodium-dependent serotonin transporter |
| Phellodendri chinensis Cortex | beta-sitosterol | Mu-type opioid receptor |
| Phellodendri chinensis Cortex | beta-sitosterol | Gamma-aminobutyric acid receptor subunit alpha-1 |
| Phellodendri chinensis Cortex | beta-sitosterol | Neuronal acetylcholine receptor protein, alpha-7 chain |
| Phellodendri chinensis Cortex | beta-sitosterol | Cytochrome P450-cam |
| Phellodendri chinensis Cortex | beta-sitosterol | Apoptosis regulator Bcl-2 |
| Phellodendri chinensis Cortex | beta-sitosterol | Apoptosis regulator BAX |
| Phellodendri chinensis Cortex | beta-sitosterol | Caspase-9 |
| Phellodendri chinensis Cortex | beta-sitosterol | Transcription factor AP-1 |
| Phellodendri chinensis Cortex | beta-sitosterol | Caspase-3 |
| Phellodendri chinensis Cortex | beta-sitosterol | Caspase-8 |
| Phellodendri chinensis Cortex | beta-sitosterol | Protein kinase C alpha type |
| Phellodendri chinensis Cortex | beta-sitosterol | Transforming growth factor beta-1 |
| Phellodendri chinensis Cortex | beta-sitosterol | Serum paraoxonase/arylesterase 1 |
| Phellodendri chinensis Cortex | beta-sitosterol | Microtubule-associated protein 2 |
| Phellodendri chinensis Cortex | Magnograndiolide | Gamma-aminobutyric-acid receptor alpha-2 subunit |
| Phellodendri chinensis Cortex | Magnograndiolide | Gamma-aminobutyric acid receptor subunit alpha-1 |
| Phellodendri chinensis Cortex | Magnograndiolide | Glutamate receptor 2 |
| Phellodendri chinensis Cortex | Magnograndiolide | Gamma-aminobutyric-acid receptor subunit alpha-6 |
| Phellodendri chinensis Cortex | (2S,3S)-3,5,7-trihydroxy-2-(4-hydroxyphenyl)chroman-4-one | Prostaglandin G/H synthase 1 |
| Phellodendri chinensis Cortex | (2S,3S)-3,5,7-trihydroxy-2-(4-hydroxyphenyl)chroman-4-one | Prostaglandin G/H synthase 2 |
| Phellodendri chinensis Cortex | (2S,3S)-3,5,7-trihydroxy-2-(4-hydroxyphenyl)chroman-4-one | Heat shock protein HSP 90 |
| Phellodendri chinensis Cortex | magnoflorine | Prostaglandin G/H synthase 1 |
| Phellodendri chinensis Cortex | magnoflorine | Potassium voltage-gated channel subfamily H member 2 |
| Phellodendri chinensis Cortex | magnoflorine | Muscarinic acetylcholine receptor M1 |
| Phellodendri chinensis Cortex | magnoflorine | Androgen receptor |
| Phellodendri chinensis Cortex | magnoflorine | Sodium channel protein type 5 subunit alpha |
| Phellodendri chinensis Cortex | magnoflorine | Prostaglandin G/H synthase 2 |
| Phellodendri chinensis Cortex | magnoflorine | Retinoic acid receptor RXR-alpha |
| Phellodendri chinensis Cortex | magnoflorine | Delta-type opioid receptor |
| Phellodendri chinensis Cortex | magnoflorine | Acetylcholinesterase |
| Phellodendri chinensis Cortex | magnoflorine | Alpha-1B adrenergic receptor |
| Phellodendri chinensis Cortex | magnoflorine | DNA topoisomerase II |
| Phellodendri chinensis Cortex | magnoflorine | Pregnane X receptor |
| Phellodendri chinensis Cortex | magnoflorine | Heat shock protein HSP 90 |
| Phellodendri chinensis Cortex | magnoflorine | Nuclear receptor coactivator 1 |
| Phellodendri chinensis Cortex | magnoflorine | Calmodulin |
| Phellodendri chinensis Cortex | magnoflorine | Nitric-oxide synthase, endothelial |
| Phellodendri chinensis Cortex | magnoflorine | Carbonic anhydrase II |
| Phellodendri chinensis Cortex | magnoflorine | Trypsin-1 |
| Phellodendri chinensis Cortex | magnoflorine | Nuclear receptor coactivator 2 |
| Phellodendri chinensis Cortex | magnoflorine | Calcium-activated potassium channel subunit alpha 1 |
| Phellodendri chinensis Cortex | magnoflorine | Muscarinic acetylcholine receptor M3 |
| Phellodendri chinensis Cortex | Menisporphine | Nitric oxide synthase, inducible |
| Phellodendri chinensis Cortex | Menisporphine | Prostaglandin G/H synthase 1 |
| Phellodendri chinensis Cortex | Menisporphine | Thrombin |
| Phellodendri chinensis Cortex | Menisporphine | Potassium voltage-gated channel subfamily H member 2 |
| Phellodendri chinensis Cortex | Menisporphine | Estrogen receptor |
| Phellodendri chinensis Cortex | Menisporphine | Androgen receptor |
| Phellodendri chinensis Cortex | Menisporphine | Sodium channel protein type 5 subunit alpha |
| Phellodendri chinensis Cortex | Menisporphine | Peroxisome proliferator activated receptor gamma |
| Phellodendri chinensis Cortex | Menisporphine | Coagulation factor Xa |
| Phellodendri chinensis Cortex | Menisporphine | Prostaglandin G/H synthase 2 |
| Phellodendri chinensis Cortex | Menisporphine | Nitric-oxide synthase, endothelial |
| Phellodendri chinensis Cortex | Menisporphine | Retinoic acid receptor RXR-alpha |
| Phellodendri chinensis Cortex | Menisporphine | Dipeptidyl peptidase IV |
| Phellodendri chinensis Cortex | Menisporphine | Mitogen-activated protein kinase 14 |
| Phellodendri chinensis Cortex | Menisporphine | Glycogen synthase kinase-3 beta |
| Phellodendri chinensis Cortex | Menisporphine | Cell division protein kinase 2 |
| Phellodendri chinensis Cortex | Menisporphine | Phosphatidylinositol-4,5-bisphosphate 3-kinase catalytic subunit, gamma isoform |
| Phellodendri chinensis Cortex | Menisporphine | Proto-oncogene serine/threonine-protein kinase Pim-1 |
| Phellodendri chinensis Cortex | Menisporphine | Cyclin-A2 |
| Phellodendri chinensis Cortex | Menisporphine | Acetylcholinesterase |
| Phellodendri chinensis Cortex | Menisporphine | Beta-2 adrenergic receptor |
| Phellodendri chinensis Cortex | Menisporphine | Trypsin-1 |
| Phellodendri chinensis Cortex | Menisporphine | Nuclear receptor coactivator 2 |
| Phellodendri chinensis Cortex | Menisporphine | Calmodulin |
| Phellodendri chinensis Cortex | palmatine | Nitric oxide synthase, inducible |
| Phellodendri chinensis Cortex | palmatine | Prostaglandin G/H synthase 1 |
| Phellodendri chinensis Cortex | palmatine | Potassium voltage-gated channel subfamily H member 2 |
| Phellodendri chinensis Cortex | palmatine | Estrogen receptor |
| Phellodendri chinensis Cortex | palmatine | Androgen receptor |
| Phellodendri chinensis Cortex | palmatine | Sodium channel protein type 5 subunit alpha |
| Phellodendri chinensis Cortex | palmatine | Prostaglandin G/H synthase 2 |
| Phellodendri chinensis Cortex | palmatine | Nitric-oxide synthase, endothelial |
| Phellodendri chinensis Cortex | palmatine | Retinoic acid receptor RXR-alpha |
| Phellodendri chinensis Cortex | palmatine | Beta-2 adrenergic receptor |
| Phellodendri chinensis Cortex | palmatine | Estrogen receptor beta |
| Phellodendri chinensis Cortex | palmatine | Heat shock protein HSP 90 |
| Phellodendri chinensis Cortex | palmatine | Trypsin-1 |
| Phellodendri chinensis Cortex | palmatine | Proto-oncogene serine/threonine-protein kinase Pim-1 |
| Phellodendri chinensis Cortex | palmatine | Nuclear receptor coactivator 2 |
| Phellodendri chinensis Cortex | palmatine | Calmodulin |
| Phellodendri chinensis Cortex | palmatine | mRNA of PKA Catalytic Subunit C-alpha |
| Phellodendri chinensis Cortex | palmatine | Cell division protein kinase 2 |
| Phellodendri chinensis Cortex | palmatine | Coagulation factor VII |
| Phellodendri chinensis Cortex | Fumarine | Prostaglandin G/H synthase 1 |
| Phellodendri chinensis Cortex | Fumarine | Muscarinic acetylcholine receptor M3 |
| Phellodendri chinensis Cortex | Fumarine | Potassium voltage-gated channel subfamily H member 2 |
| Phellodendri chinensis Cortex | Fumarine | Muscarinic acetylcholine receptor M1 |
| Phellodendri chinensis Cortex | Fumarine | Sodium channel protein type 5 subunit alpha |
| Phellodendri chinensis Cortex | Fumarine | Coagulation factor Xa |
| Phellodendri chinensis Cortex | Fumarine | Muscarinic acetylcholine receptor M5 |
| Phellodendri chinensis Cortex | Fumarine | Prostaglandin G/H synthase 2 |
| Phellodendri chinensis Cortex | Fumarine | 5-hydroxytryptamine receptor 3A |
| Phellodendri chinensis Cortex | Fumarine | Coagulation factor VII |
| Phellodendri chinensis Cortex | Fumarine | Muscarinic acetylcholine receptor M4 |
| Phellodendri chinensis Cortex | Fumarine | Delta-type opioid receptor |
| Phellodendri chinensis Cortex | Fumarine | 5-hydroxytryptamine 2A receptor |
| Phellodendri chinensis Cortex | Fumarine | Alpha-1B adrenergic receptor |
| Phellodendri chinensis Cortex | Fumarine | Beta-2 adrenergic receptor |
| Phellodendri chinensis Cortex | Fumarine | Alpha-1D adrenergic receptor |
| Phellodendri chinensis Cortex | Fumarine | Mu-type opioid receptor |
| Phellodendri chinensis Cortex | Fumarine | Heat shock protein HSP 90 |
| Phellodendri chinensis Cortex | Fumarine | mRNA of PKA Catalytic Subunit C-alpha |
| Phellodendri chinensis Cortex | Fumarine | Calmodulin |
| Phellodendri chinensis Cortex | Fumarine | Sodium-dependent serotonin transporter |
| Phellodendri chinensis Cortex | Fumarine | Voltage-dependent L-type calcium channel subunit alpha-1S |
| Phellodendri chinensis Cortex | Fumarine | CGMP-inhibited 3',5'-cyclic phosphodiesterase A |
| Phellodendri chinensis Cortex | Fumarine | Sodium-dependent dopamine transporter |
| Phellodendri chinensis Cortex | Fumarine | Type IV phosphodiesterase |
| Phellodendri chinensis Cortex | Fumarine | DNA topoisomerase II |
| Phellodendri chinensis Cortex | Fumarine | Dopamine D1 receptor |
| Phellodendri chinensis Cortex | Fumarine | Vascular endothelial growth factor receptor 2 |
| Phellodendri chinensis Cortex | Isocorypalmine | Prostaglandin G/H synthase 1 |
| Phellodendri chinensis Cortex | Isocorypalmine | Dopamine D1 receptor |
| Phellodendri chinensis Cortex | Isocorypalmine | Muscarinic acetylcholine receptor M3 |
| Phellodendri chinensis Cortex | Isocorypalmine | Potassium voltage-gated channel subfamily H member 2 |
| Phellodendri chinensis Cortex | Isocorypalmine | Muscarinic acetylcholine receptor M1 |
| Phellodendri chinensis Cortex | Isocorypalmine | D(1B) dopamine receptor |
| Phellodendri chinensis Cortex | Isocorypalmine | Sodium channel protein type 5 subunit alpha |
| Phellodendri chinensis Cortex | Isocorypalmine | Coagulation factor Xa |
| Phellodendri chinensis Cortex | Isocorypalmine | Muscarinic acetylcholine receptor M5 |
| Phellodendri chinensis Cortex | Isocorypalmine | Prostaglandin G/H synthase 2 |
| Phellodendri chinensis Cortex | Isocorypalmine | 5-hydroxytryptamine receptor 3A |
| Phellodendri chinensis Cortex | Isocorypalmine | Carbonic anhydrase II |
| Phellodendri chinensis Cortex | Isocorypalmine | Alpha-2C adrenergic receptor |
| Phellodendri chinensis Cortex | Isocorypalmine | Muscarinic acetylcholine receptor M4 |
| Phellodendri chinensis Cortex | Isocorypalmine | Retinoic acid receptor RXR-alpha |
| Phellodendri chinensis Cortex | Isocorypalmine | Delta-type opioid receptor |
| Phellodendri chinensis Cortex | Isocorypalmine | 5-hydroxytryptamine 2A receptor |
| Phellodendri chinensis Cortex | Isocorypalmine | Sodium-dependent noradrenaline transporter |
| Phellodendri chinensis Cortex | Isocorypalmine | Alpha-1A adrenergic receptor |
| Phellodendri chinensis Cortex | Isocorypalmine | 5-hydroxytryptamine 2C receptor |
| Phellodendri chinensis Cortex | Isocorypalmine | Muscarinic acetylcholine receptor M2 |
| Phellodendri chinensis Cortex | Isocorypalmine | Alpha-2B adrenergic receptor |
| Phellodendri chinensis Cortex | Isocorypalmine | Alpha-1B adrenergic receptor |
| Phellodendri chinensis Cortex | Isocorypalmine | D(3) dopamine receptor |
| Phellodendri chinensis Cortex | Isocorypalmine | Sodium-dependent dopamine transporter |
| Phellodendri chinensis Cortex | Isocorypalmine | Beta-2 adrenergic receptor |
| Phellodendri chinensis Cortex | Isocorypalmine | Alpha-1D adrenergic receptor |
| Phellodendri chinensis Cortex | Isocorypalmine | Sodium-dependent serotonin transporter |
| Phellodendri chinensis Cortex | Isocorypalmine | Mu-type opioid receptor |
| Phellodendri chinensis Cortex | Isocorypalmine | Heat shock protein HSP 90 |
| Phellodendri chinensis Cortex | Isocorypalmine | mRNA of PKA Catalytic Subunit C-alpha |
| Phellodendri chinensis Cortex | Isocorypalmine | Retinoic acid receptor RXR-beta |
| Phellodendri chinensis Cortex | Isocorypalmine | Ig gamma-1 chain C region |
| Phellodendri chinensis Cortex | Isocorypalmine | Nuclear receptor coactivator 1 |
| Phellodendri chinensis Cortex | Isocorypalmine | Calmodulin |
| Phellodendri chinensis Cortex | Isocorypalmine | Calcium-activated potassium channel subunit alpha 1 |
| Phellodendri chinensis Cortex | beta-elemene | Prostaglandin G/H synthase 2 |
| Phellodendri chinensis Cortex | beta-elemene | Gamma-aminobutyric-acid receptor alpha-2 subunit |
| Phellodendri chinensis Cortex | beta-elemene | Retinoic acid receptor RXR-alpha |
| Phellodendri chinensis Cortex | beta-elemene | Sodium-dependent noradrenaline transporter |
| Phellodendri chinensis Cortex | beta-elemene | Gamma-aminobutyric-acid receptor alpha-3 subunit |
| Phellodendri chinensis Cortex | beta-elemene | Muscarinic acetylcholine receptor M2 |
| Phellodendri chinensis Cortex | beta-elemene | Gamma-aminobutyric acid receptor subunit alpha-1 |
| Phellodendri chinensis Cortex | beta-elemene | Gamma-aminobutyric-acid receptor subunit alpha-6 |
| Phellodendri chinensis Cortex | beta-elemene | Prostaglandin G/H synthase 1 |
| Phellodendri chinensis Cortex | beta-elemene | Muscarinic acetylcholine receptor M3 |
| Phellodendri chinensis Cortex | beta-elemene | Muscarinic acetylcholine receptor M1 |
| Phellodendri chinensis Cortex | beta-elemene | Alpha-1A adrenergic receptor |
| Phellodendri chinensis Cortex | beta-elemene | Neuronal acetylcholine receptor protein, alpha-7 chain |
| Phellodendri chinensis Cortex | beta-elemene | Nuclear receptor coactivator 2 |
| Phellodendri chinensis Cortex | beta-elemene | Gamma-aminobutyric-acid receptor alpha-5 subunit |
| Phellodendri chinensis Cortex | beta-elemene | Apoptosis regulator Bcl-2 |
| Phellodendri chinensis Cortex | beta-elemene | Cyclin-dependent kinase inhibitor 1 |
| Phellodendri chinensis Cortex | beta-elemene | Eukaryotic translation initiation factor 6 |
| Phellodendri chinensis Cortex | beta-elemene | Retinoblastoma-associated protein |
| Phellodendri chinensis Cortex | beta-elemene | Cellular tumor antigen p53 |
| Phellodendri chinensis Cortex | beta-elemene | Telomerase protein component 1 |
| Phellodendri chinensis Cortex | beta-elemene | Protein CBFA2T1 |
| Phellodendri chinensis Cortex | beta-elemene | Cell division control protein 2 homolog |
| Phellodendri chinensis Cortex | beta-elemene | G2/mitotic-specific cyclin-B1 |
| Phellodendri chinensis Cortex | beta-elemene | Transforming protein RhoA |
| Phellodendri chinensis Cortex | palmitic acid | Cathepsin D |
| Phellodendri chinensis Cortex | palmitic acid | Alcohol dehydrogenase 1B |
| Phellodendri chinensis Cortex | palmitic acid | Alcohol dehydrogenase 1C |
| Phellodendri chinensis Cortex | palmitic acid | Prostaglandin G/H synthase 1 |
| Phellodendri chinensis Cortex | palmitic acid | Prostaglandin G/H synthase 2 |
| Phellodendri chinensis Cortex | palmitic acid | Rhodopsin |
| Phellodendri chinensis Cortex | palmitic acid | Ig gamma-1 chain C region |
| Phellodendri chinensis Cortex | palmitic acid | Ferrichrome-iron receptor |
| Phellodendri chinensis Cortex | palmitic acid | Nuclear receptor coactivator 2 |
| Phellodendri chinensis Cortex | palmitic acid | Apoptosis regulator Bcl-2 |
| Phellodendri chinensis Cortex | palmitic acid | Interleukin-10 |
| Phellodendri chinensis Cortex | palmitic acid | Tumor necrosis factor |
| Phellodendri chinensis Cortex | palmitic acid | Collagen alpha-1(I) chain |
| Phellodendri chinensis Cortex | palmitic acid | Phosphatidylinositol-3,4,5-trisphosphate 3-phosphatase and dual-specificity protein phosphatase PTEN |
| Phellodendri chinensis Cortex | palmitic acid | Putative beta-glucuronidase-like protein SMA3 |
| Phellodendri chinensis Cortex | palmitic acid | Solute carrier family 22 member 5 |
| Phellodendri chinensis Cortex | palmitic acid | Choline-phosphate cytidylyltransferase A |
| Phellodendri chinensis Cortex | quercetin | Prostaglandin G/H synthase 1 |
| Phellodendri chinensis Cortex | quercetin | Androgen receptor |
| Phellodendri chinensis Cortex | quercetin | Peroxisome proliferator activated receptor gamma |
| Phellodendri chinensis Cortex | quercetin | Prostaglandin G/H synthase 2 |
| Phellodendri chinensis Cortex | quercetin | Heat shock protein HSP 90 |
| Phellodendri chinensis Cortex | quercetin | Phosphatidylinositol-4,5-bisphosphate 3-kinase catalytic subunit, gamma isoform |
| Phellodendri chinensis Cortex | quercetin | Nuclear receptor coactivator 2 |
| Phellodendri chinensis Cortex | quercetin | Dipeptidyl peptidase IV |
| Phellodendri chinensis Cortex | quercetin | Aldose reductase |
| Phellodendri chinensis Cortex | quercetin | Trypsin-1 |
| Phellodendri chinensis Cortex | quercetin | DNA topoisomerase II |
| Phellodendri chinensis Cortex | quercetin | Thrombin |
| Phellodendri chinensis Cortex | quercetin | Potassium voltage-gated channel subfamily H member 2 |
| Phellodendri chinensis Cortex | quercetin | Sodium channel protein type 5 subunit alpha |
| Phellodendri chinensis Cortex | quercetin | Coagulation factor Xa |
| Phellodendri chinensis Cortex | quercetin | Beta-2 adrenergic receptor |
| Phellodendri chinensis Cortex | quercetin | Stromelysin-1 |
| Phellodendri chinensis Cortex | quercetin | mRNA of PKA Catalytic Subunit C-alpha |
| Phellodendri chinensis Cortex | quercetin | Coagulation factor VII |
| Phellodendri chinensis Cortex | quercetin | Nitric-oxide synthase, endothelial |
| Phellodendri chinensis Cortex | quercetin | Retinoic acid receptor RXR-alpha |
| Phellodendri chinensis Cortex | quercetin | Acetylcholinesterase |
| Phellodendri chinensis Cortex | quercetin | Gamma-aminobutyric acid receptor subunit alpha-1 |
| Phellodendri chinensis Cortex | quercetin | Amine oxidase [flavin-containing] B |
| Phellodendri chinensis Cortex | quercetin | Transcription factor p65 |
| Phellodendri chinensis Cortex | quercetin | Epidermal growth factor receptor |
| Phellodendri chinensis Cortex | quercetin | RAC-alpha serine/threonine-protein kinase |
| Phellodendri chinensis Cortex | quercetin | Vascular endothelial growth factor A |
| Phellodendri chinensis Cortex | quercetin | G1/S-specific cyclin-D1 |
| Phellodendri chinensis Cortex | quercetin | Apoptosis regulator Bcl-2 |
| Phellodendri chinensis Cortex | quercetin | Bcl-2-like protein 1 |
| Phellodendri chinensis Cortex | quercetin | Proto-oncogene c-Fos |
| Phellodendri chinensis Cortex | quercetin | Cyclin-dependent kinase inhibitor 1 |
| Phellodendri chinensis Cortex | quercetin | Eukaryotic translation initiation factor 6 |
| Phellodendri chinensis Cortex | quercetin | Apoptosis regulator BAX |
| Phellodendri chinensis Cortex | quercetin | Caspase-9 |
| Phellodendri chinensis Cortex | quercetin | Urokinase-type plasminogen activator |
| Phellodendri chinensis Cortex | quercetin | 72 kDa type IV collagenase |
| Phellodendri chinensis Cortex | quercetin | Matrix metalloproteinase-9 |
| Phellodendri chinensis Cortex | quercetin | Mitogen-activated protein kinase 1 |
| Phellodendri chinensis Cortex | quercetin | Interleukin-10 |
| Phellodendri chinensis Cortex | quercetin | Pro-epidermal growth factor |
| Phellodendri chinensis Cortex | quercetin | Retinoblastoma-associated protein |
| Phellodendri chinensis Cortex | quercetin | Tumor necrosis factor |
| Phellodendri chinensis Cortex | quercetin | Transcription factor AP-1 |
| Phellodendri chinensis Cortex | quercetin | Interleukin-6 |
| Phellodendri chinensis Cortex | quercetin | Cyclin-dependent kinase inhibitor 2A, isoforms 1/2/3 |
| Phellodendri chinensis Cortex | quercetin | Activator of 90 kDa heat shock protein ATPase homolog 1 |
| Phellodendri chinensis Cortex | quercetin | Caspase-3 |
| Phellodendri chinensis Cortex | quercetin | Cellular tumor antigen p53 |
| Phellodendri chinensis Cortex | quercetin | ETS domain-containing protein Elk-1 |
| Phellodendri chinensis Cortex | quercetin | NF-kappa-B inhibitor alpha |
| Phellodendri chinensis Cortex | quercetin | NADPH--cytochrome P450 reductase |
| Phellodendri chinensis Cortex | quercetin | Ornithine decarboxylase |
| Phellodendri chinensis Cortex | quercetin | Xanthine dehydrogenase/oxidase |
| Phellodendri chinensis Cortex | quercetin | Caspase-8 |
| Phellodendri chinensis Cortex | quercetin | DNA topoisomerase 1 |
| Phellodendri chinensis Cortex | quercetin | RAF proto-oncogene serine/threonine-protein kinase |
| Phellodendri chinensis Cortex | quercetin | Superoxide dismutase [Cu-Zn] |
| Phellodendri chinensis Cortex | quercetin | Protein kinase C alpha type |
| Phellodendri chinensis Cortex | quercetin | Interstitial collagenase |
| Phellodendri chinensis Cortex | quercetin | Hypoxia-inducible factor 1-alpha |
| Phellodendri chinensis Cortex | quercetin | Signal transducer and activator of transcription 1-alpha/beta |
| Phellodendri chinensis Cortex | quercetin | Protein CBFA2T1 |
| Phellodendri chinensis Cortex | quercetin | Probable E3 ubiquitin-protein ligase HERC5 |
| Phellodendri chinensis Cortex | quercetin | Cell division control protein 2 homolog |
| Phellodendri chinensis Cortex | quercetin | 78 kDa glucose-regulated protein |
| Phellodendri chinensis Cortex | quercetin | Receptor tyrosine-protein kinase erbB-2 |
| Phellodendri chinensis Cortex | quercetin | Peroxisome proliferator-activated receptor gamma |
| Phellodendri chinensis Cortex | quercetin | Acetyl-CoA carboxylase 1 |
| Phellodendri chinensis Cortex | quercetin | Heme oxygenase 1 |
| Phellodendri chinensis Cortex | quercetin | Cytochrome P450 3A4 |
| Phellodendri chinensis Cortex | quercetin | Cytochrome P450 1A2 |
| Phellodendri chinensis Cortex | quercetin | Caveolin-1 |
| Phellodendri chinensis Cortex | quercetin | Myc proto-oncogene protein |
| Phellodendri chinensis Cortex | quercetin | Tissue factor |
| Phellodendri chinensis Cortex | quercetin | Gap junction alpha-1 protein |
| Phellodendri chinensis Cortex | quercetin | Cytochrome P450 1A1 |
| Phellodendri chinensis Cortex | quercetin | Intercellular adhesion molecule 1 |
| Phellodendri chinensis Cortex | quercetin | Interleukin-1 beta |
| Phellodendri chinensis Cortex | quercetin | C-C motif chemokine 2 |
| Phellodendri chinensis Cortex | quercetin | E-selectin |
| Phellodendri chinensis Cortex | quercetin | Vascular cell adhesion protein 1 |
| Phellodendri chinensis Cortex | quercetin | Prostaglandin E2 receptor EP3 subtype |
| Phellodendri chinensis Cortex | quercetin | Interleukin-8 |
| Phellodendri chinensis Cortex | quercetin | Protein kinase C beta type |
| Phellodendri chinensis Cortex | quercetin | Baculoviral IAP repeat-containing protein 5 |
| Phellodendri chinensis Cortex | quercetin | Dual oxidase 2 |
| Phellodendri chinensis Cortex | quercetin | Nitric oxide synthase, endothelial |
| Phellodendri chinensis Cortex | quercetin | Heat shock protein beta-1 |
| Phellodendri chinensis Cortex | quercetin | Transforming growth factor beta-1 |
| Phellodendri chinensis Cortex | quercetin | Estrogen sulfotransferase |
| Phellodendri chinensis Cortex | quercetin | Maltase-glucoamylase, intestinal |
| Phellodendri chinensis Cortex | quercetin | Interleukin-2 |
| Phellodendri chinensis Cortex | quercetin | Nuclear receptor subfamily 1 group I member 2 |
| Phellodendri chinensis Cortex | quercetin | Cytochrome P450 1B1 |
| Phellodendri chinensis Cortex | quercetin | G2/mitotic-specific cyclin-B1 |
| Phellodendri chinensis Cortex | quercetin | Tissue-type plasminogen activator |
| Phellodendri chinensis Cortex | quercetin | Thrombomodulin |
| Phellodendri chinensis Cortex | quercetin | Plasminogen activator inhibitor 1 |
| Phellodendri chinensis Cortex | quercetin | Collagen alpha-1(I) chain |
| Phellodendri chinensis Cortex | quercetin | Interferon gamma |
| Phellodendri chinensis Cortex | quercetin | Arachidonate 5-lipoxygenase |
| Phellodendri chinensis Cortex | quercetin | Phosphatidylinositol-3,4,5-trisphosphate 3-phosphatase and dual-specificity protein phosphatase PTEN |
| Phellodendri chinensis Cortex | quercetin | Interleukin-1 alpha |
| Phellodendri chinensis Cortex | quercetin | Myeloperoxidase |
| Phellodendri chinensis Cortex | quercetin | DNA topoisomerase 2-alpha |
| Phellodendri chinensis Cortex | quercetin | Neutrophil cytosol factor 1 |
| Phellodendri chinensis Cortex | quercetin | ATP-binding cassette sub-family G member 2 |
| Phellodendri chinensis Cortex | quercetin | Hyaluronan synthase 2 |
| Phellodendri chinensis Cortex | quercetin | Glutathione S-transferase P |
| Phellodendri chinensis Cortex | quercetin | Nuclear factor erythroid 2-related factor 2 |
| Phellodendri chinensis Cortex | quercetin | NAD(P)H dehydrogenase [quinone] 1 |
| Phellodendri chinensis Cortex | quercetin | Poly [ADP-ribose] polymerase 1 |
| Phellodendri chinensis Cortex | quercetin | Aryl hydrocarbon receptor |
| Phellodendri chinensis Cortex | quercetin | 26S proteasome non-ATPase regulatory subunit 3 |
| Phellodendri chinensis Cortex | quercetin | Solute carrier family 2, facilitated glucose transporter member 4 |
| Phellodendri chinensis Cortex | quercetin | Collagen alpha-1(III) chain |
| Phellodendri chinensis Cortex | quercetin | DNA gyrase subunit B |
| Phellodendri chinensis Cortex | quercetin | C-X-C motif chemokine 11 |
| Phellodendri chinensis Cortex | quercetin | C-X-C motif chemokine 2 |
| Phellodendri chinensis Cortex | quercetin | DDB1- and CUL4-associated factor 5 |
| Phellodendri chinensis Cortex | quercetin | Nuclear receptor subfamily 1 group I member 3 |
| Phellodendri chinensis Cortex | quercetin | Serine/threonine-protein kinase Chk2 |
| Phellodendri chinensis Cortex | quercetin | Insulin receptor |
| Phellodendri chinensis Cortex | quercetin | Claudin-4 |
| Phellodendri chinensis Cortex | quercetin | Peroxisome proliferator-activated receptor alpha |
| Phellodendri chinensis Cortex | quercetin | Peroxisome proliferator-activated receptor delta |
| Phellodendri chinensis Cortex | quercetin | Heat shock factor protein 1 |
| Phellodendri chinensis Cortex | quercetin | C-reactive protein |
| Phellodendri chinensis Cortex | quercetin | C-X-C motif chemokine 10 |
| Phellodendri chinensis Cortex | quercetin | Inhibitor of nuclear factor kappa-B kinase subunit alpha |
| Phellodendri chinensis Cortex | quercetin | Osteopontin |
| Phellodendri chinensis Cortex | quercetin | Runt-related transcription factor 2 |
| Phellodendri chinensis Cortex | quercetin | Ras association domain-containing protein 1 |
| Phellodendri chinensis Cortex | quercetin | Transcription factor E2F1 |
| Phellodendri chinensis Cortex | quercetin | Transcription factor E2F2 |
| Phellodendri chinensis Cortex | quercetin | Prostatic acid phosphatase |
| Phellodendri chinensis Cortex | quercetin | Cathepsin D |
| Phellodendri chinensis Cortex | quercetin | Insulin-like growth factor-binding protein 3 |
| Phellodendri chinensis Cortex | quercetin | Insulin-like growth factor II |
| Phellodendri chinensis Cortex | quercetin | CD40 ligand |
| Phellodendri chinensis Cortex | quercetin | Interferon regulatory factor 1 |
| Phellodendri chinensis Cortex | quercetin | Receptor tyrosine-protein kinase erbB-3 |
| Phellodendri chinensis Cortex | quercetin | Serum paraoxonase/arylesterase 1 |
| Phellodendri chinensis Cortex | quercetin | Type I iodothyronine deiodinase |
| Phellodendri chinensis Cortex | quercetin | Procollagen C-endopeptidase enhancer 1 |
| Phellodendri chinensis Cortex | quercetin | Puromycin-sensitive aminopeptidase |
| Phellodendri chinensis Cortex | quercetin | Hexokinase-2 |
| Phellodendri chinensis Cortex | quercetin | Homeobox protein Nkx-3.1 |
| Phellodendri chinensis Cortex | quercetin | Ras GTPase-activating protein 1 |
| Phellodendri chinensis Cortex | quercetin | Peroxidase C1A |
| Phellodendri chinensis Cortex | quercetin | Glutathione S-transferase Mu 1 |
| Phellodendri chinensis Cortex | quercetin | Glutathione S-transferase Mu 2 |
| Phellodendri chinensis Cortex | Undecenal | Alcohol dehydrogenase 1C |
| Phellodendri chinensis Cortex | Furol | Alcohol dehydrogenase 1B |
| Phellodendri chinensis Cortex | Furol | Alcohol dehydrogenase 1C |
| Phellodendri chinensis Cortex | Furol | Alcohol dehydrogenase 1A |
| Phellodendri chinensis Cortex | Furol | Lysozyme |
| Phellodendri chinensis Cortex | Furol | Nicotinate-nucleotide--dimethylbenzimidazole phosphoribosyltransferase |
| Phellodendri chinensis Cortex | Myrcene | Alcohol dehydrogenase 1C |
| Phellodendri chinensis Cortex | Myrcene | Gamma-aminobutyric acid receptor subunit alpha-1 |
| Phellodendri chinensis Cortex | eugenol | Prostaglandin G/H synthase 1 |
| Phellodendri chinensis Cortex | eugenol | Dopamine D1 receptor |
| Phellodendri chinensis Cortex | eugenol | Muscarinic acetylcholine receptor M3 |
| Phellodendri chinensis Cortex | eugenol | Muscarinic acetylcholine receptor M1 |
| Phellodendri chinensis Cortex | eugenol | Beta-1 adrenergic receptor |
| Phellodendri chinensis Cortex | eugenol | Prostaglandin G/H synthase 2 |
| Phellodendri chinensis Cortex | eugenol | Nitric-oxide synthase, endothelial |
| Phellodendri chinensis Cortex | eugenol | Alpha-2A adrenergic receptor |
| Phellodendri chinensis Cortex | eugenol | Alpha-2C adrenergic receptor |
| Phellodendri chinensis Cortex | eugenol | Sodium-dependent noradrenaline transporter |
| Phellodendri chinensis Cortex | eugenol | Alpha-1A adrenergic receptor |
| Phellodendri chinensis Cortex | eugenol | Muscarinic acetylcholine receptor M2 |
| Phellodendri chinensis Cortex | eugenol | Alpha-1B adrenergic receptor |
| Phellodendri chinensis Cortex | eugenol | Sodium-dependent dopamine transporter |
| Phellodendri chinensis Cortex | eugenol | Beta-2 adrenergic receptor |
| Phellodendri chinensis Cortex | eugenol | Beta-lactamase |
| Phellodendri chinensis Cortex | eugenol | Amine oxidase [flavin-containing] B |
| Phellodendri chinensis Cortex | eugenol | Amine oxidase [flavin-containing] A |
| Phellodendri chinensis Cortex | eugenol | Lysozyme |
| Phellodendri chinensis Cortex | eugenol | Chymotrypsinogen B |
| Phellodendri chinensis Cortex | eugenol | Alpha-1D adrenergic receptor |
| Phellodendri chinensis Cortex | eugenol | Leukotriene A-4 hydrolase |
| Phellodendri chinensis Cortex | eugenol | Alpha-2B adrenergic receptor |
| Phellodendri chinensis Cortex | eugenol | Urokinase-type plasminogen activator |
| Phellodendri chinensis Cortex | eugenol | Thermolysin |
| Phellodendri chinensis Cortex | eugenol | Sodium channel protein type 5 subunit alpha |
| Phellodendri chinensis Cortex | eugenol | Transcription factor p65 |
| Phellodendri chinensis Cortex | eugenol | Cytochrome P450 1A1 |
| Phellodendri chinensis Cortex | eugenol | Cytochrome P450 1B1 |
| Phellodendri chinensis Cortex | eugenol | Arachidonate 5-lipoxygenase |
| Phellodendri chinensis Cortex | eugenol | Aryl hydrocarbon receptor |
| Phellodendri chinensis Cortex | eugenol | Canalicular multispecific organic anion transporter 1 |
| Phellodendri chinensis Cortex | eugenol | T-lymphocyte activation antigen CD86 |
| Phellodendri chinensis Cortex | eugenol | Serine/threonine-protein phosphatase 2B catalytic subunit alpha isoform |
| Phellodendri chinensis Cortex | eugenol | Pepsin A |
| Phellodendri chinensis Cortex | eugenol | Mucin-1 |
| Phellodendri chinensis Cortex | eugenol | Quinone oxidoreductase |
| Phellodendri chinensis Cortex | eugenol | Calcium-transporting ATPase type 2C member 1 |
| Phellodendri chinensis Cortex | eugenol | Short transient receptor potential channel 3 |
| Phellodendri chinensis Cortex | eugenol | Transient receptor potential cation channel subfamily V member 3 |
| Phellodendri chinensis Cortex | eugenol | Ecto-NOX disulfide-thiol exchanger 2 |
| Phellodendri chinensis Cortex | caprylic acid | Phospholipase A2 |
| Phellodendri chinensis Cortex | caprylic acid | Alcohol dehydrogenase 1B |
| Phellodendri chinensis Cortex | caprylic acid | Alcohol dehydrogenase 1C |
| Phellodendri chinensis Cortex | caprylic acid | Acyl-CoA thioesterase I precursor |
| Phellodendri chinensis Cortex | caprylic acid | Trypsin-3 |
| Phellodendri chinensis Cortex | caprylic acid | Interleukin-8 |
| Phellodendri chinensis Cortex | caprylic acid | Peroxisome proliferator-activated receptor alpha |
| Phellodendri chinensis Cortex | caprylic acid | Histone acetyltransferase p300 |
| Phellodendri chinensis Cortex | caprylic acid | Fatty acid-binding protein, liver |
| Phellodendri chinensis Cortex | caprylic acid | Retinol-binding protein 2 |
| Phellodendri chinensis Cortex | Sitogluside | Progesterone receptor |
| Phellodendri chinensis Cortex | Sitogluside | Prostaglandin G/H synthase 1 |
| Phellodendri chinensis Cortex | Sitogluside | Muscarinic acetylcholine receptor M3 |
| Phellodendri chinensis Cortex | Sitogluside | Potassium voltage-gated channel subfamily H member 2 |
| Phellodendri chinensis Cortex | Sitogluside | Muscarinic acetylcholine receptor M1 |
| Phellodendri chinensis Cortex | Sitogluside | Sodium channel protein type 5 subunit alpha |
| Phellodendri chinensis Cortex | Sitogluside | Coagulation factor Xa |
| Phellodendri chinensis Cortex | Sitogluside | Prostaglandin G/H synthase 2 |
| Phellodendri chinensis Cortex | Sitogluside | 5-hydroxytryptamine receptor 3A |
| Phellodendri chinensis Cortex | Sitogluside | Retinoic acid receptor RXR-alpha |
| Phellodendri chinensis Cortex | Sitogluside | CGMP-inhibited 3',5'-cyclic phosphodiesterase A |
| Phellodendri chinensis Cortex | Sitogluside | Alpha-1B adrenergic receptor |
| Phellodendri chinensis Cortex | Sitogluside | Beta-2 adrenergic receptor |
| Phellodendri chinensis Cortex | Sitogluside | Alpha-1D adrenergic receptor |
| Phellodendri chinensis Cortex | Sitogluside | Heat shock protein HSP 90 |
| Phellodendri chinensis Cortex | Sitogluside | Nuclear receptor coactivator 2 |
| Phellodendri chinensis Cortex | Sitogluside | Calmodulin |
| Phellodendri chinensis Cortex | FERULIC ACID (CIS) | Prostaglandin G/H synthase 1 |
| Phellodendri chinensis Cortex | FERULIC ACID (CIS) | Prostaglandin G/H synthase 2 |
| Phellodendri chinensis Cortex | FERULIC ACID (CIS) | Nitric-oxide synthase, endothelial |
| Phellodendri chinensis Cortex | FERULIC ACID (CIS) | Alpha-2A adrenergic receptor |
| Phellodendri chinensis Cortex | FERULIC ACID (CIS) | Sodium-dependent noradrenaline transporter |
| Phellodendri chinensis Cortex | FERULIC ACID (CIS) | Alpha-2B adrenergic receptor |
| Phellodendri chinensis Cortex | FERULIC ACID (CIS) | Sodium-dependent dopamine transporter |
| Phellodendri chinensis Cortex | FERULIC ACID (CIS) | Beta-2 adrenergic receptor |
| Phellodendri chinensis Cortex | FERULIC ACID (CIS) | Leukotriene A-4 hydrolase |
| Phellodendri chinensis Cortex | FERULIC ACID (CIS) | Amine oxidase [flavin-containing] B |
| Phellodendri chinensis Cortex | FERULIC ACID (CIS) | Amine oxidase [flavin-containing] A |
| Phellodendri chinensis Cortex | FERULIC ACID (CIS) | mRNA of PKA Catalytic Subunit C-alpha |
| Phellodendri chinensis Cortex | FERULIC ACID (CIS) | Chymotrypsinogen B |
| Phellodendri chinensis Cortex | FERULIC ACID (CIS) | Muscarinic acetylcholine receptor M2 |
| Phellodendri chinensis Cortex | vanillin | Amine oxidase [flavin-containing] B |
| Phellodendri chinensis Cortex | vanillin | Amine oxidase [flavin-containing] A |
| Phellodendri chinensis Cortex | vanillin | Lysozyme |
| Phellodendri chinensis Cortex | vanillin | Nicotinate-nucleotide--dimethylbenzimidazole phosphoribosyltransferase |
| Phellodendri chinensis Cortex | vanillin | Prostaglandin G/H synthase 2 |
| Phellodendri chinensis Cortex | vanillin | Gamma-aminobutyric acid receptor subunit alpha-1 |
| Phellodendri chinensis Cortex | vanillin | Alcohol dehydrogenase 1C |
| Phellodendri chinensis Cortex | vanillin | Cytochrome P450-cam |
| Phellodendri chinensis Cortex | vanillin | Matrix metalloproteinase-9 |
| Phellodendri chinensis Cortex | vanillin | Mitogen-activated protein kinase 1 |
| Phellodendri chinensis Cortex | vanillin | Transcription factor AP-1 |
| Phellodendri chinensis Cortex | PENTYLFURAN | Sodium-dependent noradrenaline transporter |
| Phellodendri chinensis Cortex | PENTYLFURAN | Alpha-1A adrenergic receptor |
| Phellodendri chinensis Cortex | oleic acid | Prostaglandin G/H synthase 1 |
| Phellodendri chinensis Cortex | oleic acid | Nuclear receptor coactivator 2 |
| Phellodendri chinensis Cortex | oleic acid | Prostaglandin G/H synthase 2 |
| Phellodendri chinensis Cortex | oleic acid | Alcohol dehydrogenase 1B |
| Phellodendri chinensis Cortex | oleic acid | Alcohol dehydrogenase 1C |
| Phellodendri chinensis Cortex | oleic acid | Alcohol dehydrogenase 1A |
| Phellodendri chinensis Cortex | oleic acid | Lysozyme |
| Phellodendri chinensis Cortex | oleic acid | Nicotinate-nucleotide--dimethylbenzimidazole phosphoribosyltransferase |
| Phellodendri chinensis Cortex | oleic acid | Trypsin-3 |
| Phellodendri chinensis Cortex | oleic acid | Retinoic acid receptor RXR-alpha |
| Phellodendri chinensis Cortex | oleic acid | Cytochrome P450-cam |
| Phellodendri chinensis Cortex | oleic acid | Urokinase-type plasminogen activator |
| Phellodendri chinensis Cortex | oleic acid | Superoxide dismutase [Cu-Zn] |
| Phellodendri chinensis Cortex | oleic acid | Catalase |
| Phellodendri chinensis Cortex | oleic acid | Telomerase protein component 1 |
| Phellodendri chinensis Cortex | oleic acid | Endothelin-1 |
| Phellodendri chinensis Cortex | oleic acid | Receptor tyrosine-protein kinase erbB-2 |
| Phellodendri chinensis Cortex | oleic acid | Peroxisome proliferator-activated receptor gamma |
| Phellodendri chinensis Cortex | oleic acid | Lipoprotein lipase |
| Phellodendri chinensis Cortex | oleic acid | Neuromodulin |
| Phellodendri chinensis Cortex | oleic acid | Plasminogen activator inhibitor 1 |
| Phellodendri chinensis Cortex | oleic acid | Brain-derived neurotrophic factor |
| Phellodendri chinensis Cortex | oleic acid | 3-hydroxy-3-methylglutaryl-coenzyme A reductase |
| Phellodendri chinensis Cortex | oleic acid | Myeloperoxidase |
| Phellodendri chinensis Cortex | oleic acid | Peroxisome proliferator-activated receptor alpha |
| Phellodendri chinensis Cortex | oleic acid | Peroxisome proliferator-activated receptor delta |
| Phellodendri chinensis Cortex | oleic acid | C-reactive protein |
| Phellodendri chinensis Cortex | oleic acid | Serum paraoxonase/arylesterase 1 |
| Phellodendri chinensis Cortex | oleic acid | Insulin |
| Phellodendri chinensis Cortex | oleic acid | Plasminogen |
| Phellodendri chinensis Cortex | oleic acid | Fatty acid-binding protein, liver |
| Phellodendri chinensis Cortex | oleic acid | Retinol-binding protein 2 |
| Phellodendri chinensis Cortex | oleic acid | Glucagon |
| Phellodendri chinensis Cortex | oleic acid | Glutamyl aminopeptidase |
| Phellodendri chinensis Cortex | oleic acid | Mitochondrial uncoupling protein 2 |
| Phellodendri chinensis Cortex | oleic acid | Sterol O-acyltransferase 1 |
| Phellodendri chinensis Cortex | oleic acid | Cholecystokinin |
| Phellodendri chinensis Cortex | oleic acid | Cbp/p300-interacting transactivator 1 |
| Phellodendri chinensis Cortex | oleic acid | BDNF/NT-3 growth factors receptor |
| Phellodendri chinensis Cortex | oleic acid | Pancreas/duodenum homeobox protein 1 |
| Phellodendri chinensis Cortex | oleic acid | Solute carrier family 2, facilitated glucose transporter member 2 |
| Phellodendri chinensis Cortex | oleic acid | Peptidyl-glycine alpha-amidating monooxygenase |
| Phellodendri chinensis Cortex | oleic acid | Acyl-CoA desaturase |
| Phellodendri chinensis Cortex | oleic acid | Mitochondrial uncoupling protein 3 |
| Phellodendri chinensis Cortex | oleic acid | Cholesteryl ester transfer protein |
| Phellodendri chinensis Cortex | oleic acid | Peptide YY |
| Phellodendri chinensis Cortex | oleic acid | Aspartyl aminopeptidase |
| Phellodendri chinensis Cortex | oleic acid | Cell-death-related nuclease 7 |
| Phellodendri chinensis Cortex | WLN: VHR | Alcohol dehydrogenase 1B |
| Phellodendri chinensis Cortex | WLN: VHR | Alcohol dehydrogenase 1C |
| Phellodendri chinensis Cortex | WLN: VHR | Alcohol dehydrogenase 1A |
| Phellodendri chinensis Cortex | WLN: VHR | Lysozyme |
| Phellodendri chinensis Cortex | WLN: VHR | Nicotinate-nucleotide--dimethylbenzimidazole phosphoribosyltransferase |
| Phellodendri chinensis Cortex | WLN: VHR | Trypsin-3 |
| Phellodendri chinensis Cortex | trans-2-nonenal | Alcohol dehydrogenase 1B |
| Phellodendri chinensis Cortex | trans-2-nonenal | Alcohol dehydrogenase 1C |
| Phellodendri chinensis Cortex | trans-2-nonenal | Trypsin-3 |
| Phellodendri chinensis Cortex | trans-2-nonenal | Neutrophil elastase |
| Phellodendri chinensis Cortex | trans-2-nonenal | Lithostathine-1-alpha |
| Phellodendri chinensis Cortex | trans-2-nonenal | Membrane-associated guanylate kinase, WW and PDZ domain-containing protein 2 |
| Phellodendri chinensis Cortex | STOCK1N-14407 | Prostaglandin G/H synthase 1 |
| Phellodendri chinensis Cortex | STOCK1N-14407 | Dopamine D1 receptor |
| Phellodendri chinensis Cortex | STOCK1N-14407 | Muscarinic acetylcholine receptor M3 |
| Phellodendri chinensis Cortex | STOCK1N-14407 | Potassium voltage-gated channel subfamily H member 2 |
| Phellodendri chinensis Cortex | STOCK1N-14407 | Muscarinic acetylcholine receptor M1 |
| Phellodendri chinensis Cortex | STOCK1N-14407 | D(1B) dopamine receptor |
| Phellodendri chinensis Cortex | STOCK1N-14407 | Beta-1 adrenergic receptor |
| Phellodendri chinensis Cortex | STOCK1N-14407 | Sodium channel protein type 5 subunit alpha |
| Phellodendri chinensis Cortex | STOCK1N-14407 | Muscarinic acetylcholine receptor M5 |
| Phellodendri chinensis Cortex | STOCK1N-14407 | Prostaglandin G/H synthase 2 |
| Phellodendri chinensis Cortex | STOCK1N-14407 | Carbonic anhydrase II |
| Phellodendri chinensis Cortex | STOCK1N-14407 | Alpha-2C adrenergic receptor |
| Phellodendri chinensis Cortex | STOCK1N-14407 | Muscarinic acetylcholine receptor M4 |
| Phellodendri chinensis Cortex | STOCK1N-14407 | Delta-type opioid receptor |
| Phellodendri chinensis Cortex | STOCK1N-14407 | 5-hydroxytryptamine 2A receptor |
| Phellodendri chinensis Cortex | STOCK1N-14407 | Sodium-dependent noradrenaline transporter |
| Phellodendri chinensis Cortex | STOCK1N-14407 | 5-hydroxytryptamine 2C receptor |
| Phellodendri chinensis Cortex | STOCK1N-14407 | Muscarinic acetylcholine receptor M2 |
| Phellodendri chinensis Cortex | STOCK1N-14407 | Alpha-1B adrenergic receptor |
| Phellodendri chinensis Cortex | STOCK1N-14407 | Sodium-dependent dopamine transporter |
| Phellodendri chinensis Cortex | STOCK1N-14407 | Beta-2 adrenergic receptor |
| Phellodendri chinensis Cortex | STOCK1N-14407 | Alpha-1D adrenergic receptor |
| Phellodendri chinensis Cortex | STOCK1N-14407 | Sodium-dependent serotonin transporter |
| Phellodendri chinensis Cortex | STOCK1N-14407 | Mu-type opioid receptor |
| Phellodendri chinensis Cortex | STOCK1N-14407 | Heat shock protein HSP 90 |
| Phellodendri chinensis Cortex | STOCK1N-14407 | Retinoic acid receptor RXR-beta |
| Phellodendri chinensis Cortex | STOCK1N-14407 | Calmodulin |
| Phellodendri chinensis Cortex | STOCK1N-14407 | Coagulation factor Xa |
| Phellodendri chinensis Cortex | STOCK1N-14407 | 5-hydroxytryptamine receptor 3A |
| Phellodendri chinensis Cortex | STOCK1N-14407 | Retinoic acid receptor RXR-alpha |
| Phellodendri chinensis Cortex | STOCK1N-14407 | D(3) dopamine receptor |
| Phellodendri chinensis Cortex | jatrorrizine | Nitric oxide synthase, inducible |
| Phellodendri chinensis Cortex | jatrorrizine | Prostaglandin G/H synthase 1 |
| Phellodendri chinensis Cortex | jatrorrizine | Potassium voltage-gated channel subfamily H member 2 |
| Phellodendri chinensis Cortex | jatrorrizine | Estrogen receptor |
| Phellodendri chinensis Cortex | jatrorrizine | Androgen receptor |
| Phellodendri chinensis Cortex | jatrorrizine | Sodium channel protein type 5 subunit alpha |
| Phellodendri chinensis Cortex | jatrorrizine | Prostaglandin G/H synthase 2 |
| Phellodendri chinensis Cortex | jatrorrizine | Nitric-oxide synthase, endothelial |
| Phellodendri chinensis Cortex | jatrorrizine | Retinoic acid receptor RXR-alpha |
| Phellodendri chinensis Cortex | jatrorrizine | Estrogen receptor beta |
| Phellodendri chinensis Cortex | jatrorrizine | Heat shock protein HSP 90 |
| Phellodendri chinensis Cortex | jatrorrizine | Cell division protein kinase 2 |
| Phellodendri chinensis Cortex | jatrorrizine | Trypsin-1 |
| Phellodendri chinensis Cortex | jatrorrizine | Proto-oncogene serine/threonine-protein kinase Pim-1 |
| Phellodendri chinensis Cortex | jatrorrizine | Cyclin-A2 |
| Phellodendri chinensis Cortex | jatrorrizine | Nuclear receptor coactivator 2 |
| Phellodendri chinensis Cortex | jatrorrizine | Calmodulin |
| Phellodendri chinensis Cortex | jatrorrizine | Calcium-activated potassium channel subunit alpha 1 |
| Phellodendri chinensis Cortex | jatrorrizine | DNA topoisomerase II |
| Phellodendri chinensis Cortex | jatrorrizine | mRNA of PKA Catalytic Subunit C-alpha |
| Phellodendri chinensis Cortex | menisperine | Prostaglandin G/H synthase 1 |
| Phellodendri chinensis Cortex | menisperine | Muscarinic acetylcholine receptor M3 |
| Phellodendri chinensis Cortex | menisperine | Potassium voltage-gated channel subfamily H member 2 |
| Phellodendri chinensis Cortex | menisperine | Androgen receptor |
| Phellodendri chinensis Cortex | menisperine | CGMP-specific 3',5'-cyclic phosphodiesterase |
| Phellodendri chinensis Cortex | menisperine | Sodium channel protein type 5 subunit alpha |
| Phellodendri chinensis Cortex | menisperine | Coagulation factor Xa |
| Phellodendri chinensis Cortex | menisperine | Prostaglandin G/H synthase 2 |
| Phellodendri chinensis Cortex | menisperine | Nitric-oxide synthase, endothelial |
| Phellodendri chinensis Cortex | menisperine | Retinoic acid receptor RXR-alpha |
| Phellodendri chinensis Cortex | menisperine | Delta-type opioid receptor |
| Phellodendri chinensis Cortex | menisperine | Acetylcholinesterase |
| Phellodendri chinensis Cortex | menisperine | Beta-2 adrenergic receptor |
| Phellodendri chinensis Cortex | menisperine | Alpha-1D adrenergic receptor |
| Phellodendri chinensis Cortex | menisperine | DNA topoisomerase II |
| Phellodendri chinensis Cortex | menisperine | Mu-type opioid receptor |
| Phellodendri chinensis Cortex | menisperine | Pregnane X receptor |
| Phellodendri chinensis Cortex | menisperine | Heat shock protein HSP 90 |
| Phellodendri chinensis Cortex | menisperine | Phosphatidylinositol-4,5-bisphosphate 3-kinase catalytic subunit, gamma isoform |
| Phellodendri chinensis Cortex | menisperine | Trypsin-1 |
| Phellodendri chinensis Cortex | menisperine | Nuclear receptor coactivator 2 |
| Phellodendri chinensis Cortex | menisperine | Nuclear receptor coactivator 1 |
| Phellodendri chinensis Cortex | menisperine | Calcium-activated potassium channel subunit alpha 1 |
| Phellodendri chinensis Cortex | menisperine | Calmodulin |
| Phellodendri chinensis Cortex | menisperine | Muscarinic acetylcholine receptor M1 |
| Phellodendri chinensis Cortex | menisperine | Alpha-1B adrenergic receptor |
| Phellodendri chinensis Cortex | menisperine | Retinoic acid receptor RXR-beta |
| Phellodendri chinensis Cortex | stearic acid | Prostaglandin G/H synthase 1 |
| Phellodendri chinensis Cortex | stearic acid | Prostaglandin G/H synthase 2 |
| Phellodendri chinensis Cortex | stearic acid | Retinoic acid receptor RXR-alpha |
| Phellodendri chinensis Cortex | stearic acid | Nuclear receptor coactivator 2 |
| Phellodendri chinensis Cortex | stearic acid | Ig gamma-1 chain C region |
| Phellodendri chinensis Cortex | stearic acid | Transcription factor Sp1 |
| Phellodendri chinensis Cortex | stearic acid | Ectonucleotide pyrophosphatase/phosphodiesterase family member 7 |
| Phellodendri chinensis Cortex | paeonol | Prostaglandin G/H synthase 1 |
| Phellodendri chinensis Cortex | paeonol | Beta-1 adrenergic receptor |
| Phellodendri chinensis Cortex | paeonol | Prostaglandin G/H synthase 2 |
| Phellodendri chinensis Cortex | paeonol | Alpha-2A adrenergic receptor |
| Phellodendri chinensis Cortex | paeonol | Alpha-2C adrenergic receptor |
| Phellodendri chinensis Cortex | paeonol | Sodium-dependent noradrenaline transporter |
| Phellodendri chinensis Cortex | paeonol | Alpha-2B adrenergic receptor |
| Phellodendri chinensis Cortex | paeonol | Alpha-1B adrenergic receptor |
| Phellodendri chinensis Cortex | paeonol | Sodium-dependent dopamine transporter |
| Phellodendri chinensis Cortex | paeonol | Beta-2 adrenergic receptor |
| Phellodendri chinensis Cortex | paeonol | Alpha-1D adrenergic receptor |
| Phellodendri chinensis Cortex | paeonol | Beta-lactamase |
| Phellodendri chinensis Cortex | paeonol | Amine oxidase [flavin-containing] B |
| Phellodendri chinensis Cortex | paeonol | Amine oxidase [flavin-containing] A |
| Phellodendri chinensis Cortex | paeonol | Chymotrypsinogen B |
| Phellodendri chinensis Cortex | paeonol | Alpha-1A adrenergic receptor |
| Phellodendri chinensis Cortex | paeonol | Muscarinic acetylcholine receptor M1 |
| Phellodendri chinensis Cortex | paeonol | Muscarinic acetylcholine receptor M2 |
| Phellodendri chinensis Cortex | paeonol | Transcription factor p65 |
| Phellodendri chinensis Cortex | paeonol | RAC-alpha serine/threonine-protein kinase |
| Phellodendri chinensis Cortex | paeonol | Apoptosis regulator Bcl-2 |
| Phellodendri chinensis Cortex | paeonol | Apoptosis regulator BAX |
| Phellodendri chinensis Cortex | paeonol | Mitogen-activated protein kinase 1 |
| Phellodendri chinensis Cortex | paeonol | Tumor necrosis factor |
| Phellodendri chinensis Cortex | paeonol | Activator of 90 kDa heat shock protein ATPase homolog 1 |
| Phellodendri chinensis Cortex | paeonol | NF-kappa-B inhibitor alpha |
| Phellodendri chinensis Cortex | paeonol | Intercellular adhesion molecule 1 |
| Phellodendri chinensis Cortex | paeonol | Interleukin-2 |
| Phellodendri chinensis Cortex | paeonol | Tyrosinase |
| Phellodendri chinensis Cortex | paeonol | Phosphatidylinositol-3,4,5-trisphosphate 3-phosphatase and dual-specificity protein phosphatase PTEN |
| Phellodendri chinensis Cortex | methyl palmitate | Prostaglandin G/H synthase 1 |
| Phellodendri chinensis Cortex | methyl palmitate | Nuclear receptor coactivator 2 |
| Phellodendri chinensis Cortex | methyl palmitate | Ig gamma-1 chain C region |
| Phellodendri chinensis Cortex | methyl palmitate | Transcription factor p65 |
| Phellodendri chinensis Cortex | methyl palmitate | Interleukin-10 |
| Phellodendri chinensis Cortex | methyl palmitate | Tumor necrosis factor |
| Phellodendri chinensis Cortex | methyl palmitate | Interleukin-6 |
| Phellodendri chinensis Cortex | methyl palmitate | Prostaglandin G/H synthase 2 |
| Phellodendri chinensis Cortex | methyl palmitate | Prostaglandin E2 receptor EP3 subtype |
| Phellodendri chinensis Cortex | Mnk | Alcohol dehydrogenase 1C |
| Phellodendri chinensis Cortex | Mnk | Ig gamma-1 chain C region |
| Phellodendri chinensis Cortex | phellamurin_qt | Estrogen receptor |
| Phellodendri chinensis Cortex | phellamurin_qt | Coagulation factor Xa |
| Phellodendri chinensis Cortex | phellamurin_qt | Prostaglandin G/H synthase 2 |
| Phellodendri chinensis Cortex | phellamurin_qt | Coagulation factor VII |
| Phellodendri chinensis Cortex | phellamurin_qt | CGMP-inhibited 3',5'-cyclic phosphodiesterase A |
| Phellodendri chinensis Cortex | phellamurin_qt | DNA topoisomerase II |
| Phellodendri chinensis Cortex | phellamurin_qt | Heat shock protein HSP 90 |
| Phellodendri chinensis Cortex | phellamurin_qt | Nuclear receptor coactivator 2 |
| Phellodendri chinensis Cortex | phellamurin_qt | Calmodulin |
| Phellodendri chinensis Cortex | phellamurin_qt | Glucocorticoid receptor |
| Phellodendri chinensis Cortex | Oxophorone | Muscarinic acetylcholine receptor M3 |
| Phellodendri chinensis Cortex | Oxophorone | Muscarinic acetylcholine receptor M1 |
| Phellodendri chinensis Cortex | Oxophorone | Beta-1 adrenergic receptor |
| Phellodendri chinensis Cortex | Oxophorone | Retinoic acid receptor RXR-alpha |
| Phellodendri chinensis Cortex | Oxophorone | 5-hydroxytryptamine 2A receptor |
| Phellodendri chinensis Cortex | Oxophorone | Sodium-dependent noradrenaline transporter |
| Phellodendri chinensis Cortex | Oxophorone | Alpha-1A adrenergic receptor |
| Phellodendri chinensis Cortex | Oxophorone | Gamma-aminobutyric-acid receptor alpha-3 subunit |
| Phellodendri chinensis Cortex | Oxophorone | Muscarinic acetylcholine receptor M2 |
| Phellodendri chinensis Cortex | Oxophorone | Alpha-1B adrenergic receptor |
| Phellodendri chinensis Cortex | Oxophorone | Sodium-dependent dopamine transporter |
| Phellodendri chinensis Cortex | Oxophorone | Beta-2 adrenergic receptor |
| Phellodendri chinensis Cortex | Oxophorone | Gamma-aminobutyric acid receptor subunit alpha-1 |
| Phellodendri chinensis Cortex | Oxophorone | Neuronal acetylcholine receptor protein, alpha-7 chain |
| Phellodendri chinensis Cortex | PENTADECYLIC ACID | Prostaglandin G/H synthase 1 |
| Phellodendri chinensis Cortex | PENTADECYLIC ACID | Rhodopsin |
| Phellodendri chinensis Cortex | PENTADECYLIC ACID | Ig gamma-1 chain C region |
| Phellodendri chinensis Cortex | PENTADECYLIC ACID | Ferrichrome-iron receptor |
| Phellodendri chinensis Cortex | PENTADECYLIC ACID | Nuclear receptor coactivator 2 |
| Phellodendri chinensis Cortex | PENTADECYLIC ACID | Prostaglandin G/H synthase 2 |
| Phellodendri chinensis Cortex | (S)-Canadine | Prostaglandin G/H synthase 1 |
| Phellodendri chinensis Cortex | (S)-Canadine | Dopamine D1 receptor |
| Phellodendri chinensis Cortex | (S)-Canadine | Muscarinic acetylcholine receptor M3 |
| Phellodendri chinensis Cortex | (S)-Canadine | Potassium voltage-gated channel subfamily H member 2 |
| Phellodendri chinensis Cortex | (S)-Canadine | Muscarinic acetylcholine receptor M1 |
| Phellodendri chinensis Cortex | (S)-Canadine | Sodium channel protein type 5 subunit alpha |
| Phellodendri chinensis Cortex | (S)-Canadine | Coagulation factor Xa |
| Phellodendri chinensis Cortex | (S)-Canadine | Muscarinic acetylcholine receptor M5 |
| Phellodendri chinensis Cortex | (S)-Canadine | Prostaglandin G/H synthase 2 |
| Phellodendri chinensis Cortex | (S)-Canadine | 5-hydroxytryptamine receptor 3A |
| Phellodendri chinensis Cortex | (S)-Canadine | Coagulation factor VII |
| Phellodendri chinensis Cortex | (S)-Canadine | Alpha-2C adrenergic receptor |
| Phellodendri chinensis Cortex | (S)-Canadine | Muscarinic acetylcholine receptor M4 |
| Phellodendri chinensis Cortex | (S)-Canadine | Retinoic acid receptor RXR-alpha |
| Phellodendri chinensis Cortex | (S)-Canadine | Delta-type opioid receptor |
| Phellodendri chinensis Cortex | (S)-Canadine | 5-hydroxytryptamine 2A receptor |
| Phellodendri chinensis Cortex | (S)-Canadine | Alpha-1A adrenergic receptor |
| Phellodendri chinensis Cortex | (S)-Canadine | 5-hydroxytryptamine 2C receptor |
| Phellodendri chinensis Cortex | (S)-Canadine | Muscarinic acetylcholine receptor M2 |
| Phellodendri chinensis Cortex | (S)-Canadine | Alpha-2B adrenergic receptor |
| Phellodendri chinensis Cortex | (S)-Canadine | Alpha-1B adrenergic receptor |
| Phellodendri chinensis Cortex | (S)-Canadine | D(3) dopamine receptor |
| Phellodendri chinensis Cortex | (S)-Canadine | Sodium-dependent dopamine transporter |
| Phellodendri chinensis Cortex | (S)-Canadine | Beta-2 adrenergic receptor |
| Phellodendri chinensis Cortex | (S)-Canadine | Alpha-1D adrenergic receptor |
| Phellodendri chinensis Cortex | (S)-Canadine | Sodium-dependent serotonin transporter |
| Phellodendri chinensis Cortex | (S)-Canadine | Mu-type opioid receptor |
| Phellodendri chinensis Cortex | (S)-Canadine | Heat shock protein HSP 90 |
| Phellodendri chinensis Cortex | (S)-Canadine | mRNA of PKA Catalytic Subunit C-alpha |
| Phellodendri chinensis Cortex | (S)-Canadine | Calmodulin |
| Phellodendri chinensis Cortex | (S)-Canadine | D(1B) dopamine receptor |
| Phellodendri chinensis Cortex | (S)-Canadine | Retinoic acid receptor RXR-beta |
| Phellodendri chinensis Cortex | columbamine | Nitric oxide synthase, inducible |
| Phellodendri chinensis Cortex | columbamine | Prostaglandin G/H synthase 1 |
| Phellodendri chinensis Cortex | columbamine | Potassium voltage-gated channel subfamily H member 2 |
| Phellodendri chinensis Cortex | columbamine | Androgen receptor |
| Phellodendri chinensis Cortex | columbamine | Sodium channel protein type 5 subunit alpha |
| Phellodendri chinensis Cortex | columbamine | Coagulation factor Xa |
| Phellodendri chinensis Cortex | columbamine | Prostaglandin G/H synthase 2 |
| Phellodendri chinensis Cortex | columbamine | Nitric-oxide synthase, endothelial |
| Phellodendri chinensis Cortex | columbamine | Coagulation factor VII |
| Phellodendri chinensis Cortex | columbamine | Heat shock protein HSP 90 |
| Phellodendri chinensis Cortex | columbamine | mRNA of PKA Catalytic Subunit C-alpha |
| Phellodendri chinensis Cortex | columbamine | Trypsin-1 |
| Phellodendri chinensis Cortex | columbamine | Nuclear receptor coactivator 2 |
| Phellodendri chinensis Cortex | columbamine | Nuclear receptor coactivator 1 |
| Phellodendri chinensis Cortex | columbamine | Calmodulin |
| Phellodendri chinensis Cortex | columbamine | Retinoic acid receptor RXR-alpha |
| Phellodendri chinensis Cortex | columbamine | Beta-2 adrenergic receptor |
| Phellodendri chinensis Cortex | columbamine | Beta-secretase |
| Phellodendri chinensis Cortex | columbamine | Pregnane X receptor |
| Phellodendri chinensis Cortex | columbamine | Calcium-activated potassium channel subunit alpha 1 |
| Phellodendri chinensis Cortex | columbamine | cAMP and cAMP-inhibited cGMP 3',5'-cyclic phosphodiesterase 10A |
| Phellodendri chinensis Cortex | EUG | Muscarinic acetylcholine receptor M1 |
| Phellodendri chinensis Cortex | EUG | Beta-1 adrenergic receptor |
| Phellodendri chinensis Cortex | EUG | Alpha-2C adrenergic receptor |
| Phellodendri chinensis Cortex | EUG | Sodium-dependent noradrenaline transporter |
| Phellodendri chinensis Cortex | EUG | Muscarinic acetylcholine receptor M2 |
| Phellodendri chinensis Cortex | EUG | Alpha-1B adrenergic receptor |
| Phellodendri chinensis Cortex | EUG | Sodium-dependent dopamine transporter |
| Phellodendri chinensis Cortex | EUG | Amine oxidase [flavin-containing] A |
| Phellodendri chinensis Cortex | EUG | Lysozyme |
| Phellodendri chinensis Cortex | EUG | Chymotrypsinogen B |
| Phellodendri chinensis Cortex | EUG | Alpha-1A adrenergic receptor |
| Phellodendri chinensis Cortex | EUG | Amine oxidase [flavin-containing] B |
| Phellodendri chinensis Cortex | poriferast-5-en-3beta-ol | Progesterone receptor |
| Phellodendri chinensis Cortex | poriferast-5-en-3beta-ol | Nuclear receptor coactivator 2 |
| Phellodendri chinensis Cortex | Isovanillin | Prostaglandin G/H synthase 1 |
| Phellodendri chinensis Cortex | Isovanillin | Prostaglandin G/H synthase 2 |
| Phellodendri chinensis Cortex | Isovanillin | Amine oxidase [flavin-containing] B |
| Phellodendri chinensis Cortex | Isovanillin | Amine oxidase [flavin-containing] A |
| Phellodendri chinensis Cortex | Isovanillin | Lysozyme |
| Phellodendri chinensis Cortex | Isovanillin | Chymotrypsinogen B |
| Phellodendri chinensis Cortex | Methyl 3-furoate | Alcohol dehydrogenase 1C |
| Phellodendri chinensis Cortex | Methyl 3-furoate | Nicotinate-nucleotide--dimethylbenzimidazole phosphoribosyltransferase |
| Phellodendri chinensis Cortex | N-Methylflindersine | Nitric oxide synthase, inducible |
| Phellodendri chinensis Cortex | N-Methylflindersine | Prostaglandin G/H synthase 1 |
| Phellodendri chinensis Cortex | N-Methylflindersine | Dopamine D1 receptor |
| Phellodendri chinensis Cortex | N-Methylflindersine | Muscarinic acetylcholine receptor M3 |
| Phellodendri chinensis Cortex | N-Methylflindersine | Muscarinic acetylcholine receptor M1 |
| Phellodendri chinensis Cortex | N-Methylflindersine | Sodium channel protein type 5 subunit alpha |
| Phellodendri chinensis Cortex | N-Methylflindersine | Muscarinic acetylcholine receptor M5 |
| Phellodendri chinensis Cortex | N-Methylflindersine | Prostaglandin G/H synthase 2 |
| Phellodendri chinensis Cortex | N-Methylflindersine | Nitric-oxide synthase, endothelial |
| Phellodendri chinensis Cortex | N-Methylflindersine | Muscarinic acetylcholine receptor M4 |
| Phellodendri chinensis Cortex | N-Methylflindersine | Retinoic acid receptor RXR-alpha |
| Phellodendri chinensis Cortex | N-Methylflindersine | CGMP-inhibited 3',5'-cyclic phosphodiesterase A |
| Phellodendri chinensis Cortex | N-Methylflindersine | Sodium-dependent noradrenaline transporter |
| Phellodendri chinensis Cortex | N-Methylflindersine | Muscarinic acetylcholine receptor M2 |
| Phellodendri chinensis Cortex | N-Methylflindersine | Alpha-1B adrenergic receptor |
| Phellodendri chinensis Cortex | N-Methylflindersine | Sodium-dependent dopamine transporter |
| Phellodendri chinensis Cortex | N-Methylflindersine | Beta-2 adrenergic receptor |
| Phellodendri chinensis Cortex | N-Methylflindersine | Sodium-dependent serotonin transporter |
| Phellodendri chinensis Cortex | N-Methylflindersine | Gamma-aminobutyric acid receptor subunit alpha-1 |
| Phellodendri chinensis Cortex | N-Methylflindersine | Heat shock protein HSP 90 |
| Phellodendri chinensis Cortex | N-Methylflindersine | Neuronal acetylcholine receptor protein, alpha-7 chain |
| Phellodendri chinensis Cortex | N-Methylflindersine | Alpha-1A adrenergic receptor |
| Phellodendri chinensis Cortex | Homocresol | Muscarinic acetylcholine receptor M1 |
| Phellodendri chinensis Cortex | Homocresol | Beta-1 adrenergic receptor |
| Phellodendri chinensis Cortex | Homocresol | Alpha-2A adrenergic receptor |
| Phellodendri chinensis Cortex | Homocresol | Alpha-2C adrenergic receptor |
| Phellodendri chinensis Cortex | Homocresol | Sodium-dependent noradrenaline transporter |
| Phellodendri chinensis Cortex | Homocresol | Alpha-1A adrenergic receptor |
| Phellodendri chinensis Cortex | Homocresol | Muscarinic acetylcholine receptor M2 |
| Phellodendri chinensis Cortex | Homocresol | Alpha-1B adrenergic receptor |
| Phellodendri chinensis Cortex | Homocresol | Sodium-dependent dopamine transporter |
| Phellodendri chinensis Cortex | Homocresol | Beta-2 adrenergic receptor |
| Phellodendri chinensis Cortex | Homocresol | Amine oxidase [flavin-containing] A |
| Phellodendri chinensis Cortex | Homocresol | Lysozyme |
| Phellodendri chinensis Cortex | beta-Rhodinol | Alcohol dehydrogenase 1C |
| Phellodendri chinensis Cortex | Phlorol | Alcohol dehydrogenase 1C |
| Phellodendri chinensis Cortex | Phlorol | Lysozyme |
| Phellodendri chinensis Cortex | Phlorol | Nicotinate-nucleotide--dimethylbenzimidazole phosphoribosyltransferase |
| Phellodendri chinensis Cortex | Phlorol | Alcohol dehydrogenase 1B |
| Phellodendri chinensis Cortex | magnoflorine | Prostaglandin G/H synthase 1 |
| Phellodendri chinensis Cortex | magnoflorine | Potassium voltage-gated channel subfamily H member 2 |
| Phellodendri chinensis Cortex | magnoflorine | Muscarinic acetylcholine receptor M1 |
| Phellodendri chinensis Cortex | magnoflorine | Androgen receptor |
| Phellodendri chinensis Cortex | magnoflorine | Sodium channel protein type 5 subunit alpha |
| Phellodendri chinensis Cortex | magnoflorine | Prostaglandin G/H synthase 2 |
| Phellodendri chinensis Cortex | magnoflorine | Carbonic anhydrase II |
| Phellodendri chinensis Cortex | magnoflorine | Retinoic acid receptor RXR-alpha |
| Phellodendri chinensis Cortex | magnoflorine | Acetylcholinesterase |
| Phellodendri chinensis Cortex | magnoflorine | Alpha-1B adrenergic receptor |
| Phellodendri chinensis Cortex | magnoflorine | Beta-2 adrenergic receptor |
| Phellodendri chinensis Cortex | magnoflorine | Alpha-1D adrenergic receptor |
| Phellodendri chinensis Cortex | magnoflorine | DNA topoisomerase II |
| Phellodendri chinensis Cortex | magnoflorine | Pregnane X receptor |
| Phellodendri chinensis Cortex | magnoflorine | Trypsin-1 |
| Phellodendri chinensis Cortex | magnoflorine | Nuclear receptor coactivator 2 |
| Phellodendri chinensis Cortex | magnoflorine | Nuclear receptor coactivator 1 |
| Phellodendri chinensis Cortex | magnoflorine | Calmodulin |
| Phellodendri chinensis Cortex | magnoflorine | Muscarinic acetylcholine receptor M3 |
| Phellodendri chinensis Cortex | magnoflorine | Nitric-oxide synthase, endothelial |
| Phellodendri chinensis Cortex | magnoflorine | Heat shock protein HSP 90 |
| Phellodendri chinensis Cortex | magnoflorine | Calcium-activated potassium channel subunit alpha 1 |
| Phellodendri chinensis Cortex | magnoflorine | Delta-type opioid receptor |
| Phellodendri chinensis Cortex | magnoflorine | Mu-type opioid receptor |
| Phellodendri chinensis Cortex | berberrubine | Nitric oxide synthase, inducible |
| Phellodendri chinensis Cortex | berberrubine | Prostaglandin G/H synthase 1 |
| Phellodendri chinensis Cortex | berberrubine | Potassium voltage-gated channel subfamily H member 2 |
| Phellodendri chinensis Cortex | berberrubine | Estrogen receptor |
| Phellodendri chinensis Cortex | berberrubine | Androgen receptor |
| Phellodendri chinensis Cortex | berberrubine | Sodium channel protein type 5 subunit alpha |
| Phellodendri chinensis Cortex | berberrubine | Prostaglandin G/H synthase 2 |
| Phellodendri chinensis Cortex | berberrubine | Nitric-oxide synthase, endothelial |
| Phellodendri chinensis Cortex | berberrubine | Retinoic acid receptor RXR-alpha |
| Phellodendri chinensis Cortex | berberrubine | mRNA of PKA Catalytic Subunit C-alpha |
| Phellodendri chinensis Cortex | berberrubine | Trypsin-1 |
| Phellodendri chinensis Cortex | berberrubine | Nuclear receptor coactivator 2 |
| Phellodendri chinensis Cortex | berberrubine | Calmodulin |
| Phellodendri chinensis Cortex | Noroxyhydrastinine | Prostaglandin G/H synthase 1 |
| Phellodendri chinensis Cortex | Noroxyhydrastinine | Muscarinic acetylcholine receptor M1 |
| Phellodendri chinensis Cortex | Noroxyhydrastinine | Prostaglandin G/H synthase 2 |
| Phellodendri chinensis Cortex | Noroxyhydrastinine | Alpha-2A adrenergic receptor |
| Phellodendri chinensis Cortex | Noroxyhydrastinine | CGMP-inhibited 3',5'-cyclic phosphodiesterase A |
| Phellodendri chinensis Cortex | Noroxyhydrastinine | Beta-2 adrenergic receptor |
| Phellodendri chinensis Cortex | Noroxyhydrastinine | Amine oxidase [flavin-containing] B |
| Phellodendri chinensis Cortex | Noroxyhydrastinine | Queuine tRNA-ribosyltransferase |
| Phellodendri chinensis Cortex | phellodendrine | Prostaglandin G/H synthase 1 |
| Phellodendri chinensis Cortex | phellodendrine | Muscarinic acetylcholine receptor M3 |
| Phellodendri chinensis Cortex | phellodendrine | Potassium voltage-gated channel subfamily H member 2 |
| Phellodendri chinensis Cortex | phellodendrine | Muscarinic acetylcholine receptor M1 |
| Phellodendri chinensis Cortex | phellodendrine | Sodium channel protein type 5 subunit alpha |
| Phellodendri chinensis Cortex | phellodendrine | Muscarinic acetylcholine receptor M5 |
| Phellodendri chinensis Cortex | phellodendrine | Prostaglandin G/H synthase 2 |
| Phellodendri chinensis Cortex | phellodendrine | Carbonic anhydrase II |
| Phellodendri chinensis Cortex | phellodendrine | Muscarinic acetylcholine receptor M4 |
| Phellodendri chinensis Cortex | phellodendrine | Alpha-1B adrenergic receptor |
| Phellodendri chinensis Cortex | phellodendrine | Beta-2 adrenergic receptor |
| Phellodendri chinensis Cortex | phellodendrine | Mu-type opioid receptor |
| Phellodendri chinensis Cortex | phellodendrine | Heat shock protein HSP 90 |
| Phellodendri chinensis Cortex | phellodendrine | Ig gamma-1 chain C region |
| Phellodendri chinensis Cortex | phellodendrine | Nuclear receptor coactivator 2 |
| Phellodendri chinensis Cortex | phellodendrine | Nuclear receptor coactivator 1 |
| Phellodendri chinensis Cortex | phellodendrine | Calmodulin |
| Phellodendri chinensis Cortex | Ethyl caffeate | Prostaglandin G/H synthase 1 |
| Phellodendri chinensis Cortex | Ethyl caffeate | Prostaglandin G/H synthase 2 |
| Phellodendri chinensis Cortex | Ethyl caffeate | Beta-2 adrenergic receptor |
| Phellodendri chinensis Cortex | Ethyl caffeate | Amine oxidase [flavin-containing] B |
| Phellodendri chinensis Cortex | Guasol | Alcohol dehydrogenase 1C |
| Phellodendri chinensis Cortex | Guasol | Lysozyme |
| Phellodendri chinensis Cortex | Guasol | Bacillolysin |
| Phellodendri chinensis Cortex | Guasol | Nicotinate-nucleotide--dimethylbenzimidazole phosphoribosyltransferase |
| Phellodendri chinensis Cortex | nonanoic acid | Phospholipase A2 |
| Phellodendri chinensis Cortex | nonanoic acid | Alcohol dehydrogenase 1B |
| Phellodendri chinensis Cortex | nonanoic acid | Alcohol dehydrogenase 1C |
| Phellodendri chinensis Cortex | nonanoic acid | Cathepsin D |
| Phellodendri chinensis Cortex | nonanoic acid | Inositol-3-phosphate synthase 1 |
| Phellodendri chinensis Cortex | naphthalene | Apoptosis regulator Bcl-2 |
| Phellodendri chinensis Cortex | naphthalene | Proto-oncogene c-Fos |
| Phellodendri chinensis Cortex | naphthalene | Eukaryotic translation initiation factor 6 |
| Phellodendri chinensis Cortex | naphthalene | Transcription factor AP-1 |
| Phellodendri chinensis Cortex | naphthalene | RAF proto-oncogene serine/threonine-protein kinase |
| Phellodendri chinensis Cortex | naphthalene | Cytochrome P450 1A1 |
| Phellodendri chinensis Cortex | naphthalene | Interleukin-8 |
| Phellodendri chinensis Cortex | naphthalene | Myeloperoxidase |
| Phellodendri chinensis Cortex | naphthalene | Ubiquitin carboxyl-terminal hydrolase isozyme L1 |
| Phellodendri chinensis Cortex | naphthalene | Peroxiredoxin-5, mitochondrial |
| Phellodendri chinensis Cortex | naphthalene | RNA-binding protein FUS |
| Phellodendri chinensis Cortex | limonin | Cytochrome P450 3A4 |
| Phellodendri chinensis Cortex | 5-METHYLFURFURAL | Alcohol dehydrogenase 1B |
| Phellodendri chinensis Cortex | 5-METHYLFURFURAL | Alcohol dehydrogenase 1C |
| Phellodendri chinensis Cortex | 5-METHYLFURFURAL | Alcohol dehydrogenase 1A |
| Phellodendri chinensis Cortex | 5-METHYLFURFURAL | Nicotinate-nucleotide--dimethylbenzimidazole phosphoribosyltransferase |
| Phellodendri chinensis Cortex | Hyperin | DNA topoisomerase II |
| Phellodendri chinensis Cortex | Hyperin | mRNA of Protein-tyrosine phosphatase, non-receptor type 1 |
| Phellodendri chinensis Cortex | Hyperin | Trypsin-1 |
| Phellodendri chinensis Cortex | Hyperin | Nitric oxide synthase, inducible |
| Phellodendri chinensis Cortex | Hyperin | Prostaglandin G/H synthase 1 |
| Phellodendri chinensis Cortex | Hyperin | Prostaglandin G/H synthase 2 |
| Phellodendri chinensis Cortex | Hyperin | Heat shock protein HSP 90 |
| Phellodendri chinensis Cortex | Hyperin | Phosphatidylinositol-4,5-bisphosphate 3-kinase catalytic subunit, gamma isoform |
| Phellodendri chinensis Cortex | Hyperin | Nuclear receptor coactivator 2 |
| Phellodendri chinensis Cortex | Maruzen M | Alcohol dehydrogenase 1B |
| Phellodendri chinensis Cortex | Maruzen M | Alcohol dehydrogenase 1C |
| Phellodendri chinensis Cortex | Maruzen M | Nicotinate-nucleotide--dimethylbenzimidazole phosphoribosyltransferase |
| Phellodendri chinensis Cortex | o-cresol | Alcohol dehydrogenase 1B |
| Phellodendri chinensis Cortex | o-cresol | Alcohol dehydrogenase 1C |
| Phellodendri chinensis Cortex | o-cresol | Alcohol dehydrogenase 1A |
| Phellodendri chinensis Cortex | o-cresol | Lysozyme |
| Phellodendri chinensis Cortex | o-cresol | Nicotinate-nucleotide--dimethylbenzimidazole phosphoribosyltransferase |
| Phellodendri chinensis Cortex | o-cresol | Prostaglandin G/H synthase 2 |
| Phellodendri chinensis Cortex | o-cresol | Prostaglandin G/H synthase 1 |
| Phellodendri chinensis Cortex | CREOSOL | Alcohol dehydrogenase 1C |
| Phellodendri chinensis Cortex | CREOSOL | Lysozyme |
| Phellodendri chinensis Cortex | CREOSOL | Nicotinate-nucleotide--dimethylbenzimidazole phosphoribosyltransferase |
| Phellodendri chinensis Cortex | Methyl naphthalene | Amine oxidase [flavin-containing] B |
| Phellodendri chinensis Cortex | Methyl naphthalene | Lysozyme |
| Phellodendri chinensis Cortex | campesterol | Progesterone receptor |
| Phellodendri chinensis Cortex | isoferulic acid | Prostaglandin G/H synthase 1 |
| Phellodendri chinensis Cortex | isoferulic acid | Beta-1 adrenergic receptor |
| Phellodendri chinensis Cortex | isoferulic acid | Prostaglandin G/H synthase 2 |
| Phellodendri chinensis Cortex | isoferulic acid | Nitric-oxide synthase, endothelial |
| Phellodendri chinensis Cortex | isoferulic acid | Alpha-2A adrenergic receptor |
| Phellodendri chinensis Cortex | isoferulic acid | Alpha-2C adrenergic receptor |
| Phellodendri chinensis Cortex | isoferulic acid | Sodium-dependent dopamine transporter |
| Phellodendri chinensis Cortex | isoferulic acid | Beta-2 adrenergic receptor |
| Phellodendri chinensis Cortex | isoferulic acid | Leukotriene A-4 hydrolase |
| Phellodendri chinensis Cortex | isoferulic acid | Amine oxidase [flavin-containing] B |
| Phellodendri chinensis Cortex | isoferulic acid | Amine oxidase [flavin-containing] A |
| Phellodendri chinensis Cortex | isoferulic acid | Alpha-1A adrenergic receptor |
| Phellodendri chinensis Cortex | Methyl caffeate | Prostaglandin G/H synthase 1 |
| Phellodendri chinensis Cortex | Methyl caffeate | Prostaglandin G/H synthase 2 |
| Phellodendri chinensis Cortex | Methyl caffeate | Sodium-dependent dopamine transporter |
| Phellodendri chinensis Cortex | Methyl caffeate | Beta-2 adrenergic receptor |
| Phellodendri chinensis Cortex | Methyl caffeate | Leukotriene A-4 hydrolase |
| Phellodendri chinensis Cortex | Methyl caffeate | Amine oxidase [flavin-containing] B |
| Phellodendri chinensis Cortex | Methyl caffeate | Amine oxidase [flavin-containing] A |
| Phellodendri chinensis Cortex | Clorius | Lysozyme |
| Phellodendri chinensis Cortex | Clorius | Nicotinate-nucleotide--dimethylbenzimidazole phosphoribosyltransferase |
| Phellodendri chinensis Cortex | Clorius | Alcohol dehydrogenase 1C |
| Phellodendri chinensis Cortex | Ptelein | Prostaglandin G/H synthase 2 |
| Phellodendri chinensis Cortex | Ptelein | Retinoic acid receptor RXR-alpha |
| Phellodendri chinensis Cortex | Ptelein | Heat shock protein HSP 90 |
| Phellodendri chinensis Cortex | Ptelein | Leukotriene A-4 hydrolase |
| Phellodendri chinensis Cortex | Ptelein | Amine oxidase [flavin-containing] B |
| Phellodendri chinensis Cortex | Ptelein | mRNA of PKA Catalytic Subunit C-alpha |
| Phellodendri chinensis Cortex | 4-[(1R,3aS,4R,6aS)-4-(4-hydroxy-3,5-dimethoxyphenyl)-1,3,3a,4,6,6a-hexahydrofuro[4,3-c]furan-1-yl]-2,6-dimethoxyphenol | Potassium voltage-gated channel subfamily H member 2 |
| Phellodendri chinensis Cortex | 4-[(1R,3aS,4R,6aS)-4-(4-hydroxy-3,5-dimethoxyphenyl)-1,3,3a,4,6,6a-hexahydrofuro[4,3-c]furan-1-yl]-2,6-dimethoxyphenol | Coagulation factor Xa |
| Phellodendri chinensis Cortex | 4-[(1R,3aS,4R,6aS)-4-(4-hydroxy-3,5-dimethoxyphenyl)-1,3,3a,4,6,6a-hexahydrofuro[4,3-c]furan-1-yl]-2,6-dimethoxyphenol | Prostaglandin G/H synthase 2 |
| Phellodendri chinensis Cortex | 4-[(1R,3aS,4R,6aS)-4-(4-hydroxy-3,5-dimethoxyphenyl)-1,3,3a,4,6,6a-hexahydrofuro[4,3-c]furan-1-yl]-2,6-dimethoxyphenol | DNA topoisomerase II |
| Phellodendri chinensis Cortex | 4-[(1R,3aS,4R,6aS)-4-(4-hydroxy-3,5-dimethoxyphenyl)-1,3,3a,4,6,6a-hexahydrofuro[4,3-c]furan-1-yl]-2,6-dimethoxyphenol | Nuclear receptor coactivator 2 |
| Phellodendri chinensis Cortex | 4-[(1R,3aS,4R,6aS)-4-(4-hydroxy-3,5-dimethoxyphenyl)-1,3,3a,4,6,6a-hexahydrofuro[4,3-c]furan-1-yl]-2,6-dimethoxyphenol | Calmodulin |
| Phellodendri chinensis Cortex | guanidine | Integrin alpha-6 |
| Phellodendri chinensis Cortex | 7-hydroxy-6-(2-hydroxyethyl)coumarin | Prostaglandin G/H synthase 1 |
| Phellodendri chinensis Cortex | 7-hydroxy-6-(2-hydroxyethyl)coumarin | Prostaglandin G/H synthase 2 |
| Phellodendri chinensis Cortex | 7-hydroxy-6-(2-hydroxyethyl)coumarin | Alpha-2A adrenergic receptor |
| Phellodendri chinensis Cortex | 7-hydroxy-6-(2-hydroxyethyl)coumarin | Alpha-2B adrenergic receptor |
| Phellodendri chinensis Cortex | 7-hydroxy-6-(2-hydroxyethyl)coumarin | mRNA of Protein-tyrosine phosphatase, non-receptor type 1 |
| Phellodendri chinensis Cortex | 7-hydroxy-6-(2-hydroxyethyl)coumarin | Beta-2 adrenergic receptor |
| Phellodendri chinensis Cortex | 7-hydroxy-6-(2-hydroxyethyl)coumarin | Amine oxidase [flavin-containing] B |
| Phellodendri chinensis Cortex | 7-hydroxy-6-(2-hydroxyethyl)coumarin | mRNA of PKA Catalytic Subunit C-alpha |
| Phellodendri chinensis Cortex | 7-hydroxy-6-(2-hydroxyethyl)coumarin | cAMP-dependent protein kinase inhibitor alpha |
| Phellodendri chinensis Cortex | thalifendine | Nitric oxide synthase, inducible |
| Phellodendri chinensis Cortex | thalifendine | Prostaglandin G/H synthase 1 |
| Phellodendri chinensis Cortex | thalifendine | Potassium voltage-gated channel subfamily H member 2 |
| Phellodendri chinensis Cortex | thalifendine | Estrogen receptor |
| Phellodendri chinensis Cortex | thalifendine | Androgen receptor |
| Phellodendri chinensis Cortex | thalifendine | Sodium channel protein type 5 subunit alpha |
| Phellodendri chinensis Cortex | thalifendine | Prostaglandin G/H synthase 2 |
| Phellodendri chinensis Cortex | thalifendine | Nitric-oxide synthase, endothelial |
| Phellodendri chinensis Cortex | thalifendine | Retinoic acid receptor RXR-alpha |
| Phellodendri chinensis Cortex | thalifendine | mRNA of PKA Catalytic Subunit C-alpha |
| Phellodendri chinensis Cortex | thalifendine | Trypsin-1 |
| Phellodendri chinensis Cortex | thalifendine | Nuclear receptor coactivator 2 |
| Phellodendri chinensis Cortex | thalifendine | Calmodulin |
| Phellodendri chinensis Cortex | thalifendine | Heat shock protein HSP 90 |
| Phellodendri chinensis Cortex | Furfuranol | Alcohol dehydrogenase 1B |
| Phellodendri chinensis Cortex | Furfuranol | Alcohol dehydrogenase 1C |
| Phellodendri chinensis Cortex | Furfuranol | Alcohol dehydrogenase 1A |
| Phellodendri chinensis Cortex | Furfuranol | Lysozyme |
| Phellodendri chinensis Cortex | Furfuranol | Nicotinate-nucleotide--dimethylbenzimidazole phosphoribosyltransferase |
| Phellodendri chinensis Cortex | (S)-4-Nonanolide | Prostaglandin G/H synthase 2 |
| Phellodendri chinensis Cortex | (S)-4-Nonanolide | Sodium-dependent noradrenaline transporter |
| Phellodendri chinensis Cortex | (S)-4-Nonanolide | Gamma-aminobutyric acid receptor subunit alpha-1 |
| Phellodendri chinensis Cortex | Methyl atratate | Prostaglandin G/H synthase 1 |
| Phellodendri chinensis Cortex | Methyl atratate | Prostaglandin G/H synthase 2 |
| Phellodendri chinensis Cortex | Methyl atratate | Gamma-aminobutyric-acid receptor alpha-2 subunit |
| Phellodendri chinensis Cortex | Methyl atratate | Gamma-aminobutyric-acid receptor alpha-3 subunit |
| Phellodendri chinensis Cortex | Methyl atratate | Alpha-1B adrenergic receptor |
| Phellodendri chinensis Cortex | Methyl atratate | Sodium-dependent dopamine transporter |
| Phellodendri chinensis Cortex | Methyl atratate | Beta-2 adrenergic receptor |
| Phellodendri chinensis Cortex | Methyl atratate | Gamma-aminobutyric acid receptor subunit alpha-1 |
| Phellodendri chinensis Cortex | Methyl atratate | Amine oxidase [flavin-containing] B |
| Phellodendri chinensis Cortex | Methyl atratate | Glutamate receptor 2 |
| Phellodendri chinensis Cortex | Acetylfuran | Alcohol dehydrogenase 1C |
| Phellodendri chinensis Cortex | Acetylfuran | Lysozyme |
| Phellodendri chinensis Cortex | Acetylfuran | Bacillolysin |
| Phellodendri chinensis Cortex | Acetylfuran | Nicotinate-nucleotide--dimethylbenzimidazole phosphoribosyltransferase |
| Phellodendri chinensis Cortex | candicine | Sodium-dependent noradrenaline transporter |
| Phellodendri chinensis Cortex | candicine | Sodium-dependent dopamine transporter |
| Phellodendri chinensis Cortex | candicine | Sodium-dependent serotonin transporter |
| Phellodendri chinensis Cortex | 2-undecenoic acid | Alcohol dehydrogenase 1C |
| Phellodendri chinensis Cortex | Homoveratrole | Muscarinic acetylcholine receptor M1 |
| Phellodendri chinensis Cortex | Homoveratrole | Alpha-2C adrenergic receptor |
| Phellodendri chinensis Cortex | Homoveratrole | Sodium-dependent noradrenaline transporter |
| Phellodendri chinensis Cortex | Homoveratrole | Alpha-1B adrenergic receptor |
| Phellodendri chinensis Cortex | Homoveratrole | Lysozyme |
| Phellodendri chinensis Cortex | Auraptene | Muscarinic acetylcholine receptor M1 |
| Phellodendri chinensis Cortex | Auraptene | Sodium channel protein type 5 subunit alpha |
| Phellodendri chinensis Cortex | Auraptene | Prostaglandin G/H synthase 2 |
| Phellodendri chinensis Cortex | Auraptene | Carbonic anhydrase II |
| Phellodendri chinensis Cortex | Auraptene | Retinoic acid receptor RXR-alpha |
| Phellodendri chinensis Cortex | Auraptene | CGMP-inhibited 3',5'-cyclic phosphodiesterase A |
| Phellodendri chinensis Cortex | Auraptene | Alpha-1B adrenergic receptor |
| Phellodendri chinensis Cortex | Auraptene | Beta-2 adrenergic receptor |
| Phellodendri chinensis Cortex | Auraptene | Leukotriene A-4 hydrolase |
| Gardenia Fructus | crocetin | Cytochrome P450-cam |
| Gardenia Fructus | crocetin | Muscarinic acetylcholine receptor M3 |
| Gardenia Fructus | crocetin | Muscarinic acetylcholine receptor M1 |
| Gardenia Fructus | crocetin | Gamma-aminobutyric-acid receptor alpha-2 subunit |
| Gardenia Fructus | crocetin | Gamma-aminobutyric-acid receptor alpha-5 subunit |
| Gardenia Fructus | crocetin | Alpha-1A adrenergic receptor |
| Gardenia Fructus | crocetin | Gamma-aminobutyric-acid receptor alpha-3 subunit |
| Gardenia Fructus | crocetin | Muscarinic acetylcholine receptor M2 |
| Gardenia Fructus | crocetin | Alpha-1B adrenergic receptor |
| Gardenia Fructus | crocetin | Gamma-aminobutyric acid receptor subunit alpha-1 |
| Gardenia Fructus | crocetin | Ig gamma-1 chain C region |
| Gardenia Fructus | crocetin | Prostaglandin G/H synthase 2 |
| Gardenia Fructus | crocetin | Nuclear receptor coactivator 2 |
| Gardenia Fructus | crocetin | Vascular cell adhesion protein 1 |
| Gardenia Fructus | genipin | Prostaglandin G/H synthase 1 |
| Gardenia Fructus | genipin | Prostaglandin G/H synthase 2 |
| Gardenia Fructus | genipin | Carbonic anhydrase II |
| Gardenia Fructus | genipin | Gamma-aminobutyric-acid receptor alpha-2 subunit |
| Gardenia Fructus | genipin | Gamma-aminobutyric acid receptor subunit alpha-1 |
| Gardenia Fructus | genipin | Thymidine kinase |
| Gardenia Fructus | genipin | Trypsin-1 |
| Gardenia Fructus | genipin | Glutamate receptor 2 |
| Gardenia Fructus | genipin | Gamma-aminobutyric-acid receptor subunit alpha-6 |
| Gardenia Fructus | genipin | Canalicular multispecific organic anion transporter 1 |
| Gardenia Fructus | Ammidin | Thrombin |
| Gardenia Fructus | Ammidin | Muscarinic acetylcholine receptor M1 |
| Gardenia Fructus | Ammidin | Prostaglandin G/H synthase 2 |
| Gardenia Fructus | Ammidin | Gamma-aminobutyric acid receptor subunit alpha-1 |
| Gardenia Fructus | Ammidin | Dipeptidyl peptidase IV |
| Gardenia Fructus | Ammidin | Phosphatidylinositol-4,5-bisphosphate 3-kinase catalytic subunit, gamma isoform |
| Gardenia Fructus | Ammidin | mRNA of PKA Catalytic Subunit C-alpha |
| Gardenia Fructus | Ammidin | Amine oxidase [flavin-containing] B |
| Gardenia Fructus | Hirsutrin | DNA topoisomerase II |
| Gardenia Fructus | Hirsutrin | mRNA of Protein-tyrosine phosphatase, non-receptor type 1 |
| Gardenia Fructus | Hirsutrin | Coagulation factor Xa |
| Gardenia Fructus | crocin | Sphingomyelin phosphodiesterase 2 |
| Gardenia Fructus | geniposide | Carbonic anhydrase II |
| Gardenia Fructus | geniposide | Apoptosis regulator Bcl-2 |
| Gardenia Fructus | geniposide | Heme oxygenase 1 |
| Gardenia Fructus | geniposide | Neuromodulin |
| Gardenia Fructus | geniposide | Phospholipase B1, membrane-associated |
| Gardenia Fructus | geniposide | Glucagon |
| Gardenia Fructus | geniposide | Glutathione S-transferase Mu 1 |
| Gardenia Fructus | geniposide | Glutathione S-transferase Mu 2 |
| Gardenia Fructus | rutin | DNA topoisomerase II |
| Gardenia Fructus | rutin | Transcription factor p65 |
| Gardenia Fructus | rutin | Tumor necrosis factor |
| Gardenia Fructus | rutin | Interleukin-6 |
| Gardenia Fructus | rutin | Caspase-3 |
| Gardenia Fructus | rutin | NADPH--cytochrome P450 reductase |
| Gardenia Fructus | rutin | Superoxide dismutase [Cu-Zn] |
| Gardenia Fructus | rutin | Catalase |
| Gardenia Fructus | rutin | Interleukin-1 beta |
| Gardenia Fructus | rutin | Interleukin-8 |
| Gardenia Fructus | rutin | Protein kinase C beta type |
| Gardenia Fructus | rutin | Arachidonate 5-lipoxygenase |
| Gardenia Fructus | rutin | 3-hydroxy-3-methylglutaryl-coenzyme A reductase |
| Gardenia Fructus | rutin | Hyaluronan synthase 2 |
| Gardenia Fructus | rutin | Glutathione S-transferase P |
| Gardenia Fructus | rutin | Type I iodothyronine deiodinase |
| Gardenia Fructus | rutin | C5a anaphylatoxin chemotactic receptor |
| Gardenia Fructus | rutin | Insulin |
| Gardenia Fructus | rutin | Low affinity immunoglobulin epsilon Fc receptor |
| Gardenia Fructus | rutin | Integrin beta-2 |
| Gardenia Fructus | rutin | Thromboxane A2 receptor |
| Gardenia Fructus | (1S,4aS,5R,7S,7aS)-5,7-dihydroxy-7-methyl-1-[(2S,3R,4S,5S,6R)-3,4,5-trihydroxy-6-(hydroxymethyl)oxan-2-yl]oxy-4a,5,6,7a-tetrahydro-1H-cyclopenta[d]pyran-4-carboxylic acid | Carbonic anhydrase II |
| Gardenia Fructus | (1S,4aS,5R,7S,7aS)-5,7-dihydroxy-7-methyl-1-[(2S,3R,4S,5S,6R)-3,4,5-trihydroxy-6-(hydroxymethyl)oxan-2-yl]oxy-4a,5,6,7a-tetrahydro-1H-cyclopenta[d]pyran-4-carboxylic acid | Dipeptidyl peptidase IV |
| Gardenia Fructus | SHANZHISIDE_qt | Trypsin-1 |
| Gardenia Fructus | SHANZHISIDE_qt | Glutamate receptor 2 |
| Gardenia Fructus | Sudan III | Thrombin |
| Gardenia Fructus | Sudan III | Estrogen receptor |
| Gardenia Fructus | Sudan III | Prostaglandin G/H synthase 2 |
| Gardenia Fructus | Sudan III | Coagulation factor VII |
| Gardenia Fructus | Sudan III | Estrogen receptor beta |
| Gardenia Fructus | Sudan III | Dipeptidyl peptidase IV |
| Gardenia Fructus | Sudan III | Mitogen-activated protein kinase 14 |
| Gardenia Fructus | Sudan III | Glycogen synthase kinase-3 beta |
| Gardenia Fructus | Sudan III | Mitogen-activated protein kinase 10 |
| Gardenia Fructus | Sudan III | Cell division protein kinase 2 |
| Gardenia Fructus | Sudan III | mRNA of PKA Catalytic Subunit C-alpha |
| Gardenia Fructus | Sudan III | Proto-oncogene serine/threonine-protein kinase Pim-1 |
| Gardenia Fructus | Sudan III | Cyclin-A2 |
| Gardenia Fructus | ursolic acid | Urokinase-type plasminogen activator |
| Gardenia Fructus | ursolic acid | Cathepsin B |
| Gardenia Fructus | ursolic acid | Transcription factor p65 |
| Gardenia Fructus | ursolic acid | Signal transducer and activator of transcription 3 |
| Gardenia Fructus | ursolic acid | Vascular endothelial growth factor A |
| Gardenia Fructus | ursolic acid | G1/S-specific cyclin-D1 |
| Gardenia Fructus | ursolic acid | Apoptosis regulator Bcl-2 |
| Gardenia Fructus | ursolic acid | Bcl-2-like protein 1 |
| Gardenia Fructus | ursolic acid | Proto-oncogene c-Fos |
| Gardenia Fructus | ursolic acid | Cyclin-dependent kinase inhibitor 1 |
| Gardenia Fructus | ursolic acid | Apoptosis regulator BAX |
| Gardenia Fructus | ursolic acid | Caspase-9 |
| Gardenia Fructus | ursolic acid | 72 kDa type IV collagenase |
| Gardenia Fructus | ursolic acid | Matrix metalloproteinase-9 |
| Gardenia Fructus | ursolic acid | Cell division protein kinase 4 |
| Gardenia Fructus | ursolic acid | Tumor necrosis factor |
| Gardenia Fructus | ursolic acid | Transcription factor AP-1 |
| Gardenia Fructus | ursolic acid | Interleukin-6 |
| Gardenia Fructus | ursolic acid | Cell division protein kinase 6 |
| Gardenia Fructus | ursolic acid | Caspase-3 |
| Gardenia Fructus | ursolic acid | Cellular tumor antigen p53 |
| Gardenia Fructus | ursolic acid | Mitogen-activated protein kinase 8 |
| Gardenia Fructus | ursolic acid | Prostaglandin G/H synthase 2 |
| Gardenia Fructus | ursolic acid | NF-kappa-B inhibitor alpha |
| Gardenia Fructus | ursolic acid | Caspase-8 |
| Gardenia Fructus | ursolic acid | Fatty acid synthase |
| Gardenia Fructus | ursolic acid | Interstitial collagenase |
| Gardenia Fructus | ursolic acid | Stromelysin-1 |
| Gardenia Fructus | ursolic acid | Probable E3 ubiquitin-protein ligase HERC5 |
| Gardenia Fructus | ursolic acid | Heparin-binding growth factor 2 |
| Gardenia Fructus | ursolic acid | Stromelysin-2 |
| Gardenia Fructus | ursolic acid | Intercellular adhesion molecule 1 |
| Gardenia Fructus | ursolic acid | Interleukin-1 beta |
| Gardenia Fructus | ursolic acid | Cyclic AMP-responsive element-binding protein 1 |
| Gardenia Fructus | ursolic acid | E-selectin |
| Gardenia Fructus | ursolic acid | Prostaglandin E2 receptor EP3 subtype |
| Gardenia Fructus | ursolic acid | Prostaglandin G/H synthase 1 |
| Gardenia Fructus | ursolic acid | Induced myeloid leukemia cell differentiation protein Mcl-1 |
| Gardenia Fructus | ursolic acid | Protein kinase C gamma type |
| Gardenia Fructus | ursolic acid | Cyclic AMP-dependent transcription factor ATF-2 |
| Gardenia Fructus | ursolic acid | Granulocyte-macrophage colony-stimulating factor |
| Gardenia Fructus | ursolic acid | Platelet endothelial cell adhesion molecule |
| Gardenia Fructus | ursolic acid | C-Jun-amino-terminal kinase-interacting protein 2 |
| Gardenia Fructus | ursolic acid | Baculoviral IAP repeat-containing protein 5 |
| Gardenia Fructus | ursolic acid | Tyrosine-protein phosphatase non-receptor type 6 |
| Gardenia Fructus | ursolic acid | Neuromodulin |
| Gardenia Fructus | ursolic acid | Dual oxidase 2 |
| Gardenia Fructus | ursolic acid | Nitric oxide synthase, endothelial |
| Gardenia Fructus | ursolic acid | Tyrosine-protein phosphatase non-receptor type 1 |
| Gardenia Fructus | ursolic acid | Phosphatidylinositol-3,4,5-trisphosphate 5-phosphatase 2 |
| Gardenia Fructus | ursolic acid | Lipopolysaccharide-induced tumor necrosis factor-alpha factor |
| Gardenia Fructus | ursolic acid | G1/S-specific cyclin-D2 |
| Gardenia Fructus | ursolic acid | Tumor necrosis factor ligand superfamily member 6 |
| Gardenia Fructus | ursolic acid | Caspase-1 |
| Gardenia Fructus | ursolic acid | Ectonucleotide pyrophosphatase/phosphodiesterase family member 7 |
| Gardenia Fructus | quercetin | Prostaglandin G/H synthase 1 |
| Gardenia Fructus | quercetin | Androgen receptor |
| Gardenia Fructus | quercetin | Peroxisome proliferator activated receptor gamma |
| Gardenia Fructus | quercetin | Prostaglandin G/H synthase 2 |
| Gardenia Fructus | quercetin | Heat shock protein HSP 90 |
| Gardenia Fructus | quercetin | Phosphatidylinositol-4,5-bisphosphate 3-kinase catalytic subunit, gamma isoform |
| Gardenia Fructus | quercetin | Nuclear receptor coactivator 2 |
| Gardenia Fructus | quercetin | Dipeptidyl peptidase IV |
| Gardenia Fructus | quercetin | Aldose reductase |
| Gardenia Fructus | quercetin | Trypsin-1 |
| Gardenia Fructus | quercetin | DNA topoisomerase II |
| Gardenia Fructus | quercetin | Thrombin |
| Gardenia Fructus | quercetin | Potassium voltage-gated channel subfamily H member 2 |
| Gardenia Fructus | quercetin | Sodium channel protein type 5 subunit alpha |
| Gardenia Fructus | quercetin | Coagulation factor Xa |
| Gardenia Fructus | quercetin | Beta-2 adrenergic receptor |
| Gardenia Fructus | quercetin | Stromelysin-1 |
| Gardenia Fructus | quercetin | mRNA of PKA Catalytic Subunit C-alpha |
| Gardenia Fructus | quercetin | Coagulation factor VII |
| Gardenia Fructus | quercetin | Nitric-oxide synthase, endothelial |
| Gardenia Fructus | quercetin | Retinoic acid receptor RXR-alpha |
| Gardenia Fructus | quercetin | Acetylcholinesterase |
| Gardenia Fructus | quercetin | Gamma-aminobutyric acid receptor subunit alpha-1 |
| Gardenia Fructus | quercetin | Amine oxidase [flavin-containing] B |
| Gardenia Fructus | quercetin | Transcription factor p65 |
| Gardenia Fructus | quercetin | Epidermal growth factor receptor |
| Gardenia Fructus | quercetin | RAC-alpha serine/threonine-protein kinase |
| Gardenia Fructus | quercetin | Vascular endothelial growth factor A |
| Gardenia Fructus | quercetin | G1/S-specific cyclin-D1 |
| Gardenia Fructus | quercetin | Apoptosis regulator Bcl-2 |
| Gardenia Fructus | quercetin | Bcl-2-like protein 1 |
| Gardenia Fructus | quercetin | Proto-oncogene c-Fos |
| Gardenia Fructus | quercetin | Cyclin-dependent kinase inhibitor 1 |
| Gardenia Fructus | quercetin | Eukaryotic translation initiation factor 6 |
| Gardenia Fructus | quercetin | Apoptosis regulator BAX |
| Gardenia Fructus | quercetin | Caspase-9 |
| Gardenia Fructus | quercetin | Urokinase-type plasminogen activator |
| Gardenia Fructus | quercetin | 72 kDa type IV collagenase |
| Gardenia Fructus | quercetin | Matrix metalloproteinase-9 |
| Gardenia Fructus | quercetin | Mitogen-activated protein kinase 1 |
| Gardenia Fructus | quercetin | Interleukin-10 |
| Gardenia Fructus | quercetin | Pro-epidermal growth factor |
| Gardenia Fructus | quercetin | Retinoblastoma-associated protein |
| Gardenia Fructus | quercetin | Tumor necrosis factor |
| Gardenia Fructus | quercetin | Transcription factor AP-1 |
| Gardenia Fructus | quercetin | Interleukin-6 |
| Gardenia Fructus | quercetin | Cyclin-dependent kinase inhibitor 2A, isoforms 1/2/3 |
| Gardenia Fructus | quercetin | Activator of 90 kDa heat shock protein ATPase homolog 1 |
| Gardenia Fructus | quercetin | Caspase-3 |
| Gardenia Fructus | quercetin | Cellular tumor antigen p53 |
| Gardenia Fructus | quercetin | ETS domain-containing protein Elk-1 |
| Gardenia Fructus | quercetin | NF-kappa-B inhibitor alpha |
| Gardenia Fructus | quercetin | NADPH--cytochrome P450 reductase |
| Gardenia Fructus | quercetin | Ornithine decarboxylase |
| Gardenia Fructus | quercetin | Xanthine dehydrogenase/oxidase |
| Gardenia Fructus | quercetin | Caspase-8 |
| Gardenia Fructus | quercetin | DNA topoisomerase 1 |
| Gardenia Fructus | quercetin | RAF proto-oncogene serine/threonine-protein kinase |
| Gardenia Fructus | quercetin | Superoxide dismutase [Cu-Zn] |
| Gardenia Fructus | quercetin | Protein kinase C alpha type |
| Gardenia Fructus | quercetin | Interstitial collagenase |
| Gardenia Fructus | quercetin | Hypoxia-inducible factor 1-alpha |
| Gardenia Fructus | quercetin | Signal transducer and activator of transcription 1-alpha/beta |
| Gardenia Fructus | quercetin | Protein CBFA2T1 |
| Gardenia Fructus | quercetin | Probable E3 ubiquitin-protein ligase HERC5 |
| Gardenia Fructus | quercetin | Cell division control protein 2 homolog |
| Gardenia Fructus | quercetin | 78 kDa glucose-regulated protein |
| Gardenia Fructus | quercetin | Receptor tyrosine-protein kinase erbB-2 |
| Gardenia Fructus | quercetin | Peroxisome proliferator-activated receptor gamma |
| Gardenia Fructus | quercetin | Acetyl-CoA carboxylase 1 |
| Gardenia Fructus | quercetin | Heme oxygenase 1 |
| Gardenia Fructus | quercetin | Cytochrome P450 3A4 |
| Gardenia Fructus | quercetin | Cytochrome P450 1A2 |
| Gardenia Fructus | quercetin | Caveolin-1 |
| Gardenia Fructus | quercetin | Myc proto-oncogene protein |
| Gardenia Fructus | quercetin | Tissue factor |
| Gardenia Fructus | quercetin | Gap junction alpha-1 protein |
| Gardenia Fructus | quercetin | Cytochrome P450 1A1 |
| Gardenia Fructus | quercetin | Intercellular adhesion molecule 1 |
| Gardenia Fructus | quercetin | Interleukin-1 beta |
| Gardenia Fructus | quercetin | C-C motif chemokine 2 |
| Gardenia Fructus | quercetin | E-selectin |
| Gardenia Fructus | quercetin | Vascular cell adhesion protein 1 |
| Gardenia Fructus | quercetin | Prostaglandin E2 receptor EP3 subtype |
| Gardenia Fructus | quercetin | Interleukin-8 |
| Gardenia Fructus | quercetin | Protein kinase C beta type |
| Gardenia Fructus | quercetin | Baculoviral IAP repeat-containing protein 5 |
| Gardenia Fructus | quercetin | Dual oxidase 2 |
| Gardenia Fructus | quercetin | Nitric oxide synthase, endothelial |
| Gardenia Fructus | quercetin | Heat shock protein beta-1 |
| Gardenia Fructus | quercetin | Transforming growth factor beta-1 |
| Gardenia Fructus | quercetin | Estrogen sulfotransferase |
| Gardenia Fructus | quercetin | Maltase-glucoamylase, intestinal |
| Gardenia Fructus | quercetin | Interleukin-2 |
| Gardenia Fructus | quercetin | Nuclear receptor subfamily 1 group I member 2 |
| Gardenia Fructus | quercetin | Cytochrome P450 1B1 |
| Gardenia Fructus | quercetin | G2/mitotic-specific cyclin-B1 |
| Gardenia Fructus | quercetin | Tissue-type plasminogen activator |
| Gardenia Fructus | quercetin | Thrombomodulin |
| Gardenia Fructus | quercetin | Plasminogen activator inhibitor 1 |
| Gardenia Fructus | quercetin | Collagen alpha-1(I) chain |
| Gardenia Fructus | quercetin | Interferon gamma |
| Gardenia Fructus | quercetin | Arachidonate 5-lipoxygenase |
| Gardenia Fructus | quercetin | Phosphatidylinositol-3,4,5-trisphosphate 3-phosphatase and dual-specificity protein phosphatase PTEN |
| Gardenia Fructus | quercetin | Interleukin-1 alpha |
| Gardenia Fructus | quercetin | Myeloperoxidase |
| Gardenia Fructus | quercetin | DNA topoisomerase 2-alpha |
| Gardenia Fructus | quercetin | Neutrophil cytosol factor 1 |
| Gardenia Fructus | quercetin | ATP-binding cassette sub-family G member 2 |
| Gardenia Fructus | quercetin | Hyaluronan synthase 2 |
| Gardenia Fructus | quercetin | Glutathione S-transferase P |
| Gardenia Fructus | quercetin | Nuclear factor erythroid 2-related factor 2 |
| Gardenia Fructus | quercetin | NAD(P)H dehydrogenase [quinone] 1 |
| Gardenia Fructus | quercetin | Poly [ADP-ribose] polymerase 1 |
| Gardenia Fructus | quercetin | Aryl hydrocarbon receptor |
| Gardenia Fructus | quercetin | 26S proteasome non-ATPase regulatory subunit 3 |
| Gardenia Fructus | quercetin | Solute carrier family 2, facilitated glucose transporter member 4 |
| Gardenia Fructus | quercetin | Collagen alpha-1(III) chain |
| Gardenia Fructus | quercetin | DNA gyrase subunit B |
| Gardenia Fructus | quercetin | C-X-C motif chemokine 11 |
| Gardenia Fructus | quercetin | C-X-C motif chemokine 2 |
| Gardenia Fructus | quercetin | DDB1- and CUL4-associated factor 5 |
| Gardenia Fructus | quercetin | Nuclear receptor subfamily 1 group I member 3 |
| Gardenia Fructus | quercetin | Serine/threonine-protein kinase Chk2 |
| Gardenia Fructus | quercetin | Insulin receptor |
| Gardenia Fructus | quercetin | Claudin-4 |
| Gardenia Fructus | quercetin | Peroxisome proliferator-activated receptor alpha |
| Gardenia Fructus | quercetin | Peroxisome proliferator-activated receptor delta |
| Gardenia Fructus | quercetin | Heat shock factor protein 1 |
| Gardenia Fructus | quercetin | C-reactive protein |
| Gardenia Fructus | quercetin | C-X-C motif chemokine 10 |
| Gardenia Fructus | quercetin | Inhibitor of nuclear factor kappa-B kinase subunit alpha |
| Gardenia Fructus | quercetin | Osteopontin |
| Gardenia Fructus | quercetin | Runt-related transcription factor 2 |
| Gardenia Fructus | quercetin | Ras association domain-containing protein 1 |
| Gardenia Fructus | quercetin | Transcription factor E2F1 |
| Gardenia Fructus | quercetin | Transcription factor E2F2 |
| Gardenia Fructus | quercetin | Prostatic acid phosphatase |
| Gardenia Fructus | quercetin | Cathepsin D |
| Gardenia Fructus | quercetin | Insulin-like growth factor-binding protein 3 |
| Gardenia Fructus | quercetin | Insulin-like growth factor II |
| Gardenia Fructus | quercetin | CD40 ligand |
| Gardenia Fructus | quercetin | Interferon regulatory factor 1 |
| Gardenia Fructus | quercetin | Receptor tyrosine-protein kinase erbB-3 |
| Gardenia Fructus | quercetin | Serum paraoxonase/arylesterase 1 |
| Gardenia Fructus | quercetin | Type I iodothyronine deiodinase |
| Gardenia Fructus | quercetin | Procollagen C-endopeptidase enhancer 1 |
| Gardenia Fructus | quercetin | Puromycin-sensitive aminopeptidase |
| Gardenia Fructus | quercetin | Hexokinase-2 |
| Gardenia Fructus | quercetin | Homeobox protein Nkx-3.1 |
| Gardenia Fructus | quercetin | Ras GTPase-activating protein 1 |
| Gardenia Fructus | quercetin | Peroxidase C1A |
| Gardenia Fructus | quercetin | Glutathione S-transferase Mu 1 |
| Gardenia Fructus | quercetin | Glutathione S-transferase Mu 2 |
| Gardenia Fructus | MTL | Alcohol dehydrogenase 1C |
| Gardenia Fructus | MTL | Cytochrome P450-cam |
| Gardenia Fructus | Hemo-sol | Prostaglandin G/H synthase 2 |
| Gardenia Fructus | Hemo-sol | Gamma-aminobutyric acid receptor subunit alpha-1 |
| Gardenia Fructus | Hemo-sol | Alcohol dehydrogenase 1B |
| Gardenia Fructus | Hemo-sol | Alcohol dehydrogenase 1C |
| Gardenia Fructus | Hemo-sol | Cytochrome P450-cam |
| Gardenia Fructus | Hemo-sol | Nuclear receptor coactivator 2 |
| Gardenia Fructus | Hemo-sol | Nuclear receptor coactivator 1 |
| Gardenia Fructus | Hemo-sol | Muscarinic acetylcholine receptor M2 |
| Gardenia Fructus | Hemo-sol | Gamma-aminobutyric-acid receptor alpha-2 subunit |
| Gardenia Fructus | Hemo-sol | Muscarinic acetylcholine receptor M1 |
| Gardenia Fructus | Hemo-sol | Gamma-aminobutyric-acid receptor alpha-5 subunit |
| Gardenia Fructus | Hemo-sol | Gamma-aminobutyric-acid receptor alpha-3 subunit |
| Gardenia Fructus | Hemo-sol | Ig gamma-1 chain C region |
| Gardenia Fructus | Hemo-sol | Gamma-aminobutyric-acid receptor subunit alpha-6 |
| Gardenia Fructus | beta-Selinene | Prostaglandin G/H synthase 1 |
| Gardenia Fructus | beta-Selinene | Muscarinic acetylcholine receptor M3 |
| Gardenia Fructus | beta-Selinene | Muscarinic acetylcholine receptor M1 |
| Gardenia Fructus | beta-Selinene | Prostaglandin G/H synthase 2 |
| Gardenia Fructus | beta-Selinene | Gamma-aminobutyric-acid receptor alpha-2 subunit |
| Gardenia Fructus | beta-Selinene | Retinoic acid receptor RXR-alpha |
| Gardenia Fructus | beta-Selinene | Sodium-dependent noradrenaline transporter |
| Gardenia Fructus | beta-Selinene | Gamma-aminobutyric-acid receptor alpha-3 subunit |
| Gardenia Fructus | beta-Selinene | Muscarinic acetylcholine receptor M2 |
| Gardenia Fructus | beta-Selinene | Alpha-1B adrenergic receptor |
| Gardenia Fructus | beta-Selinene | Gamma-aminobutyric acid receptor subunit alpha-1 |
| Gardenia Fructus | beta-Selinene | Nuclear receptor coactivator 2 |
| Gardenia Fructus | beta-Selinene | Gamma-aminobutyric-acid receptor subunit alpha-6 |
| Gardenia Fructus | protocatechuic acid | Prostaglandin G/H synthase 1 |
| Gardenia Fructus | protocatechuic acid | Arachidonate 5-lipoxygenase |
| Gardenia Fructus | protocatechuic acid | Prostaglandin G/H synthase 2 |
| Gardenia Fructus | protocatechuic acid | Amine oxidase [flavin-containing] B |
| Gardenia Fructus | protocatechuic acid | Lysozyme |
| Gardenia Fructus | protocatechuic acid | Nicotinate-nucleotide--dimethylbenzimidazole phosphoribosyltransferase |
| Gardenia Fructus | protocatechuic acid | Alcohol dehydrogenase 1C |
| Gardenia Fructus | protocatechuic acid | Trypsin-3 |
| Gardenia Fructus | protocatechuic acid | Protein kinase C alpha type |
| Gardenia Fructus | protocatechuic acid | Protein kinase C beta type |
| Gardenia Fructus | protocatechuic acid | Protein kinase C gamma type |
| Gardenia Fructus | protocatechuic acid | Maltase-glucoamylase, intestinal |
| Gardenia Fructus | protocatechuic acid | Protein kinase C zeta type |
| Gardenia Fructus | Nonanal | Alcohol dehydrogenase 1B |
| Gardenia Fructus | Nonanal | Alcohol dehydrogenase 1C |
| Gardenia Fructus | Nonanal | Gamma-aminobutyric-acid receptor alpha-2 subunit |
| Gardenia Fructus | Nonanal | Gamma-aminobutyric acid receptor subunit alpha-1 |
| Gardenia Fructus | Nonanal | Cytochrome P450-cam |
| Gardenia Fructus | dec-2-enal | Alcohol dehydrogenase 1B |
| Gardenia Fructus | dec-2-enal | Alcohol dehydrogenase 1C |
| Gardenia Fructus | EIC | Prostaglandin G/H synthase 1 |
| Gardenia Fructus | EIC | Prostaglandin G/H synthase 2 |
| Gardenia Fructus | EIC | Retinoic acid receptor RXR-alpha |
| Gardenia Fructus | EIC | Nuclear receptor coactivator 2 |
| Gardenia Fructus | EIC | Lysozyme |
| Gardenia Fructus | EIC | Nicotinate-nucleotide--dimethylbenzimidazole phosphoribosyltransferase |
| Gardenia Fructus | EIC | Sodium-dependent noradrenaline transporter |
| Gardenia Fructus | EIC | Ig gamma-1 chain C region |
| Gardenia Fructus | EIC | Gamma-aminobutyric-acid receptor alpha-2 subunit |
| Gardenia Fructus | EIC | Gamma-aminobutyric acid receptor subunit alpha-1 |
| Gardenia Fructus | EIC | Cytochrome P450-cam |
| Gardenia Fructus | EIC | Transient receptor potential cation channel subfamily V member 1 |
| Gardenia Fructus | EIC | Muscarinic acetylcholine receptor M1 |
| Gardenia Fructus | EIC | Muscarinic acetylcholine receptor M2 |
| Gardenia Fructus | EIC | Gamma-aminobutyric-acid receptor subunit alpha-6 |
| Gardenia Fructus | caffeic acid | Prostaglandin G/H synthase 1 |
| Gardenia Fructus | caffeic acid | Beta-1 adrenergic receptor |
| Gardenia Fructus | caffeic acid | Prostaglandin G/H synthase 2 |
| Gardenia Fructus | caffeic acid | Alpha-2A adrenergic receptor |
| Gardenia Fructus | caffeic acid | Alpha-2C adrenergic receptor |
| Gardenia Fructus | caffeic acid | Beta-2 adrenergic receptor |
| Gardenia Fructus | caffeic acid | Amine oxidase [flavin-containing] B |
| Gardenia Fructus | caffeic acid | Chymotrypsinogen B |
| Gardenia Fructus | caffeic acid | Sodium-dependent dopamine transporter |
| Gardenia Fructus | caffeic acid | Aldose reductase |
| Gardenia Fructus | caffeic acid | Urokinase-type plasminogen activator |
| Gardenia Fructus | caffeic acid | Amine oxidase [flavin-containing] A |
| Gardenia Fructus | caffeic acid | Lysozyme |
| Gardenia Fructus | caffeic acid | Alpha-1A adrenergic receptor |
| Gardenia Fructus | caffeic acid | Alpha-1D adrenergic receptor |
| Gardenia Fructus | caffeic acid | Tumor necrosis factor |
| Gardenia Fructus | caffeic acid | Cytochrome P450 1A1 |
| Gardenia Fructus | caffeic acid | Protein kinase C beta type |
| Gardenia Fructus | caffeic acid | Tyrosine-protein kinase BTK |
| Gardenia Fructus | caffeic acid | Glial fibrillary acidic protein |
| Gardenia Fructus | caffeic acid | Insulin-like growth factor II |
| Gardenia Fructus | caffeic acid | P-selectin |
| Gardenia Fructus | caffeic acid | Ras-related C3 botulinum toxin substrate 1 |
| Gardenia Fructus | farnesol | Prostaglandin G/H synthase 2 |
| Gardenia Fructus | farnesol | Sodium-dependent noradrenaline transporter |
| Gardenia Fructus | farnesol | Amine oxidase [flavin-containing] B |
| Gardenia Fructus | farnesol | Nuclear receptor coactivator 2 |
| Gardenia Fructus | farnesol | Prostaglandin G/H synthase 1 |
| Gardenia Fructus | farnesol | Retinoic acid receptor RXR-alpha |
| Gardenia Fructus | farnesol | Interleukin-6 |
| Gardenia Fructus | farnesol | Caspase-3 |
| Gardenia Fructus | farnesol | Involucrin |
| Gardenia Fructus | farnesol | Ras-specific guanine nucleotide-releasing factor 2 |
| Gardenia Fructus | farnesol | Bcl-2 homologous antagonist/killer |
| Gardenia Fructus | farnesol | Toll-like receptor 4 |
| Gardenia Fructus | farnesol | Lipoprotein lipase |
| Gardenia Fructus | farnesol | 3-hydroxy-3-methylglutaryl-coenzyme A reductase |
| Gardenia Fructus | farnesol | Peroxisome proliferator-activated receptor alpha |
| Gardenia Fructus | farnesol | Toll-like receptor 2 |
| Gardenia Fructus | farnesol | Beta-defensin 2 |
| Gardenia Fructus | farnesol | Protein HIRA |
| Gardenia Fructus | farnesol | SERTA domain-containing protein 3 |
| Gardenia Fructus | farnesol | Progesterone receptor |
| Gardenia Fructus | farnesol | Bile acid receptor |
| Gardenia Fructus | farnesol | Glutaminase liver isoform, mitochondrial |
| Gardenia Fructus | farnesol | Ig delta chain C region |
| Gardenia Fructus | oleanolic acid | Caspase-9 |
| Gardenia Fructus | oleanolic acid | Caspase-3 |
| Gardenia Fructus | oleanolic acid | Heme oxygenase 1 |
| Gardenia Fructus | oleanolic acid | Intercellular adhesion molecule 1 |
| Gardenia Fructus | oleanolic acid | NAD(P)H dehydrogenase [quinone] 1 |
| Gardenia Fructus | oleanolic acid | Pancreatic alpha-amylase |
| Gardenia Fructus | lauric acid | Prostaglandin G/H synthase 1 |
| Gardenia Fructus | lauric acid | Cholinesterase |
| Gardenia Fructus | lauric acid | Phospholipase A2 |
| Gardenia Fructus | lauric acid | Alcohol dehydrogenase 1B |
| Gardenia Fructus | lauric acid | Alcohol dehydrogenase 1C |
| Gardenia Fructus | lauric acid | Ig gamma-1 chain C region |
| Gardenia Fructus | lauric acid | Ferrichrome-iron receptor |
| Gardenia Fructus | lauric acid | 3-oxoacyl-[acyl-carrier-protein] synthase 1 |
| Gardenia Fructus | lauric acid | Prostaglandin G/H synthase 2 |
| Gardenia Fructus | lauric acid | Rhinovirus coat protein |
| Gardenia Fructus | lauric acid | Transcription factor p65 |
| Gardenia Fructus | lauric acid | RAC-alpha serine/threonine-protein kinase |
| Gardenia Fructus | lauric acid | Interleukin-6 |
| Gardenia Fructus | lauric acid | Interleukin-8 |
| Gardenia Fructus | lauric acid | Dual oxidase 2 |
| Gardenia Fructus | lauric acid | T-lymphocyte activation antigen CD80 |
| Gardenia Fructus | lauric acid | T-lymphocyte activation antigen CD86 |
| Gardenia Fructus | lauric acid | Tumor necrosis factor receptor superfamily member 5 |
| Gardenia Fructus | beta-sitosterol | Progesterone receptor |
| Gardenia Fructus | beta-sitosterol | Nuclear receptor coactivator 2 |
| Gardenia Fructus | beta-sitosterol | Prostaglandin G/H synthase 1 |
| Gardenia Fructus | beta-sitosterol | Prostaglandin G/H synthase 2 |
| Gardenia Fructus | beta-sitosterol | Heat shock protein HSP 90 |
| Gardenia Fructus | beta-sitosterol | Phosphatidylinositol-4,5-bisphosphate 3-kinase catalytic subunit, gamma isoform |
| Gardenia Fructus | beta-sitosterol | Potassium voltage-gated channel subfamily H member 2 |
| Gardenia Fructus | beta-sitosterol | mRNA of PKA Catalytic Subunit C-alpha |
| Gardenia Fructus | beta-sitosterol | Dopamine D1 receptor |
| Gardenia Fructus | beta-sitosterol | Muscarinic acetylcholine receptor M3 |
| Gardenia Fructus | beta-sitosterol | Muscarinic acetylcholine receptor M1 |
| Gardenia Fructus | beta-sitosterol | Sodium channel protein type 5 subunit alpha |
| Gardenia Fructus | beta-sitosterol | Gamma-aminobutyric-acid receptor alpha-2 subunit |
| Gardenia Fructus | beta-sitosterol | Muscarinic acetylcholine receptor M4 |
| Gardenia Fructus | beta-sitosterol | CGMP-inhibited 3',5'-cyclic phosphodiesterase A |
| Gardenia Fructus | beta-sitosterol | 5-hydroxytryptamine 2A receptor |
| Gardenia Fructus | beta-sitosterol | Gamma-aminobutyric-acid receptor alpha-5 subunit |
| Gardenia Fructus | beta-sitosterol | Alpha-1A adrenergic receptor |
| Gardenia Fructus | beta-sitosterol | Gamma-aminobutyric-acid receptor alpha-3 subunit |
| Gardenia Fructus | beta-sitosterol | Muscarinic acetylcholine receptor M2 |
| Gardenia Fructus | beta-sitosterol | Alpha-1B adrenergic receptor |
| Gardenia Fructus | beta-sitosterol | Beta-2 adrenergic receptor |
| Gardenia Fructus | beta-sitosterol | Neuronal acetylcholine receptor subunit alpha-2 |
| Gardenia Fructus | beta-sitosterol | Sodium-dependent serotonin transporter |
| Gardenia Fructus | beta-sitosterol | Mu-type opioid receptor |
| Gardenia Fructus | beta-sitosterol | Gamma-aminobutyric acid receptor subunit alpha-1 |
| Gardenia Fructus | beta-sitosterol | Neuronal acetylcholine receptor protein, alpha-7 chain |
| Gardenia Fructus | beta-sitosterol | Cytochrome P450-cam |
| Gardenia Fructus | beta-sitosterol | Apoptosis regulator Bcl-2 |
| Gardenia Fructus | beta-sitosterol | Apoptosis regulator BAX |
| Gardenia Fructus | beta-sitosterol | Caspase-9 |
| Gardenia Fructus | beta-sitosterol | Transcription factor AP-1 |
| Gardenia Fructus | beta-sitosterol | Caspase-3 |
| Gardenia Fructus | beta-sitosterol | Caspase-8 |
| Gardenia Fructus | beta-sitosterol | Protein kinase C alpha type |
| Gardenia Fructus | beta-sitosterol | Transforming growth factor beta-1 |
| Gardenia Fructus | beta-sitosterol | Serum paraoxonase/arylesterase 1 |
| Gardenia Fructus | beta-sitosterol | Microtubule-associated protein 2 |
| Gardenia Fructus | syringaresinol | Coagulation factor Xa |
| Gardenia Fructus | syringaresinol | Prostaglandin G/H synthase 2 |
| Gardenia Fructus | syringaresinol | DNA topoisomerase II |
| Gardenia Fructus | syringaresinol | Nuclear receptor coactivator 2 |
| Gardenia Fructus | syringaresinol | Calmodulin |
| Gardenia Fructus | syringaresinol | Potassium voltage-gated channel subfamily H member 2 |
| Gardenia Fructus | syringaresinol | Coagulation factor VII |
| Gardenia Fructus | syringaresinol | Calcium-activated potassium channel subunit alpha 1 |
| Gardenia Fructus | syringaresinol | Heat shock protein HSP 90 |
| Gardenia Fructus | kaempferol | Nitric oxide synthase, inducible |
| Gardenia Fructus | kaempferol | Prostaglandin G/H synthase 1 |
| Gardenia Fructus | kaempferol | Androgen receptor |
| Gardenia Fructus | kaempferol | Peroxisome proliferator activated receptor gamma |
| Gardenia Fructus | kaempferol | Prostaglandin G/H synthase 2 |
| Gardenia Fructus | kaempferol | Heat shock protein HSP 90 |
| Gardenia Fructus | kaempferol | Phosphatidylinositol-4,5-bisphosphate 3-kinase catalytic subunit, gamma isoform |
| Gardenia Fructus | kaempferol | mRNA of PKA Catalytic Subunit C-alpha |
| Gardenia Fructus | kaempferol | Nuclear receptor coactivator 2 |
| Gardenia Fructus | kaempferol | Dipeptidyl peptidase IV |
| Gardenia Fructus | kaempferol | Trypsin-1 |
| Gardenia Fructus | kaempferol | Progesterone receptor |
| Gardenia Fructus | kaempferol | Thrombin |
| Gardenia Fructus | kaempferol | Muscarinic acetylcholine receptor M1 |
| Gardenia Fructus | kaempferol | Nitric-oxide synthase, endothelial |
| Gardenia Fructus | kaempferol | Gamma-aminobutyric-acid receptor alpha-2 subunit |
| Gardenia Fructus | kaempferol | Acetylcholinesterase |
| Gardenia Fructus | kaempferol | Sodium-dependent noradrenaline transporter |
| Gardenia Fructus | kaempferol | Muscarinic acetylcholine receptor M2 |
| Gardenia Fructus | kaempferol | Alpha-1B adrenergic receptor |
| Gardenia Fructus | kaempferol | Gamma-aminobutyric acid receptor subunit alpha-1 |
| Gardenia Fructus | kaempferol | DNA topoisomerase II |
| Gardenia Fructus | kaempferol | Coagulation factor VII |
| Gardenia Fructus | kaempferol | Calmodulin |
| Gardenia Fructus | kaempferol | Transcription factor p65 |
| Gardenia Fructus | kaempferol | Inhibitor of nuclear factor kappa-B kinase subunit beta |
| Gardenia Fructus | kaempferol | RAC-alpha serine/threonine-protein kinase |
| Gardenia Fructus | kaempferol | Apoptosis regulator Bcl-2 |
| Gardenia Fructus | kaempferol | Apoptosis regulator BAX |
| Gardenia Fructus | kaempferol | Tumor necrosis factor |
| Gardenia Fructus | kaempferol | Transcription factor AP-1 |
| Gardenia Fructus | kaempferol | Activator of 90 kDa heat shock protein ATPase homolog 1 |
| Gardenia Fructus | kaempferol | Caspase-3 |
| Gardenia Fructus | kaempferol | Mitogen-activated protein kinase 8 |
| Gardenia Fructus | kaempferol | Xanthine dehydrogenase/oxidase |
| Gardenia Fructus | kaempferol | Interstitial collagenase |
| Gardenia Fructus | kaempferol | Signal transducer and activator of transcription 1-alpha/beta |
| Gardenia Fructus | kaempferol | Cell division control protein 2 homolog |
| Gardenia Fructus | kaempferol | Peroxisome proliferator-activated receptor gamma |
| Gardenia Fructus | kaempferol | Heme oxygenase 1 |
| Gardenia Fructus | kaempferol | Cytochrome P450 3A4 |
| Gardenia Fructus | kaempferol | Cytochrome P450 1A2 |
| Gardenia Fructus | kaempferol | Cytochrome P450 1A1 |
| Gardenia Fructus | kaempferol | Intercellular adhesion molecule 1 |
| Gardenia Fructus | kaempferol | E-selectin |
| Gardenia Fructus | kaempferol | Vascular cell adhesion protein 1 |
| Gardenia Fructus | kaempferol | Nuclear receptor subfamily 1 group I member 2 |
| Gardenia Fructus | kaempferol | Cytochrome P450 1B1 |
| Gardenia Fructus | kaempferol | Arachidonate 5-lipoxygenase |
| Gardenia Fructus | kaempferol | Hyaluronan synthase 2 |
| Gardenia Fructus | kaempferol | Glutathione S-transferase P |
| Gardenia Fructus | kaempferol | Aryl hydrocarbon receptor |
| Gardenia Fructus | kaempferol | 26S proteasome non-ATPase regulatory subunit 3 |
| Gardenia Fructus | kaempferol | Solute carrier family 2, facilitated glucose transporter member 4 |
| Gardenia Fructus | kaempferol | Nuclear receptor subfamily 1 group I member 3 |
| Gardenia Fructus | kaempferol | Insulin receptor |
| Gardenia Fructus | kaempferol | Type I iodothyronine deiodinase |
| Gardenia Fructus | kaempferol | Serine/threonine-protein phosphatase 2B catalytic subunit alpha isoform |
| Gardenia Fructus | kaempferol | Peroxidase C1A |
| Gardenia Fructus | kaempferol | Glutathione S-transferase Mu 1 |
| Gardenia Fructus | kaempferol | Glutathione S-transferase Mu 2 |
| Gardenia Fructus | kaempferol | Aldo-keto reductase family 1 member C3 |
| Gardenia Fructus | kaempferol | Antileukoproteinase |
| Gardenia Fructus | Stigmasterol | Progesterone receptor |
| Gardenia Fructus | Stigmasterol | Mineralocorticoid receptor |
| Gardenia Fructus | Stigmasterol | Nuclear receptor coactivator 2 |
| Gardenia Fructus | Stigmasterol | Alcohol dehydrogenase 1C |
| Gardenia Fructus | Stigmasterol | Ig gamma-1 chain C region |
| Gardenia Fructus | Stigmasterol | Retinoic acid receptor RXR-alpha |
| Gardenia Fructus | Stigmasterol | Nuclear receptor coactivator 1 |
| Gardenia Fructus | Stigmasterol | Prostaglandin G/H synthase 1 |
| Gardenia Fructus | Stigmasterol | Prostaglandin G/H synthase 2 |
| Gardenia Fructus | Stigmasterol | Alpha-2A adrenergic receptor |
| Gardenia Fructus | Stigmasterol | Sodium-dependent noradrenaline transporter |
| Gardenia Fructus | Stigmasterol | Sodium-dependent dopamine transporter |
| Gardenia Fructus | Stigmasterol | Beta-2 adrenergic receptor |
| Gardenia Fructus | Stigmasterol | Aldose reductase |
| Gardenia Fructus | Stigmasterol | Urokinase-type plasminogen activator |
| Gardenia Fructus | Stigmasterol | Leukotriene A-4 hydrolase |
| Gardenia Fructus | Stigmasterol | Amine oxidase [flavin-containing] B |
| Gardenia Fructus | Stigmasterol | Amine oxidase [flavin-containing] A |
| Gardenia Fructus | Stigmasterol | mRNA of PKA Catalytic Subunit C-alpha |
| Gardenia Fructus | Stigmasterol | Chymotrypsinogen B |
| Gardenia Fructus | Stigmasterol | Muscarinic acetylcholine receptor M3 |
| Gardenia Fructus | Stigmasterol | Muscarinic acetylcholine receptor M1 |
| Gardenia Fructus | Stigmasterol | Beta-1 adrenergic receptor |
| Gardenia Fructus | Stigmasterol | Sodium channel protein type 5 subunit alpha |
| Gardenia Fructus | Stigmasterol | 5-hydroxytryptamine 2A receptor |
| Gardenia Fructus | Stigmasterol | Alpha-1A adrenergic receptor |
| Gardenia Fructus | Stigmasterol | Gamma-aminobutyric-acid receptor alpha-3 subunit |
| Gardenia Fructus | Stigmasterol | Muscarinic acetylcholine receptor M2 |
| Gardenia Fructus | Stigmasterol | Alpha-1B adrenergic receptor |
| Gardenia Fructus | Stigmasterol | Gamma-aminobutyric acid receptor subunit alpha-1 |
| Gardenia Fructus | Stigmasterol | Neuronal acetylcholine receptor protein, alpha-7 chain |
| Gardenia Fructus | Eucarvone | Gamma-aminobutyric-acid receptor alpha-2 subunit |
| Gardenia Fructus | Eucarvone | Gamma-aminobutyric-acid receptor alpha-5 subunit |
| Gardenia Fructus | Eucarvone | Muscarinic acetylcholine receptor M2 |
| Gardenia Fructus | Eucarvone | Gamma-aminobutyric acid receptor subunit alpha-1 |
| Gardenia Fructus | Stigmasterol | Muscarinic acetylcholine receptor M3 |
| Gardenia Fructus | Stigmasterol | Muscarinic acetylcholine receptor M1 |
| Gardenia Fructus | Stigmasterol | Beta-1 adrenergic receptor |
| Gardenia Fructus | Stigmasterol | Sodium channel protein type 5 subunit alpha |
| Gardenia Fructus | Stigmasterol | 5-hydroxytryptamine 2A receptor |
| Gardenia Fructus | Stigmasterol | Alpha-1A adrenergic receptor |
| Gardenia Fructus | Stigmasterol | Gamma-aminobutyric-acid receptor alpha-3 subunit |
| Gardenia Fructus | Stigmasterol | Muscarinic acetylcholine receptor M2 |
| Gardenia Fructus | Stigmasterol | Alpha-1B adrenergic receptor |
| Gardenia Fructus | Stigmasterol | Gamma-aminobutyric acid receptor subunit alpha-1 |
| Gardenia Fructus | Stigmasterol | Neuronal acetylcholine receptor protein, alpha-7 chain |
| Gardenia Fructus | Eucarvone | Gamma-aminobutyric-acid receptor alpha-2 subunit |
| Gardenia Fructus | Eucarvone | Gamma-aminobutyric-acid receptor alpha-5 subunit |
| Gardenia Fructus | Eucarvone | Muscarinic acetylcholine receptor M2 |
| Gardenia Fructus | Eucarvone | Gamma-aminobutyric acid receptor subunit alpha-1 |
| Gardenia Fructus | oleic acid | Alcohol dehydrogenase 1A |
| Gardenia Fructus | oleic acid | Lysozyme |
| Gardenia Fructus | oleic acid | Nicotinate-nucleotide--dimethylbenzimidazole phosphoribosyltransferase |
| Gardenia Fructus | oleic acid | Trypsin-3 |
| Gardenia Fructus | oleic acid | Retinoic acid receptor RXR-alpha |
| Gardenia Fructus | oleic acid | Cytochrome P450-cam |
| Gardenia Fructus | oleic acid | Urokinase-type plasminogen activator |
| Gardenia Fructus | oleic acid | Superoxide dismutase [Cu-Zn] |
| Gardenia Fructus | oleic acid | Catalase |
| Gardenia Fructus | oleic acid | Telomerase protein component 1 |
| Gardenia Fructus | oleic acid | Endothelin-1 |
| Gardenia Fructus | oleic acid | Receptor tyrosine-protein kinase erbB-2 |
| Gardenia Fructus | oleic acid | Peroxisome proliferator-activated receptor gamma |
| Gardenia Fructus | oleic acid | Lipoprotein lipase |
| Gardenia Fructus | oleic acid | Neuromodulin |
| Gardenia Fructus | oleic acid | Plasminogen activator inhibitor 1 |
| Gardenia Fructus | oleic acid | Brain-derived neurotrophic factor |
| Gardenia Fructus | oleic acid | 3-hydroxy-3-methylglutaryl-coenzyme A reductase |
| Gardenia Fructus | oleic acid | Myeloperoxidase |
| Gardenia Fructus | oleic acid | Peroxisome proliferator-activated receptor alpha |
| Gardenia Fructus | oleic acid | Peroxisome proliferator-activated receptor delta |
| Gardenia Fructus | oleic acid | C-reactive protein |
| Gardenia Fructus | oleic acid | Serum paraoxonase/arylesterase 1 |
| Gardenia Fructus | oleic acid | Insulin |
| Gardenia Fructus | oleic acid | Plasminogen |
| Gardenia Fructus | oleic acid | Fatty acid-binding protein, liver |
| Gardenia Fructus | oleic acid | Retinol-binding protein 2 |
| Gardenia Fructus | oleic acid | Glucagon |
| Gardenia Fructus | oleic acid | Glutamyl aminopeptidase |
| Gardenia Fructus | oleic acid | Mitochondrial uncoupling protein 2 |
| Gardenia Fructus | oleic acid | Sterol O-acyltransferase 1 |
| Gardenia Fructus | oleic acid | Cholecystokinin |
| Gardenia Fructus | oleic acid | Cbp/p300-interacting transactivator 1 |
| Gardenia Fructus | oleic acid | BDNF/NT-3 growth factors receptor |
| Gardenia Fructus | oleic acid | Pancreas/duodenum homeobox protein 1 |
| Gardenia Fructus | oleic acid | Solute carrier family 2, facilitated glucose transporter member 2 |
| Gardenia Fructus | oleic acid | Peptidyl-glycine alpha-amidating monooxygenase |
| Gardenia Fructus | oleic acid | Acyl-CoA desaturase |
| Gardenia Fructus | oleic acid | Mitochondrial uncoupling protein 3 |
| Gardenia Fructus | oleic acid | Cholesteryl ester transfer protein |
| Gardenia Fructus | oleic acid | Peptide YY |
| Gardenia Fructus | oleic acid | Aspartyl aminopeptidase |
| Gardenia Fructus | oleic acid | Cell-death-related nuclease 7 |
| Gardenia Fructus | trans-2,4-decadienal | Gamma-aminobutyric-acid receptor alpha-2 subunit |
| Gardenia Fructus | trans-2,4-decadienal | Gamma-aminobutyric acid receptor subunit alpha-1 |
| Gardenia Fructus | trans-2,4-decadienal | Cytochrome P450-cam |
| Gardenia Fructus | trans-2,4-decadienal | Alcohol dehydrogenase 1C |
| Gardenia Fructus | trans-2,4-decadienal | G1/S-specific cyclin-D1 |
| Gardenia Fructus | trans-2,4-decadienal | Eukaryotic translation initiation factor 6 |
| Gardenia Fructus | trans-2,4-decadienal | Retinoblastoma-associated protein |
| Gardenia Fructus | trans-2,4-decadienal | Cell division protein kinase 4 |
| Gardenia Fructus | stearic acid | Prostaglandin G/H synthase 1 |
| Gardenia Fructus | stearic acid | Prostaglandin G/H synthase 2 |
| Gardenia Fructus | stearic acid | Retinoic acid receptor RXR-alpha |
| Gardenia Fructus | stearic acid | Nuclear receptor coactivator 2 |
| Gardenia Fructus | stearic acid | Ig gamma-1 chain C region |
| Gardenia Fructus | stearic acid | Transcription factor Sp1 |
| Gardenia Fructus | stearic acid | Ectonucleotide pyrophosphatase/phosphodiesterase family member 7 |
| Gardenia Fructus | paeonol | Prostaglandin G/H synthase 1 |
| Gardenia Fructus | paeonol | Beta-1 adrenergic receptor |
| Gardenia Fructus | paeonol | Prostaglandin G/H synthase 2 |
| Gardenia Fructus | paeonol | Alpha-2A adrenergic receptor |
| Gardenia Fructus | paeonol | Alpha-2C adrenergic receptor |
| Gardenia Fructus | paeonol | Sodium-dependent noradrenaline transporter |
| Gardenia Fructus | paeonol | Alpha-2B adrenergic receptor |
| Gardenia Fructus | paeonol | Alpha-1B adrenergic receptor |
| Gardenia Fructus | paeonol | Sodium-dependent dopamine transporter |
| Gardenia Fructus | paeonol | Beta-2 adrenergic receptor |
| Gardenia Fructus | paeonol | Alpha-1D adrenergic receptor |
| Gardenia Fructus | paeonol | Beta-lactamase |
| Gardenia Fructus | paeonol | Amine oxidase [flavin-containing] B |
| Gardenia Fructus | paeonol | Amine oxidase [flavin-containing] A |
| Gardenia Fructus | paeonol | Chymotrypsinogen B |
| Gardenia Fructus | paeonol | Alpha-1A adrenergic receptor |
| Gardenia Fructus | paeonol | Muscarinic acetylcholine receptor M1 |
| Gardenia Fructus | paeonol | Muscarinic acetylcholine receptor M2 |
| Gardenia Fructus | paeonol | Transcription factor p65 |
| Gardenia Fructus | paeonol | RAC-alpha serine/threonine-protein kinase |
| Gardenia Fructus | paeonol | Apoptosis regulator Bcl-2 |
| Gardenia Fructus | paeonol | Apoptosis regulator BAX |
| Gardenia Fructus | paeonol | Mitogen-activated protein kinase 1 |
| Gardenia Fructus | paeonol | Tumor necrosis factor |
| Gardenia Fructus | paeonol | Activator of 90 kDa heat shock protein ATPase homolog 1 |
| Gardenia Fructus | paeonol | NF-kappa-B inhibitor alpha |
| Gardenia Fructus | paeonol | Intercellular adhesion molecule 1 |
| Gardenia Fructus | paeonol | Interleukin-2 |
| Gardenia Fructus | paeonol | Tyrosinase |
| Gardenia Fructus | paeonol | Phosphatidylinositol-3,4,5-trisphosphate 3-phosphatase and dual-specificity protein phosphatase PTEN |
| Gardenia Fructus | methyl palmitate | Prostaglandin G/H synthase 1 |
| Gardenia Fructus | methyl palmitate | Nuclear receptor coactivator 2 |
| Gardenia Fructus | methyl palmitate | Ig gamma-1 chain C region |
| Gardenia Fructus | methyl palmitate | Transcription factor p65 |
| Gardenia Fructus | methyl palmitate | Interleukin-10 |
| Gardenia Fructus | methyl palmitate | Tumor necrosis factor |
| Gardenia Fructus | methyl palmitate | Interleukin-6 |
| Gardenia Fructus | methyl palmitate | Prostaglandin G/H synthase 2 |
| Gardenia Fructus | methyl palmitate | Prostaglandin E2 receptor EP3 subtype |
| Gardenia Fructus | 13657-68-6 | Mineralocorticoid receptor |
| Gardenia Fructus | 13657-68-6 | Muscarinic acetylcholine receptor M1 |
| Gardenia Fructus | 13657-68-6 | Gamma-aminobutyric-acid receptor alpha-2 subunit |
| Gardenia Fructus | 13657-68-6 | Gamma-aminobutyric-acid receptor alpha-5 subunit |
| Gardenia Fructus | 13657-68-6 | Gamma-aminobutyric-acid receptor alpha-3 subunit |
| Gardenia Fructus | 13657-68-6 | Muscarinic acetylcholine receptor M2 |
| Gardenia Fructus | 13657-68-6 | Gamma-aminobutyric acid receptor subunit alpha-1 |
| Gardenia Fructus | 13657-68-6 | Ig gamma-1 chain C region |
| Gardenia Fructus | 13657-68-6 | Gamma-aminobutyric-acid receptor subunit alpha-6 |
| Gardenia Fructus | Germacron | Gamma-aminobutyric-acid receptor alpha-2 subunit |
| Gardenia Fructus | Germacron | Gamma-aminobutyric acid receptor subunit alpha-1 |
| Gardenia Fructus | Germacron | Cytochrome P450-cam |
| Gardenia Fructus | Germacron | Prostaglandin G/H synthase 1 |
| Gardenia Fructus | Germacron | Muscarinic acetylcholine receptor M1 |
| Gardenia Fructus | Germacron | Prostaglandin G/H synthase 2 |
| Gardenia Fructus | Germacron | Sodium-dependent noradrenaline transporter |
| Gardenia Fructus | Germacron | Glutamate receptor 2 |
| Gardenia Fructus | LINALOOL (D) | Muscarinic acetylcholine receptor M1 |
| Gardenia Fructus | LINALOOL (D) | Gamma-aminobutyric-acid receptor alpha-2 subunit |
| Gardenia Fructus | LINALOOL (D) | Muscarinic acetylcholine receptor M2 |
| Gardenia Fructus | LINALOOL (D) | Sodium-dependent noradrenaline transporter |
| Gardenia Fructus | LINALOOL (D) | Gamma-aminobutyric-acid receptor alpha-5 subunit |
| Gardenia Fructus | LINALOOL (D) | Gamma-aminobutyric-acid receptor alpha-3 subunit |
| Gardenia Fructus | LINALOOL (D) | Gamma-aminobutyric-acid receptor subunit alpha-6 |
| Gardenia Fructus | LINALOOL (D) | Prostaglandin G/H synthase 2 |
| Gardenia Fructus | LINALOOL (D) | Alcohol dehydrogenase 1C |
| Gardenia Fructus | LINALOOL (D) | Gamma-aminobutyric acid receptor subunit alpha-1 |
| Gardenia Fructus | LINALOOL (D) | Lysozyme |
| Gardenia Fructus | LINALOOL (D) | Nicotinate-nucleotide--dimethylbenzimidazole phosphoribosyltransferase |
| Gardenia Fructus | LINALOOL (D) | Cytochrome P450-cam |
| Gardenia Fructus | LINALOOL (D) | Prostaglandin G/H synthase 1 |
| Gardenia Fructus | LINALOOL (D) | Muscarinic acetylcholine receptor M3 |
| Gardenia Fructus | LINALOOL (D) | Beta-1 adrenergic receptor |
| Gardenia Fructus | LINALOOL (D) | Alpha-2A adrenergic receptor |
| Gardenia Fructus | LINALOOL (D) | Retinoic acid receptor RXR-alpha |
| Gardenia Fructus | LINALOOL (D) | Alpha-1A adrenergic receptor |
| Gardenia Fructus | LINALOOL (D) | Alpha-1B adrenergic receptor |
| Gardenia Fructus | LINALOOL (D) | Sodium-dependent dopamine transporter |
| Gardenia Fructus | LINALOOL (D) | Beta-2 adrenergic receptor |
| Gardenia Fructus | LINALOOL (D) | Sodium-dependent serotonin transporter |
| Gardenia Fructus | LINALOOL (D) | Leukotriene A-4 hydrolase |
| Gardenia Fructus | LINALOOL (D) | Amine oxidase [flavin-containing] B |
| Gardenia Fructus | LINALOOL (D) | cAMP-dependent protein kinase inhibitor alpha |
| Gardenia Fructus | Ethylpalmitate | Nuclear receptor coactivator 2 |
| Gardenia Fructus | C09704 | Prostaglandin G/H synthase 2 |
| Gardenia Fructus | C09704 | Nuclear receptor coactivator 2 |
| Gardenia Fructus | myristic acid | Prostaglandin G/H synthase 1 |
| Gardenia Fructus | myristic acid | Prostaglandin G/H synthase 2 |
| Gardenia Fructus | myristic acid | Cholinesterase |
| Gardenia Fructus | myristic acid | Phospholipase A2 |
| Gardenia Fructus | myristic acid | Rhinovirus coat protein |
| Gardenia Fructus | myristic acid | Ig gamma-1 chain C region |
| Gardenia Fructus | myristic acid | Ferrichrome-iron receptor |
| Gardenia Fructus | myristic acid | 3-oxoacyl-[acyl-carrier-protein] synthase 1 |
| Gardenia Fructus | myristic acid | Nuclear receptor coactivator 2 |
| Gardenia Fructus | myristic acid | Nuclear receptor coactivator 1 |
| Gardenia Fructus | myristic acid | Phosphatidylcholine-sterol acyltransferase |
| Gardenia Fructus | OCTENAL | Alcohol dehydrogenase 1B |
| Gardenia Fructus | OCTENAL | Alcohol dehydrogenase 1C |
| Gardenia Fructus | OCTENAL | Trypsin-3 |
| Gardenia Fructus | Mandenol | Prostaglandin G/H synthase 1 |
| Gardenia Fructus | Mandenol | Prostaglandin G/H synthase 2 |
| Gardenia Fructus | Mandenol | Nuclear receptor coactivator 2 |
| Gardenia Fructus | Daturic acid | Prostaglandin G/H synthase 1 |
| Gardenia Fructus | Daturic acid | Ig gamma-1 chain C region |
| Gardenia Fructus | Daturic acid | Nuclear receptor coactivator 2 |
| Gardenia Fructus | METHYL LINOLEATE | Prostaglandin G/H synthase 1 |
| Gardenia Fructus | METHYL LINOLEATE | Prostaglandin G/H synthase 2 |
| Gardenia Fructus | METHYL LINOLEATE | Nuclear receptor coactivator 2 |
| Gardenia Fructus | METHYL LINOLEATE | Retinoic acid receptor RXR-alpha |
| Gardenia Fructus | Scandoside methyl ester | Carbonic anhydrase II |
| Gardenia Fructus | scandoside_qt | Prostaglandin G/H synthase 1 |
| Gardenia Fructus | scandoside_qt | Prostaglandin G/H synthase 2 |
| Gardenia Fructus | scandoside_qt | Carbonic anhydrase II |
| Gardenia Fructus | scandoside_qt | Trypsin-1 |
| Gardenia Fructus | scandoside_qt | Glutamate receptor 2 |
| Gardenia Fructus | Deacetyl asperulosidic acid methyl ester | Carbonic anhydrase II |
| Gardenia Fructus | Deacetyl asperulosidic acid methyl ester | Trypsin-1 |
| Gardenia Fructus | deacetyl asperuloside acid_qt | Prostaglandin G/H synthase 1 |
| Gardenia Fructus | deacetyl asperuloside acid_qt | Prostaglandin G/H synthase 2 |
| Gardenia Fructus | deacetyl asperuloside acid_qt | Carbonic anhydrase II |
| Gardenia Fructus | deacetyl asperuloside acid_qt | Trypsin-1 |
| Gardenia Fructus | deacetyl asperuloside acid_qt | Glutamate receptor 2 |
| Gardenia Fructus | Geniposidic acid | Carbonic anhydrase II |
| Gardenia Fructus | Geniposidic acid | Thrombin |
| Gardenia Fructus | geniposidie acid_qt | Prostaglandin G/H synthase 2 |
| Gardenia Fructus | geniposidie acid_qt | Carbonic anhydrase II |
| Gardenia Fructus | geniposidie acid_qt | Gamma-aminobutyric acid receptor subunit alpha-1 |
| Gardenia Fructus | geniposidie acid_qt | Trypsin-1 |
| Gardenia Fructus | geniposidie acid_qt | Glutamate receptor 2 |
| Gardenia Fructus | geniposidie acid_qt | Ig gamma-1 chain C region |
| Gardenia Fructus | zoomaric acid | Prostaglandin G/H synthase 1 |
| Gardenia Fructus | zoomaric acid | Prostaglandin G/H synthase 2 |
| Gardenia Fructus | zoomaric acid | Nuclear receptor coactivator 2 |
| Gardenia Fructus | Methyl vaccenate | Nuclear receptor coactivator 2 |
| Gardenia Fructus | Izoforon | Gamma-aminobutyric-acid receptor alpha-2 subunit |
| Gardenia Fructus | Izoforon | Gamma-aminobutyric-acid receptor alpha-3 subunit |
| Gardenia Fructus | Izoforon | Gamma-aminobutyric acid receptor subunit alpha-1 |
| Gardenia Fructus | Izoforon | Cytochrome P450-cam |
| Gardenia Fructus | Izoforon | Gamma-aminobutyric-acid receptor subunit alpha-6 |
| Gardenia Fructus | isoimperatorin | Prostaglandin G/H synthase 2 |
| Gardenia Fructus | hexanoic acid | Alcohol dehydrogenase 1C |
| Gardenia Fructus | hexanoic acid | Trypsin-3 |
| Gardenia Fructus | hexanoic acid | Bacillolysin |
| Gardenia Fructus | hexanoic acid | Inositol-3-phosphate synthase 1 |
| Gardenia Fructus | Exceparl M-OL | Prostaglandin G/H synthase 1 |
| Gardenia Fructus | Exceparl M-OL | Prostaglandin G/H synthase 2 |
| Gardenia Fructus | Exceparl M-OL | Retinoic acid receptor RXR-alpha |
| Gardenia Fructus | Exceparl M-OL | Nuclear receptor coactivator 2 |
| Gardenia Fructus | chrysin | Prostaglandin G/H synthase 1 |
| Gardenia Fructus | chrysin | Androgen receptor |
| Gardenia Fructus | chrysin | Prostaglandin G/H synthase 2 |
| Gardenia Fructus | chrysin | CGMP-inhibited 3',5'-cyclic phosphodiesterase A |
| Gardenia Fructus | chrysin | Sodium-dependent serotonin transporter |
| Gardenia Fructus | chrysin | Gamma-aminobutyric acid receptor subunit alpha-1 |
| Gardenia Fructus | chrysin | Dipeptidyl peptidase IV |
| Gardenia Fructus | chrysin | Heat shock protein HSP 90 |
| Gardenia Fructus | chrysin | Phosphatidylinositol-4,5-bisphosphate 3-kinase catalytic subunit, gamma isoform |
| Gardenia Fructus | chrysin | Amine oxidase [flavin-containing] B |
| Gardenia Fructus | chrysin | mRNA of PKA Catalytic Subunit C-alpha |
| Gardenia Fructus | chrysin | cAMP-dependent protein kinase inhibitor alpha |
| Gardenia Fructus | chrysin | Thrombin |
| Gardenia Fructus | chrysin | Cyclin-dependent kinase inhibitor 1 |
| Gardenia Fructus | chrysin | Transforming growth factor beta-1 |
| Gardenia Fructus | chrysin | Interleukin-4 |
| Gardenia Fructus | chrysin | Cytochrome P450 19A1 |
| Gardenia Fructus | chrysin | Interleukin-13 |
| Gardenia Fructus | chrysin | High affinity immunoglobulin epsilon receptor subunit beta |
| Gardenia Fructus | Ethyl oleate (NF) | Nuclear receptor coactivator 2 |
| Gardenia Fructus | 5-hydroxy-7-methoxy-2-(3,4,5-trimethoxyphenyl)chromone | Nitric oxide synthase, inducible |
| Gardenia Fructus | 5-hydroxy-7-methoxy-2-(3,4,5-trimethoxyphenyl)chromone | Prostaglandin G/H synthase 1 |
| Gardenia Fructus | 5-hydroxy-7-methoxy-2-(3,4,5-trimethoxyphenyl)chromone | Thrombin |
| Gardenia Fructus | 5-hydroxy-7-methoxy-2-(3,4,5-trimethoxyphenyl)chromone | Potassium voltage-gated channel subfamily H member 2 |
| Gardenia Fructus | 5-hydroxy-7-methoxy-2-(3,4,5-trimethoxyphenyl)chromone | Estrogen receptor |
| Gardenia Fructus | 5-hydroxy-7-methoxy-2-(3,4,5-trimethoxyphenyl)chromone | Androgen receptor |
| Gardenia Fructus | 5-hydroxy-7-methoxy-2-(3,4,5-trimethoxyphenyl)chromone | Sodium channel protein type 5 subunit alpha |
| Gardenia Fructus | 5-hydroxy-7-methoxy-2-(3,4,5-trimethoxyphenyl)chromone | Peroxisome proliferator activated receptor gamma |
| Gardenia Fructus | 5-hydroxy-7-methoxy-2-(3,4,5-trimethoxyphenyl)chromone | Coagulation factor Xa |
| Gardenia Fructus | 5-hydroxy-7-methoxy-2-(3,4,5-trimethoxyphenyl)chromone | Prostaglandin G/H synthase 2 |
| Gardenia Fructus | 5-hydroxy-7-methoxy-2-(3,4,5-trimethoxyphenyl)chromone | Nitric-oxide synthase, endothelial |
| Gardenia Fructus | 5-hydroxy-7-methoxy-2-(3,4,5-trimethoxyphenyl)chromone | Voltage-dependent calcium channel subunit alpha-2/delta-1 |
| Gardenia Fructus | 5-hydroxy-7-methoxy-2-(3,4,5-trimethoxyphenyl)chromone | DNA topoisomerase II |
| Gardenia Fructus | 5-hydroxy-7-methoxy-2-(3,4,5-trimethoxyphenyl)chromone | Estrogen receptor beta |
| Gardenia Fructus | 5-hydroxy-7-methoxy-2-(3,4,5-trimethoxyphenyl)chromone | Dipeptidyl peptidase IV |
| Gardenia Fructus | 5-hydroxy-7-methoxy-2-(3,4,5-trimethoxyphenyl)chromone | Mitogen-activated protein kinase 14 |
| Gardenia Fructus | 5-hydroxy-7-methoxy-2-(3,4,5-trimethoxyphenyl)chromone | Glycogen synthase kinase-3 beta |
| Gardenia Fructus | 5-hydroxy-7-methoxy-2-(3,4,5-trimethoxyphenyl)chromone | Heat shock protein HSP 90 |
| Gardenia Fructus | 5-hydroxy-7-methoxy-2-(3,4,5-trimethoxyphenyl)chromone | Serine/threonine-protein kinase Chk1 |
| Gardenia Fructus | 5-hydroxy-7-methoxy-2-(3,4,5-trimethoxyphenyl)chromone | Trypsin-1 |
| Gardenia Fructus | 5-hydroxy-7-methoxy-2-(3,4,5-trimethoxyphenyl)chromone | Nuclear receptor coactivator 2 |
| Gardenia Fructus | 5-hydroxy-7-methoxy-2-(3,4,5-trimethoxyphenyl)chromone | Nuclear receptor coactivator 1 |
| Gardenia Fructus | 5-hydroxy-7-methoxy-2-(3,4,5-trimethoxyphenyl)chromone | Calcium-activated potassium channel subunit alpha 1 |
| Gardenia Fructus | 5-hydroxy-7-methoxy-2-(3,4,5-trimethoxyphenyl)chromone | Calmodulin |
| Gardenia Fructus | 5-hydroxy-7-methoxy-2-(3,4,5-trimethoxyphenyl)chromone | Beta-2 adrenergic receptor |
| Gardenia Fructus | 5-hydroxy-7-methoxy-2-(3,4,5-trimethoxyphenyl)chromone | Beta-secretase |
| Gardenia Fructus | PANA | Prostaglandin G/H synthase 1 |
| Gardenia Fructus | PANA | Prostaglandin G/H synthase 2 |
| Gardenia Fructus | Clorius | Lysozyme |
| Gardenia Fructus | Clorius | Nicotinate-nucleotide--dimethylbenzimidazole phosphoribosyltransferase |
| Gardenia Fructus | Clorius | Alcohol dehydrogenase 1C |
| Gardenia Fructus | methyl (1S,4aS,5R,7S,7aS)-5,7-dihydroxy-7-methyl-1-[(2S,3R,4S,5S,6R)-3,4,5-trihydroxy-6-(hydroxymethyl)oxan-2-yl]oxy-4a,5,6,7a-tetrahydro-1H-cyclopenta[d]pyran-4-carboxylate | Carbonic anhydrase II |
| Gardenia Fructus | methyl (1S,4aS,5R,7S,7aS)-5,7-dihydroxy-7-methyl-1-[(2S,3R,4S,5S,6R)-3,4,5-trihydroxy-6-(hydroxymethyl)oxan-2-yl]oxy-4a,5,6,7a-tetrahydro-1H-cyclopenta[d]pyran-4-carboxylate | Dipeptidyl peptidase IV |
| Gardenia Fructus | shanzhiside methyl ester_qt | Prostaglandin G/H synthase 2 |
| Gardenia Fructus | shanzhiside methyl ester_qt | Carbonic anhydrase II |
| Gardenia Fructus | shanzhiside methyl ester_qt | Trypsin-1 |
| Gardenia Fructus | shanzhiside methyl ester_qt | Glutamate receptor 2 |
| Gardenia Fructus | 3,4-di-o-caffeoylquinic acid | Coagulation factor Xa |
| Gardenia Fructus | 3,4-di-o-caffeoylquinic acid | mRNA of Protein-tyrosine phosphatase, non-receptor type 1 |
| Gardenia Fructus | 3-Methylkempferol | Nitric oxide synthase, inducible |
| Gardenia Fructus | 3-Methylkempferol | Prostaglandin G/H synthase 1 |
| Gardenia Fructus | 3-Methylkempferol | Androgen receptor |
| Gardenia Fructus | 3-Methylkempferol | Prostaglandin G/H synthase 2 |
| Gardenia Fructus | 3-Methylkempferol | Dipeptidyl peptidase IV |
| Gardenia Fructus | 3-Methylkempferol | Mitogen-activated protein kinase 14 |
| Gardenia Fructus | 3-Methylkempferol | Glycogen synthase kinase-3 beta |
| Gardenia Fructus | 3-Methylkempferol | Heat shock protein HSP 90 |
| Gardenia Fructus | 3-Methylkempferol | Cell division protein kinase 2 |
| Gardenia Fructus | 3-Methylkempferol | Phosphatidylinositol-4,5-bisphosphate 3-kinase catalytic subunit, gamma isoform |
| Gardenia Fructus | 3-Methylkempferol | mRNA of PKA Catalytic Subunit C-alpha |
| Gardenia Fructus | desacetyl asperulosidic acid | Carbonic anhydrase II |
| Gardenia Fructus | desacetyl asperulosidic acid_qt | Prostaglandin G/H synthase 2 |
| Gardenia Fructus | desacetyl asperulosidic acid_qt | Carbonic anhydrase II |
| Gardenia Fructus | desacetyl asperulosidic acid_qt | Trypsin-1 |
| Gardenia Fructus | desacetyl asperulosidic acid_qt | Glutamate receptor 2 |

Supplementary table 2. The ingredients and target of herbs from HLJDD.
