# Supplementary material for: Intermodule Coupling Analysis of Huang-Lian-Jie-Du Decoction on Stroke
Source: Front Pharmacol. 2019 Nov 5;10:1288. doi: 10.3389/fphar.2019.01288 (PMC6848980; doi:10.3389/fphar.2019.01288)
Supplement: Table S3 — Target proteins and annotation of HLJDD. [file Table_3.docx]

Supplementary table 3

| **Official symbol** | **Protein annotation** |
| --- | --- |
| GABRA1 | Gamma-aminobutyric acid (GABA) A receptor, alpha 1; Component of the heteropentameric receptor for GABA, the major inhibitory neurotransmitter in the vertebrate brain. Functions also as histamine receptor and mediates cellular responses to histamine. Functions as receptor for diazepines and various anesthetics, such as pentobarbital; these are bound at a separate allosteric effector binding site. Functions as ligand- gated chloride channel (By similarity) |
| PYGM | Phosphorylase, glycogen, muscle; Phosphorylase is an important allosteric enzyme in carbohydrate metabolism. Enzymes from different sources differ in their regulatory mechanisms and in their natural substrates. However, all known phosphorylases share catalytic and structural properties |
| CETP | Cholesteryl ester transfer protein, plasma; Involved in the transfer of neutral lipids, including cholesteryl ester and triglyceride, among lipoprotein particles. Allows the net movement of cholesteryl ester from high density lipoproteins/HDL to triglyceride-rich very low density lipoproteins/VLDL, and the equimolar transport of triglyceride from VLDL to HDL (PubMed:3600759, PubMed:24293641). Regulates the reverse cholesterol transport, by which excess cholesterol is removed from peripheral tissues and returned to the liver for elimination (PubMed:17237796) |
| ESR1 | Estrogen receptor 1; Nuclear hormone receptor. The steroid hormones and their receptors are involved in the regulation of eukaryotic gene expression and affect cellular proliferation and differentiation in target tissues. Ligand-dependent nuclear transactivation involves either direct homodimer binding to a palindromic estrogen response element (ERE) sequence or association with other DNA- binding transcription factors, such as AP-1/c-Jun, c-Fos, ATF-2, Sp1 and Sp3, to mediate ERE-independent signaling. Ligand binding induces a conformational change allowing subsequent or combinatorial [...] |
| ADH1A | Alcohol dehydrogenase 1A (class I), alpha polypeptide |
| SERPIND1 | Serpin peptidase inhibitor, clade D (heparin cofactor), member 1; Thrombin inhibitor activated by the glycosaminoglycans, heparin or dermatan sulfate. In the presence of the latter, HC-II becomes the predominant thrombin inhibitor in place of antithrombin III (AT-III). Also inhibits chymotrypsin, but in a glycosaminoglycan-independent manner |
| MAPK1 | Mitogen-activated protein kinase 1; Serine/threonine kinase which acts as an essential component of the MAP kinase signal transduction pathway. MAPK1/ERK2 and MAPK3/ERK1 are the 2 MAPKs which play an important role in the MAPK/ERK cascade. They participate also in a signaling cascade initiated by activated KIT and KITLG/SCF. Depending on the cellular context, the MAPK/ERK cascade mediates diverse biological functions such as cell growth, adhesion, survival and differentiation through the regulation of transcription, translation, cytoskeletal rearrangements. The MAPK/ERK cascade plays a [...] |
| HMOX1 | Heme oxygenase (decycling) 1; Heme oxygenase cleaves the heme ring at the alpha methene bridge to form biliverdin. Biliverdin is subsequently converted to bilirubin by biliverdin reductase. Under physiological conditions, the activity of heme oxygenase is highest in the spleen, where senescent erythrocytes are sequestrated and destroyed. Exhibits cytoprotective effects since excess of free heme sensitizes cells to undergo apoptosis |
| NFKBIA | Nuclear factor of kappa light polypeptide gene enhancer in B-cells inhibitor, alpha; Inhibits the activity of dimeric NF-kappa-B/REL complexes by trapping REL dimers in the cytoplasm through masking of their nuclear localization signals. On cellular stimulation by immune and proinflammatory responses, becomes phosphorylated promoting ubiquitination and degradation, enabling the dimeric RELA to translocate to the nucleus and activate transcription |
| TIMP1 | TIMP metallopeptidase inhibitor 1; Metalloproteinase inhibitor that functions by forming one to one complexes with target metalloproteinases, such as collagenases, and irreversibly inactivates them by binding to their catalytic zinc cofactor. Acts on MMP1, MMP2, MMP3, MMP7, MMP8, MMP9, MMP10, MMP11, MMP12, MMP13 and MMP16. Does not act on MMP14. Also functions as a growth factor that regulates cell differentiation, migration and cell death and activates cellular signaling cascades via CD63 and ITGB1. Plays a role in integrin signaling. Mediates erythropoiesis in vitro; but, unlike IL3, [...] |
| MMP2 | Matrix metallopeptidase 2 (gelatinase A, 72kDa gelatinase, 72kDa type IV collagenase); Ubiquitinous metalloproteinase that is involved in diverse functions such as remodeling of the vasculature, angiogenesis, tissue repair, tumor invasion, inflammation, and atherosclerotic plaque rupture. As well as degrading extracellular matrix proteins, can also act on several nonmatrix proteins such as big endothelial 1 and beta-type CGRP promoting vasoconstriction. Also cleaves KISS at a Gly-\|-Leu bond. Appears to have a role in myocardial cell death pathways. Contributes to myocardial oxidative s [...] |
| SLC6A2 | Solute carrier family 6 (neurotransmitter transporter, noradrenalin), member 2; Amine transporter. Terminates the action of noradrenaline by its high affinity sodium-dependent reuptake into presynaptic terminals |
| PLAT | Plasminogen activator, tissue; Converts the abundant, but inactive, zymogen plasminogen to plasmin by hydrolyzing a single Arg-Val bond in plasminogen. By controlling plasmin-mediated proteolysis, it plays an important role in tissue remodeling and degradation, in cell migration and many other physiopathological events. Plays a direct role in facilitating neuronal migration |
| GSR | Glutathione reductase; Maintains high levels of reduced glutathione in the cytosol |
| TGFB1 | Transforming growth factor, beta 1; Multifunctional protein that controls proliferation, differentiation and other functions in many cell types. Many cells synthesize TGFB1 and have specific receptors for it. It positively and negatively regulates many other growth factors. It plays an important role in bone remodeling as it is a potent stimulator of osteoblastic bone formation, causing chemotaxis, proliferation and differentiation in committed osteoblasts. Can promote either T- helper 17 cells (Th17) or regulatory T-cells (Treg) lineage differentiation in a concentration-dependent man [...] |
| SLC5A5 | Solute carrier family 5 (sodium iodide symporter), member 5; Mediates iodide uptake in the thyroid gland |
| PON1 | Paraoxonase 1; Hydrolyzes the toxic metabolites of a variety of organophosphorus insecticides. Capable of hydrolyzing a broad spectrum of organophosphate substrates and lactones, and a number of aromatic carboxylic acid esters. Mediates an enzymatic protection of low density lipoproteins against oxidative modification and the consequent series of events leading to atheroma formation |
| PCOLCE | Procollagen C-endopeptidase enhancer; Binds to the C-terminal propeptide of type I procollagen and enhances procollagen C-proteinase activity |
| SERPINE1 | Serpin peptidase inhibitor, clade E (nexin, plasminogen activator inhibitor type 1), member 1; Serine protease inhibitor. This inhibitor acts as 'bait' for tissue plasminogen activator, urokinase, protein C and matriptase-3/TMPRSS7. Its rapid interaction with PLAT may function as a major control point in the regulation of fibrinolysis |
| MPO | Myeloperoxidase; Part of the host defense system of polymorphonuclear leukocytes. It is responsible for microbicidal activity against a wide range of organisms. In the stimulated PMN, MPO catalyzes the production of hypohalous acids, primarily hypochlorous acid in physiologic situations, and other toxic intermediates that greatly enhance PMN microbicidal activity |
| CCL2 | Chemokine (C-C motif) ligand 2; Chemotactic factor that attracts monocytes and basophils but not neutrophils or eosinophils. Augments monocyte anti-tumor activity. Has been implicated in the pathogenesis of diseases characterized by monocytic infiltrates, like psoriasis, rheumatoid arthritis or atherosclerosis. May be involved in the recruitment of monocytes into the arterial wall during the disease process of atherosclerosis |
| COL1A1 | Collagen, type I, alpha 1; Type I collagen is a member of group I collagen (fibrillar forming collagen) |
| SULT1E1 | Sulfotransferase family 1E, estrogen-preferring, member 1; Sulfotransferase that utilizes 3'-phospho-5'-adenylyl sulfate (PAPS) as sulfonate donor to catalyze the sulfate conjugation of estradiol and estrone. May play a role in the regulation of estrogen receptor activity by metabolizing free estradiol. Maximally sulfates beta-estradiol and estrone at concentrations of 20 nM. Also sulfates dehydroepiandrosterone, pregnenolone, ethinylestradiol, equalenin, diethylstilbesterol and 1-naphthol, at significantly higher concentrations; however, cortisol, testosterone and dopamine are not sulfated |
| IL2 | Interleukin 2; Produced by T-cells in response to antigenic or mitogenic stimulation, this protein is required for T-cell proliferation and other activities crucial to regulation of the immune response. Can stimulate B-cells, monocytes, lymphokine- activated killer cells, natural killer cells, and glioma cells |
| CCND1 | Cyclin D1; Regulatory component of the cyclin D1-CDK4 (DC) complex that phosphorylates and inhibits members of the retinoblastoma (RB) protein family including RB1 and regulates the cell-cycle during G(1)/S transition. Phosphorylation of RB1 allows dissociation of the transcription factor E2F from the RB/E2F complex and the subsequent transcription of E2F target genes which are responsible for the progression through the G(1) phase. Hypophosphorylates RB1 in early G(1) phase. Cyclin D-CDK4 complexes are major integrators of various mitogenenic and antimitogenic signals. Also substrate [...] |
| DAO | D-amino-acid oxidase; Regulates the level of the neuromodulator D-serine in the brain. Has high activity towards D-DOPA and contributes to dopamine synthesis. Could act as a detoxifying agent which removes D-amino acids accumulated during aging. Acts on a variety of D- amino acids with a preference for those having small hydrophobic side chains followed by those bearing polar, aromatic, and basic groups. Does not act on acidic amino acids |
| LTA4H | Leukotriene A4 hydrolase; Epoxide hydrolase that catalyzes the final step in the biosynthesis of the proinflammatory mediator leukotriene B4. Has also aminopeptidase activity |
| IFNG | Interferon, gamma; Produced by lymphocytes activated by specific antigens or mitogens. IFN-gamma, in addition to having antiviral activity, has important immunoregulatory functions. It is a potent activator of macrophages, it has antiproliferative effects on transformed cells and it can potentiate the antiviral and antitumor effects of the type I interferons |
| MAPK14 | Mitogen-activated protein kinase 14; Serine/threonine kinase which acts as an essential component of the MAP kinase signal transduction pathway. MAPK14 is one of the four p38 MAPKs which play an important role in the cascades of cellular responses evoked by extracellular stimuli such as proinflammatory cytokines or physical stress leading to direct activation of transcription factors. Accordingly, p38 MAPKs phosphorylate a broad range of proteins and it has been estimated that they may have approximately 200 to 300 substrates each. Some of the targets are downstream kinases which are a [...] |
| IL4 | Interleukin 4; Participates in at least several B-cell activation processes as well as of other cell types (PubMed:3016727). It is a costimulator of DNA-synthesis. It induces the expression of class II MHC molecules on resting B-cells. It enhances both secretion and cell surface expression of IgE and IgG1. It also regulates the expression of the low affinity Fc receptor for IgE (CD23) on both lymphocytes and monocytes. Positively regulates IL31RA expression in macrophages (By similarity) |
| NR3C1 | Nuclear receptor subfamily 3, group C, member 1 (glucocorticoid receptor); Isoform Alpha-D3: Has lowest transcriptional activation activity of all isoforms created by alternative initiation (PubMed:15866175, PubMed:23820903). Has transcriptional repression activity (PubMed:23303127) |
| RBP2 | Retinol binding protein 2, cellular; Intracellular transport of retinol |
| REG1A | Regenerating islet-derived 1 alpha; Might act as an inhibitor of spontaneous calcium carbonate precipitation. May be associated with neuronal sprouting in brain, and with brain and pancreas regeneration |
| ODC1 | Ornithine decarboxylase 1; Key enzyme of polyamine biosynthesis that converts ornithine into putrescine, which is the precursor for the polyamines, spermidine and spermine |
| OPRD1 | Opioid receptor, delta 1; G-protein coupled receptor that functions as receptor for endogenous enkephalins and for a subset of other opioids. Ligand binding causes a conformation change that triggers signaling via guanine nucleotide-binding proteins (G proteins) and modulates the activity of down-stream effectors, such as adenylate cyclase. Signaling leads to the inhibition of adenylate cyclase activity. Inhibits neurotransmitter release by reducing calcium ion currents and increasing potassium ion conductance. Plays a role in the perception of pain and in opiate-mediated analgesia. Pl [...] |
| CTSD | Cathepsin D; Acid protease active in intracellular protein breakdown. Involved in the pathogenesis of several diseases such as breast cancer and possibly Alzheimer disease |
| ABCG2 | ATP-binding cassette, sub-family G (WHITE), member 2; High-capacity urate exporter functioning in both renal and extrarenal urate excretion. Plays a role in porphyrin homeostasis as it is able to mediates the export of protoporhyrin IX (PPIX) both from mitochondria to cytosol and from cytosol to extracellular space, and cellular export of hemin, and heme. Xenobiotic transporter that may play an important role in the exclusion of xenobiotics from the brain. Appears to play a major role in the multidrug resistance phenotype of several cancer cell lines. Implicated in the efflux of numero [...] |
| ALG5 | Asparagine-linked glycosylation 5, dolichyl-phosphate beta-glucosyltransferase homolog (S. cerevisiae) |
| CAT | Catalase; Occurs in almost all aerobically respiring organisms and serves to protect cells from the toxic effects of hydrogen peroxide. Promotes growth of cells including T-cells, B-cells, myeloid leukemia cells, melanoma cells, mastocytoma cells and normal and transformed fibroblast cells |
| GSTM2 | Glutathione S-transferase mu 2 (muscle); Conjugation of reduced glutathione to a wide number of exogenous and endogenous hydrophobic electrophiles |
| AHR | Aryl hydrocarbon receptor; Ligand-activated transcriptional activator. Binds to the XRE promoter region of genes it activates. Activates the expression of multiple phase I and II xenobiotic chemical metabolizing enzyme genes (such as the CYP1A1 gene). Mediates biochemical and toxic effects of halogenated aromatic hydrocarbons. Involved in cell-cycle regulation. Likely to play an important role in the development and maturation of many tissues. Regulates the circadian clock by inhibiting the basal and circadian expression of the core circadian component PER1. Inhibits PER1 by repressing [...] |
| CDKN1A | Cyclin-dependent kinase inhibitor 1A (p21, Cip1); May be the important intermediate by which p53/TP53 mediates its role as an inhibitor of cellular proliferation in response to DNA damage. Binds to and inhibits cyclin-dependent kinase activity, preventing phosphorylation of critical cyclin- dependent kinase substrates and blocking cell cycle progression. Functions in the nuclear localization and assembly of cyclin D- CDK4 complex and promotes its kinase activity towards RB1. At higher stoichiometric ratios, inhibits the kinase activity of the cyclin D-CDK4 complex |
| SLC22A5 | Solute carrier family 22 (organic cation/carnitine transporter), member 5; Sodium-ion dependent, high affinity carnitine transporter. Involved in the active cellular uptake of carnitine. Transports one sodium ion with one molecule of carnitine. Also transports organic cations such as tetraethylammonium (TEA) without the involvement of sodium. Also relative uptake activity ratio of carnitine to TEA is 11.3 |
| IRF1 | Interferon regulatory factor 1; Transcriptional regulator which displays a remarkable functional diversity in the regulation of cellular responses. These include the regulation of IFN and IFN-inducible genes, host response to viral and bacterial infections, regulation of many genes expressed during hematopoiesis, inflammation, immune responses and cell proliferation and differentiation, regulation of the cell cycle and induction of growth arrest and programmed cell death following DNA damage. Stimulates both innate and acquired immune responses through the activation of specific target [...] |
| ELK1 | ELK1, member of ETS oncogene family; Transcription factor that binds to purine-rich DNA sequences. Forms a ternary complex with SRF and the ETS and SRF motifs of the serum response element (SRE) on the promoter region of immediate early genes such as FOS and IER2. Induces target gene transcription upon JNK-signaling pathway stimulation (By similarity) |
| HSPB1 | Heat shock 27kDa protein 1; Involved in stress resistance and actin organization |
| QTRT1 | Queuine tRNA-ribosyltransferase 1; Interacts with QTRTD1 to form an active queuine tRNA- ribosyltransferase. This enzyme exchanges queuine for the guanine at the wobble position of tRNAs with GU(N) anticodons (tRNA-Asp, -Asn, -His and -Tyr), thereby forming the hypermodified nucleoside queuosine (Q) (7-(((4,5-cis-dihydroxy-2-cyclopenten-1- yl)amino)methyl)-7-deazaguanosine) (By similarity) |
| INS | Insulin; Insulin decreases blood glucose concentration. It increases cell permeability to monosaccharides, amino acids and fatty acids. It accelerates glycolysis, the pentose phosphate cycle, and glycogen synthesis in liver |
| ALOX12 | Arachidonate 12-lipoxygenase; Non-heme iron-containing dioxygenase that catalyzes the stereo-specific peroxidation of free and esterified polyunsaturated fatty acids generating a spectrum of bioactive lipid mediators. Mainly converts arachidonic acid to (12S)- hydroperoxyeicosatetraenoic acid/(12S)-HPETE but can also metabolize linoleic acid. Has a dual activity since it also converts leukotriene A4/LTA4 into both the bioactive lipoxin A4/LXA4 and lipoxin B4/LXB4. Through the production of specific bioactive lipids like (12S)-HPETE it regulates different biological processes including [...] |
| GFAP | Glial fibrillary acidic protein; GFAP, a class-III intermediate filament, is a cell- specific marker that, during the development of the central nervous system, distinguishes astrocytes from other glial cells |
| G6PC | Glucose-6-phosphatase, catalytic subunit; Hydrolyzes glucose-6-phosphate to glucose in the endoplasmic reticulum. Forms with the glucose-6-phosphate transporter (SLC37A4/G6PT) the complex responsible for glucose production through glycogenolysis and gluconeogenesis. Hence, it is the key enzyme in homeostatic regulation of blood glucose levels |
| FUS | Fused in sarcoma; Binds both single-stranded and double-stranded DNA and promotes ATP-independent annealing of complementary single- stranded DNAs and D-loop formation in superhelical double-stranded DNA. May play a role in maintenance of genomic integrity |
| CRP | C-reactive protein, pentraxin-related; Displays several functions associated with host defense: it promotes agglutination, bacterial capsular swelling, phagocytosis and complement fixation through its calcium-dependent binding to phosphorylcholine. Can interact with DNA and histones and may scavenge nuclear material released from damaged circulating cells |
| CHRM3 | Cholinergic receptor, muscarinic 3; The muscarinic acetylcholine receptor mediates various cellular responses, including inhibition of adenylate cyclase, breakdown of phosphoinositides and modulation of potassium channels through the action of G proteins. Primary transducing effect is Pi turnover |
| CCNB1 | Cyclin B1; Essential for the control of the cell cycle at the G2/M (mitosis) transition |
| CDK7 | Cyclin-dependent kinase 7; Serine/threonine kinase involved in cell cycle control and in RNA polymerase II-mediated RNA transcription. Cyclin- dependent kinases (CDKs) are activated by the binding to a cyclin and mediate the progression through the cell cycle. Each different complex controls a specific transition between 2 subsequent phases in the cell cycle. Required for both activation and complex formation of CDK1/cyclin-B during G2-M transition, and for activation of CDK2/cyclins during G1-S transition (but not complex formation). CDK7 is the catalytic subunit of the CDK-activating [...] |
| APC | Adenomatous polyposis coli; Tumor suppressor. Promotes rapid degradation of CTNNB1 and participates in Wnt signaling as a negative regulator. APC activity is correlated with its phosphorylation state. Activates the GEF activity of SPATA13 and ARHGEF4. Plays a role in hepatocyte growth factor (HGF)-induced cell migration. Required for MMP9 up-regulation via the JNK signaling pathway in colorectal tumor cells. Acts as a mediator of ERBB2-dependent stabilization of microtubules at the cell cortex. It is required for the localization of MACF1 to the cell membrane and this localization of M [...] |
| CDK4 | Cyclin-dependent kinase 4; Ser/Thr-kinase component of cyclin D-CDK4 (DC) complexes that phosphorylate and inhibit members of the retinoblastoma (RB) protein family including RB1 and regulate the cell-cycle during G(1)/S transition. Phosphorylation of RB1 allows dissociation of the transcription factor E2F from the RB/E2F complexes and the subsequent transcription of E2F target genes which are responsible for the progression through the G(1) phase. Hypophosphorylates RB1 in early G(1) phase. Cyclin D-CDK4 complexes are major integrators of various mitogenenic and antimitogenic signals. [...] |
| SMPD2 | Sphingomyelin phosphodiesterase 2, neutral membrane (neutral sphingomyelinase); Converts sphingomyelin to ceramide. Hydrolyze 1-acyl-2- lyso-sn-glycero-3-phosphocholine (lyso-PC) and 1-O-alkyl-2-lyso- sn-glycero-3-phosphocholine (lyso-platelet-activating factor). The physiological substrate seems to be Lyso-PAF |
| IL6 | Interleukin 6 (interferon, beta 2); Cytokine with a wide variety of biological functions. It is a potent inducer of the acute phase response. Plays an essential role in the final differentiation of B-cells into Ig- secreting cells Involved in lymphocyte and monocyte differentiation. Acts on B-cells, T-cells, hepatocytes, hematopoietic progenitor cells and cells of the CNS. Required for the generation of T(H)17 cells. Also acts as a myokine. It is discharged into the bloodstream after muscle contraction and acts to increase the breakdown of fats and to improve insulin resistance. It ind [...] |
| TLR2 | Toll-like receptor 2; Cooperates with LY96 to mediate the innate immune response to bacterial lipoproteins and other microbial cell wall components. Cooperates with TLR1 or TLR6 to mediate the innate immune response to bacterial lipoproteins or lipopeptides (PubMed:17889651). Acts via MYD88 and TRAF6, leading to NF-kappa-B activation, cytokine secretion and the inflammatory response. May also promote apoptosis in response to lipoproteins (PubMed:10426996). Recognizes mycoplasmal macrophage-activating lipopeptide-2kD (MALP-2), soluble tuberculosis factor (STF), phenol-soluble modulin (P [...] |
| CYP19A1 | Cytochrome P450, family 19, subfamily A, polypeptide 1; Catalyzes the formation of aromatic C18 estrogens from C19 androgens |
| CYP1B1 | Cytochrome P450, family 1, subfamily B, polypeptide 1; Cytochromes P450 are a group of heme-thiolate monooxygenases. In liver microsomes, this enzyme is involved in an NADPH-dependent electron transport pathway. It oxidizes a variety of structurally unrelated compounds, including steroids, fatty acids, retinoid and xenobiotics. Preferentially oxidizes 17beta- estradiol to the carcinogenic 4-hydroxy derivative, and a variety of procarcinogenic compounds to their activated forms, including polycyclic aromatic hydrocarbons. Promotes angiogenesis by removing cellular oxygenation products, [...] |
| CYP2C9 | Cytochrome P450, family 2, subfamily C, polypeptide 9; Cytochromes P450 are a group of heme-thiolate monooxygenases. In liver microsomes, this enzyme is involved in an NADPH-dependent electron transport pathway. It oxidizes a variety of structurally unrelated compounds, including steroids, fatty acids, and xenobiotics (PubMed:25994031). This enzyme contributes to the wide pharmacokinetics variability of the metabolism of drugs such as S-warfarin, diclofenac, phenytoin, tolbutamide and losartan (PubMed:25994031) |
| CCND2 | Cyclin D2; Regulatory component of the cyclin D2-CDK4 (DC) complex that phosphorylates and inhibits members of the retinoblastoma (RB) protein family including RB1 and regulates the cell-cycle during G(1)/S transition. Phosphorylation of RB1 allows dissociation of the transcription factor E2F from the RB/E2F complex and the subsequent transcription of E2F target genes which are responsible for the progression through the G(1) phase. Hypophosphorylates RB1 in early G(1) phase. Cyclin D-CDK4 complexes are major integrators of various mitogenenic and antimitogenic signals. Also substrate [...] |
| LYZ | Lysozyme; Lysozymes have primarily a bacteriolytic function; those in tissues and body fluids are associated with the monocyte- macrophage system and enhance the activity of immunoagents |
| SLC6A4 | Solute carrier family 6 (neurotransmitter transporter, serotonin), member 4; Serotonin transporter whose primary function in the central nervous system involves the regulation of serotonergic signaling via transport of serotonin molecules from the synaptic cleft back into the pre-synaptic terminal for re-utilization. Plays a key role in mediating regulation of the availability of serotonin to other receptors of serotonergic systems. Terminates the action of serotonin and recycles it in a sodium-dependent manner |
| AAGAB | Alpha- and gamma-adaptin binding protein; May be involved in endocytic recycling of growth factor receptors such as EGFR |
| ITGB3 | Integrin, beta 3 (platelet glycoprotein IIIa, antigen CD61); Integrin alpha-V/beta-3 (ITGAV:ITGB3) is a receptor for cytotactin, fibronectin, laminin, matrix metalloproteinase-2, osteopontin, osteomodulin, prothrombin, thrombospondin, vitronectin and von Willebrand factor. Integrin alpha-IIb/beta-3 (ITGA2B:ITGB3) is a receptor for fibronectin, fibrinogen, plasminogen, prothrombin, thrombospondin and vitronectin. Integrins alpha-IIb/beta-3 and alpha-V/beta-3 recognize the sequence R-G-D in a wide array of ligands. Integrin alpha- IIb/beta-3 recognizes the sequence H-H-L-G-G-G-A-K-Q-A-G- [...] |
| SMAD7 | SMAD family member 7; Antagonist of signaling by TGF-beta (transforming growth factor) type 1 receptor superfamily members; has been shown to inhibit TGF-beta (Transforming growth factor) and activin signaling by associating with their receptors thus preventing SMAD2 access. Functions as an adapter to recruit SMURF2 to the TGF-beta receptor complex. Also acts by recruiting the PPP1R15A- PP1 complex to TGFBR1, which promotes its dephosphorylation. Positively regulates PDPK1 kinase activity by stimulating its dissociation from the 14-3-3 protein YWHAQ which acts as a negative regulator ( [...] |
| KCNH2 | Potassium voltage-gated channel, subfamily H (eag-related), member 2; Pore-forming (alpha) subunit of voltage-gated inwardly rectifying potassium channel. Channel properties are modulated by cAMP and subunit assembly. Mediates the rapidly activating component of the delayed rectifying potassium current in heart (IKr). Isoforms USO have no channel activity by themself, but modulates channel characteristics by forming heterotetramers with other isoforms which are retained intracellularly and undergo ubiquitin-dependent degradation |
| ITGA2B | Integrin, alpha 2b (platelet glycoprotein IIb of IIb/IIIa complex, antigen CD41); Integrin alpha-IIb/beta-3 is a receptor for fibronectin, fibrinogen, plasminogen, prothrombin, thrombospondin and vitronectin. It recognizes the sequence R-G-D in a wide array of ligands. It recognizes the sequence H-H-L-G-G-G-A-K-Q-A-G-D-V in fibrinogen gamma chain. Following activation integrin alpha- IIb/beta-3 brings about platelet/platelet interaction through binding of soluble fibrinogen. This step leads to rapid platelet aggregation which physically plugs ruptured endothelial cell surface |
| TEP1 | Telomerase-associated protein 1; Component of the telomerase ribonucleoprotein complex that is essential for the replication of chromosome termini. Also component of the ribonucleoprotein vaults particle, a multi- subunit structure involved in nucleo-cytoplasmic transport. Responsible for the localizing and stabilizing vault RNA (vRNA) association in the vault ribonucleoprotein particle. Binds to TERC (By similarity) |
| PPARA | Peroxisome proliferator-activated receptor alpha; Ligand-activated transcription factor. Key regulator of lipid metabolism. Activated by the endogenous ligand 1-palmitoyl- 2-oleoyl-sn-glycerol-3-phosphocholine (16:0/18:1-GPC). Activated by oleylethanolamide, a naturally occurring lipid that regulates satiety. Receptor for peroxisome proliferators such as hypolipidemic drugs and fatty acids. Regulates the peroxisomal beta-oxidation pathway of fatty acids. Functions as transcription activator for the ACOX1 and P450 genes. Transactivation activity requires heterodimerization with RXRA and [...] |
| HIRA | HIR histone cell cycle regulation defective homolog A (S. cerevisiae); Cooperates with ASF1A to promote replication-independent chromatin assembly. Required for the periodic repression of histone gene transcription during the cell cycle. Required for the formation of senescence-associated heterochromatin foci (SAHF) and efficient senescence-associated cell cycle exit |
| EP300 | E1A binding protein p300; Functions as histone acetyltransferase and regulates transcription via chromatin remodeling. Acetylates all four core histones in nucleosomes. Histone acetylation gives an epigenetic tag for transcriptional activation. Mediates cAMP-gene regulation by binding specifically to phosphorylated CREB protein. Mediates acetylation of histone H3 at 'Lys-122' (H3K122ac), a modification that localizes at the surface of the histone octamer and stimulates transcription, possibly by promoting nucleosome instability. Mediates acetylation of histone H3 at 'Lys-27' (H3K27ac). [...] |
| TYR | Tyrosinase; This is a copper-containing oxidase that functions in the formation of pigments such as melanins and other polyphenolic compounds. Catalyzes the rate-limiting conversions of tyrosine to DOPA, DOPA to DOPA-quinone and possibly 5,6-dihydroxyindole to indole-5,6 quinone |
| IL1A | Interleukin 1, alpha; Produced by activated macrophages, IL-1 stimulates thymocyte proliferation by inducing IL-2 release, B-cell maturation and proliferation, and fibroblast growth factor activity. IL-1 proteins are involved in the inflammatory response, being identified as endogenous pyrogens, and are reported to stimulate the release of prostaglandin and collagenase from synovial cells |
| IL1B | Interleukin 1, beta; Potent proinflammatory cytokine. Initially discovered as the major endogenous pyrogen, induces prostaglandin synthesis, neutrophil influx and activation, T-cell activation and cytokine production, B-cell activation and antibody production, and fibroblast proliferation and collagen production. Promotes Th17 differentiation of T-cells |
| PRKCG | Protein kinase C, gamma; Calcium-activated, phospholipid- and diacylglycerol (DAG)-dependent serine/threonine-protein kinase that plays diverse roles in neuronal cells and eye tissues, such as regulation of the neuronal receptors GRIA4/GLUR4 and GRIN1/NMDAR1, modulation of receptors and neuronal functions related to sensitivity to opiates, pain and alcohol, mediation of synaptic function and cell survival after ischemia, and inhibition of gap junction activity after oxidative stress. Binds and phosphorylates GRIA4/GLUR4 glutamate receptor and regulates its function by increasing plasma [...] |
| ELANE | Elastase, neutrophil expressed; Modifies the functions of natural killer cells, monocytes and granulocytes. Inhibits C5a-dependent neutrophil enzyme release and chemotaxis |
| SELP | Selectin P (granule membrane protein 140kDa, antigen CD62); Ca(2+)-dependent receptor for myeloid cells that binds to carbohydrates on neutrophils and monocytes. Mediates the interaction of activated endothelial cells or platelets with leukocytes. The ligand recognized is sialyl-Lewis X. Mediates rapid rolling of leukocyte rolling over vascular surfaces during the initial steps in inflammation through interaction with PSGL1 |
| KDR | Kinase insert domain receptor (a type III receptor tyrosine kinase); Tyrosine-protein kinase that acts as a cell-surface receptor for VEGFA, VEGFC and VEGFD. Plays an essential role in the regulation of angiogenesis, vascular development, vascular permeability, and embryonic hematopoiesis. Promotes proliferation, survival, migration and differentiation of endothelial cells. Promotes reorganization of the actin cytoskeleton. Isoforms lacking a transmembrane domain, such as isoform 2 and isoform 3, may function as decoy receptors for VEGFA, VEGFC and/or VEGFD. Isoform 2 plays an importan [...] |
| LCAT | Lecithin-cholesterol acyltransferase; Central enzyme in the extracellular metabolism of plasma lipoproteins. Synthesized mainly in the liver and secreted into plasma where it converts cholesterol and phosphatidylcholines (lecithins) to cholesteryl esters and lysophosphatidylcholines on the surface of high and low density lipoproteins (HDLs and LDLs) (PubMed:10329423, PubMed:19065001, PubMed:26195816). The cholesterol ester is then transported back to the liver. Has a preference for plasma 16:0-18:2 or 18:O-18:2 phosphatidylcholines (PubMed:8820107). Also produced in the brain by primar [...] |
| FCER2 | Fc fragment of IgE, low affinity II, receptor for (CD23); Low-affinity receptor for immunoglobulin E (IgE) and CR2/CD21. Has essential roles in the regulation of IgE production and in the differentiation of B-cells (it is a B-cell-specific antigen) |
| ATF2 | Activating transcription factor 2; Transcriptional activator which regulates the transcription of various genes, including those involved in anti- apoptosis, cell growth, and DNA damage response. Dependent on its binding partner, binds to CRE (cAMP response element) consensus sequences (5'-TGACGTCA-3') or to AP-1 (activator protein 1) consensus sequences (5'-TGACTCA-3'). In the nucleus, contributes to global transcription and the DNA damage response, in addition to specific transcriptional activities that are related to cell development, proliferation and death. In the cytoplasm, inter [...] |
| CD80 | CD80 molecule; Involved in the costimulatory signal essential for T- lymphocyte activation. T-cell proliferation and cytokine production is induced by the binding of CD28, binding to CTLA-4 has opposite effects and inhibits T-cell activation |
| BCHE | Butyrylcholinesterase; Esterase with broad substrate specificity. Contributes to the inactivation of the neurotransmitter acetylcholine. Can degrade neurotoxic organophosphate esters |
| GRIA2 | Glutamate receptor, ionotropic, AMPA 2; Receptor for glutamate that functions as ligand-gated ion channel in the central nervous system and plays an important role in excitatory synaptic transmission. L-glutamate acts as an excitatory neurotransmitter at many synapses in the central nervous system. Binding of the excitatory neurotransmitter L- glutamate induces a conformation change, leading to the opening of the cation channel, and thereby converts the chemical signal to an electrical impulse. The receptor then desensitizes rapidly and enters a transient inactive state, characterized [...] |
| FGF2 | Fibroblast growth factor 2 (basic); Plays an important role in the regulation of cell survival, cell division, angiogenesis, cell differentiation and cell migration. Functions as potent mitogen in vitro |
| PSMD3 | Proteasome (prosome, macropain) 26S subunit, non-ATPase, 3; Acts as a regulatory subunit of the 26 proteasome which is involved in the ATP-dependent degradation of ubiquitinated proteins |
| STAT3 | Signal transducer and activator of transcription 3 (acute-phase response factor); Signal transducer and transcription activator that mediates cellular responses to interleukins, KITLG/SCF, LEP and other growth factors. Once activated, recruits coactivators, such as NCOA1 or MED1, to the promoter region of the target gene (PubMed:17344214). May mediate cellular responses to activated FGFR1, FGFR2, FGFR3 and FGFR4. Binds to the interleukin-6 (IL-6)- responsive elements identified in the promoters of various acute- phase protein genes. Activated by IL31 through IL31RA. Involved in cell cy [...] |
| FOSL2 | FOS-like antigen 2; Controls osteoclast survival and size. As a dimer with JUN, activates LIF transcription. Activates CEBPB transcription in PGE2-activated osteoblasts |
| ICAM1 | Intercellular adhesion molecule 1; ICAM proteins are ligands for the leukocyte adhesion protein LFA-1 (integrin alpha-L/beta-2). During leukocyte trans- endothelial migration, ICAM1 engagement promotes the assembly of endothelial apical cups through ARHGEF26/SGEF and RHOG activation |
| ENPEP | Glutamyl aminopeptidase (aminopeptidase A); Appears to have a role in the catabolic pathway of the renin-angiotensin system. Probably plays a role in regulating growth and differentiation of early B-lineage cells |
| EGF | Epidermal growth factor; EGF stimulates the growth of various epidermal and epithelial tissues in vivo and in vitro and of some fibroblasts in cell culture. Magnesiotropic hormone that stimulates magnesium reabsorption in the renal distal convoluted tubule via engagement of EGFR and activation of the magnesium channel TRPM6. Can induce neurite outgrowth in motoneurons of the pond snail Lymnaea stagnalis in vitro (PubMed:10964941) |
| PRDX5 | Peroxiredoxin 5; Reduces hydrogen peroxide and alkyl hydroperoxides with reducing equivalents provided through the thioredoxin system. Involved in intracellular redox signaling |
| CDK6 | Cyclin-dependent kinase 6; Serine/threonine-protein kinase involved in the control of the cell cycle and differentiation; promotes G1/S transition. Phosphorylates pRB/RB1 and NPM1. Interacts with D-type G1 cyclins during interphase at G1 to form a pRB/RB1 kinase and controls the entrance into the cell cycle. Involved in initiation and maintenance of cell cycle exit during cell differentiation; prevents cell proliferation and regulates negatively cell differentiation, but is required for the proliferation of specific cell types (e.g. erythroid and hematopoietic cells). Essential for cel [...] |
| CDK2 | Cyclin-dependent kinase 2; Serine/threonine-protein kinase involved in the control of the cell cycle; essential for meiosis, but dispensable for mitosis. Phosphorylates CTNNB1, USP37, p53/TP53, NPM1, CDK7, RB1, BRCA2, MYC, NPAT, EZH2. Interacts with cyclins A, B1, B3, D, or E. Triggers duplication of centrosomes and DNA. Acts at the G1-S transition to promote the E2F transcriptional program and the initiation of DNA synthesis, and modulates G2 progression; controls the timing of entry into mitosis/meiosis by controlling the subsequent activation of cyclin B/CDK1 by phosphorylation, and [...] |
| ERBB3 | V-erb-b2 erythroblastic leukemia viral oncogene homolog 3 (avian); Binds and is activated by neuregulins and NTAK. May also be activated by CSPG5 |
| RB1 | Retinoblastoma 1; Key regulator of entry into cell division that acts as a tumor suppressor. Promotes G0-G1 transition when phosphorylated by CDK3/cyclin-C. Acts as a transcription repressor of E2F1 target genes. The underphosphorylated, active form of RB1 interacts with E2F1 and represses its transcription activity, leading to cell cycle arrest. Directly involved in heterochromatin formation by maintaining overall chromatin structure and, in particular, that of constitutive heterochromatin by stabilizing histone methylation. Recruits and targets histone methyltransferases SUV39H1, KMT [...] |
| IGF1R | Insulin-like growth factor 1 receptor; Receptor tyrosine kinase which mediates actions of insulin-like growth factor 1 (IGF1). Binds IGF1 with high affinity and IGF2 and insulin (INS) with a lower affinity. The activated IGF1R is involved in cell growth and survival control. IGF1R is crucial for tumor transformation and survival of malignant cell. Ligand binding activates the receptor kinase, leading to receptor autophosphorylation, and tyrosines phosphorylation of multiple substrates, that function as signaling adapter proteins including, the insulin-receptor substrates (IRS1/2), Shc [...] |
| TP53 | Tumor protein p53; Acts as a tumor suppressor in many tumor types; induces growth arrest or apoptosis depending on the physiological circumstances and cell type. Involved in cell cycle regulation as a trans-activator that acts to negatively regulate cell division by controlling a set of genes required for this process. One of the activated genes is an inhibitor of cyclin-dependent kinases. Apoptosis induction seems to be mediated either by stimulation of BAX and FAS antigen expression, or by repression of Bcl-2 expression. In cooperation with mitochondrial PPIF is involved in activatin [...] |
| ERBB2 | V-erb-b2 erythroblastic leukemia viral oncogene homolog 2, neuro/glioblastoma derived oncogene homolog (avian); Protein tyrosine kinase that is part of several cell surface receptor complexes, but that apparently needs a coreceptor for ligand binding. Essential component of a neuregulin-receptor complex, although neuregulins do not interact with it alone. GP30 is a potential ligand for this receptor. Regulates outgrowth and stabilization of peripheral microtubules (MTs). Upon ERBB2 activation, the MEMO1-RHOA-DIAPH1 signaling pathway elicits the phosphorylation and thus the inhibition o [...] |
| SOD1 | Superoxide dismutase 1, soluble; Destroys radicals which are normally produced within the cells and which are toxic to biological systems |
| AKT1 | V-akt murine thymoma viral oncogene homolog 1; AKT1 is one of 3 closely related serine/threonine- protein kinases (AKT1, AKT2 and AKT3) called the AKT kinase, and which regulate many processes including metabolism, proliferation, cell survival, growth and angiogenesis. This is mediated through serine and/or threonine phosphorylation of a range of downstream substrates. Over 100 substrate candidates have been reported so far, but for most of them, no isoform specificity has been reported. AKT is responsible of the regulation of glucose uptake by mediating insulin-induced translocation o [...] |
| SLC6A3 | Solute carrier family 6 (neurotransmitter transporter, dopamine), member 3; Amine transporter. Terminates the action of dopamine by its high affinity sodium-dependent reuptake into presynaptic terminals |
| CALM2 | Calmodulin 2 (phosphorylase kinase, delta); Calmodulin mediates the control of a large number of enzymes, ion channels, aquaporins and other proteins by Ca(2+). Among the enzymes to be stimulated by the calmodulin-Ca(2+) complex are a number of protein kinases and phosphatases. Together with CCP110 and centrin, is involved in a genetic pathway that regulates the centrosome cycle and progression through cytokinesis |
| DNPEP | Aspartyl aminopeptidase; Aminopeptidase with specificity towards an acidic amino acid at the N-terminus. Likely to play an important role in intracellular protein and peptide metabolism |
| CCNA2 | Cyclin A2; Essential for the control of the cell cycle at the G1/S (start) and the G2/M (mitosis) transitions |
| RASA1 | RAS p21 protein activator (GTPase activating protein) 1; Inhibitory regulator of the Ras-cyclic AMP pathway. Stimulates the GTPase of normal but not oncogenic Ras p21; this stimulation may be further increased in the presence of NCK1 |
| EGFR | Epidermal growth factor receptor; Receptor tyrosine kinase binding ligands of the EGF family and activating several signaling cascades to convert extracellular cues into appropriate cellular responses. Known ligands include EGF, TGFA/TGF-alpha, amphiregulin, epigen/EPGN, BTC/betacellulin, epiregulin/EREG and HBEGF/heparin-binding EGF. Ligand binding triggers receptor homo- and/or heterodimerization and autophosphorylation on key cytoplasmic residues. The phosphorylated receptor recruits adapter proteins like GRB2 which in turn activates complex downstream signaling cascades. Activates [...] |
| HTR2C | 5-hydroxytryptamine (serotonin) receptor 2C, G protein-coupled; G-protein coupled receptor for 5-hydroxytryptamine (serotonin). Also functions as a receptor for various drugs and psychoactive substances, including ergot alkaloid derivatives, 1- 2,5,-dimethoxy-4-iodophenyl-2-aminopropane (DOI) and lysergic acid diethylamide (LSD). Ligand binding causes a conformation change that triggers signaling via guanine nucleotide-binding proteins (G proteins) and modulates the activity of down-stream effectors. Beta-arrestin family members inhibit signaling via G proteins and mediate activation o [...] |
| NTRK2 | Neurotrophic tyrosine kinase, receptor, type 2; Receptor tyrosine kinase involved in the development and the maturation of the central and the peripheral nervous systems through regulation of neuron survival, proliferation, migration, differentiation, and synapse formation and plasticity. Receptor for BDNF/brain-derived neurotrophic factor and NTF4/neurotrophin- 4. Alternatively can also bind NTF3/neurotrophin-3 which is less efficient in activating the receptor but regulates neuron survival through NTRK2. Upon ligand-binding, undergoes homodimerization, autophosphorylation and activat [...] |
| MMP10 | Matrix metallopeptidase 10 (stromelysin 2); Can degrade fibronectin, gelatins of type I, III, IV, and V; weakly collagens III, IV, and V. Activates procollagenase |
| ADRA2A | Adrenoceptor alpha 2A; Alpha-2 adrenergic receptors mediate the catecholamine- induced inhibition of adenylate cyclase through the action of G proteins. The rank order of potency for agonists of this receptor is oxymetazoline > clonidine > epinephrine > norepinephrine > phenylephrine > dopamine > p-synephrine > p-tyramine > serotonin = p-octopamine. For antagonists, the rank order is yohimbine > phentolamine = mianserine > chlorpromazine = spiperone = prazosin > propanolol > alprenolol = pindolol |
| GJA1 | Gap junction protein, alpha 1, 43kDa; Gap junction protein that acts as a regulator of bladder capacity. A gap junction consists of a cluster of closely packed pairs of transmembrane channels, the connexons, through which materials of low MW diffuse from one cell to a neighboring cell. May play a critical role in the physiology of hearing by participating in the recycling of potassium to the cochlear endolymph. Negative regulator of bladder functional capacity: acts by enhancing intercellular electrical and chemical transmission, thus sensitizing bladder muscles to cholinergic neural s [...] |
| UCHL1 | Ubiquitin carboxyl-terminal esterase L1 (ubiquitin thiolesterase); Ubiquitin-protein hydrolase involved both in the processing of ubiquitin precursors and of ubiquitinated proteins. This enzyme is a thiol protease that recognizes and hydrolyzes a peptide bond at the C-terminal glycine of ubiquitin. Also binds to free monoubiquitin and may prevent its degradation in lysosomes. The homodimer may have ATP-independent ubiquitin ligase activity |
| CA2 | Carbonic anhydrase II; Essential for bone resorption and osteoclast differentiation (By similarity). Reversible hydration of carbon dioxide. Can hydrate cyanamide to urea. Involved in the regulation of fluid secretion into the anterior chamber of the eye. Contributes to intracellular pH regulation in the duodenal upper villous epithelium during proton-coupled peptide absorption. Stimulates the chloride-bicarbonate exchange activity of SLC26A6 |
| AKR1B1 | Aldo-keto reductase family 1, member B1 (aldose reductase); Catalyzes the NADPH-dependent reduction of a wide variety of carbonyl-containing compounds to their corresponding alcohols with a broad range of catalytic efficiencies |
| PPARG | Peroxisome proliferator-activated receptor gamma; Nuclear receptor that binds peroxisome proliferators such as hypolipidemic drugs and fatty acids. Once activated by a ligand, the nuclear receptor binds to DNA specific PPAR response elements (PPRE) and modulates the transcription of its target genes, such as acyl-CoA oxidase. It therefore controls the peroxisomal beta-oxidation pathway of fatty acids. Key regulator of adipocyte differentiation and glucose homeostasis. ARF6 acts as a key regulator of the tissue-specific adipocyte P2 (aP2) enhancer. Acts as a critical regulator of gut ho [...] |
| HMGCR | 3-hydroxy-3-methylglutaryl-CoA reductase; Transmembrane glycoprotein that is the rate-limiting enzyme in cholesterol biosynthesis as well as in the biosynthesis of nonsterol isoprenoids that are essential for normal cell function including ubiquinone and geranylgeranyl proteins |
| NCF1 | Neutrophil cytosolic factor 1; NCF2, NCF1, and a membrane bound cytochrome b558 are required for activation of the latent NADPH oxidase (necessary for superoxide production) |
| HK2 | Hexokinase 2 |
| PCYT1A | Phosphate cytidylyltransferase 1, choline, alpha; Controls phosphatidylcholine synthesis |
| CCL16 | Chemokine (C-C motif) ligand 16; Shows chemotactic activity for lymphocytes and monocytes but not neutrophils. Also shows potent myelosuppressive activity, suppresses proliferation of myeloid progenitor cells. Recombinant SCYA16 shows chemotactic activity for monocytes and THP-1 monocytes, but not for resting lymphocytes and neutrophils. Induces a calcium flux in THP-1 cells that were desensitized by prior expression to RANTES |
| BAX | BCL2-associated X protein; Accelerates programmed cell death by binding to, and antagonizing the apoptosis repressor BCL2 or its adenovirus homolog E1B 19k protein. Under stress conditions, undergoes a conformation change that causes translocation to the mitochondrion membrane, leading to the release of cytochrome c that then triggers apoptosis. Promotes activation of CASP3, and thereby apoptosis |
| PSME3 | Proteasome (prosome, macropain) activator subunit 3 (PA28 gamma; Ki); Subunit of the 11S REG-gamma (also called PA28-gamma) proteasome regulator, a doughnut-shaped homoheptamer which associates with the proteasome. 11S REG-gamma activates the trypsin-like catalytic subunit of the proteasome but inhibits the chymotrypsin-like and postglutamyl-preferring (PGPH) subunits. Facilitates the MDM2-p53/TP53 interaction which promotes ubiquitination- and MDM2-dependent proteasomal degradation of p53/TP53, limiting its accumulation and resulting in inhibited apoptosis after DNA damage. May also b [...] |
| VCAM1 | Vascular cell adhesion molecule 1; Important in cell-cell recognition. Appears to function in leukocyte-endothelial cell adhesion. Interacts with integrin alpha-4/beta-1 (ITGA4/ITGB1) on leukocytes, and mediates both adhesion and signal transduction. The VCAM1/ITGA4/ITGB1 interaction may play a pathophysiologic role both in immune responses and in leukocyte emigration to sites of inflammation |
| ALPI | Alkaline phosphatase, intestinal |
| FABP1 | Fatty acid binding protein 1, liver; Plays a role in lipoprotein-mediated cholesterol uptake in hepatocytes (PubMed:25732850). Binds cholesterol (PubMed:25732850). Binds free fatty acids and their coenzyme A derivatives, bilirubin, and some other small molecules in the cytoplasm. May be involved in intracellular lipid transport (By similarity) |
| RHO | Rhodopsin; Photoreceptor required for image-forming vision at low light intensity. Required for photoreceptor cell viability after birth. Light-induced isomerization of 11-cis to all-trans retinal triggers a conformational change leading to G-protein activation and release of all-trans retinal |
| CSF2 | Colony stimulating factor 2 (granulocyte-macrophage); Cytokine that stimulates the growth and differentiation of hematopoietic precursor cells from various lineages, including granulocytes, macrophages, eosinophils and erythrocytes |
| FABP5 | Fatty acid binding protein 5 (psoriasis-associated); High specificity for fatty acids. Highest affinity for C18 chain length. Decreasing the chain length or introducing double bonds reduces the affinity. May be involved in keratinocyte differentiation |
| NOS3 | Nitric oxide synthase 3 (endothelial cell); Produces nitric oxide (NO) which is implicated in vascular smooth muscle relaxation through a cGMP-mediated signal transduction pathway. NO mediates vascular endothelial growth factor (VEGF)-induced angiogenesis in coronary vessels and promotes blood clotting through the activation of platelets |
| MMP3 | Matrix metallopeptidase 3 (stromelysin 1, progelatinase); Can degrade fibronectin, laminin, gelatins of type I, III, IV, and V; collagens III, IV, X, and IX, and cartilage proteoglycans. Activates procollagenase |
| TRPM2 | Transient receptor potential cation channel, subfamily M, member 2; Nonselective, voltage-independent cation channel mediating sodium and calcium ion influx in response to oxidative stress. Extracellular calcium passes through the channel and acts from the intracellular side as a positive regulator in channel activation. Activated by ADP-ribose, nicotinamide adenine dinucleotide (NAD(+)), reactive nitrogen species and arachidonic acid. Inactivated by intracellular ATP. Confers susceptibility to cell death following oxidative stress. Isoform 2 does not seem to be regulated by ADPR. Has [...] |
| BIRC5 | Baculoviral IAP repeat containing 5 |
| BCL2L1 | BCL2-like 1; Potent inhibitor of cell death. Inhibits activation of caspases. Appears to regulate cell death by blocking the voltage- dependent anion channel (VDAC) by binding to it and preventing the release of the caspase activator, CYC1, from the mitochondrial membrane. Also acts as a regulator of G2 checkpoint and progression to cytokinesis during mitosis |
| ACHE | Acetylcholinesterase; Terminates signal transduction at the neuromuscular junction by rapid hydrolysis of the acetylcholine released into the synaptic cleft. Role in neuronal apoptosis |
| ITGB2 | Integrin, beta 2 (complement component 3 receptor 3 and 4 subunit); Integrin alpha-L/beta-2 is a receptor for ICAM1, ICAM2, ICAM3 and ICAM4. Integrins alpha-M/beta-2 and alpha-X/beta-2 are receptors for the iC3b fragment of the third complement component and for fibrinogen. Integrin alpha-X/beta-2 recognizes the sequence G-P-R in fibrinogen alpha-chain. Integrin alpha-M/beta-2 recognizes P1 and P2 peptides of fibrinogen gamma chain. Integrin alpha-M/beta-2 is also a receptor for factor X. Integrin alpha- D/beta-2 is a receptor for ICAM3 and VCAM1. Triggers neutrophil transmigration dur [...] |
| DEFB4A | Defensin, beta 4A; Has antibacterial activity |
| INSR | Insulin receptor; Receptor tyrosine kinase which mediates the pleiotropic actions of insulin. Binding of insulin leads to phosphorylation of several intracellular substrates, including, insulin receptor substrates (IRS1, 2, 3, 4), SHC, GAB1, CBL and other signaling intermediates. Each of these phosphorylated proteins serve as docking proteins for other signaling proteins that contain Src- homology-2 domains (SH2 domain) that specifically recognize different phosphotyrosines residues, including the p85 regulatory subunit of PI3K and SHP2. Phosphorylation of IRSs proteins lead to the act [...] |
| COL3A1 | Collagen, type III, alpha 1; Collagen type III occurs in most soft connective tissues along with type I collagen. Involved in regulation of cortical development. Is the major ligand of GPR56 in the developing brain and binding to GPR56 inhibits neuronal migration and activates the RhoA pathway by coupling GPR56 to GNA13 and possibly GNA12 |
| FASN | Fatty acid synthase; Fatty acid synthetase catalyzes the formation of long- chain fatty acids from acetyl-CoA, malonyl-CoA and NADPH. This multifunctional protein has 7 catalytic activities and an acyl carrier protein |
| IL13 | Interleukin 13; Cytokine (PubMed:8096327, PubMed:8097324). Inhibits inflammatory cytokine production (PubMed:8096327). Synergizes with IL2 in regulating interferon-gamma synthesis (PubMed:8096327). May be critical in regulating inflammatory and immune responses (PubMed:8096327, PubMed:8097324). Positively regulates IL31RA expression in macrophages (By similarity) |
| PRKCB | Protein kinase C, beta; Calcium-activated, phospholipid- and diacylglycerol (DAG)-dependent serine/threonine-protein kinase involved in various cellular processes such as regulation of the B-cell receptor (BCR) signalosome, oxidative stress-induced apoptosis, androgen receptor-dependent transcription regulation, insulin signaling and endothelial cells proliferation. Plays a key role in B-cell activation by regulating BCR-induced NF-kappa-B activation. Mediates the activation of the canonical NF-kappa-B pathway (NFKB1) by direct phosphorylation of CARD11/CARMA1 at 'Ser-559', 'Ser-644' a [...] |
| ADRB2 | Adrenoceptor beta 2, surface; Beta-adrenergic receptors mediate the catecholamine- induced activation of adenylate cyclase through the action of G proteins. The beta-2-adrenergic receptor binds epinephrine with an approximately 30-fold greater affinity than it does norepinephrine |
| CXCL10 | Chemokine (C-X-C motif) ligand 10; Chemotactic for monocytes and T-lymphocytes. Binds to CXCR3 |
| GAA | Glucosidase, alpha; acid; Essential for the degradation of glygogen to glucose in lysosomes |
| PAM | Peptidylglycine alpha-amidating monooxygenase; Bifunctional enzyme that catalyzes 2 sequential steps in C-terminal alpha-amidation of peptides. The monooxygenase part produces an unstable peptidyl(2-hydroxyglycine) intermediate that is dismutated to glyoxylate and the corresponding desglycine peptide amide by the lyase part. C-terminal amidation of peptides such as neuropeptides is essential for full biological activity |
| DRD5 | Dopamine receptor D5; Dopamine receptor whose activity is mediated by G proteins which activate adenylyl cyclase |
| FOS | FBJ murine osteosarcoma viral oncogene homolog; Nuclear phosphoprotein which forms a tight but non- covalently linked complex with the JUN/AP-1 transcription factor. In the heterodimer, FOS and JUN/AP-1 basic regions each seems to interact with symmetrical DNA half sites. On TGF-beta activation, forms a multimeric SMAD3/SMAD4/JUN/FOS complex at the AP1/SMAD- binding site to regulate TGF-beta-mediated signaling. Has a critical function in regulating the development of cells destined to form and maintain the skeleton. It is thought to have an important role in signal transduction, cell p [...] |
| CHRM1 | Cholinergic receptor, muscarinic 1; The muscarinic acetylcholine receptor mediates various cellular responses, including inhibition of adenylate cyclase, breakdown of phosphoinositides and modulation of potassium channels through the action of G proteins. Primary transducing effect is Pi turnover |
| IL8 | Interleukin 8; IL-8 is a chemotactic factor that attracts neutrophils, basophils, and T-cells, but not monocytes. It is also involved in neutrophil activation. It is released from several cell types in response to an inflammatory stimulus. IL-8(6-77) has a 5-10-fold higher activity on neutrophil activation, IL-8(5-77) has increased activity on neutrophil activation and IL-8(7-77) has a higher affinity to receptors CXCR1 and CXCR2 as compared to IL-8(1-77), respectively |
| ADH1B | Alcohol dehydrogenase 1B (class I), beta polypeptide |
| ADRA1B | Adrenoceptor alpha 1B; This alpha-adrenergic receptor mediates its action by association with G proteins that activate a phosphatidylinositol- calcium second messenger system. Its effect is mediated by G(q) and G(11) proteins. Nuclear ADRA1A-ADRA1B heterooligomers regulate phenylephrine (PE)-stimulated ERK signaling in cardiac myocytes |
| CXCL11 | Chemokine (C-X-C motif) ligand 11; Chemotactic for interleukin-activated T-cells but not unstimulated T-cells, neutrophils or monocytes. Induces calcium release in activated T-cells. Binds to CXCR3. May play an important role in CNS diseases which involve T-cell recruitment. May play a role in skin immune responses |
| HAS2 | Hyaluronan synthase 2; Catalyzes the addition of GlcNAc or GlcUA monosaccharides to the nascent hyaluronan polymer. Therefore, it is essential to hyaluronan synthesis a major component of most extracellular matrices that has a structural role in tissues architectures and regulates cell adhesion, migration and differentiation. This is one of the isozymes catalyzing that reaction and it is particularly responsible for the synthesis of high molecular mass hyaluronan. Required for the transition of endocardial cushion cells into mesenchymal cells, a process crucial for heart development. M [...] |
| CYCS | Cytochrome c, somatic; Electron carrier protein. The oxidized form of the cytochrome c heme group can accept an electron from the heme group of the cytochrome c1 subunit of cytochrome reductase. Cytochrome c then transfers this electron to the cytochrome oxidase complex, the final protein carrier in the mitochondrial electron-transport chain |
| BTK | Bruton agammaglobulinemia tyrosine kinase; Non-receptor tyrosine kinase indispensable for B lymphocyte development, differentiation and signaling. Binding of antigen to the B-cell antigen receptor (BCR) triggers signaling that ultimately leads to B-cell activation. After BCR engagement and activation at the plasma membrane, phosphorylates PLCG2 at several sites, igniting the downstream signaling pathway through calcium mobilization, followed by activation of the protein kinase C (PKC) family members. PLCG2 phosphorylation is performed in close cooperation with the adapter protein B-cel [...] |
| PRSS1 | Protease, serine, 1 (trypsin 1); Has activity against the synthetic substrates Boc-Phe- Ser-Arg-Mec, Boc-Leu-Thr-Arg-Mec, Boc-Gln-Ala-Arg-Mec and Boc-Val- Pro-Arg-Mec. The single-chain form is more active than the two- chain form against all of these substrates |
| PLG | Plasminogen; Plasmin dissolves the fibrin of blood clots and acts as a proteolytic factor in a variety of other processes including embryonic development, tissue remodeling, tumor invasion, and inflammation. In ovulation, weakens the walls of the Graafian follicle. It activates the urokinase-type plasminogen activator, collagenases and several complement zymogens, such as C1 and C5. Cleavage of fibronectin and laminin leads to cell detachment and apoptosis. Also cleaves fibrin, thrombospondin and von Willebrand factor. Its role in tissue remodeling and tumor invasion may be modulated b [...] |
| BAD | BCL2-associated agonist of cell death; Promotes cell death. Successfully competes for the binding to Bcl-X(L), Bcl-2 and Bcl-W, thereby affecting the level of heterodimerization of these proteins with BAX. Can reverse the death repressor activity of Bcl-X(L), but not that of Bcl-2 (By similarity). Appears to act as a link between growth factor receptor signaling and the apoptotic pathways |
| LPL | Lipoprotein lipase; The primary function of this lipase is the hydrolysis of triglycerides of circulating chylomicrons and very low density lipoproteins (VLDL). Binding to heparin sulfate proteogylcans at the cell surface is vital to the function. The apolipoprotein, APOC2, acts as a coactivator of LPL activity in the presence of lipids on the luminal surface of vascular endothelium (By similarity) |
| FOSL1 | FOS-like antigen 1 |
| GLS2 | Glutaminase 2 (liver, mitochondrial); Plays an important role in the regulation of glutamine catabolism. Promotes mitochondrial respiration and increases ATP generation in cells by catalyzing the synthesis of glutamate and alpha-ketoglutarate. Increases cellular anti-oxidant function via NADH and glutathione production. May play a role in preventing tumor proliferation |
| PPARD | Peroxisome proliferator-activated receptor delta; Ligand-activated transcription factor. Receptor that binds peroxisome proliferators such as hypolipidemic drugs and fatty acids. Has a preference for poly-unsaturated fatty acids, such as gamma-linoleic acid and eicosapentanoic acid. Once activated by a ligand, the receptor binds to promoter elements of target genes. Regulates the peroxisomal beta-oxidation pathway of fatty acids. Functions as transcription activator for the acyl-CoA oxidase gene. Decreases expression of NPC1L1 once activated by a ligand |
| CASP3 | Caspase 3, apoptosis-related cysteine peptidase; Involved in the activation cascade of caspases responsible for apoptosis execution. At the onset of apoptosis it proteolytically cleaves poly(ADP-ribose) polymerase (PARP) at a '216-Asp-\|-Gly-217' bond. Cleaves and activates sterol regulatory element binding proteins (SREBPs) between the basic helix-loop- helix leucine zipper domain and the membrane attachment domain. Cleaves and activates caspase-6, -7 and -9. Involved in the cleavage of huntingtin. Triggers cell adhesion in sympathetic neurons through RET cleavage |
| GSTM1 | Glutathione S-transferase mu 1; Conjugation of reduced glutathione to a wide number of exogenous and endogenous hydrophobic electrophiles |
| UCP2 | Uncoupling protein 2 (mitochondrial, proton carrier); UCP are mitochondrial transporter proteins that create proton leaks across the inner mitochondrial membrane, thus uncoupling oxidative phosphorylation from ATP synthesis. As a result, energy is dissipated in the form of heat |
| PLA2G1B | Phospholipase A2, group IB (pancreas); PA2 catalyzes the calcium-dependent hydrolysis of the 2- acyl groups in 3-sn-phosphoglycerides, this releases glycerophospholipids and arachidonic acid that serve as the precursors of signal molecules |
| CFLAR | CASP8 and FADD-like apoptosis regulator; Apoptosis regulator protein which may function as a crucial link between cell survival and cell death pathways in mammalian cells. Acts as an inhibitor of TNFRSF6 mediated apoptosis. A proteolytic fragment (p43) is likely retained in the death-inducing signaling complex (DISC) thereby blocking further recruitment and processing of caspase-8 at the complex. Full length and shorter isoforms have been shown either to induce apoptosis or to reduce TNFRSF-triggered apoptosis. Lacks enzymatic (caspase) activity |
| NQO1 | NAD(P)H dehydrogenase, quinone 1; The enzyme apparently serves as a quinone reductase in connection with conjugation reactions of hydroquinons involved in detoxification pathways as well as in biosynthetic processes such as the vitamin K-dependent gamma-carboxylation of glutamate residues in prothrombin synthesis |
| CHRM2 | Cholinergic receptor, muscarinic 2; The muscarinic acetylcholine receptor mediates various cellular responses, including inhibition of adenylate cyclase, breakdown of phosphoinositides and modulation of potassium channels through the action of G proteins. Primary transducing effect is adenylate cyclase inhibition. Signaling promotes phospholipase C activity, leading to the release of inositol trisphosphate (IP3); this then triggers calcium ion release into the cytosol |
| NPEPPS | Aminopeptidase puromycin sensitive; Aminopeptidase with broad substrate specificity for several peptides. Involved in proteolytic events essential for cell growth and viability. May act as regulator of neuropeptide activity. Plays a role in the antigen-processing pathway for MHC class I molecules. Involved in the N-terminal trimming of cytotoxic T-cell epitope precursors. Digests the poly-Q peptides found in many cellular proteins. Digests tau from normal brain more efficiently than tau from Alzheimer disease brain |
| SLC2A4 | Solute carrier family 2 (facilitated glucose transporter), member 4; Insulin-regulated facilitative glucose transporter |
| NCOA1 | Nuclear receptor coactivator 1; Nuclear receptor coactivator that directly binds nuclear receptors and stimulates the transcriptional activities in a hormone-dependent fashion. Involved in the coactivation of different nuclear receptors, such as for steroids (PGR, GR and ER), retinoids (RXRs), thyroid hormone (TRs) and prostanoids (PPARs). Also involved in coactivation mediated by STAT3, STAT5A, STAT5B and STAT6 transcription factors. Displays histone acetyltransferase activity toward H3 and H4; the relevance of such activity remains however unclear. Plays a central role in creating mu [...] |
| ITM2C | Integral membrane protein 2C; Negative regulator of beta amyloid peptide production. May inhibit the processing of APP by blocking its access to alpha- and beta-secretase. Binding to the beta-secretase-cleaved APP C- terminal fragment is negligible, suggesting that ITM2C is a poor gamma-secretase cleavage inhibitor. May play a role in TNF-induced cell death and neuronal differentiation (By similarity) |
| MMP1 | Matrix metallopeptidase 1 (interstitial collagenase); Cleaves collagens of types I, II, and III at one site in the helical domain. Also cleaves collagens of types VII and X. In case of HIV infection, interacts and cleaves the secreted viral Tat protein, leading to a decrease in neuronal Tat's mediated neurotoxicity |
| ACPP | Acid phosphatase, prostate; A non-specific tyrosine phosphatase that dephosphorylates a diverse number of substrates under acidic conditions (pH 4-6) including alkyl, aryl, and acyl orthophosphate monoesters and phosphorylated proteins. Has lipid phosphatase activity and inactivates lysophosphatidic acid in seminal plasma |
| SLC2A2 | Solute carrier family 2 (facilitated glucose transporter), member 2; Facilitative glucose transporter. This isoform likely mediates the bidirectional transfer of glucose across the plasma membrane of hepatocytes and is responsible for uptake of glucose by the beta cells; may comprise part of the glucose-sensing mechanism of the beta cell. May also participate with the Na(+)/glucose cotransporter in the transcellular transport of glucose in the small intestine and kidney |
| UCP3 | Uncoupling protein 3 (mitochondrial, proton carrier); UCP are mitochondrial transporter proteins that create proton leaks across the inner mitochondrial membrane, thus uncoupling oxidative phosphorylation. As a result, energy is dissipated in the form of heat. May play a role in the modulation of tissue respiratory control. Participates in thermogenesis and energy balance |
| HSPA5 | Heat shock 70kDa protein 5 (glucose-regulated protein, 78kDa); Probably plays a role in facilitating the assembly of multimeric protein complexes inside the endoplasmic reticulum. Involved in the correct folding of proteins and degradation of misfolded proteins via its interaction with DNAJC10, probably to facilitate the release of DNAJC10 from its substrate |
| CYP2B6 | Cytochrome P450, family 2, subfamily B, polypeptide 6; Cytochromes P450 are a group of heme-thiolate monooxygenases. In liver microsomes, this enzyme is involved in an NADPH-dependent electron transport pathway. It oxidizes a variety of structurally unrelated compounds, including steroids, fatty acids, and xenobiotics. Acts as a 1,4-cineole 2-exo-monooxygenase |
| GSK3B | Glycogen synthase kinase 3 beta; Constitutively active protein kinase that acts as a negative regulator in the hormonal control of glucose homeostasis, Wnt signaling and regulation of transcription factors and microtubules, by phosphorylating and inactivating glycogen synthase (GYS1 or GYS2), EIF2B, CTNNB1/beta-catenin, APC, AXIN1, DPYSL2/CRMP2, JUN, NFATC1/NFATC, MAPT/TAU and MACF1. Requires primed phosphorylation of the majority of its substrates. In skeletal muscle, contributes to insulin regulation of glycogen synthesis by phosphorylating and inhibiting GYS1 activity and hence glyc [...] |
| PGR | Progesterone receptor; The steroid hormones and their receptors are involved in the regulation of eukaryotic gene expression and affect cellular proliferation and differentiation in target tissues. Progesterone receptor isoform B (PRB) is involved activation of c-SRC/MAPK signaling on hormone stimulation |
| SERTAD3 | SERTA domain containing 3; Strong transcriptional coactivator |
| NOS2 | Nitric oxide synthase 2, inducible; Produces nitric oxide (NO) which is a messenger molecule with diverse functions throughout the body (PubMed:7531687, PubMed:7544004). In macrophages, NO mediates tumoricidal and bactericidal actions. Also has nitrosylase activity and mediates cysteine S-nitrosylation of cytoplasmic target proteins such PTGS2/COX2 (By similarity). As component of the iNOS-S100A8/9 transnitrosylase complex involved in the selective inflammatory stimulus-dependent S-nitrosylation of GAPDH on 'Cys-247' implicated in regulation of the GAIT complex activity and probably mu [...] |
| OR1D2 | Olfactory receptor, family 1, subfamily D, member 2; Odorant receptor which may be involved in sperm chemotaxis. Bourgeonal is a strong chemoattractant for sperm in vitro and is shown to be a strong agonist for OR1D2 in vitro. May also function in olfactory reception |
| DRD1 | Dopamine receptor D1; Dopamine receptor whose activity is mediated by G proteins which activate adenylyl cyclase |
| NFATC1 | Nuclear factor of activated T-cells, cytoplasmic, calcineurin-dependent 1; Plays a role in the inducible expression of cytokine genes in T-cells, especially in the induction of the IL-2 or IL-4 gene transcription. Also controls gene expression in embryonic cardiac cells. Could regulate not only the activation and proliferation but also the differentiation and programmed death of T-lymphocytes as well as lymphoid and non-lymphoid cells (PubMed:10358178). Required for osteoclastogenesis and regulates many genes important for osteoclast differentiation and function (By similarity) |
| SCN5A | Sodium channel, voltage-gated, type V, alpha subunit; This protein mediates the voltage-dependent sodium ion permeability of excitable membranes. Assuming opened or closed conformations in response to the voltage difference across the membrane, the protein forms a sodium-selective channel through which Na(+) ions may pass in accordance with their electrochemical gradient. It is a tetrodotoxin-resistant Na(+) channel isoform. This channel is responsible for the initial upstroke of the action potential. Channel inactivation is regulated by intracellular calcium levels |
| SP1 | Sp1 transcription factor; Transcription factor that can activate or repress transcription in response to physiological and pathological stimuli. Binds with high affinity to GC-rich motifs and regulates the expression of a large number of genes involved in a variety of processes such as cell growth, apoptosis, differentiation and immune responses. Highly regulated by post-translational modifications (phosphorylations, sumoylation, proteolytic cleavage, glycosylation and acetylation). Binds also the PDGFR- alpha G-box promoter. May have a role in modulating the cellular response to DNA d [...] |
| BCL2 | B-cell CLL/lymphoma 2; Suppresses apoptosis in a variety of cell systems including factor-dependent lymphohematopoietic and neural cells. Regulates cell death by controlling the mitochondrial membrane permeability. Appears to function in a feedback loop system with caspases. Inhibits caspase activity either by preventing the release of cytochrome c from the mitochondria and/or by binding to the apoptosis-activating factor (APAF-1). May attenuate inflammation by impairing NLRP1-inflammasome activation, hence CASP1 activation and IL1B release (PubMed:17418785) |
| CASP9 | Caspase 9, apoptosis-related cysteine peptidase; Involved in the activation cascade of caspases responsible for apoptosis execution. Binding of caspase-9 to Apaf- 1 leads to activation of the protease which then cleaves and activates caspase-3. Promotes DNA damage-induced apoptosis in a ABL1/c-Abl-dependent manner. Proteolytically cleaves poly(ADP- ribose) polymerase (PARP) |
| PLB1 | Phospholipase B1; Membrane-associated phospholipase. Exhibits a calcium- independent broad substrate specificity including phospholipase A2/lysophospholipase activity. Preferential hydrolysis at the sn-2 position of diacylphospholipids and diacyglycerol, whereas it shows no positional specificity toward triacylglycerol. Exhibits also esterase activity toward p-nitrophenyl. May act on the brush border membrane to facilitate the absorption of digested lipids (By similarity) |
| PRKCD | Protein kinase C, delta; Calcium-independent, phospholipid- and diacylglycerol (DAG)-dependent serine/threonine-protein kinase that plays contrasting roles in cell death and cell survival by functioning as a pro-apoptotic protein during DNA damage-induced apoptosis, but acting as an anti-apoptotic protein during cytokine receptor- initiated cell death, is involved in tumor suppression as well as survival of several cancers, is required for oxygen radical production by NADPH oxidase and acts as positive or negative regulator in platelet functional responses. Negatively regulates B cell [...] |
| SELE | Selectin E; Cell-surface glycoprotein having a role in immunoadhesion. Mediates in the adhesion of blood neutrophils in cytokine-activated endothelium through interaction with PSGL1/SELPLG. May have a role in capillary morphogenesis |
| CD86 | CD86 molecule; Receptor involved in the costimulatory signal essential for T-lymphocyte proliferation and interleukin-2 production, by binding CD28 or CTLA-4. May play a critical role in the early events of T-cell activation and costimulation of naive T-cells, such as deciding between immunity and anergy that is made by T- cells within 24 hours after activation. Isoform 2 interferes with the formation of CD86 clusters, and thus acts as a negative regulator of T-cell activation |
| SMAD3 | SMAD family member 3; Receptor-regulated SMAD (R-SMAD) that is an intracellular signal transducer and transcriptional modulator activated by TGF-beta (transforming growth factor) and activin type 1 receptor kinases. Binds the TRE element in the promoter region of many genes that are regulated by TGF-beta and, on formation of the SMAD3/SMAD4 complex, activates transcription. Also can form a SMAD3/SMAD4/JUN/FOS complex at the AP-1/SMAD site to regulate TGF-beta-mediated transcription. Has an inhibitory effect on wound healing probably by modulating both growth and migration of primary ke [...] |
| F3 | Coagulation factor III (thromboplastin, tissue factor); Initiates blood coagulation by forming a complex with circulating factor VII or VIIa. The [TF:VIIa] complex activates factors IX or X by specific limited protolysis. TF plays a role in normal hemostasis by initiating the cell-surface assembly and propagation of the coagulation protease cascade |
| CCK | Cholecystokinin; This peptide hormone induces gall bladder contraction and the release of pancreatic enzymes in the gut. Its function in the brain is not clear. Binding to CCK-A receptors stimulates amylase release from the pancreas, binding to CCK-B receptors stimulates gastric acid secretion |
| NR1I2 | Nuclear receptor subfamily 1, group I, member 2; Nuclear receptor that binds and is activated by variety of endogenous and xenobiotic compounds. Transcription factor that activates the transcription of multiple genes involved in the metabolism and secretion of potentially harmful xenobiotics, drugs and endogenous compounds. Activated by the antibiotic rifampicin and various plant metabolites, such as hyperforin, guggulipid, colupulone, and isoflavones. Response to specific ligands is species-specific. Activated by naturally occurring steroids, such as pregnenolone and progesterone. Bin [...] |
| PKIA | Protein kinase (cAMP-dependent, catalytic) inhibitor alpha; Extremely potent competitive inhibitor of cAMP-dependent protein kinase activity, this protein interacts with the catalytic subunit of the enzyme after the cAMP-induced dissociation of its regulatory chains |
| ENOX2 | ecto-NOX disulfide-thiol exchanger 2; May be involved in cell growth. Probably acts as a terminal oxidase of plasma electron transport from cytosolic NAD(P)H via hydroquinones to acceptors at the cell surface. Hydroquinone oxidase activity alternates with a protein disulfide- thiol interchange/oxidoreductase activity which may control physical membrane displacements associated with vesicle budding or cell enlargement. The activities oscillate with a period length of 22 minutes and play a role in control of the ultradian cellular biological clock |
| ISYNA1 | Inositol-3-phosphate synthase 1; Key enzyme in myo-inositol biosynthesis pathway that catalyzes the conversion of glucose 6-phosphate to 1-myo-inositol 1-phosphate in a NAD-dependent manner. Rate-limiting enzyme in the synthesis of all inositol-containing compounds |
| CYP3A4 | Cytochrome P450, family 3, subfamily A, polypeptide 4; Cytochromes P450 are a group of heme-thiolate monooxygenases. In liver microsomes, this enzyme is involved in an NADPH-dependent electron transport pathway. It performs a variety of oxidation reactions (e.g. caffeine 8-oxidation, omeprazole sulphoxidation, midazolam 1'-hydroxylation and midazolam 4- hydroxylation) of structurally unrelated compounds, including steroids, fatty acids, and xenobiotics. Acts as a 1,8-cineole 2- exo-monooxygenase. The enzyme also hydroxylates etoposide (PubMed:11159812). Catalyzes 4-beta-hydroxylation o [...] |
| HIF1A | Hypoxia inducible factor 1, alpha subunit (basic helix-loop-helix transcription factor); Functions as a master transcriptional regulator of the adaptive response to hypoxia. Under hypoxic conditions, activates the transcription of over 40 genes, including erythropoietin, glucose transporters, glycolytic enzymes, vascular endothelial growth factor, HILPDA, and other genes whose protein products increase oxygen delivery or facilitate metabolic adaptation to hypoxia. Plays an essential role in embryonic vascularization, tumor angiogenesis and pathophysiology of ischemic disease. Binds to [...] |
| CAV1 | Caveolin 1, caveolae protein, 22kDa; May act as a scaffolding protein within caveolar membranes. Interacts directly with G-protein alpha subunits and can functionally regulate their activity (By similarity). Involved in the costimulatory signal essential for T-cell receptor (TCR)- mediated T-cell activation. Its binding to DPP4 induces T-cell proliferation and NF-kappa-B activation in a T-cell receptor/CD3- dependent manner. Recruits CTNNB1 to caveolar membranes and may regulate CTNNB1-mediated signaling through the Wnt pathway. Negatively regulates TGFB1-mediated activation of SMAD2/3 [...] |
| CRYZ | Crystallin, zeta (quinone reductase); Does not have alcohol dehydrogenase activity. Binds NADP and acts through a one-electron transfer process. Orthoquinones, such as 1,2-naphthoquinone or 9,10-phenanthrenequinone, are the best substrates (in vitro). May act in the detoxification of xenobiotics. Interacts with (AU)-rich elements (ARE) in the 3'-UTR of target mRNA species. Enhances the stability of mRNA coding for BCL2. NADPH binding interferes with mRNA binding |
| MAOA | Monoamine oxidase A; Catalyzes the oxidative deamination of biogenic and xenobiotic amines and has important functions in the metabolism of neuroactive and vasoactive amines in the central nervous system and peripheral tissues. MAOA preferentially oxidizes biogenic amines such as 5-hydroxytryptamine (5-HT), norepinephrine and epinephrine |
| DCAF5 | DDB1 and CUL4 associated factor 5; May function as a substrate receptor for CUL4-DDB1 E3 ubiquitin-protein ligase complex |
| NR3C2 | Nuclear receptor subfamily 3, group C, member 2; Receptor for both mineralocorticoids (MC) such as aldosterone and glucocorticoids (GC) such as corticosterone or cortisol. Binds to mineralocorticoid response elements (MRE) and transactivates target genes. The effect of MC is to increase ion and water transport and thus raise extracellular fluid volume and blood pressure and lower potassium levels |
| CYP1A2 | Cytochrome P450, family 1, subfamily A, polypeptide 2; Cytochromes P450 are a group of heme-thiolate monooxygenases. In liver microsomes, this enzyme is involved in an NADPH-dependent electron transport pathway. It oxidizes a variety of structurally unrelated compounds, including steroids, fatty acids, and xenobiotics. Most active in catalyzing 2-hydroxylation. Caffeine is metabolized primarily by cytochrome CYP1A2 in the liver through an initial N3-demethylation. Also acts in the metabolism of aflatoxin B1 and acetaminophen. Participates in the bioactivation of carcinogenic aromatic a [...] |
| CTSB | Cathepsin B; Thiol protease which is believed to participate in intracellular degradation and turnover of proteins. Has also been implicated in tumor invasion and metastasis |
| SLPI | Secretory leukocyte peptidase inhibitor; Acid-stable proteinase inhibitor with strong affinities for trypsin, chymotrypsin, elastase, and cathepsin G (PubMed:3533531, PubMed:3462719, PubMed:2039600, PubMed:2110563, PubMed:10702419, PubMed:24121345). Modulates the inflammatory and immune responses after bacterial infection, and after infection by the intracellular parasite L.major. Down-regulates responses to bacterial lipopolysaccharide (LPS) (By similarity). Plays a role in regulating the activation of NF-kappa-B and inflammatory responses (PubMed:10702419, PubMed:24352879). Has antim [...] |
| CLDN4 | Claudin 4; Plays a major role in tight junction-specific obliteration of the intercellular space |
| ESR2 | Estrogen receptor 2 (ER beta); Nuclear hormone receptor. Binds estrogens with an affinity similar to that of ESR1, and activates expression of reporter genes containing estrogen response elements (ERE) in an estrogen-dependent manner (PubMed:20074560). Isoform beta-cx lacks ligand binding ability and has no or only very low ere binding activity resulting in the loss of ligand-dependent transactivation ability. DNA-binding by ESR1 and ESR2 is rapidly lost at 37 degrees Celsius in the absence of ligand while in the presence of 17 beta-estradiol and 4-hydroxy-tamoxifen loss in DNA-binding [...] |
| ACACA | acetyl-CoA carboxylase alpha; Catalyzes the rate-limiting reaction in the biogenesis of long-chain fatty acids. Carries out three functions: biotin carboxyl carrier protein, biotin carboxylase and carboxyltransferase |
| APOD | Apolipoprotein D; APOD occurs in the macromolecular complex with lecithin- cholesterol acyltransferase. It is probably involved in the transport and binding of bilin. Appears to be able to transport a variety of ligands in a number of different contexts |
| E2F1 | E2F transcription factor 1; Transcription activator that binds DNA cooperatively with DP proteins through the E2 recognition site, 5'-TTTC[CG]CGC- 3' found in the promoter region of a number of genes whose products are involved in cell cycle regulation or in DNA replication. The DRTF1/E2F complex functions in the control of cell-cycle progression from G1 to S phase. E2F1 binds preferentially RB1 in a cell-cycle dependent manner. It can mediate both cell proliferation and TP53/p53-dependent apoptosis. Blocks adipocyte differentiation by binding to specific promoters repressing CEBPA bin [...] |
| C5AR1 | Complement component 5a receptor 1; Receptor for the chemotactic and inflammatory peptide anaphylatoxin C5a (PubMed:1847994, PubMed:8182049, PubMed:7622471, PubMed:9553099, PubMed:10636859, PubMed:15153520). The ligand interacts with at least two sites on the receptor: a high-affinity site on the extracellular N-terminus, and a second site in the transmembrane region which activates downstream signaling events (PubMed:8182049, PubMed:7622471, PubMed:9553099). Receptor activation stimulates chemotaxis, granule enzyme release, intracellular calcium release and superoxide anion production [...] |
| TDRD7 | Tudor domain containing 7; Component of specific cytoplasmic RNA granules involved in post-transcriptional regulation of specific genes: probably acts by binding to specific mRNAs and regulating their translation. Required for lens transparency during lens development, by regulating translation of genes such as CRYBB3 and HSPB1 in the developing lens. Also required during spermatogenesis |
| HTR3A | 5-hydroxytryptamine (serotonin) receptor 3A, ionotropic |
| XIAP | X-linked inhibitor of apoptosis; Multi-functional protein which regulates not only caspases and apoptosis, but also modulates inflammatory signaling and immunity, copper homeostasis, mitogenic kinase signaling, cell proliferation, as well as cell invasion and metastasis. Acts as a direct caspase inhibitor. Directly bind to the active site pocket of CASP3 and CASP7 and obstructs substrate entry. Inactivates CASP9 by keeping it in a monomeric, inactive state. Acts as an E3 ubiquitin-protein ligase regulating NF-kappa-B signaling and the target proteins for its E3 ubiquitin-protein ligase [...] |
| RAC1 | Ras-related C3 botulinum toxin substrate 1 (rho family, small GTP binding protein Rac1) |
| PTGER3 | Prostaglandin E receptor 3 (subtype EP3); Receptor for prostaglandin E2 (PGE2); the EP3 receptor may be involved in inhibition of gastric acid secretion, modulation of neurotransmitter release in central and peripheral neurons, inhibition of sodium and water reabsorption in kidney tubulus and contraction in uterine smooth muscle. The activity of this receptor can couple to both the inhibition of adenylate cyclase mediated by G-I proteins, and to an elevation of intracellular calcium. The various isoforms have identical ligand binding properties but can interact with different second me [...] |
| RASSF1 | Ras association (RalGDS/AF-6) domain family member 1; Potential tumor suppressor. Required for death receptor- dependent apoptosis. Mediates activation of STK3/MST2 and STK4/MST1 during Fas-induced apoptosis by preventing their dephosphorylation. When associated with MOAP1, promotes BAX conformational change and translocation to mitochondrial membranes in response to TNF and TNFSF10 stimulation. Isoform A interacts with CDC20, an activator of the anaphase-promoting complex, APC, resulting in the inhibition of APC activity and mitotic progression. Inhibits proliferation by negatively re [...] |
| ACTB | Actin, beta; Actins are highly conserved proteins that are involved in various types of cell motility and are ubiquitously expressed in all eukaryotic cells |
| NF1 | Neurofibromin 1; Stimulates the GTPase activity of Ras. NF1 shows greater affinity for Ras GAP, but lower specific activity. May be a regulator of Ras activity |
| CASP8 | Caspase 8, apoptosis-related cysteine peptidase; Most upstream protease of the activation cascade of caspases responsible for the TNFRSF6/FAS mediated and TNFRSF1A induced cell death. Binding to the adapter molecule FADD recruits it to either receptor. The resulting aggregate called death- inducing signaling complex (DISC) performs CASP8 proteolytic activation. The active dimeric enzyme is then liberated from the DISC and free to activate downstream apoptotic proteases. Proteolytic fragments of the N-terminal propeptide (termed CAP3, CAP5 and CAP6) are likely retained in the DISC. Clea [...] |
| MAPK10 | Mitogen-activated protein kinase 10; Serine/threonine-protein kinase involved in various processes such as neuronal proliferation, differentiation, migration and programmed cell death. Extracellular stimuli such as proinflammatory cytokines or physical stress stimulate the stress- activated protein kinase/c-Jun N-terminal kinase (SAP/JNK) signaling pathway. In this cascade, two dual specificity kinases MAP2K4/MKK4 and MAP2K7/MKK7 phosphorylate and activate MAPK10/JNK3. In turn, MAPK10/JNK3 phosphorylates a number of transcription factors, primarily components of AP-1 such as JUN and AT [...] |
| PYY | Peptide YY; This gut peptide inhibits exocrine pancreatic secretion, has a vasoconstrictory action and inhibitis jejunal and colonic mobility |
| MAPK8 | Mitogen-activated protein kinase 8; Serine/threonine-protein kinase involved in various processes such as cell proliferation, differentiation, migration, transformation and programmed cell death. Extracellular stimuli such as proinflammatory cytokines or physical stress stimulate the stress-activated protein kinase/c-Jun N-terminal kinase (SAP/JNK) signaling pathway. In this cascade, two dual specificity kinases MAP2K4/MKK4 and MAP2K7/MKK7 phosphorylate and activate MAPK8/JNK1. In turn, MAPK8/JNK1 phosphorylates a number of transcription factors, primarily components of AP-1 such as JU [...] |
| MAP2 | Microtubule-associated protein 2; Cotranslationally removes the N-terminal methionine from nascent proteins. The N-terminal methionine is often cleaved when the second residue in the primary sequence is small and uncharged (Met-Ala-, Cys, Gly, Pro, Ser, Thr, or Val). The catalytic activity of human METAP2 toward Met-Val peptides is consistently two orders of magnitude higher than that of METAP1, suggesting that it is responsible for processing proteins containing N- terminal Met-Val and Met-Thr sequences in vivo |
| MME | Membrane metallo-endopeptidase; Thermolysin-like specificity, but is almost confined on acting on polypeptides of up to 30 amino acids (PubMed:15283675, PubMed:8168535). Biologically important in the destruction of opioid peptides such as Met- and Leu-enkephalins by cleavage of a Gly-Phe bond (PubMed:17101991). Able to cleave angiotensin-1, angiotensin-2 and angiotensin 1-9 (PubMed:15283675). Involved in the degradation of atrial natriuretic factor (ANF) (PubMed:2531377, PubMed:2972276). Displays UV-inducible elastase activity toward skin preelastic and elastic fibers (PubMed:20876573) |
| DPP4 | Dipeptidyl-peptidase 4; Cell surface glycoprotein receptor involved in the costimulatory signal essential for T-cell receptor (TCR)-mediated T-cell activation. Acts as a positive regulator of T-cell coactivation, by binding at least ADA, CAV1, IGF2R, and PTPRC. Its binding to CAV1 and CARD11 induces T-cell proliferation and NF- kappa-B activation in a T-cell receptor/CD3-dependent manner. Its interaction with ADA also regulates lymphocyte-epithelial cell adhesion. In association with FAP is involved in the pericellular proteolysis of the extracellular matrix (ECM), the migration and in [...] |
| BAK1 | BCL2-antagonist/killer 1; In the presence of an appropriate stimulus, accelerates programmed cell death by binding to, and antagonizing the anti- apoptotic action of BCL2 or its adenovirus homolog E1B 19k protein. Low micromolar levels of zinc ions inhibit the promotion of apoptosis |
| PRSS3 | Protease, serine, 3; Digestive protease specialized for the degradation of trypsin inhibitors. In the ileum, may be involved in defensin processing, including DEFA5 |
| STAT1 | Signal transducer and activator of transcription 1, 91kDa; Signal transducer and transcription activator that mediates cellular responses to interferons (IFNs), cytokine KITLG/SCF and other cytokines and other growth factors. Following type I IFN (IFN-alpha and IFN-beta) binding to cell surface receptors, signaling via protein kinases leads to activation of Jak kinases (TYK2 and JAK1) and to tyrosine phosphorylation of STAT1 and STAT2. The phosphorylated STATs dimerize and associate with ISGF3G/IRF-9 to form a complex termed ISGF3 transcription factor, that enters the nucleus. ISGF3 bi [...] |
| TOP1 | Topoisomerase (DNA) I; Releases the supercoiling and torsional tension of DNA introduced during the DNA replication and transcription by transiently cleaving and rejoining one strand of the DNA duplex. Introduces a single-strand break via transesterification at a target site in duplex DNA. The scissile phosphodiester is attacked by the catalytic tyrosine of the enzyme, resulting in the formation of a DNA-(3'-phosphotyrosyl)-enzyme intermediate and the expulsion of a 5'-OH DNA strand. The free DNA strand then undergoes passage around the unbroken strand thus removing DNA supercoils. Fin [...] |
| PTGS1 | Prostaglandin-endoperoxide synthase 1 (prostaglandin G/H synthase and cyclooxygenase); Converts arachidonate to prostaglandin H2 (PGH2), a committed step in prostanoid synthesis. Involved in the constitutive production of prostanoids in particular in the stomach and platelets. In gastric epithelial cells, it is a key step in the generation of prostaglandins, such as prostaglandin E2 (PGE2), which plays an important role in cytoprotection. In platelets, it is involved in the generation of thromboxane A2 (TXA2), which promotes platelet activation and aggregation, vasoconstriction and pro [...] |
| DIO1 | Deiodinase, iodothyronine, type I; Responsible for the deiodination of T4 (3,5,3',5'- tetraiodothyronine) into T3 (3,5,3'-triiodothyronine) and of T3 into T2 (3,3'-diiodothyronine). Plays a role in providing a source of plasma T3 by deiodination of T4 in peripheral tissues such as liver and kidney |
| CACNA1S | Calcium channel, voltage-dependent, L type, alpha 1S subunit; Voltage-sensitive calcium channels (VSCC) mediate the entry of calcium ions into excitable cells and are also involved in a variety of calcium-dependent processes, including muscle contraction, hormone or neurotransmitter release, gene expression, cell motility, cell division and cell death. The isoform alpha-1S gives rise to L-type calcium currents. Long-lasting (L-type) calcium channels belong to the 'high-voltage activated' (HVA) group. They are blocked by dihydropyridines (DHP), phenylalkylamines, benzothiazepines, and b [...] |
| E2F2 | E2F transcription factor 2; Transcription activator that binds DNA cooperatively with DP proteins through the E2 recognition site, 5'-TTTC[CG]CGC- 3' found in the promoter region of a number of genes whose products are involved in cell cycle regulation or in DNA replication. The DRTF1/E2F complex functions in the control of cell-cycle progression from g1 to s phase. E2F2 binds specifically to RB1 in a cell-cycle dependent manner |
| EGLN1 | Egl nine homolog 1 (C. elegans); Cellular oxygen sensor that catalyzes, under normoxic conditions, the post-translational formation of 4-hydroxyproline in hypoxia-inducible factor (HIF) alpha proteins. Hydroxylates a specific proline found in each of the oxygen-dependent degradation (ODD) domains (N-terminal, NODD, and C-terminal, CODD) of HIF1A. Also hydroxylates HIF2A. Has a preference for the CODD site for both HIF1A and HIF1B. Hydroxylated HIFs are then targeted for proteasomal degradation via the von Hippel-Lindau ubiquitination complex. Under hypoxic conditions, the hydroxylation [...] |
| PARP1 | Poly (ADP-ribose) polymerase 1; Involved in the base excision repair (BER) pathway, by catalyzing the poly(ADP-ribosyl)ation of a limited number of acceptor proteins involved in chromatin architecture and in DNA metabolism. This modification follows DNA damages and appears as an obligatory step in a detection/signaling pathway leading to the reparation of DNA strand breaks. Mediates the poly(ADP- ribosyl)ation of APLF and CHFR. Positively regulates the transcription of MTUS1 and negatively regulates the transcription of MTUS2/TIP150. With EEF1A1 and TXK, forms a complex that acts as a [...] |
| PTGS2 | Prostaglandin-endoperoxide synthase 2 (prostaglandin G/H synthase and cyclooxygenase); Converts arachidonate to prostaglandin H2 (PGH2), a committed step in prostanoid synthesis. Constitutively expressed in some tissues in physiological conditions, such as the endothelium, kidney and brain, and in pathological conditions, such as in cancer. PTGS2 is responsible for production of inflammatory prostaglandins. Up-regulation of PTGS2 is also associated with increased cell adhesion, phenotypic changes, resistance to apoptosis and tumor angiogenesis. In cancer cells, PTGS2 is a key step in t [...] |
| SOAT1 | Sterol O-acyltransferase 1; Catalyzes the formation of fatty acid-cholesterol esters, which are less soluble in membranes than cholesterol. Plays a role in lipoprotein assembly and dietary cholesterol absorption. In addition to its acyltransferase activity, it may act as a ligase |
| NR1I3 | Nuclear receptor subfamily 1, group I, member 3; Binds and transactivates the retinoic acid response elements that control expression of the retinoic acid receptor beta 2 and alcohol dehydrogenase 3 genes. Transactivates both the phenobarbital responsive element module of the human CYP2B6 gene and the CYP3A4 xenobiotic response element |
| MUC1 | Mucin 1, cell surface associated; The alpha subunit has cell adhesive properties. Can act both as an adhesion and an anti-adhesion protein. May provide a protective layer on epithelial cells against bacterial and enzyme attack |
| IVL | Involucrin; Part of the insoluble cornified cell envelope (CE) of stratified squamous epithelia |
| ADRB1 | Adrenoceptor beta 1; Beta-adrenergic receptors mediate the catecholamine- induced activation of adenylate cyclase through the action of G proteins. This receptor binds epinephrine and norepinephrine with approximately equal affinity. Mediates Ras activation through G(s)-alpha- and cAMP-mediated signaling |
| IKBKG | Inhibitor of kappa light polypeptide gene enhancer in B-cells, kinase gamma; Regulatory subunit of the IKK core complex which phosphorylates inhibitors of NF-kappa-B thus leading to the dissociation of the inhibitor/NF-kappa-B complex and ultimately the degradation of the inhibitor. Its binding to scaffolding polyubiquitin seems to play a role in IKK activation by multiple signaling receptor pathways. However, the specific type of polyubiquitin recognized upon cell stimulation (either 'Lys-63'- linked or linear polyubiquitin) and its functional importance is reported conflictingly. Als [...] |
| SCD | stearoyl-CoA desaturase (delta-9-desaturase); Stearyl-CoA desaturase that utilizes O(2) and electrons from reduced cytochrome b5 to introduce the first double bond into saturated fatty acyl-CoA substrates (PubMed:15907797, PubMed:18765284). Catalyzes the insertion of a cis double bond at the delta-9 position into fatty acyl-CoA substrates including palmitoyl-CoA and stearoyl-CoA (PubMed:15907797, PubMed:18765284). Gives rise to a mixture of 16:1 and 18:1 unsaturated fatty acids (PubMed:15610069). Plays an important role in lipid biosynthesis. Plays an important role in regulating the e [...] |
| CD40LG | CD40 ligand; Cytokine that binds to CD40/TNFRSF5 (PubMed:1280226). Costimulates T-cell proliferation and cytokine production. Its cross-linking on T-cells generates a costimulatory signal which enhances the production of IL4 and IL10 in conjunction with the TCR/CD3 ligation and CD28 costimulation (PubMed:8617933). Induces the activation of NF-kappa-B and kinases MAPK8 and PAK2 in T- cells. Induces tyrosine phosphorylation of isoform 3 of CD28 (PubMed:15067037). Mediates B-cell proliferation in the absence of co-stimulus as well as IgE production in the presence of IL4. Involved in immu [...] |
| JUN | Jun proto-oncogene; Transcription factor that recognizes and binds to the enhancer heptamer motif 5'-TGA[CG]TCA-3'. Promotes activity of NR5A1 when phosphorylated by HIPK3 leading to increased steroidogenic gene expression upon cAMP signaling pathway stimulation. Involved in activated KRAS-mediated transcriptional activation of USP28 in colorectal cancer (CRC) cells (PubMed:24623306). Binds to the USP28 promoter in colorectal cancer (CRC) cells (PubMed:24623306) |
| RUNX2 | Runt-related transcription factor 2; Transcription factor involved in osteoblastic differentiation and skeletal morphogenesis. Essential for the maturation of osteoblasts and both intramembranous and endochondral ossification. CBF binds to the core site, 5'- PYGPYGGT-3', of a number of enhancers and promoters, including murine leukemia virus, polyomavirus enhancer, T-cell receptor enhancers, osteocalcin, osteopontin, bone sialoprotein, alpha 1(I) collagen, LCK, IL-3 and GM-CSF promoters. In osteoblasts, supports transcription activation: synergizes with SPEN/MINT to enhance FGFR2-media [...] |
| PTPN1 | Protein tyrosine phosphatase, non-receptor type 1; Tyrosine-protein phosphatase which acts as a regulator of endoplasmic reticulum unfolded protein response. Mediates dephosphorylation of EIF2AK3/PERK; inactivating the protein kinase activity of EIF2AK3/PERK. May play an important role in CKII- and p60c-src-induced signal transduction cascades. May regulate the EFNA5-EPHA3 signaling pathway which modulates cell reorganization and cell-cell repulsion. May also regulate the hepatocyte growth factor receptor signaling pathway through dephosphorylation of MET |
| VEGFA | Vascular endothelial growth factor A; Growth factor active in angiogenesis, vasculogenesis and endothelial cell growth. Induces endothelial cell proliferation, promotes cell migration, inhibits apoptosis and induces permeabilization of blood vessels. Binds to the FLT1/VEGFR1 and KDR/VEGFR2 receptors, heparan sulfate and heparin. NRP1/Neuropilin-1 binds isoforms VEGF-165 and VEGF-145. Isoform VEGF165B binds to KDR but does not activate downstream signaling pathways, does not activate angiogenesis and inhibits tumor growth |
| MMP9 | Matrix metallopeptidase 9 (gelatinase B, 92kDa gelatinase, 92kDa type IV collagenase); May play an essential role in local proteolysis of the extracellular matrix and in leukocyte migration. Could play a role in bone osteoclastic resorption. Cleaves KiSS1 at a Gly-\|-Leu bond. Cleaves type IV and type V collagen into large C-terminal three quarter fragments and shorter N-terminal one quarter fragments. Degrades fibronectin but not laminin or Pz-peptide |
| PLAU | Plasminogen activator, urokinase; Specifically cleaves the zymogen plasminogen to form the active enzyme plasmin |
| TLR4 | Toll-like receptor 4; Cooperates with LY96 and CD14 to mediate the innate immune response to bacterial lipopolysaccharide (LPS). Acts via MYD88, TIRAP and TRAF6, leading to NF-kappa-B activation, cytokine secretion and the inflammatory response (PubMed:9237759, PubMed:10835634). Also involved in LPS-independent inflammatory responses triggered by free fatty acids, such as palmitate, and Ni(2+). Responses triggered by Ni(2+) require non-conserved histidines and are, therefore, species-specific (PubMed:20711192). In complex with TLR6, promotes sterile inflammation in monocytes/macrophage [...] |
| ALOX5 | Arachidonate 5-lipoxygenase; Catalyzes the first step in leukotriene biosynthesis, and thereby plays a role in inflammatory processes |
| EIF6 | Eukaryotic translation initiation factor 6; Binds to the 60S ribosomal subunit and prevents its association with the 40S ribosomal subunit to form the 80S initiation complex in the cytoplasm. May behave as a stimulatory translation initiation factor downstream insulin/growth factors. Is also involved in ribosome biogenesis. Associates with pre-60S subunits in the nucleus and is involved in its nuclear export. Cytoplasmic release of TIF6 from 60S subunits and nuclear relocalization is promoted by a RACK1 (RACK1)-dependent protein kinase C activity |
| RXRB | Retinoid X receptor, beta; Receptor for retinoic acid. Retinoic acid receptors bind as heterodimers to their target response elements in response to their ligands, all-trans or 9-cis retinoic acid, and regulate gene expression in various biological processes. The RAR/RXR heterodimers bind to the retinoic acid response elements (RARE) |
| AR | Androgen receptor; Steroid hormone receptors are ligand-activated transcription factors that regulate eukaryotic gene expression and affect cellular proliferation and differentiation in target tissues. Transcription factor activity is modulated by bound coactivator and corepressor proteins. Transcription activation is down-regulated by NR0B2. Activated, but not phosphorylated, by HIPK3 and ZIPK/DAPK3 |
| PLA2G2E | Phospholipase A2, group IIE; PA2 catalyzes the calcium-dependent hydrolysis of the 2- acyl groups in 3-sn-phosphoglycerides. Has a preference for arachidonic-containing phospholipids |
| F7 | Coagulation factor VII (serum prothrombin conversion accelerator); Initiates the extrinsic pathway of blood coagulation. Serine protease that circulates in the blood in a zymogen form. Factor VII is converted to factor VIIa by factor Xa, factor XIIa, factor IXa, or thrombin by minor proteolysis. In the presence of tissue factor and calcium ions, factor VIIa then converts factor X to factor Xa by limited proteolysis. Factor VIIa will also convert factor IX to factor IXa in the presence of tissue factor and calcium |
| TNFRSF1B | Tumor necrosis factor receptor superfamily, member 1B; Receptor with high affinity for TNFSF2/TNF-alpha and approximately 5-fold lower affinity for homotrimeric TNFSF1/lymphotoxin-alpha. The TRAF1/TRAF2 complex recruits the apoptotic suppressors BIRC2 and BIRC3 to TNFRSF1B/TNFR2. This receptor mediates most of the metabolic effects of TNF-alpha. Isoform 2 blocks TNF-alpha-induced apoptosis, which suggests that it regulates TNF-alpha function by antagonizing its biological activity |
| THBD | Thrombomodulin; Thrombomodulin is a specific endothelial cell receptor that forms a 1:1 stoichiometric complex with thrombin. This complex is responsible for the conversion of protein C to the activated protein C (protein Ca). Once evolved, protein Ca scissions the activated cofactors of the coagulation mechanism, factor Va and factor VIIIa, and thereby reduces the amount of thrombin generated |
| MYC | V-myc myelocytomatosis viral oncogene homolog (avian); Transcription factor that binds DNA in a non-specific manner, yet also specifically recognizes the core sequence 5'- CAC[GA]TG-3'. Activates the transcription of growth-related genes |
| MAOB | Monoamine oxidase B; Catalyzes the oxidative deamination of biogenic and xenobiotic amines and has important functions in the metabolism of neuroactive and vasoactive amines in the central nervous system and peripheral tissues. MAOB preferentially degrades benzylamine and phenylethylamine |
| PRKCZ | Protein kinase C, zeta; Calcium- and diacylglycerol-independent serine/threonine-protein kinase that functions in phosphatidylinositol 3-kinase (PI3K) pathway and mitogen-activated protein (MAP) kinase cascade, and is involved in NF-kappa-B activation, mitogenic signaling, cell proliferation, cell polarity, inflammatory response and maintenance of long-term potentiation (LTP). Upon lipopolysaccharide (LPS) treatment in macrophages, or following mitogenic stimuli, functions downstream of PI3K to activate MAP2K1/MEK1-MAPK1/ERK2 signaling cascade independently of RAF1 activation. Required [...] |
| HTR2A | 5-hydroxytryptamine (serotonin) receptor 2A, G protein-coupled; G-protein coupled receptor for 5-hydroxytryptamine (serotonin). Also functions as a receptor for various drugs and psychoactive substances, including mescaline, psilocybin, 1-(2,5- dimethoxy-4-iodophenyl)-2-aminopropane (DOI) and lysergic acid diethylamide (LSD). Ligand binding causes a conformation change that triggers signaling via guanine nucleotide-binding proteins (G proteins) and modulates the activity of down-stream effectors. Beta-arrestin family members inhibit signaling via G proteins and mediate activation of al [...] |
| EDN1 | Endothelin 1; Endothelins are endothelium-derived vasoconstrictor peptides |
| XDH | Xanthine dehydrogenase; Key enzyme in purine degradation. Catalyzes the oxidation of hypoxanthine to xanthine. Catalyzes the oxidation of xanthine to uric acid. Contributes to the generation of reactive oxygen species. Has also low oxidase activity towards aldehydes (in vitro) |
| ADRA1D | Adrenoceptor alpha 1D; This alpha-adrenergic receptor mediates its effect through the influx of extracellular calcium |
| TRPC3 | Transient receptor potential cation channel, subfamily C, member 3; Thought to form a receptor-activated non-selective calcium permeant cation channel. Probably is operated by a phosphatidylinositol second messenger system activated by receptor tyrosine kinases or G-protein coupled receptors. Activated by diacylglycerol (DAG) in a membrane-delimited fashion, independently of protein kinase C, and by inositol 1,4,5- triphosphate receptors (ITPR) with bound IP3. May also be activated by internal calcium store depletion |
| CYP1A1 | Cytochrome P450, family 1, subfamily A, polypeptide 1; Cytochromes P450 are a group of heme-thiolate monooxygenases. In liver microsomes, this enzyme is involved in an NADPH-dependent electron transport pathway. It oxidizes a variety of structurally unrelated compounds, including steroids, fatty acids, and xenobiotics |
| IL2RA | Interleukin 2 receptor, alpha; Receptor for interleukin-2. The receptor is involved in the regulation of immune tolerance by controlling regulatory T cells (TREGs) activity. TREGs suppress the activation and expansion of autoreactive T-cells |
| AKR1C3 | Aldo-keto reductase family 1, member C3 (3-alpha hydroxysteroid dehydrogenase, type II); Catalyzes the conversion of aldehydes and ketones to alcohols. Catalyzes the reduction of prostaglandin (PG) D2, PGH2 and phenanthrenequinone (PQ) and the oxidation of 9-alpha,11-beta- PGF2 to PGD2. Functions as a bi-directional 3-alpha-, 17-beta- and 20-alpha HSD. Can interconvert active androgens, estrogens and progestins with their cognate inactive metabolites. Preferentially transforms androstenedione (4-dione) to testosterone |
| NKX3-1 | NK3 homeobox 1; Transcription factor, which binds preferentially the consensus sequence 5'-TAAGT[AG]-3' and can behave as a transcriptional repressor. Plays an important role in normal prostate development, regulating proliferation of glandular epithelium and in the formation of ducts in prostate. Acts as a tumor suppressor controlling prostate carcinogenesis, as shown by the ability to inhibit proliferation and invasion activities of PC-3 prostate cancer cells |
| PDX1 | Pancreatic and duodenal homeobox 1; Activates insulin, somatostatin, glucokinase, islet amyloid polypeptide and glucose transporter type 2 gene transcription. Particularly involved in glucose-dependent regulation of insulin gene transcription. As part of a PDX1:PBX1b:MEIS2b complex in pancreatic acinar cells is involved in the transcriptional activation of the ELA1 enhancer; the complex binds to the enhancer B element and cooperates with the transcription factor 1 complex (PTF1) bound to the enhancer A element. Binds preferentially the DNA motif 5'-[CT]TAAT[TG]-3'. During development, [...] |
| IGFBP3 | Insulin-like growth factor binding protein 3; IGF-binding proteins prolong the half-life of the IGFs and have been shown to either inhibit or stimulate the growth promoting effects of the IGFs on cell culture. They alter the interaction of IGFs with their cell surface receptors. Also exhibits IGF-independent antiproliferative and apoptotic effects mediated by its receptor TMEM219/IGFBP-3R |
| CHEK2 | Checkpoint kinase 2 |
| CHRM5 | Cholinergic receptor, muscarinic 5; The muscarinic acetylcholine receptor mediates various cellular responses, including inhibition of adenylate cyclase, breakdown of phosphoinositides and modulation of potassium channels through the action of G proteins. Primary transducing effect is Pi turnover |
| DRD3 | Dopamine receptor D3; Dopamine receptor whose activity is mediated by G proteins which inhibit adenylyl cyclase. Promotes cell proliferation |
| NOX5 | NADPH oxidase, EF-hand calcium binding domain 5; Calcium-dependent NADPH oxidase that generates superoxide. Also functions as a calcium-dependent proton channel and may regulate redox-dependent processes in lymphocytes and spermatozoa. May play a role in cell growth and apoptosis. Isoform v2 and isoform v5 are involved in endothelial generation of reactive oxygen species (ROS), proliferation and angiogenesis and contribute to endothelial response to thrombin |
| TYRP1 | Tyrosinase-related protein 1; Oxidation of 5,6-dihydroxyindole-2-carboxylic acid (DHICA) into indole-5,6-quinone-2-carboxylic acid. May regulate or influence the type of melanin synthesized |
| DUOX2 | Dual oxidase 2; Generates hydrogen peroxide which is required for the activity of thyroid peroxidase/TPO and lactoperoxidase/LPO. Plays a role in thyroid hormones synthesis and lactoperoxidase-mediated antimicrobial defense at the surface of mucosa. May have its own peroxidase activity through its N-terminal peroxidase-like domain |
| ATP2C1 | ATPase, Ca++ transporting, type 2C, member 1; This magnesium-dependent enzyme catalyzes the hydrolysis of ATP coupled with the transport of the calcium |
| GAP43 | Growth associated protein 43; This protein is associated with nerve growth. It is a major component of the motile "growth cones" that form the tips of elongating axons. Plays a role in axonal and dendritic filopodia induction |
| SPP1 | Secreted phosphoprotein 1; Binds tightly to hydroxyapatite. Appears to form an integral part of the mineralized matrix. Probably important to cell-matrix interaction |
| CDK1 | Cyclin-dependent kinase 1; Plays a key role in the control of the eukaryotic cell cycle by modulating the centrosome cycle as well as mitotic onset; promotes G2-M transition, and regulates G1 progress and G1-S transition via association with multiple interphase cyclins. Required in higher cells for entry into S-phase and mitosis. Phosphorylates PARVA/actopaxin, APC, AMPH, APC, BARD1, Bcl- xL/BCL2L1, BRCA2, CALD1, CASP8, CDC7, CDC20, CDC25A, CDC25C, CC2D1A, CSNK2 proteins/CKII, FZR1/CDH1, CDK7, CEBPB, CHAMP1, DMD/dystrophin, EEF1 proteins/EF-1, EZH2, KIF11/EG5, EGFR, FANCG, FOS, GFAP, G [...] |
| NFE2L2 | Nuclear factor (erythroid-derived 2)-like 2; Transcription activator that binds to antioxidant response (ARE) elements in the promoter regions of target genes. Important for the coordinated up-regulation of genes in response to oxidative stress. May be involved in the transcriptional activation of genes of the beta-globin cluster by mediating enhancer activity of hypersensitive site 2 of the beta-globin locus control region |
| GSTP1 | Glutathione S-transferase pi 1; Conjugation of reduced glutathione to a wide number of exogenous and endogenous hydrophobic electrophiles. Regulates negatively CDK5 activity via p25/p35 translocation to prevent neurodegeneration |
| TRPV1 | Transient receptor potential cation channel, subfamily V, member 1; Ligand-activated non-selective calcium permeant cation channel involved in detection of noxious chemical and thermal stimuli. Seems to mediate proton influx and may be involved in intracellular acidosis in nociceptive neurons. Involved in mediation of inflammatory pain and hyperalgesia. Sensitized by a phosphatidylinositol second messenger system activated by receptor tyrosine kinases, which involves PKC isozymes and PCL. Can be activated by endogenous compounds, including 12- hydroperoxytetraenoic acid and bradykinin. [...] |
| RELA | V-rel reticuloendotheliosis viral oncogene homolog A (avian); NF-kappa-B is a pleiotropic transcription factor present in almost all cell types and is the endpoint of a series of signal transduction events that are initiated by a vast array of stimuli related to many biological processes such as inflammation, immunity, differentiation, cell growth, tumorigenesis and apoptosis. NF-kappa-B is a homo- or heterodimeric complex formed by the Rel-like domain-containing proteins RELA/p65, RELB, NFKB1/p105, NFKB1/p50, REL and NFKB2/p52 and the heterodimeric p65-p50 complex appears to be most a [...] |
| CHRNA2 | Cholinergic receptor, nicotinic, alpha 2 (neuronal); After binding acetylcholine, the AChR responds by an extensive change in conformation that affects all subunits and leads to opening of an ion-conducting channel across the plasma membrane |
| ADRA2C | Adrenoceptor alpha 2C; Alpha-2 adrenergic receptors mediate the catecholamine- induced inhibition of adenylate cyclase through the action of G proteins |
| ITGA6 | Integrin, alpha 6; Integrin alpha-6/beta-1 is a receptor for laminin on platelets. Integrin alpha-6/beta-4 is a receptor for laminin in epithelial cells and it plays a critical structural role in the hemidesmosome |
| ADRA2B | Adrenoceptor alpha 2B; Alpha-2 adrenergic receptors mediate the catecholamine- induced inhibition of adenylate cyclase through the action of G proteins. The rank order of potency for agonists of this receptor is clonidine > norepinephrine > epinephrine = oxymetazoline > dopamine > p-tyramine = phenylephrine > serotonin > p-synephrine / p-octopamine. For antagonists, the rank order is yohimbine > chlorpromazine > phentolamine > mianserine > spiperone > prazosin > alprenolol > propanolol > pindolol |
| GCG | Glucagon; Glicentin may modulate gastric acid secretion and the gastro-pyloro-duodenal activity. May play an important role in intestinal mucosal growth in the early period of life |
| CREB1 | cAMP responsive element binding protein 1; Phosphorylation-dependent transcription factor that stimulates transcription upon binding to the DNA cAMP response element (CRE), a sequence present in many viral and cellular promoters. Transcription activation is enhanced by the TORC coactivators which act independently of Ser-133 phosphorylation. Involved in different cellular processes including the synchronization of circadian rhythmicity and the differentiation of adipose cells |
| CITED1 | Cbp/p300-interacting transactivator, with Glu/Asp-rich carboxy-terminal domain, 1; Transcriptional coactivator of the p300/CBP-mediated trancription complex. Enhances SMAD-mediated transcription by strengthening the functional link between the DNA-binding SMAD transcription factors and the p300/CBP transcription coactivator complex. Stimulates estrogen-dependent transactivation activity mediated by estrogen receptors signaling; stabilizes the interaction of estrogen receptor ESR1 and histone acetyltransferase EP300. Positively regulates TGF-beta signaling through its association with t [...] |
| CHEK1 | Checkpoint kinase 1; Serine/threonine-protein kinase which is required for checkpoint-mediated cell cycle arrest and activation of DNA repair in response to the presence of DNA damage or unreplicated DNA. May also negatively regulate cell cycle progression during unperturbed cell cycles. This regulation is achieved by a number of mechanisms that together help to preserve the integrity of the genome. Recognizes the substrate consensus sequence [R-X-X-S/T]. Binds to and phosphorylates CDC25A, CDC25B and CDC25C. Phosphorylation of CDC25A at 'Ser-178' and 'Thr-507' and phosphorylation of C [...] |
| TBXAS1 | Thromboxane A synthase 1 (platelet) |
| PTPN6 | Protein tyrosine phosphatase, non-receptor type 6; Modulates signaling by tyrosine phosphorylated cell surface receptors such as KIT and the EGF receptor/EGFR. The SH2 regions may interact with other cellular components to modulate its own phosphatase activity against interacting substrates. Together with MTUS1, induces UBE2V2 expression upon angiotensin II stimulation. Plays a key role in hematopoiesis |
| IGF2 | Insulin-like growth factor 2 (somatomedin A); The insulin-like growth factors possess growth-promoting activity. In vitro, they are potent mitogens for cultured cells. IGF-II is influenced by placental lactogen and may play a role in fetal development |
| TBXA2R | Thromboxane A2 receptor; Receptor for thromboxane A2 (TXA2), a potent stimulator of platelet aggregation. The activity of this receptor is mediated by a G-protein that activates a phosphatidylinositol-calcium second messenger system. In the kidney, the binding of TXA2 to glomerular TP receptors causes intense vasoconstriction. Activates phospholipase C. Isoform 1 activates adenylyl cyclase. Isoform 2 inhibits adenylyl cyclase |
| OPRM1 | Opioid receptor, mu 1; Receptor for endogenous opioids such as beta-endorphin and endomorphin. Receptor for natural and synthetic opioids including morphine, heroin, DAMGO, fentanyl, etorphine, buprenorphin and methadone. Agonist binding to the receptor induces coupling to an inactive GDP-bound heterotrimeric G-protein complex and subsequent exchange of GDP for GTP in the G-protein alpha subunit leading to dissociation of the G-protein complex with the free GTP-bound G-protein alpha and the G-protein beta- gamma dimer activating downstream cellular effectors. The agonist- and cell type [...] |
| TOP2B | Topoisomerase (DNA) II beta 180kDa; Control of topological states of DNA by transient breakage and subsequent rejoining of DNA strands. Topoisomerase II makes double-strand breaks |
| AMY2A | Amylase, alpha 2A (pancreatic) |
| TNF | Tumor necrosis factor; Cytokine that binds to TNFRSF1A/TNFR1 and TNFRSF1B/TNFBR. It is mainly secreted by macrophages and can induce cell death of certain tumor cell lines. It is potent pyrogen causing fever by direct action or by stimulation of interleukin-1 secretion and is implicated in the induction of cachexia, Under certain conditions it can stimulate cell proliferation and induce cell differentiation. Impairs regulatory T-cells (Treg) function in individuals with rheumatoid arthritis via FOXP3 dephosphorylation. Upregulates the expression of protein phosphatase 1 (PP1), which de [...] |
| NCOA2 | Nuclear receptor coactivator 2; Transcriptional coactivator for steroid receptors and nuclear receptors. Coactivator of the steroid binding domain (AF- 2) but not of the modulating N-terminal domain (AF-1). Required with NCOA1 to control energy balance between white and brown adipose tissues. Critical regulator of glucose metabolism regulation, acts as RORA coactivator to specifically modulate G6PC expression. Involved in the positive regulation of the transcriptional activity of the glucocorticoid receptor NR3C1 by sumoylation enhancer RWDD3. Positively regulates the circadian clock b [...] |
| RHOA | Ras homolog family member A; Regulates a signal transduction pathway linking plasma membrane receptors to the assembly of focal adhesions and actin stress fibers. Involved in a microtubule-dependent signal that is required for the myosin contractile ring formation during cell cycle cytokinesis. Plays an essential role in cleavage furrow formation. Required for the apical junction formation of keratinocyte cell-cell adhesion. Stimulates PKN2 kinase activity. May be an activator of PLCE1. Activated by ARHGEF2, which promotes the exchange of GDP for GTP. Essential for the SPATA13-mediated [...] |
| RUNX1T1 | Runt-related transcription factor 1; translocated to, 1 (cyclin D-related); Transcriptional corepressor which facilitates transcriptional repression via its association with DNA-binding transcription factors and recruitment of other corepressors and histone-modifying enzymes (PubMed:12559562, PubMed:15203199). Can repress the expression of MMP7 in a ZBTB33-dependent manner (PubMed:23251453). Can repress transactivation mediated by TCF12 (PubMed:16803958). Acts as a negative regulator of adipogenesis (By similarity) |
| BBC3 | BCL2 binding component 3; Essential mediator of p53/TP53-dependent and p53/TP53- independent apoptosis. Isoform 3 fails to show any growth- inhibitory or apoptotic activity |
| PRKCA | Protein kinase C, alpha; Calcium-activated, phospholipid- and diacylglycerol (DAG)-dependent serine/threonine-protein kinase that is involved in positive and negative regulation of cell proliferation, apoptosis, differentiation, migration and adhesion, tumorigenesis, cardiac hypertrophy, angiogenesis, platelet function and inflammation, by directly phosphorylating targets such as RAF1, BCL2, CSPG4, TNNT2/CTNT, or activating signaling cascade involving MAPK1/3 (ERK1/2) and RAP1GAP. Involved in cell proliferation and cell growth arrest by positive and negative regulation of the cell cycl [...] |
| CHRM4 | Cholinergic receptor, muscarinic 4; The muscarinic acetylcholine receptor mediates various cellular responses, including inhibition of adenylate cyclase, breakdown of phosphoinositides and modulation of potassium channels through the action of G proteins. Primary transducing effect is inhibition of adenylate cyclase |
| CASP1 | Caspase 1, apoptosis-related cysteine peptidase; Thiol protease that cleaves IL-1 beta between an Asp and an Ala, releasing the mature cytokine which is involved in a variety of inflammatory processes. Important for defense against pathogens. Cleaves and activates sterol regulatory element binding proteins (SREBPs). Can also promote apoptosis |
| TOP2A | Topoisomerase (DNA) II alpha 170kDa; Control of topological states of DNA by transient breakage and subsequent rejoining of DNA strands. Topoisomerase II makes double-strand breaks. Essential during mitosis and meiosis for proper segregation of daughter chromosomes. May play a role in regulating the period length of ARNTL/BMAL1 transcriptional oscillation (By similarity) |
| IL10 | Interleukin 10; Inhibits the synthesis of a number of cytokines, including IFN-gamma, IL-2, IL-3, TNF and GM-CSF produced by activated macrophages and by helper T-cells |
| BDNF | Brain-derived neurotrophic factor; During development, promotes the survival and differentiation of selected neuronal populations of the peripheral and central nervous systems. Participates in axonal growth, pathfinding and in the modulation of dendritic growth and morphology. Major regulator of synaptic transmission and plasticity at adult synapses in many regions of the CNS. The versatility of BDNF is emphasized by its contribution to a range of adaptive neuronal responses including long-term potentiation (LTP), long-term depression (LTD), certain forms of short-term synaptic plastic [...] |
| TK2 | Thymidine kinase 2, mitochondrial; Deoxyribonucleoside kinase that phosphorylates thymidine, deoxycytidine, and deoxyuridine. Also phosphorylates anti-viral and anti-cancer nucleoside analogs |
| MDM2 | Mdm2, p53 E3 ubiquitin protein ligase homolog (mouse); E3 ubiquitin-protein ligase that mediates ubiquitination of p53/TP53, leading to its degradation by the proteasome. Inhibits p53/TP53- and p73/TP73-mediated cell cycle arrest and apoptosis by binding its transcriptional activation domain. Also acts as a ubiquitin ligase E3 toward itself and ARRB1. Permits the nuclear export of p53/TP53. Promotes proteasome-dependent ubiquitin-independent degradation of retinoblastoma RB1 protein. Inhibits DAXX-mediated apoptosis by inducing its ubiquitination and degradation. Component of the TRIM2 [...] |
| RXRA | Retinoid X receptor, alpha; Receptor for retinoic acid. Retinoic acid receptors bind as heterodimers to their target response elements in response to their ligands, all-trans or 9-cis retinoic acid, and regulate gene expression in various biological processes. The RAR/RXR heterodimers bind to the retinoic acid response elements (RARE) composed of tandem 5'-AGGTCA-3' sites known as DR1-DR5. The high affinity ligand for RXRs is 9-cis retinoic acid. RXRA serves as a common heterodimeric partner for a number of nuclear receptors. The RXR/RAR heterodimers bind to the retinoic acid response [...] |
| POR | P450 (cytochrome) oxidoreductase; This enzyme is required for electron transfer from NADP to cytochrome P450 in microsomes. It can also provide electron transfer to heme oxygenase and cytochrome B5 |
| CXCL2 | Chemokine (C-X-C motif) ligand 2; Produced by activated monocytes and neutrophils and expressed at sites of inflammation. Hematoregulatory chemokine, which, in vitro, suppresses hematopoietic progenitor cell proliferation. GRO-beta(5-73) shows a highly enhanced hematopoietic activity |
| HSF1 | Heat shock transcription factor 1; DNA-binding protein that specifically binds heat shock promoter elements (HSE) and activates transcription. In higher eukaryotes, HSF is unable to bind to the HSE unless the cells are heat shocked |
| NR1H4 | Nuclear receptor subfamily 1, group H, member 4; Isoform 4: Promotes transcriptional activation of target genes ABCB11/BSEP (inducible by unconjugated CDCA, ACA and DCA), NR0B2/SHP (inducible by unconjugated CDCA, ACA and DCA), SLC51B/OSTB (inducible by unconjugated CDCA and DCA) and FABP6/IBAP; most efficient isoform compared to isoforms 1 to 3; not inducible by taurine- and glycine-amidated CDCA |
| MGAM | Maltase-glucoamylase (alpha-glucosidase); May serve as an alternate pathway for starch digestion when luminal alpha-amylase activity is reduced because of immaturity or malnutrition. May play a unique role in the digestion of malted dietary oligosaccharides used in food manufacturing |
| TRPV3 | Transient receptor potential cation channel, subfamily V, member 3; Putative receptor-activated non-selective calcium permeant cation channel. It is activated by innocuous (warm) temperatures and shows an increased response at noxious temperatures greater than 39 degrees Celsius. Activation exhibits an outward rectification. May associate with TRPV1 and may modulate its activity. Is a negative regulator of hair growth and cycling: TRPV3-coupled signaling suppresses keratinocyte proliferation in hair follicles and induces apoptosis and premature hair follicle regression (catagen) |

Supplementary table 3. Target proteins and annotation of HLJDD.
