# Supplementary material for: Intermodule Coupling Analysis of Huang-Lian-Jie-Du Decoction on Stroke
Source: Front Pharmacol. 2019 Nov 5;10:1288. doi: 10.3389/fphar.2019.01288 (PMC6848980; doi:10.3389/fphar.2019.01288)
Supplement: Table S4 — The enriched KEGG pathways and their categories of stroke-modules. [file Table_4.docx]

Supplementary table 4

| **Module** | **Category** | **KFGG term** | **P-Value** | **Genes** |
| --- | --- | --- | --- | --- |
| Stroke-module 1 | 1.5 Amino acid metabolism | hsa00220:Arginine biosynthesis | 0.001941654 | NOS1, NOS3, NOS2 |
| Stroke-module 1 | 1.5 Amino acid metabolism | hsa00330:Arginine and proline metabolism | 0.011782862 | NOS1, NOS3, NOS2 |
| Stroke-module 1 | 3.2 Signal transduction | hsa04020:Calcium signaling pathway | 1.94E-05 | AGTR1, NOS1, GNAQ, TBXA2R, NOS3, NOS2, HTR2A |
| Stroke-module 1 | 3.2 Signal transduction | hsa04668:TNF signaling pathway | 0.004970699 | VCAM1, TNF, MMP9, PIK3CA |
| Stroke-module 1 | 3.2 Signal transduction | hsa04071:Sphingolipid signaling pathway | 0.007019938 | TNF, GNAQ, PIK3CA, NOS3 |
| Stroke-module 1 | 3.2 Signal transduction | hsa04022:cGMP-PKG signaling pathway | 0.016941816 | AGTR1, GNAQ, PIK3CA, NOS3 |
| Stroke-module 1 | 3.2 Signal transduction | hsa04066:HIF-1 signaling pathway | 0.041519828 | PIK3CA, NOS3, NOS2 |
| Stroke-module 1 | 3.3 Signaling molecules and interaction | hsa04080:Neuroactive ligand-receptor interaction | 0.012249219 | AGTR1, F2, TBXA2R, PLG, HTR2A |
| Stroke-module 1 | 5.1 Immune system | hsa04611:Platelet activation | 0.008751187 | GNAQ, TBXA2R, PIK3CA, NOS3 |
| Stroke-module 1 | 5.1 Immune system | hsa04610:Complement and coagulation cascades | 0.021719375 | THBD, F2, PLG |
| Stroke-module 1 | 5.10 Environmental adaptation | hsa04713:Circadian entrainment | 0.039238622 | NOS1, GNAQ, GNB3 |
| Stroke-module 1 | 5.2 Endocrine system | hsa04915:Estrogen signaling pathway | 0.00410306 | GNAQ, MMP9, PIK3CA, NOS3 |
| Stroke-module 1 | 5.4 Digestive system | hsa04975:Fat digestion and absorption | 0.007287247 | APOA1, ABCA1, MTTP |
| Stroke-module 1 | 5.6 Nervous system | hsa04726:Serotonergic synapse | 0.005653639 | APP, GNAQ, GNB3, HTR2A |
| Stroke-module 1 | 5.7 Sensory system | hsa04750:Inflammatory mediator regulation of TRP channels | 0.041519828 | GNAQ, PIK3CA, HTR2A |
| Stroke-module 1 | 6.1 Cancers: Overview | hsa05200:Pathways in cancer | 0.008329501 | AGTR1, GNAQ, MMP9, PIK3CA, GNB3, NOS2 |
| Stroke-module 1 | 6.10 Infectious diseases: Parasitic | hsa05143:African trypanosomiasis | 1.65E-04 | VCAM1, TNF, APOA1, GNAQ |
| Stroke-module 1 | 6.10 Infectious diseases: Parasitic | hsa05142:Chagas disease (American trypanosomiasis) | 0.004712389 | TNF, GNAQ, PIK3CA, NOS2 |
| Stroke-module 1 | 6.10 Infectious diseases: Parasitic | hsa05146:Amoebiasis | 0.004970699 | TNF, GNAQ, PIK3CA, NOS2 |
| Stroke-module 1 | 6.4 Neurodegenerative diseases | hsa05010:Alzheimer's disease | 1.89E-04 | LPL, APP, TNF, NOS1, GNAQ, APOE |
| Stroke-module 1 | 6.7 Endocrine and metabolic diseases | hsa04931:Insulin resistance | 0.005237464 | SREBF1, TNF, PIK3CA, NOS3 |
| Stroke-module 2 | 1.2 Energy metabolism | hsa00190:Oxidative phosphorylation | 4.97E-18 | NDUFB3, NDUFS6, NDUFS4, NDUFB9, NDUFV1, NDUFV2, NDUFS3, NDUFA1, NDUFS2, NDUFS1, NDUFA11 |
| Stroke-module 2 | 6.4 Neurodegenerative diseases | hsa05012:Parkinson's disease | 9.78E-18 | NDUFB3, NDUFS6, NDUFS4, NDUFB9, NDUFV1, NDUFV2, NDUFS3, NDUFA1, NDUFS2, NDUFS1, NDUFA11 |
| Stroke-module 2 | 6.7 Endocrine and metabolic diseases | hsa04932:Non-alcoholic fatty liver disease (NAFLD) | 1.84E-17 | NDUFB3, NDUFS6, NDUFS4, NDUFB9, NDUFV1, NDUFV2, NDUFS3, NDUFA1, NDUFS2, NDUFS1, NDUFA11 |
| Stroke-module 2 | 6.4 Neurodegenerative diseases | hsa05010:Alzheimer's disease | 5.53E-17 | NDUFB3, NDUFS6, NDUFS4, NDUFB9, NDUFV1, NDUFV2, NDUFS3, NDUFA1, NDUFS2, NDUFS1, NDUFA11 |
| Stroke-module 2 | 6.4 Neurodegenerative diseases | hsa05016:Huntington's disease | 2.18E-16 | NDUFB3, NDUFS6, NDUFS4, NDUFB9, NDUFV1, NDUFV2, NDUFS3, NDUFA1, NDUFS2, NDUFS1, NDUFA11 |
| Stroke-module 2 | 1.0 Global and overview maps | hsa01100:Metabolic pathways | 3.05E-08 | NDUFB3, NDUFS6, NDUFS4, NDUFB9, NDUFV1, NDUFV2, NDUFS3, NDUFA1, NDUFS2, NDUFS1, NDUFA11 |
| Stroke-module 3 | 5.1 Immune system | hsa04610:Complement and coagulation cascades | 6.19E-07 | A2M, F5, FGB, F8, PROC |
| Stroke-module 4 | 5.1 Immune system | hsa04640:Hematopoietic cell lineage | 0.003017118 | IL4, ITGA2, EPO |
| Stroke-module 4 | 3.2 Signal transduction | hsa04151:PI3K-Akt signaling pathway | 0.044180666 | IL4, ITGA2, EPO |
| Stroke-module 4 | 6.10 Infectious diseases: Parasitic | hsa05144:Malaria | 0.048615281 | SELP, SELE |
| Stroke-module 5 | 5.4 Digestive system | hsa04971:Gastric acid secretion | 6.52E-04 | KCNE2, KCNJ2, KCNQ1 |
| Stroke-module 7 | 3.3 Signaling molecules and interaction | hsa04512:ECM-receptor interaction | 4.66E-04 | COL4A2, COL4A1, COL3A1 |
| Stroke-module 7 | 5.4 Digestive system | hsa04974:Protein digestion and absorption | 4.77E-04 | COL4A2, COL4A1, COL3A1 |
| Stroke-module 7 | 6.10 Infectious diseases: Parasitic | hsa05146:Amoebiasis | 6.92E-04 | COL4A2, COL4A1, COL3A1 |
| Stroke-module 7 | 4.3 Cellular community - eukaryotes | hsa04510:Focal adhesion | 0.002601439 | COL4A2, COL4A1, COL3A1 |
| Stroke-module 7 | 3.2 Signal transduction | hsa04151:PI3K-Akt signaling pathway | 0.007210848 | COL4A2, COL4A1, COL3A1 |
| Stroke-module 7 | 6.2 Cancers: Specific types | hsa05222:Small cell lung cancer | 0.036456167 | COL4A2, COL4A1 |
| Stroke-module 8 | 1.5 Amino acid metabolism | hsa00280:Valine, leucine and isoleucine degradation | 2.95E-07 | MUT, MCCC1, PCCB, PCCA |
| Stroke-module 8 | 1.1 Carbohydrate metabolism | hsa00630:Glyoxylate and dicarboxylate metabolism | 4.40E-05 | MUT, PCCB, PCCA |
| Stroke-module 8 | 1.1 Carbohydrate metabolism | hsa00640:Propanoate metabolism | 4.74E-05 | MUT, PCCB, PCCA |
| Stroke-module 8 | 1.0 Global and overview maps | hsa01200:Carbon metabolism | 7.87E-04 | MUT, PCCB, PCCA |
| Stroke-module 8 | 1.0 Global and overview maps | hsa01100:Metabolic pathways | 0.005601287 | MUT, MCCC1, PCCB, PCCA |
| Stroke-module 9 | 1.5 Amino acid metabolism | hsa00220:Arginine biosynthesis | 2.07E-08 | ARG1, ASS1, OTC, ASL |
| Stroke-module 9 | 1.0 Global and overview maps | hsa01230:Biosynthesis of amino acids | 1.18E-06 | ARG1, ASS1, OTC, ASL |
| Stroke-module 9 | 1.0 Global and overview maps | hsa01130:Biosynthesis of antibiotics | 2.85E-05 | ARG1, ASS1, OTC, ASL |
| Stroke-module 9 | 1.0 Global and overview maps | hsa01100:Metabolic pathways | 0.005601287 | ARG1, ASS1, OTC, ASL |
| Stroke-module 9 | 1.5 Amino acid metabolism | hsa00250:Alanine, aspartate and glutamate metabolism | 0.01512071 | ASS1, ASL |

Supplementary table 4. The enriched KEGG pathways and their categories of stroke-modules.
