# Supplementary material for: Intermodule Coupling Analysis of Huang-Lian-Jie-Du Decoction on Stroke
Source: Front Pharmacol. 2019 Nov 5;10:1288. doi: 10.3389/fphar.2019.01288 (PMC6848980; doi:10.3389/fphar.2019.01288)
Supplement: Table S5 — The enriched KEGG pathways and their categories of HLJDD-modules. [file Table_5.docx]

Supplementary table 5

| **Module** | **Category** | **KEGG Term** | **P-Value** | **Genes** |
| --- | --- | --- | --- | --- |
| HLJDD-moudle 1 | 3.2 Signal transduction | hsa04668:TNF signaling pathway | 1.52E-14 | ICAM1, CSF2, IL6, TNF, CCL2, PTGS2, RELA, MMP9, CXCL2, EDN1, CXCL10, MAPK1, FOS, JUN |
| HLJDD-moudle 1 | 3.2 Signal transduction | hsa04066:HIF-1 signaling pathway | 3.85E-09 | MAPK1, IL6, INS, RELA, BCL2, EDN1, VEGFA, IFNG, TLR4, STAT3 |
| HLJDD-moudle 1 | 3.2 Signal transduction | hsa04151:PI3K-Akt signaling pathway | 4.75E-09 | IL4, IL6, IL2RA, RELA, TP53, TLR4, BCL2L1, MAPK1, INS, CHRM2, BCL2, VEGFA, FGF2, MYC, IL2 |
| HLJDD-moudle 1 | 3.2 Signal transduction | hsa04630:Jak-STAT signaling pathway | 7.80E-09 | IL4, CSF2, IL6, IL2RA, IFNG, IL13, BCL2L1, MYC, IL10, STAT3, IL2 |
| HLJDD-moudle 1 | 3.2 Signal transduction | hsa04064:NF-kappa B signaling pathway | 1.06E-05 | ICAM1, TNF, PTGS2, RELA, BCL2, TLR4, BCL2L1 |
| HLJDD-moudle 1 | 3.2 Signal transduction | hsa04010:MAPK signaling pathway | 6.58E-04 | MAPK1, FOS, TNF, JUN, RELA, TP53, FGF2, MYC |
| HLJDD-moudle 1 | 3.2 Signal transduction | hsa04071:Sphingolipid signaling pathway | 6.60E-04 | MAPK1, TNF, RELA, BCL2, TP53, OPRD1 |
| HLJDD-moudle 1 | 3.2 Signal transduction | hsa04022:cGMP-PKG signaling pathway | 0.002805 | MAPK1, INS, ADRA2A, ADRA2C, ADRA2B, OPRD1 |
| HLJDD-moudle 1 | 3.2 Signal transduction | hsa04024:cAMP signaling pathway | 0.005951 | MAPK1, FOS, PTGER3, CHRM2, JUN, RELA |
| HLJDD-moudle 1 | 3.2 Signal transduction | hsa04068:FoxO signaling pathway | 0.007858 | MAPK1, IL6, INS, IL10, STAT3 |
| HLJDD-moudle 1 | 3.2 Signal transduction | hsa04014:Ras signaling pathway | 0.010277 | MAPK1, INS, RELA, VEGFA, BCL2L1, FGF2 |
| HLJDD-moudle 1 | 3.2 Signal transduction | hsa04350:TGF-beta signaling pathway | 0.01325 | MAPK1, TNF, IFNG, MYC |
| HLJDD-moudle 1 | 3.2 Signal transduction | hsa04150:mTOR signaling pathway | 0.046074 | MAPK1, TNF, INS |
| HLJDD-moudle 1 | 3.3 Signaling molecules and interaction | hsa04060:Cytokine-cytokine receptor interaction | 4.67E-09 | IL4, CSF2, IL6, TNF, CCL2, IL2RA, VEGFA, IFNG, IL13, CXCL11, IL10, CXCL10, IL2 |
| HLJDD-moudle 1 | 3.3 Signaling molecules and interaction | hsa04080:Neuroactive ligand-receptor interaction | 2.66E-05 | OPRM1, PTGER3, CHRM4, C5AR1, DRD3, CHRM2, ADRA2A, ADRA2C, ADRA2B, OPRD1 |
| HLJDD-moudle 1 | 4.2 Cell growth and death | hsa04210:Apoptosis | 4.64E-04 | TNF, RELA, BCL2, TP53, BCL2L1 |
| HLJDD-moudle 1 | 4.3 Cellular community - eukaryotes | hsa04550:Signaling pathways regulating pluripotency of stem cells | 0.049603 | MAPK1, FGF2, MYC, STAT3 |
| HLJDD-moudle 1 | 4.5 Cell motility | hsa04810:Regulation of actin cytoskeleton | 0.035446 | MAPK1, CHRM4, CHRM2, INS, FGF2 |
| HLJDD-moudle 1 | 5.1 Immune system | hsa04660:T cell receptor signaling pathway | 6.02E-09 | IL4, CSF2, MAPK1, FOS, TNF, JUN, RELA, IFNG, IL10, IL2 |
| HLJDD-moudle 1 | 5.1 Immune system | hsa04620:Toll-like receptor signaling pathway | 1.48E-07 | MAPK1, FOS, IL6, TNF, JUN, RELA, TLR4, CXCL11, CXCL10 |
| HLJDD-moudle 1 | 5.1 Immune system | hsa04062:Chemokine signaling pathway | 9.47E-05 | MAPK1, CCL2, RELA, CXCL2, CXCL11, CCL16, STAT3, CXCL10 |
| HLJDD-moudle 1 | 5.1 Immune system | hsa04621:NOD-like receptor signaling pathway | 2.92E-04 | MAPK1, IL6, TNF, CCL2, RELA |
| HLJDD-moudle 1 | 5.1 Immune system | hsa04664:Fc epsilon RI signaling pathway | 6.60E-04 | IL4, CSF2, MAPK1, TNF, IL13 |
| HLJDD-moudle 1 | 5.1 Immune system | hsa04640:Hematopoietic cell lineage | 0.001527 | IL4, CSF2, IL6, TNF, IL2RA |
| HLJDD-moudle 1 | 5.1 Immune system | hsa04672:Intestinal immune network for IgA production | 0.002623 | IL4, IL6, IL10, IL2 |
| HLJDD-moudle 1 | 5.1 Immune system | hsa04650:Natural killer cell mediated cytotoxicity | 0.005657 | CSF2, ICAM1, MAPK1, TNF, IFNG |
| HLJDD-moudle 1 | 5.1 Immune system | hsa04662:B cell receptor signaling pathway | 0.007746 | MAPK1, FOS, JUN, RELA |
| HLJDD-moudle 1 | 5.2 Endocrine system | hsa04915:Estrogen signaling pathway | 2.72E-04 | OPRM1, MAPK1, FOS, JUN, MMP9, MMP2 |
| HLJDD-moudle 1 | 5.2 Endocrine system | hsa04917:Prolactin signaling pathway | 7.78E-04 | MAPK1, FOS, INS, RELA, STAT3 |
| HLJDD-moudle 1 | 5.6 Nervous system | hsa04725:Cholinergic synapse | 0.004042 | MAPK1, FOS, CHRM4, CHRM2, BCL2 |
| HLJDD-moudle 1 | 5.6 Nervous system | hsa04722:Neurotrophin signaling pathway | 0.005336 | MAPK1, JUN, RELA, BCL2, TP53 |
| HLJDD-moudle 1 | 5.8 Development | hsa04380:Osteoclast differentiation | 9.83E-04 | MAPK1, FOS, TNF, JUN, RELA, IFNG |
| HLJDD-moudle 1 | 6.1 Cancers: Overview | hsa05200:Pathways in cancer | 2.63E-09 | IL6, PTGER3, PTGS2, RELA, MMP9, TP53, BCL2L1, MMP2, STAT3, MAPK1, FOS, JUN, BCL2, VEGFA, FGF2, MYC |
| HLJDD-moudle 1 | 6.1 Cancers: Overview | hsa05205:Proteoglycans in cancer | 1.87E-06 | MAPK1, TNF, MMP9, VEGFA, TP53, TLR4, FGF2, MMP2, MYC, STAT3 |
| HLJDD-moudle 1 | 6.1 Cancers: Overview | hsa05202:Transcriptional misregulation in cancer | 4.19E-04 | CSF2, IL6, RELA, MMP9, TP53, BCL2L1, MYC |
| HLJDD-moudle 1 | 6.1 Cancers: Overview | hsa05206:MicroRNAs in cancer | 0.006216 | PTGS2, BCL2, MMP9, VEGFA, TP53, MYC, STAT3 |
| HLJDD-moudle 1 | 6.1 Cancers: Overview | hsa05203:Viral carcinogenesis | 0.032366 | MAPK1, JUN, RELA, TP53, STAT3 |
| HLJDD-moudle 1 | 6.10 Infectious diseases: Parasitic | hsa05140:Leishmaniasis | 2.04E-10 | IL4, MAPK1, FOS, TNF, PTGS2, JUN, RELA, IFNG, TLR4, IL10 |
| HLJDD-moudle 1 | 6.10 Infectious diseases: Parasitic | hsa05142:Chagas disease (American trypanosomiasis) | 2.93E-10 | MAPK1, FOS, IL6, TNF, CCL2, JUN, RELA, IFNG, TLR4, IL10, IL2 |
| HLJDD-moudle 1 | 6.10 Infectious diseases: Parasitic | hsa05145:Toxoplasmosis | 3.40E-07 | MAPK1, TNF, RELA, BCL2, IFNG, TLR4, BCL2L1, IL10, STAT3 |
| HLJDD-moudle 1 | 6.10 Infectious diseases: Parasitic | hsa05144:Malaria | 3.46E-07 | ICAM1, IL6, TNF, CCL2, IFNG, TLR4, IL10 |
| HLJDD-moudle 1 | 6.10 Infectious diseases: Parasitic | hsa05146:Amoebiasis | 3.29E-05 | CSF2, IL6, TNF, RELA, IFNG, TLR4, IL10 |
| HLJDD-moudle 1 | 6.10 Infectious diseases: Parasitic | hsa05143:African trypanosomiasis | 3.86E-05 | ICAM1, IL6, TNF, IFNG, IL10 |
| HLJDD-moudle 1 | 6.2 Cancers: Specific types | hsa05219:Bladder cancer | 3.66E-06 | MAPK1, MMP9, VEGFA, TP53, MMP2, MYC |
| HLJDD-moudle 1 | 6.2 Cancers: Specific types | hsa05210:Colorectal cancer | 2.89E-05 | MAPK1, FOS, JUN, BCL2, TP53, MYC |
| HLJDD-moudle 1 | 6.2 Cancers: Specific types | hsa05212:Pancreatic cancer | 3.64E-05 | MAPK1, RELA, VEGFA, TP53, BCL2L1, STAT3 |
| HLJDD-moudle 1 | 6.2 Cancers: Specific types | hsa05222:Small cell lung cancer | 1.32E-04 | PTGS2, RELA, BCL2, TP53, BCL2L1, MYC |
| HLJDD-moudle 1 | 6.2 Cancers: Specific types | hsa05220:Chronic myeloid leukemia | 8.20E-04 | MAPK1, RELA, TP53, BCL2L1, MYC |
| HLJDD-moudle 1 | 6.2 Cancers: Specific types | hsa05215:Prostate cancer | 0.001736 | MAPK1, INS, RELA, BCL2, TP53 |
| HLJDD-moudle 1 | 6.2 Cancers: Specific types | hsa05221:Acute myeloid leukemia | 0.004322 | MAPK1, RELA, MYC, STAT3 |
| HLJDD-moudle 1 | 6.2 Cancers: Specific types | hsa05216:Thyroid cancer | 0.012603 | MAPK1, TP53, MYC |
| HLJDD-moudle 1 | 6.2 Cancers: Specific types | hsa05213:Endometrial cancer | 0.037789 | MAPK1, TP53, MYC |
| HLJDD-moudle 1 | 6.3 Immune diseases | hsa05321:Inflammatory bowel disease (IBD) | 2.00E-12 | IL4, IL6, TNF, JUN, RELA, IFNG, IL13, TLR4, IL10, STAT3, IL2 |
| HLJDD-moudle 1 | 6.3 Immune diseases | hsa05323:Rheumatoid arthritis | 1.46E-09 | CSF2, ICAM1, FOS, IL6, TNF, CCL2, JUN, VEGFA, IFNG, TLR4 |
| HLJDD-moudle 1 | 6.3 Immune diseases | hsa05330:Allograft rejection | 6.12E-05 | IL4, TNF, IFNG, IL10, IL2 |
| HLJDD-moudle 1 | 6.3 Immune diseases | hsa05310:Asthma | 7.04E-04 | IL4, TNF, IL13, IL10 |
| HLJDD-moudle 1 | 6.3 Immune diseases | hsa05332:Graft-versus-host disease | 9.35E-04 | IL6, TNF, IFNG, IL2 |
| HLJDD-moudle 1 | 6.3 Immune diseases | hsa05320:Autoimmune thyroid disease | 0.037789 | IL4, IL10, IL2 |
| HLJDD-moudle 1 | 6.4 Neurodegenerative diseases | hsa05014:Amyotrophic lateral sclerosis (ALS) | 0.003132 | TNF, BCL2, TP53, BCL2L1 |
| HLJDD-moudle 1 | 6.7 Endocrine and metabolic diseases | hsa04940:Type I diabetes mellitus | 0.001896 | TNF, INS, IFNG, IL2 |
| HLJDD-moudle 1 | 6.7 Endocrine and metabolic diseases | hsa04931:Insulin resistance | 0.003664 | IL6, TNF, INS, RELA, STAT3 |
| HLJDD-moudle 1 | 6.7 Endocrine and metabolic diseases | hsa04932:Non-alcoholic fatty liver disease (NAFLD) | 0.011849 | IL6, TNF, INS, JUN, RELA |
| HLJDD-moudle 1 | 6.7 Endocrine and metabolic diseases | hsa04930:Type II diabetes mellitus | 0.032626 | MAPK1, TNF, INS |
| HLJDD-moudle 1 | 6.8 Infectious diseases: Bacterial | hsa05132:Salmonella infection | 2.13E-08 | CSF2, MAPK1, FOS, IL6, JUN, RELA, CXCL2, IFNG, TLR4 |
| HLJDD-moudle 1 | 6.8 Infectious diseases: Bacterial | hsa05133:Pertussis | 2.23E-07 | MAPK1, FOS, IL6, TNF, JUN, RELA, TLR4, IL10 |
| HLJDD-moudle 1 | 6.8 Infectious diseases: Bacterial | hsa05152:Tuberculosis | 6.92E-05 | MAPK1, IL6, TNF, RELA, BCL2, IFNG, TLR4, IL10 |
| HLJDD-moudle 1 | 6.8 Infectious diseases: Bacterial | hsa05134:Legionellosis | 2.72E-04 | IL6, TNF, RELA, CXCL2, TLR4 |
| HLJDD-moudle 1 | 6.8 Infectious diseases: Bacterial | hsa05150:Staphylococcus aureus infection | 0.04048 | ICAM1, C5AR1, IL10 |
| HLJDD-moudle 1 | 6.9 Infectious diseases: Viral | hsa05161:Hepatitis B | 4.37E-10 | MAPK1, FOS, IL6, TNF, JUN, RELA, BCL2, MMP9, TP53, TLR4, MYC, STAT3 |
| HLJDD-moudle 1 | 6.9 Infectious diseases: Viral | hsa05162:Measles | 5.75E-08 | IL4, IL6, IL2RA, RELA, IFNG, TP53, IL13, TLR4, STAT3, IL2 |
| HLJDD-moudle 1 | 6.9 Infectious diseases: Viral | hsa05166:HTLV-I infection | 1.72E-07 | CSF2, ICAM1, FOS, IL6, TNF, IL2RA, JUN, RELA, TP53, BCL2L1, MYC, IL2 |
| HLJDD-moudle 1 | 6.9 Infectious diseases: Viral | hsa05164:Influenza A | 5.80E-07 | ICAM1, MAPK1, IL6, TNF, CCL2, JUN, RELA, IFNG, TLR4, CXCL10 |
| HLJDD-moudle 1 | 6.9 Infectious diseases: Viral | hsa05169:Epstein-Barr virus infection | 1.24E-05 | ICAM1, JUN, RELA, BCL2, IFNG, TP53, MYC, IL10, STAT3 |
| HLJDD-moudle 1 | 6.9 Infectious diseases: Viral | hsa05168:Herpes simplex infection | 8.55E-05 | FOS, IL6, TNF, CCL2, JUN, RELA, IFNG, TP53 |
| HLJDD-moudle 1 | 6.9 Infectious diseases: Viral | hsa05160:Hepatitis C | 0.007656 | MAPK1, TNF, RELA, TP53, STAT3 |
| HLJDD-moudle 1 | 5.2 Endocrine system | hsa04923:Regulation of lipolysis in adipocytes | 0.043243 | PTGER3, PTGS2, INS |
| HLJDD-moudle 2 | 3.2 Signal transduction | hsa04668:TNF signaling pathway | 1.04E-08 | VCAM1, AKT1, CASP3, MAPK14, CREB1, IL1B, MAPK8, SELE, ATF2 |
| HLJDD-moudle 2 | 3.2 Signal transduction | hsa04010:MAPK signaling pathway | 7.99E-07 | AKT1, EGFR, CASP3, MAPK14, IL1B, MAPK8, EGF, TGFB1, IL1A, ATF2 |
| HLJDD-moudle 2 | 3.2 Signal transduction | hsa04066:HIF-1 signaling pathway | 3.15E-06 | AKT1, EGFR, ERBB2, SERPINE1, NOS3, EGF, TIMP1 |
| HLJDD-moudle 2 | 3.2 Signal transduction | hsa04068:FoxO signaling pathway | 1.93E-05 | AKT1, EGFR, CCND1, MAPK14, MAPK8, EGF, TGFB1 |
| HLJDD-moudle 2 | 3.2 Signal transduction | hsa04012:ErbB signaling pathway | 5.00E-04 | AKT1, EGFR, ERBB2, MAPK8, EGF |
| HLJDD-moudle 2 | 3.2 Signal transduction | hsa04151:PI3K-Akt signaling pathway | 5.46E-04 | AKT1, EGFR, CCND1, CREB1, TLR2, NOS3, EGF, ATF2 |
| HLJDD-moudle 2 | 3.2 Signal transduction | hsa04071:Sphingolipid signaling pathway | 0.001672 | AKT1, MAPK14, BAX, MAPK8, NOS3 |
| HLJDD-moudle 2 | 3.2 Signal transduction | hsa04064:NF-kappa B signaling pathway | 0.006123 | VCAM1, XIAP, CD40LG, IL1B |
| HLJDD-moudle 2 | 3.2 Signal transduction | hsa04152:AMPK signaling pathway | 0.015405 | AKT1, CCND1, CREB1, PPARG |
| HLJDD-moudle 2 | 3.2 Signal transduction | hsa04390:Hippo signaling pathway | 0.026982 | CCND1, SERPINE1, BIRC5, TGFB1 |
| HLJDD-moudle 2 | 3.2 Signal transduction | hsa04370:VEGF signaling pathway | 0.028459 | AKT1, MAPK14, NOS3 |
| HLJDD-moudle 2 | 3.2 Signal transduction | hsa04022:cGMP-PKG signaling pathway | 0.034389 | AKT1, CREB1, NOS3, ATF2 |
| HLJDD-moudle 2 | 3.3 Signaling molecules and interaction | hsa04060:Cytokine-cytokine receptor interaction | 0.002821 | EGFR, CD40LG, IL1B, EGF, TGFB1, IL1A |
| HLJDD-moudle 2 | 3.3 Signaling molecules and interaction | hsa04514:Cell adhesion molecules (CAMs) | 0.003092 | VCAM1, CD86, CD80, CD40LG, SELE |
| HLJDD-moudle 2 | 4.2 Cell growth and death | hsa04210:Apoptosis | 1.35E-04 | AKT1, CASP3, XIAP, BAX, CYCS |
| HLJDD-moudle 2 | 4.2 Cell growth and death | hsa04115:p53 signaling pathway | 1.83E-04 | CASP3, CCND1, BAX, CYCS, SERPINE1 |
| HLJDD-moudle 2 | 4.3 Cellular community - eukaryotes | hsa04510:Focal adhesion | 2.13E-04 | AKT1, EGFR, CCND1, XIAP, ERBB2, MAPK8, EGF |
| HLJDD-moudle 2 | 5.1 Immune system | hsa04620:Toll-like receptor signaling pathway | 2.49E-07 | AKT1, CD86, CD80, MAPK14, TLR2, IL1B, MAPK8, STAT1 |
| HLJDD-moudle 2 | 5.1 Immune system | hsa04672:Intestinal immune network for IgA production | 0.001053 | CD86, CD80, CD40LG, TGFB1 |
| HLJDD-moudle 2 | 5.1 Immune system | hsa04621:NOD-like receptor signaling pathway | 0.023467 | MAPK14, IL1B, MAPK8 |
| HLJDD-moudle 2 | 5.1 Immune system | hsa04664:Fc epsilon RI signaling pathway | 0.034771 | AKT1, MAPK14, MAPK8 |
| HLJDD-moudle 2 | 5.2 Endocrine system | hsa04917:Prolactin signaling pathway | 2.29E-04 | AKT1, CCND1, MAPK14, MAPK8, STAT1 |
| HLJDD-moudle 2 | 5.2 Endocrine system | hsa04915:Estrogen signaling pathway | 8.16E-04 | AKT1, EGFR, CREB1, NOS3, ATF2 |
| HLJDD-moudle 2 | 5.3 Circulatory system | hsa04261:Adrenergic signaling in cardiomyocytes | 0.024728 | AKT1, MAPK14, CREB1, ATF2 |
| HLJDD-moudle 2 | 5.6 Nervous system | hsa04728:Dopaminergic synapse | 0.00212 | AKT1, MAPK14, CREB1, MAPK8, ATF2 |
| HLJDD-moudle 2 | 5.6 Nervous system | hsa04722:Neurotrophin signaling pathway | 0.014739 | AKT1, MAPK14, BAX, MAPK8 |
| HLJDD-moudle 2 | 5.8 Development | hsa04380:Osteoclast differentiation | 5.55E-08 | AKT1, MAPK14, CREB1, PPARG, IL1B, MAPK8, STAT1, TGFB1, IL1A |
| HLJDD-moudle 2 | 6.1 Cancers: Overview | hsa05200:Pathways in cancer | 1.15E-11 | EGFR, XIAP, ERBB2, PPARG, CYCS, BIRC5, RB1, STAT1, TGFB1, MMP1, AKT1, CASP3, CCND1, BAX, MAPK8, EGF |
| HLJDD-moudle 2 | 6.1 Cancers: Overview | hsa05205:Proteoglycans in cancer | 1.77E-05 | AKT1, EGFR, CASP3, CCND1, MAPK14, ERBB2, TLR2, TGFB1 |
| HLJDD-moudle 2 | 6.1 Cancers: Overview | hsa05203:Viral carcinogenesis | 0.001703 | CASP3, CCND1, BAX, CREB1, RB1, ATF2 |
| HLJDD-moudle 2 | 6.1 Cancers: Overview | hsa05231:Choline metabolism in cancer | 0.009243 | AKT1, EGFR, MAPK8, EGF |
| HLJDD-moudle 2 | 6.1 Cancers: Overview | hsa05230:Central carbon metabolism in cancer | 0.031102 | AKT1, EGFR, ERBB2 |
| HLJDD-moudle 2 | 6.10 Infectious diseases: Parasitic | hsa05145:Toxoplasmosis | 9.62E-10 | AKT1, CASP3, XIAP, CD40LG, MAPK14, CYCS, TLR2, MAPK8, STAT1, TGFB1 |
| HLJDD-moudle 2 | 6.10 Infectious diseases: Parasitic | hsa05144:Malaria | 1.81E-06 | VCAM1, CD40LG, TLR2, IL1B, SELE, TGFB1 |
| HLJDD-moudle 2 | 6.10 Infectious diseases: Parasitic | hsa05142:Chagas disease (American trypanosomiasis) | 4.45E-06 | AKT1, MAPK14, SERPINE1, TLR2, IL1B, MAPK8, TGFB1 |
| HLJDD-moudle 2 | 6.10 Infectious diseases: Parasitic | hsa05140:Leishmaniasis | 1.16E-05 | MAPK14, TLR2, IL1B, STAT1, TGFB1, IL1A |
| HLJDD-moudle 2 | 6.10 Infectious diseases: Parasitic | hsa05143:African trypanosomiasis | 0.00885 | VCAM1, IL1B, SELE |
| HLJDD-moudle 2 | 6.10 Infectious diseases: Parasitic | hsa05146:Amoebiasis | 0.010545 | CASP3, TLR2, IL1B, TGFB1 |
| HLJDD-moudle 2 | 6.2 Cancers: Specific types | hsa05212:Pancreatic cancer | 1.96E-10 | AKT1, EGFR, CCND1, ERBB2, MAPK8, RB1, EGF, STAT1, TGFB1 |
| HLJDD-moudle 2 | 6.2 Cancers: Specific types | hsa05210:Colorectal cancer | 5.73E-09 | AKT1, CASP3, CCND1, BAX, CYCS, BIRC5, MAPK8, TGFB1 |
| HLJDD-moudle 2 | 6.2 Cancers: Specific types | hsa05219:Bladder cancer | 7.31E-07 | EGFR, CCND1, ERBB2, RB1, EGF, MMP1 |
| HLJDD-moudle 2 | 6.2 Cancers: Specific types | hsa05215:Prostate cancer | 1.67E-06 | AKT1, EGFR, CCND1, CREB1, ERBB2, RB1, EGF |
| HLJDD-moudle 2 | 6.2 Cancers: Specific types | hsa05223:Non-small cell lung cancer | 3.56E-06 | AKT1, EGFR, CCND1, ERBB2, RB1, EGF |
| HLJDD-moudle 2 | 6.2 Cancers: Specific types | hsa05213:Endometrial cancer | 6.76E-05 | AKT1, EGFR, CCND1, ERBB2, EGF |
| HLJDD-moudle 2 | 6.2 Cancers: Specific types | hsa05214:Glioma | 1.63E-04 | AKT1, EGFR, CCND1, RB1, EGF |
| HLJDD-moudle 2 | 6.2 Cancers: Specific types | hsa05218:Melanoma | 2.29E-04 | AKT1, EGFR, CCND1, RB1, EGF |
| HLJDD-moudle 2 | 6.2 Cancers: Specific types | hsa05222:Small cell lung cancer | 4.58E-04 | AKT1, CCND1, XIAP, CYCS, RB1 |
| HLJDD-moudle 2 | 6.2 Cancers: Specific types | hsa05220:Chronic myeloid leukemia | 0.003599 | AKT1, CCND1, RB1, TGFB1 |
| HLJDD-moudle 2 | 6.3 Immune diseases | hsa05323:Rheumatoid arthritis | 1.67E-06 | CD86, CD80, TLR2, IL1B, TGFB1, IL1A, MMP1 |
| HLJDD-moudle 2 | 6.3 Immune diseases | hsa05321:Inflammatory bowel disease (IBD) | 1.53E-04 | TLR2, IL1B, STAT1, TGFB1, IL1A |
| HLJDD-moudle 2 | 6.3 Immune diseases | hsa05332:Graft-versus-host disease | 3.69E-04 | CD86, CD80, IL1B, IL1A |
| HLJDD-moudle 2 | 6.3 Immune diseases | hsa05330:Allograft rejection | 0.011044 | CD86, CD80, CD40LG |
| HLJDD-moudle 2 | 6.3 Immune diseases | hsa05320:Autoimmune thyroid disease | 0.021123 | CD86, CD80, CD40LG |
| HLJDD-moudle 2 | 6.4 Neurodegenerative diseases | hsa05014:Amyotrophic lateral sclerosis (ALS) | 0.001261 | CASP3, MAPK14, BAX, CYCS |
| HLJDD-moudle 2 | 6.4 Neurodegenerative diseases | hsa05020:Prion diseases | 0.00885 | BAX, IL1B, IL1A |
| HLJDD-moudle 2 | 6.4 Neurodegenerative diseases | hsa05016:Huntington's disease | 0.008992 | CASP3, BAX, CREB1, CYCS, PPARG |
| HLJDD-moudle 2 | 6.6 Cardiovascular diseases | hsa05416:Viral myocarditis | 3.89E-06 | CASP3, CD86, CCND1, CD80, CD40LG, CYCS |
| HLJDD-moudle 2 | 6.7 Endocrine and metabolic diseases | hsa04932:Non-alcoholic fatty liver disease (NAFLD) | 2.76E-06 | AKT1, CASP3, BAX, CYCS, IL1B, MAPK8, TGFB1, IL1A |
| HLJDD-moudle 2 | 6.7 Endocrine and metabolic diseases | hsa04940:Type I diabetes mellitus | 7.56E-04 | CD86, CD80, IL1B, IL1A |
| HLJDD-moudle 2 | 6.7 Endocrine and metabolic diseases | hsa04931:Insulin resistance | 0.011095 | AKT1, CREB1, MAPK8, NOS3 |
| HLJDD-moudle 2 | 6.8 Infectious diseases: Bacterial | hsa05152:Tuberculosis | 8.18E-11 | AKT1, CASP3, MAPK14, BAX, CREB1, CYCS, TLR2, IL1B, MAPK8, STAT1, TGFB1, IL1A |
| HLJDD-moudle 2 | 6.8 Infectious diseases: Bacterial | hsa05133:Pertussis | 2.83E-04 | CASP3, MAPK14, IL1B, MAPK8, IL1A |
| HLJDD-moudle 2 | 6.8 Infectious diseases: Bacterial | hsa05134:Legionellosis | 0.001578 | CASP3, CYCS, TLR2, IL1B |
| HLJDD-moudle 2 | 6.8 Infectious diseases: Bacterial | hsa05120:Epithelial cell signaling in Helicobacter pylori infection | 0.002934 | EGFR, CASP3, MAPK14, MAPK8 |
| HLJDD-moudle 2 | 6.8 Infectious diseases: Bacterial | hsa05132:Salmonella infection | 0.00537 | MAPK14, IL1B, MAPK8, IL1A |
| HLJDD-moudle 2 | 6.9 Infectious diseases: Viral | hsa05161:Hepatitis B | 2.89E-13 | AKT1, CASP3, CCND1, BAX, CREB1, CYCS, TLR2, BIRC5, MAPK8, RB1, STAT1, TGFB1, ATF2 |
| HLJDD-moudle 2 | 6.9 Infectious diseases: Viral | hsa05164:Influenza A | 7.10E-06 | AKT1, MAPK14, CYCS, IL1B, MAPK8, STAT1, IL1A, ATF2 |
| HLJDD-moudle 2 | 6.9 Infectious diseases: Viral | hsa05166:HTLV-I infection | 9.16E-06 | VCAM1, AKT1, CCND1, XIAP, BAX, CREB1, RB1, TGFB1, ATF2 |
| HLJDD-moudle 2 | 6.9 Infectious diseases: Viral | hsa05160:Hepatitis C | 2.37E-04 | AKT1, EGFR, MAPK14, MAPK8, EGF, STAT1 |
| HLJDD-moudle 2 | 6.9 Infectious diseases: Viral | hsa05162:Measles | 2.37E-04 | AKT1, CCND1, TLR2, IL1B, STAT1, IL1A |
| HLJDD-moudle 2 | 6.9 Infectious diseases: Viral | hsa05168:Herpes simplex infection | 0.001026 | CASP3, CYCS, TLR2, IL1B, MAPK8, STAT1 |
| HLJDD-moudle 2 | 6.9 Infectious diseases: Viral | hsa05169:Epstein-Barr virus infection | 0.008673 | AKT1, MAPK14, MAPK8, RB1, ATF2 |
| HLJDD-moudle 3 | 1.3 Lipid metabolism | hsa00590:Arachidonic acid metabolism | 5.44E-12 | PLB1, CYP2B6, CYP2C9, PTGS1, PLA2G1B, ALOX5, PLA2G2E, ALOX12 |
| HLJDD-moudle 3 | 1.11 Xenobiotics biodegradation and metabolism | hsa00980:Metabolism of xenobiotics by cytochrome P450 | 1.97E-11 | GSTM1, CYP3A4, GSTM2, CYP1A1, CYP2B6, CYP2C9, CYP1A2, GSTP1 |
| HLJDD-moudle 3 | 1.3 Lipid metabolism | hsa00591:Linoleic acid metabolism | 1.14E-09 | CYP3A4, PLB1, CYP2C9, PLA2G1B, CYP1A2, PLA2G2E |
| HLJDD-moudle 3 | 1.11 Xenobiotics biodegradation and metabolism | hsa00982:Drug metabolism - cytochrome P450 | 1.18E-09 | GSTM1, CYP3A4, GSTM2, CYP2B6, CYP2C9, CYP1A2, GSTP1 |
| HLJDD-moudle 3 | 6.1 Cancers: Overview | hsa05204:Chemical carcinogenesis | 3.20E-09 | GSTM1, CYP3A4, GSTM2, CYP1A1, CYP2C9, CYP1A2, GSTP1 |
| HLJDD-moudle 3 | 1.8 Metabolism of cofactors and vitamins | hsa00830:Retinol metabolism | 4.79E-06 | CYP3A4, CYP1A1, CYP2B6, CYP2C9, CYP1A2 |
| HLJDD-moudle 3 | 1.0 Global and overview maps | hsa01100:Metabolic pathways | 5.16E-06 | CYP3A4, PLB1, CYP1A1, CYP2B6, CYP2C9, PTGS1, PLA2G1B, ALOX5, CYP1A2, PLA2G2E, ALOX12 |
| HLJDD-moudle 3 | 1.3 Lipid metabolism | hsa00592:alpha-Linolenic acid metabolism | 9.57E-04 | PLB1, PLA2G1B, PLA2G2E |
| HLJDD-moudle 3 | 5.6 Nervous system | hsa04726:Serotonergic synapse | 0.001026 | CYP2C9, PTGS1, ALOX5, ALOX12 |
| HLJDD-moudle 3 | 1.3 Lipid metabolism | hsa00565:Ether lipid metabolism | 0.003091 | PLB1, PLA2G1B, PLA2G2E |
| HLJDD-moudle 3 | 1.6 Metabolism of other amino acids | hsa00480:Glutathione metabolism | 0.003955 | GSTM1, GSTM2, GSTP1 |
| HLJDD-moudle 3 | 1.3 Lipid metabolism | hsa00140:Steroid hormone biosynthesis | 0.00509 | CYP3A4, CYP1A1, CYP1A2 |
| HLJDD-moudle 3 | 1.3 Lipid metabolism | hsa00564:Glycerophospholipid metabolism | 0.013219 | PLB1, PLA2G1B, PLA2G2E |
| HLJDD-moudle 4 | 3.2 Signal transduction | hsa04020:Calcium signaling pathway | 1.38E-06 | CHRM5, CHRM3, CHRM1, ADRA1B, TBXA2R, NOS2, HTR2C, ADRA1D, HTR2A |
| HLJDD-moudle 4 | 3.2 Signal transduction | hsa04068:FoxO signaling pathway | 0.003577 | CCNB1, CDKN1A, EP300, CCND2, CDK2 |
| HLJDD-moudle 4 | 3.2 Signal transduction | hsa04310:Wnt signaling pathway | 0.003976 | EP300, CCND2, RHOA, FOSL1, NFATC1 |
| HLJDD-moudle 4 | 3.2 Signal transduction | hsa04151:PI3K-Akt signaling pathway | 0.005223 | CDKN1A, CASP9, CCND2, CHRM1, CDK6, CDK4, CDK2 |
| HLJDD-moudle 4 | 3.2 Signal transduction | hsa04024:cAMP signaling pathway | 0.013968 | EP300, CHRM1, RHOA, NFKBIA, NFATC1 |
| HLJDD-moudle 4 | 3.2 Signal transduction | hsa04022:cGMP-PKG signaling pathway | 0.04388 | ADRA1B, RHOA, ADRA1D, NFATC1 |
| HLJDD-moudle 4 | 3.3 Signaling molecules and interaction | hsa04080:Neuroactive ligand-receptor interaction | 2.68E-04 | CHRM5, CHRM3, CHRM1, ADRA1B, TBXA2R, HTR2C, ADRA1D, HTR2A |
| HLJDD-moudle 4 | 4.2 Cell growth and death | hsa04110:Cell cycle | 5.50E-12 | CCNB1, E2F1, CDK1, E2F2, CDKN1A, EP300, CCND2, CDK6, CDK7, CDK4, CCNA2, CDK2 |
| HLJDD-moudle 4 | 4.2 Cell growth and death | hsa04115:p53 signaling pathway | 5.83E-10 | CCNB1, CDK1, CDKN1A, CASP9, CCND2, CASP8, CDK6, CDK4, CDK2 |
| HLJDD-moudle 4 | 4.2 Cell growth and death | hsa04114:Oocyte meiosis | 0.014775 | PGR, CDK1, AR, CDK2 |
| HLJDD-moudle 4 | 4.2 Cell growth and death | hsa04210:Apoptosis | 0.034996 | CASP9, CASP8, NFKBIA |
| HLJDD-moudle 4 | 5.1 Immune system | hsa04660:T cell receptor signaling pathway | 0.01269 | RHOA, NFKBIA, CDK4, NFATC1 |
| HLJDD-moudle 4 | 5.2 Endocrine system | hsa04914:Progesterone-mediated oocyte maturation | 7.26E-04 | CCNB1, PGR, CDK1, CCNA2, CDK2 |
| HLJDD-moudle 4 | 6.1 Cancers: Overview | hsa05203:Viral carcinogenesis | 1.31E-09 | CDK1, BAK1, CDKN1A, EP300, CCND2, CASP8, RHOA, NFKBIA, CDK6, CDK4, CCNA2, CDK2 |
| HLJDD-moudle 4 | 6.1 Cancers: Overview | hsa05200:Pathways in cancer | 1.16E-07 | E2F1, E2F2, AR, CDKN1A, EP300, CASP9, CASP8, RHOA, NFKBIA, CDK6, NOS2, CDK4, CDK2 |
| HLJDD-moudle 4 | 6.1 Cancers: Overview | hsa05206:MicroRNAs in cancer | 3.20E-04 | E2F1, E2F2, BAK1, CDKN1A, EP300, CCND2, RHOA, CDK6 |
| HLJDD-moudle 4 | 6.10 Infectious diseases: Parasitic | hsa05145:Toxoplasmosis | 0.018249 | CASP9, CASP8, NFKBIA, NOS2 |
| HLJDD-moudle 4 | 6.2 Cancers: Specific types | hsa05222:Small cell lung cancer | 1.09E-07 | E2F1, E2F2, CASP9, NFKBIA, CDK6, NOS2, CDK4, CDK2 |
| HLJDD-moudle 4 | 6.2 Cancers: Specific types | hsa05215:Prostate cancer | 1.39E-07 | E2F1, E2F2, AR, CDKN1A, EP300, CASP9, NFKBIA, CDK2 |
| HLJDD-moudle 4 | 6.2 Cancers: Specific types | hsa05220:Chronic myeloid leukemia | 2.02E-05 | E2F1, E2F2, CDKN1A, NFKBIA, CDK6, CDK4 |
| HLJDD-moudle 4 | 6.2 Cancers: Specific types | hsa05223:Non-small cell lung cancer | 1.33E-04 | E2F1, E2F2, CASP9, CDK6, CDK4 |
| HLJDD-moudle 4 | 6.2 Cancers: Specific types | hsa05214:Glioma | 2.38E-04 | E2F1, E2F2, CDKN1A, CDK6, CDK4 |
| HLJDD-moudle 4 | 6.2 Cancers: Specific types | hsa05212:Pancreatic cancer | 2.38E-04 | E2F1, E2F2, CASP9, CDK6, CDK4 |
| HLJDD-moudle 4 | 6.2 Cancers: Specific types | hsa05218:Melanoma | 3.34E-04 | E2F1, E2F2, CDKN1A, CDK6, CDK4 |
| HLJDD-moudle 4 | 6.2 Cancers: Specific types | hsa05219:Bladder cancer | 9.35E-04 | E2F1, E2F2, CDKN1A, CDK4 |
| HLJDD-moudle 4 | 6.6 Cardiovascular diseases | hsa05416:Viral myocarditis | 0.029975 | CAV1, CASP9, CASP8 |
| HLJDD-moudle 4 | 6.8 Infectious diseases: Bacterial | hsa05152:Tuberculosis | 0.009534 | EP300, CASP9, CASP8, RHOA, NOS2 |
| HLJDD-moudle 4 | 6.8 Infectious diseases: Bacterial | hsa05134:Legionellosis | 0.027116 | CASP9, CASP8, NFKBIA |
| HLJDD-moudle 4 | 6.9 Infectious diseases: Viral | hsa05161:Hepatitis B | 3.09E-11 | E2F1, E2F2, CDKN1A, EP300, CASP9, CASP8, NFKBIA, CDK6, CDK4, CCNA2, CDK2, NFATC1 |
| HLJDD-moudle 4 | 6.9 Infectious diseases: Viral | hsa05166:HTLV-I infection | 1.97E-05 | E2F1, E2F2, CDKN1A, EP300, CCND2, NFKBIA, CDK4, FOSL1, NFATC1 |
| HLJDD-moudle 4 | 6.9 Infectious diseases: Viral | hsa05169:Epstein-Barr virus infection | 0.001892 | CDK1, CDKN1A, EP300, NFKBIA, CCNA2, CDK2 |
| HLJDD-moudle 4 | 6.9 Infectious diseases: Viral | hsa05162:Measles | 0.003482 | CCND2, NFKBIA, CDK6, CDK4, CDK2 |
| HLJDD-moudle 4 | 6.9 Infectious diseases: Viral | hsa05168:Herpes simplex infection | 0.01069 | CDK1, EP300, CASP8, NFKBIA, CDK2 |
| HLJDD-moudle 5 | 5.2 Endocrine system | hsa03320:PPAR signaling pathway | 8.59E-06 | PPARD, RXRB, RXRA, FABP1 |
| HLJDD-moudle 5 | 5.2 Endocrine system | hsa04920:Adipocytokine signaling pathway | 9.92E-04 | SLC2A4, RXRB, RXRA |
| HLJDD-moudle 5 | 5.2 Endocrine system | hsa04919:Thyroid hormone signaling pathway | 0.002612 | NCOA2, RXRB, RXRA |
| HLJDD-moudle 5 | 6.2 Cancers: Specific types | hsa05216:Thyroid cancer | 0.020815 | RXRB, RXRA |
| HLJDD-moudle 5 | 6.1 Cancers: Overview | hsa05200:Pathways in cancer | 0.028768 | PPARD, RXRB, RXRA |
| HLJDD-moudle 5 | 6.2 Cancers: Specific types | hsa05223:Non-small cell lung cancer | 0.039881 | RXRB, RXRA |
| HLJDD-moudle 6 | 3.2 Signal transduction | hsa04020:Calcium signaling pathway | 1.71E-05 | DRD1, ADRB2, ADRB1, DRD5 |
| HLJDD-moudle 6 | 3.2 Signal transduction | hsa04024:cAMP signaling pathway | 2.32E-05 | DRD1, ADRB2, ADRB1, DRD5 |
| HLJDD-moudle 6 | 3.3 Signaling molecules and interaction | hsa04080:Neuroactive ligand-receptor interaction | 6.37E-05 | DRD1, ADRB2, ADRB1, DRD5 |
| HLJDD-moudle 6 | 5.2 Endocrine system | hsa04923:Regulation of lipolysis in adipocytes | 0.02412 | ADRB2, ADRB1 |
| HLJDD-moudle 6 | 5.2 Endocrine system | hsa04924:Renin secretion | 0.027533 | ADRB2, ADRB1 |
| HLJDD-moudle 6 | 5.4 Digestive system | hsa04970:Salivary secretion | 0.03688 | ADRB2, ADRB1 |
| HLJDD-moudle 6 | 4.3 Cellular community - eukaryotes | hsa04540:Gap junction | 0.037726 | DRD1, ADRB1 |
| HLJDD-moudle 7 | 3.2 Signal transduction | hsa04014:Ras signaling pathway | 8.98E-07 | PRKCA, ELK1, PRKCG, INSR, RASA1, CALM2, PRKCB |
| HLJDD-moudle 7 | 3.2 Signal transduction | hsa04015:Rap1 signaling pathway | 3.39E-04 | PRKCA, PRKCG, INSR, CALM2, PRKCB |
| HLJDD-moudle 7 | 3.2 Signal transduction | hsa04012:ErbB signaling pathway | 3.91E-04 | PRKCA, ELK1, PRKCG, PRKCB |
| HLJDD-moudle 7 | 3.2 Signal transduction | hsa04070:Phosphatidylinositol signaling system | 5.55E-04 | PRKCA, PRKCG, CALM2, PRKCB |
| HLJDD-moudle 7 | 3.2 Signal transduction | hsa04066:HIF-1 signaling pathway | 5.55E-04 | PRKCA, PRKCG, INSR, PRKCB |
| HLJDD-moudle 7 | 3.2 Signal transduction | hsa04010:MAPK signaling pathway | 7.09E-04 | PRKCA, ELK1, PRKCG, RASA1, PRKCB |
| HLJDD-moudle 7 | 3.2 Signal transduction | hsa04310:Wnt signaling pathway | 0.001502 | PRKCA, PRKCG, PRKCB, APC |
| HLJDD-moudle 7 | 3.2 Signal transduction | hsa04020:Calcium signaling pathway | 0.003165 | PRKCA, PRKCG, CALM2, PRKCB |
| HLJDD-moudle 7 | 3.2 Signal transduction | hsa04150:mTOR signaling pathway | 0.00433 | PRKCA, PRKCG, PRKCB |
| HLJDD-moudle 7 | 3.2 Signal transduction | hsa04370:VEGF signaling pathway | 0.00478 | PRKCA, PRKCG, PRKCB |
| HLJDD-moudle 7 | 3.2 Signal transduction | hsa04071:Sphingolipid signaling pathway | 0.017617 | PRKCA, PRKCG, PRKCB |
| HLJDD-moudle 7 | 4.3 Cellular community - eukaryotes | hsa04510:Focal adhesion | 0.004708 | PRKCA, ELK1, PRKCG, PRKCB |
| HLJDD-moudle 7 | 4.3 Cellular community - eukaryotes | hsa04540:Gap junction | 0.009741 | PRKCA, PRKCG, PRKCB |
| HLJDD-moudle 7 | 4.3 Cellular community - eukaryotes | hsa04530:Tight junction | 0.022613 | PRKCA, PRKCG, PRKCB |
| HLJDD-moudle 7 | 5.1 Immune system | hsa04666:Fc gamma R-mediated phagocytosis | 0.008905 | PRKCA, PRKCG, PRKCB |
| HLJDD-moudle 7 | 5.1 Immune system | hsa04670:Leukocyte transendothelial migration | 0.017065 | PRKCA, PRKCG, PRKCB |
| HLJDD-moudle 7 | 5.1 Immune system | hsa04650:Natural killer cell mediated cytotoxicity | 0.018177 | PRKCA, PRKCG, PRKCB |
| HLJDD-moudle 7 | 5.10 Environmental adaptation | hsa04713:Circadian entrainment | 5.06E-04 | PRKCA, PRKCG, CALM2, PRKCB |
| HLJDD-moudle 7 | 5.2 Endocrine system | hsa04921:Oxytocin signaling pathway | 1.13E-04 | PRKCA, ELK1, PRKCG, CALM2, PRKCB |
| HLJDD-moudle 7 | 5.2 Endocrine system | hsa04925:Aldosterone synthesis and secretion | 3.16E-04 | PRKCA, PRKCG, CALM2, PRKCB |
| HLJDD-moudle 7 | 5.2 Endocrine system | hsa04912:GnRH signaling pathway | 4.46E-04 | PRKCA, ELK1, CALM2, PRKCB |
| HLJDD-moudle 7 | 5.2 Endocrine system | hsa04916:Melanogenesis | 5.89E-04 | PRKCA, PRKCG, CALM2, PRKCB |
| HLJDD-moudle 7 | 5.2 Endocrine system | hsa04918:Thyroid hormone synthesis | 0.006253 | PRKCA, PRKCG, PRKCB |
| HLJDD-moudle 7 | 5.2 Endocrine system | hsa04911:Insulin secretion | 0.009111 | PRKCA, PRKCG, PRKCB |
| HLJDD-moudle 7 | 5.2 Endocrine system | hsa04919:Thyroid hormone signaling pathway | 0.015985 | PRKCA, PRKCG, PRKCB |
| HLJDD-moudle 7 | 5.2 Endocrine system | hsa04910:Insulin signaling pathway | 0.022923 | ELK1, INSR, CALM2 |
| HLJDD-moudle 7 | 5.3 Circulatory system | hsa04270:Vascular smooth muscle contraction | 9.78E-04 | PRKCA, PRKCG, CALM2, PRKCB |
| HLJDD-moudle 7 | 5.4 Digestive system | hsa04971:Gastric acid secretion | 2.32E-04 | PRKCA, PRKCG, CALM2, PRKCB |
| HLJDD-moudle 7 | 5.4 Digestive system | hsa04970:Salivary secretion | 3.78E-04 | PRKCA, PRKCG, CALM2, PRKCB |
| HLJDD-moudle 7 | 5.4 Digestive system | hsa04972:Pancreatic secretion | 0.010834 | PRKCA, PRKCG, PRKCB |
| HLJDD-moudle 7 | 5.5 Excretory system | hsa04960:Aldosterone-regulated sodium reabsorption | 3.53E-05 | PRKCA, PRKCG, INSR, PRKCB |
| HLJDD-moudle 7 | 5.5 Excretory system | hsa04961:Endocrine and other factor-regulated calcium reabsorption | 0.002626 | PRKCA, PRKCG, PRKCB |
| HLJDD-moudle 7 | 5.6 Nervous system | hsa04720:Long-term potentiation | 1.72E-04 | PRKCA, PRKCG, CALM2, PRKCB |
| HLJDD-moudle 7 | 5.6 Nervous system | hsa04728:Dopaminergic synapse | 0.001209 | PRKCA, PRKCG, CALM2, PRKCB |
| HLJDD-moudle 7 | 5.6 Nervous system | hsa04730:Long-term depression | 0.004627 | PRKCA, PRKCG, PRKCB |
| HLJDD-moudle 7 | 5.6 Nervous system | hsa04727:GABAergic synapse | 0.009111 | PRKCA, PRKCG, PRKCB |
| HLJDD-moudle 7 | 5.6 Nervous system | hsa04723:Retrograde endocannabinoid signaling | 0.012691 | PRKCA, PRKCG, PRKCB |
| HLJDD-moudle 7 | 5.6 Nervous system | hsa04726:Serotonergic synapse | 0.015195 | PRKCA, PRKCG, PRKCB |
| HLJDD-moudle 7 | 5.6 Nervous system | hsa04725:Cholinergic synapse | 0.015195 | PRKCA, PRKCG, PRKCB |
| HLJDD-moudle 7 | 5.6 Nervous system | hsa04724:Glutamatergic synapse | 0.015985 | PRKCA, PRKCG, PRKCB |
| HLJDD-moudle 7 | 5.7 Sensory system | hsa04750:Inflammatory mediator regulation of TRP channels | 5.55E-04 | PRKCA, PRKCG, CALM2, PRKCB |
| HLJDD-moudle 7 | 6.1 Cancers: Overview | hsa05205:Proteoglycans in cancer | 0.004332 | PRKCA, ELK1, PRKCG, PRKCB |
| HLJDD-moudle 7 | 6.1 Cancers: Overview | hsa05206:MicroRNAs in cancer | 0.011576 | PRKCA, PRKCG, PRKCB, APC |
| HLJDD-moudle 7 | 6.1 Cancers: Overview | hsa05231:Choline metabolism in cancer | 0.012691 | PRKCA, PRKCG, PRKCB |
| HLJDD-moudle 7 | 6.1 Cancers: Overview | hsa05200:Pathways in cancer | 0.027347 | PRKCA, PRKCG, PRKCB, APC |
| HLJDD-moudle 7 | 6.10 Infectious diseases: Parasitic | hsa05143:African trypanosomiasis | 0.001417 | PRKCA, PRKCG, PRKCB |
| HLJDD-moudle 7 | 6.10 Infectious diseases: Parasitic | hsa05146:Amoebiasis | 0.013918 | PRKCA, PRKCG, PRKCB |
| HLJDD-moudle 7 | 6.2 Cancers: Specific types | hsa05214:Glioma | 1.65E-04 | PRKCA, PRKCG, CALM2, PRKCB |
| HLJDD-moudle 7 | 6.2 Cancers: Specific types | hsa05223:Non-small cell lung cancer | 0.004042 | PRKCA, PRKCG, PRKCB |
| HLJDD-moudle 7 | 6.5 Substance dependence | hsa05031:Amphetamine addiction | 1.72E-04 | PRKCA, PRKCG, CALM2, PRKCB |
| HLJDD-moudle 7 | 6.5 Substance dependence | hsa05032:Morphine addiction | 0.010391 | PRKCA, PRKCG, PRKCB |
| HLJDD-moudle 7 | 6.8 Infectious diseases: Bacterial | hsa05110:Vibrio cholerae infection | 0.003627 | PRKCA, PRKCG, PRKCB |
| HLJDD-moudle 7 | 6.9 Infectious diseases: Viral | hsa05161:Hepatitis B | 0.001733 | PRKCA, ELK1, PRKCG, PRKCB |
| HLJDD-moudle 9 | 6.1 Cancers: Overview | hsa05205:Proteoglycans in cancer | 8.34E-04 | HIF1A, MDM2, KDR |
| HLJDD-moudle 9 | 5.2 Endocrine system | hsa04919:Thyroid hormone signaling pathway | 0.032726 | HIF1A, MDM2 |
| HLJDD-moudle 11 | 1.3 Lipid metabolism | hsa00140:Steroid hormone biosynthesis | 6.92E-05 | AKR1C3, SULT1E1, CYP19A1 |
| HLJDD-moudle 11 | 5.2 Endocrine system | hsa04913:Ovarian steroidogenesis | 0.014133 | AKR1C3, CYP19A1 |
| HLJDD-moudle 12 | 4.5 Cell motility | hsa04810:Regulation of actin cytoskeleton | 9.28E-04 | ITGA6, ITGB2, ITGB3 |
| HLJDD-moudle 12 | 6.6 Cardiovascular diseases | hsa05412:Arrhythmogenic right ventricular cardiomyopathy (ARVC) | 0.020446 | ITGA6, ITGB3 |
| HLJDD-moudle 12 | 6.6 Cardiovascular diseases | hsa05410:Hypertrophic cardiomyopathy (HCM) | 0.02245 | ITGA6, ITGB3 |
| HLJDD-moudle 12 | 6.6 Cardiovascular diseases | hsa05414:Dilated cardiomyopathy | 0.024167 | ITGA6, ITGB3 |
| HLJDD-moudle 12 | 5.1 Immune system | hsa04640:Hematopoietic cell lineage | 0.024452 | ITGA6, ITGB3 |
| HLJDD-moudle 12 | 3.3 Signaling molecules and interaction | hsa04512:ECM-receptor interaction | 0.025024 | ITGA6, ITGB3 |
| HLJDD-moudle 12 | 3.3 Signaling molecules and interaction | hsa04514:Cell adhesion molecules (CAMs) | 0.04068 | ITGA6, ITGB2 |
| HLJDD-moudle 12 | 4.1 Transport and catabolism | hsa04145:Phagosome | 0.043797 | ITGB2, ITGB3 |
| HLJDD-moudle 13 | 1.0 Global and overview maps | hsa01212:Fatty acid metabolism | 4.73E-05 | SCD, ACACA, FASN |
| HLJDD-moudle 13 | 3.2 Signal transduction | hsa04152:AMPK signaling pathway | 3.09E-04 | SCD, ACACA, FASN |
| HLJDD-moudle 13 | 1.3 Lipid metabolism | hsa00061:Fatty acid biosynthesis | 0.003759 | ACACA, FASN |
| HLJDD-moudle 13 | 5.2 Endocrine system | hsa04910:Insulin signaling pathway | 0.039546 | ACACA, FASN |

Supplementary table 5. The enriched KEGG pathways and their categories of HLJDD-modules.
